# Desymmetrization of *C*_2_‐Symmetric Bis(Boronic Esters) by Zweifel Olefinations

**DOI:** 10.1002/chem.202000599

**Published:** 2020-06-03

**Authors:** Yannick Linne, Axel Schönwald, Sebastian Weißbach, Markus Kalesse

**Affiliations:** ^1^ Institute for Organic Chemistry Gottfried Wilhelm Leibniz Universität Hannover Schneiderberg 1B 30167 Hannover Germany; ^2^ Centre of Biomolecular Drug Research (BMWZ) Gottfried Wilhelm Leibniz Universität Hannover Schneiderberg 38 30167 Hannover Germany; ^3^ Helmholtz Centre for Infection Research (HZI) Inhoffenstrasse 7 38124 Braunschweig Germany

**Keywords:** *C*_2_-symmetry, desymmetrization, lithiation–borylation chemistry, mono-Zweifel olefination, natural product synthesis

## Abstract

*anti*‐Configured 1,3‐dimethyl deoxypropionate motifs are important sub structures in natural products. Herein, we describe a bidirectional approach for the rapid construction of natural products featuring such motifs by using *C*_2_‐symmetrical 1,3‐bis(boronic esters). As for its application in convergent syntheses it was important to establish a selective mono‐Zweifel olefination we describe the scope and limitations by using different 1,3‐bis(boronic esters) and nucleophiles. This protocol takes advantage of the combination of the Hoppe–Matteson–Zweifel chemistry, which was elegantly put into practice by Aggarwal et al. In order to show its applicability the total syntheses of two natural products, serricornin and (+)‐invictolide, were performed.

## Graphical abstract

**It's a crab**! By combining the Hoppe, Matteson, Aggarwal, and Zweifel chemistry, a bidirectional access to polyketides featuring the challenging 1,3‐deoxypropionate motif is presented. The desymmetrization of various 1,3‐bis(boronic esters) led to synthetically interesting building blocks. With this reliable method the pheromones invictolide and serricornin were synthesized.

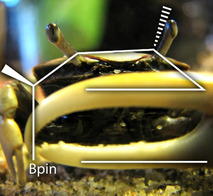

In the context of our program to establish synthetic access to various natural products^1^ we focused on those featuring the 1,3‐(poly)deoxypropionate motif. Polyketides and polyketide–peptide hybrids featuring this motif like sambutoxin (**1**), borrelidin (**2**), (+)‐kalkitoxin (**3**), and (+)‐invictolide (**4**) are challenging to synthesize without functional group interconversions by using established aldol chemistry (Figure 1).^2^

**Figure 1:**
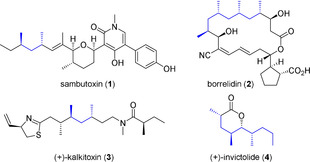
Natural products containing the 1,3‐deoxypropionate motif.

On the other hand, an elegant and rapid concept for the synthesis of those polydeoxypropionates is the so called assembly line synthesis developed by Aggarwal and co‐workers by using boronic esters.^3^ This method was applied to the total synthesis of (+)‐hydroxyphthioceranic acid, (+)‐kalkitoxin (**3**),2c and many more natural products.^6^ One advantage of this strategy, namely the individual control of each chiral center, is also a drawback as it requires a linear work flow and an iterative introduction of every single methyl or ethyl group.2c, 4 Based on this state of development and the fact that a large variety of natural products feature *anti*‐configured 1,3‐dimethyl deoxypropionate motifs, we envisioned to use *C*_2_‐symmetrical 1,3‐bis(boronic esters) for the sequential Zweifel olefination with different nucleophiles. The synthesis of such a precursor was already described by the group of Aggarwal^5^ and recently, Aggarwal and co‐workers subjected this 1,3‐bis(boronic ester) to a double‐sided vinylidene homologation.^6^ However, our goal was to use this *C*_2_‐symmetrical 1,3‐bis(boronic esters) in desymmetrizing mono‐Zweifel olefinations (Scheme 1).^7^

**Scheme 1:**
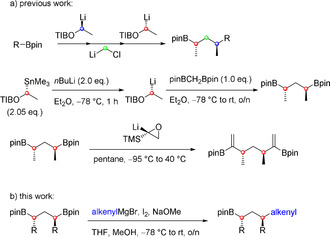
Desymmetrization of *C*_2_‐symmetric 1,3‐bis(boronic esters) by mono‐Zweifel olefination (pin=pinacolato, TIB=2,4,6‐triisopropylbenzoyl, TMS=trimethylsilyl).

At the outset of our investigation we started by optimizing the reaction conditions for the addition of simple vinyl metal species to the 1,3‐bis(boronic ester) **5 a** 5 (Scheme 2). First transformations with vinyl lithium^8^ generated yields around 35 %, but during the upscaling process the yield dropped drastically to 10 % or less (Scheme 2). Subsequently, vinylmagnesium bromide was used as the nucleophile^9^ and the yields were around 25 %. We then further optimized the mono‐Zweifel olefination by adding iodine as a solid^10^ and not as a methanolic solution. The subsequent methanol addition was done with a very slow addition rate of 0.15 mL min^−1^. This variation gave satisfactory and reliable yields around 48 and 94 % based on recovered starting material and works equally well for small and large scale reactions (for details see the Supporting Information). It should be pointed out that separation of the starting material and the olefination product was easily achievable upon standard column chromatography.

**Scheme 2:**
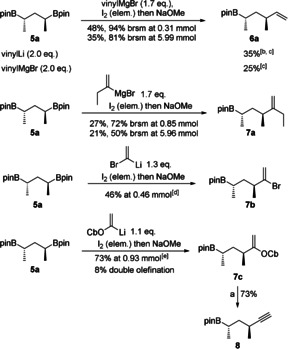
Optimized conditions for the mono‐Zweifel olefination. Standard reaction conditions: **5 a** (1.0 equiv), vinyl compound (1.1 to 2.0 equiv), THF, 30 min at −78 °C, then 30 min at RT, I_2_ (elem., 4.0 equiv), MeOH (0.15 mL min^−1^) 30 min at −78 °C, then NaOMe (8.0 equiv) in MeOH (0.50 mL min^−1^) at −78 °C, 30 min, then RT, 16 h. [b] Yield dropped to 10 % during upscaling. [c] I_2_ addition as a methanolic solution. [d] Ate‐complex formation and I_2_ addition at −95 °C. [e] Ate‐complex formation from −78 °C to 0 °C. a) Lithium diisopropylamide (LDA), Et_2_O, THF, −78 °C to 0 °C, 1.5 h, 73 %. **6 a**, **7 a**, **7 b**, and **7 c** were obtained as single diastereoisomers. Cb = *N*,*N*‐diisopropyl carbamoyl.

After having successfully accomplished the addition of vinylmagnesium bromide we turned our attention to other olefins that could be useful intermediates in total synthesis (Scheme 2). For this we used but‐1‐en‐2‐ylmagnesium bromide^11^ and obtained, by using the previously optimized conditions, the corresponding product **7 a**. As we will describe below, this product can be easily converted to its corresponding ethyl ketone. We also investigated the addition of different lithiated enol ethers,8b, 9, 12 however, only the lithiated vinyl carbamate led to the desired Zweifel olefination product **7 c**. In the beginning, we obtained the double‐olefination product in favor of the mono‐olefination but we overcame this problem by reducing the equivalents of the vinyl species to only 1.1 equivalents and obtained the desired mono‐Zweifel product **7 c** in 73 % yield. Beyond that, lithiated vinyl bromide was used as nucleophile giving us the versatile boronic ester **7 b** in 46 % yield. Interestingly, all of these nucleophiles were more reactive than vinylmagnesium bromide because unreacted starting material could not be re‐isolated. To show the utility of these products, mono‐Zweifel product **7 c** was subjected to an elimination under basic conditions affording alkyne **8**. In our case higher yields were observed when LDA (73 %) was used instead of *t*BuLi (41 %, Scheme 2). In both cases no epimerization was observed.^12^ Alkyne **8** could be a powerful intermediate in total synthesis after hydrometalation, cross‐coupling or even additional hydroboration.

After examination of different nucleophiles we investigated structural variations of the 1,3‐bis(boronic esters, Scheme 3). The sterically demanding isobutyl and cyclohexyl residues were well tolerated by the mono‐Zweifel olefination, which is quite remarkable due to the steric hindrance next to the reaction center.^7^, 8b Boronic esters containing terminal double bonds (**6 g**–**6 h**) could also be prepared in good yields. Due to their additional functionalities they could be valuable building blocks in total synthesis. Compounds **6 i** and **6 j** could also be synthesized by using the mono‐Zweifel olefination, but partial epimerization was observed. In the case of boronic ester **6 k** we observed traces of the double‐olefination product.

**Scheme 3:**
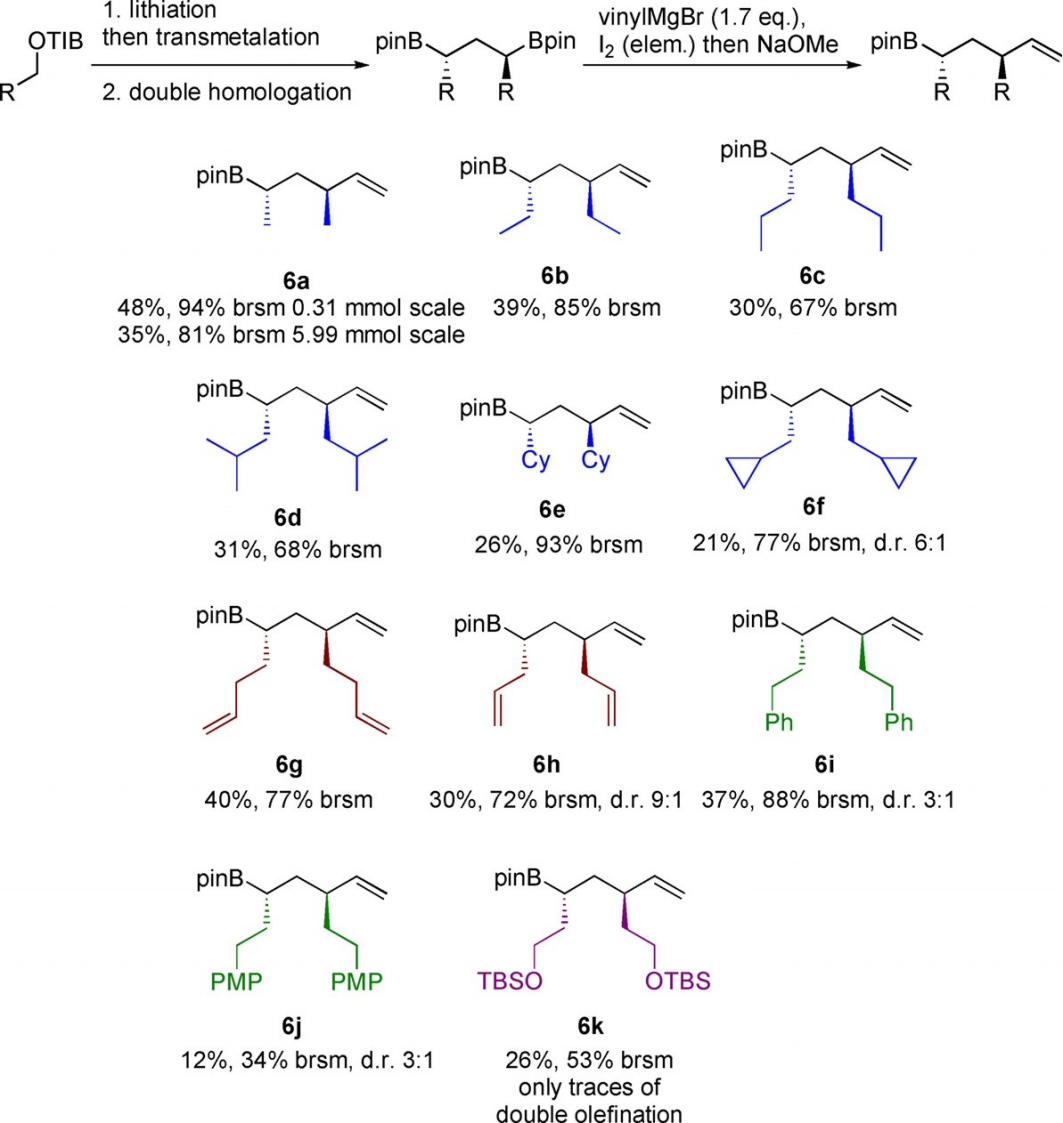
Substrate scope of the mono‐Zweifel olefination. All reactions were run by using the optimized conditions of Scheme 2. Yields refer to isolated products after flash column chromatography. The diastereomeric ratio (d.r.) was determined by ^1^H NMR spectroscopy (brsm=based on recovered starting material).

With a reliable method in hand, we applied building block **6 a** to the total synthesis of (+)‐invictolide (**4**) and serricornin (**20**).2d, 2e, 2f, 13 (−)‐Invictolide is one of the lactons used for the queen recognition of the red fire ant *Solenopsis invicta (Buren)*.^13^ With its three methyl groups and the 1,3‐*anti*‐deoxypropionate motif (+)‐invictolide (**4**) matches perfectly our synthetic strategy. In our retrosynthetic analysis (Scheme 4) we envisioned lactol formation and oxidation as the last steps, whereby the double bond of the Zweifel olefination functions as precursor for the carbonyl moiety. The required secondary alcohol in the linear precursor **9** should be installed by lithiation–borylation chemistry from the mono‐Zweifel product **6 a** and the TIB ester **10**. The corresponding alcohol could either be derived from 1‐propylboronic acid pinacol ester (**17**) by assembly line synthesis or by Myers alkylation by using auxiliary **14**. We on purpose performed both routes in order to compare steps and yields and by doing so obtaining an assessment of both strategies.

**Scheme 4:**
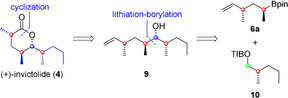
Retrosynthetic analysis of (+)‐invictolide (4).

Our synthesis started with the gram‐scale preparation of the *C*_2_‐symmetric 1,3‐bis(boronic ester) **5 a** 5 by using a slightly modified protocol of the Aggarwal group (see the Supporting Information). The described mono‐Zweifel olefination gave us boronic ester **6 a** in an overall yield of 45 %^14^ (Scheme 5).

**Scheme 5:**
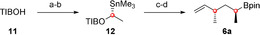
Synthesis of boronic ester **6 a**. a) Bromoethane, *n*Bu_4_NHSO_4_, NaOH, CHCl_3_, H_2_O, RT, o/n, ≥95 %. b) *s*BuLi, (+)‐sparteine, Et_2_O, −78 °C, 5 h, then Me_3_SnCl, Et_2_O, −78 °C to RT, 1.5 h, 70 %, enantiomeric ratio (e.r.) 99:1. c) *n*BuLi, Et_2_O, −78 °C, 1.5 h, then pinBCH_2_Bpin, Et_2_O, −78 °C to RT, o/n, 82 %, d.r. ≥95:5. d) VinylMgBr, THF, 30 min at −78 °C, then 30 min at RT, then I_2_, MeOH 30 min at −78 °C, then NaOMe, MeOH, 30 min at −78 °C, then RT, overnight, 35 %, 81 % brsm.

Alcohol **15** was synthesized through two different routes: Myers alkylation and assembly line synthesis (Scheme 6). The first step in the Myers alkylation route was the preparation of the auxiliary **14** from (−)‐pseudoephedrine (**13**).^15^ Afterwards, alkylation by using 1‐propyliodide (**16**) and subsequent reduction finished the synthesis of alcohol **15**.15b, 16 The assembly line route started with the synthesis of 1‐propylboronic acid pinacol ester (**17**) from 1‐propyliodide (**16**).4b Subsequently, a three‐step sequence consisting of lithiation–borylation, Matteson homologation, and oxidation led to alcohol **15**.2c This alcohol was then subjected to a Mitsunobu reaction^17^ by using TIBOH (**11**) to afford TIB ester **10** in 92 % yield.

**Scheme 6:**
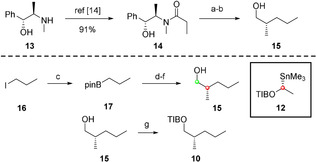
Synthesis of TIB ester **10** by Myers alkylation and assembly line synthesis. a) LDA, LiCl, THF, −78 °C to RT, 1.5 h, then 1‐propyliodide (**16**), THF, 0 °C to RT, overnight. b) LDA, BH_3_ **⋅**NH_3_, THF, −78 °C to RT, 5 h, 53 % over 2 steps, e.r. 99:1. c) *t*BuLi, pinBO*i*Pr, Et_2_O, −105 °C, 10 min, 88 %. d) *n*BuLi, **12**, Et_2_O, −78 °C to RT, overnight. e) BrCH_2_Cl, *n*BuLi, Et_2_O, −78 °C to RT, overnight. f) 2 m NaOH, H_2_O_2_, THF, −20 °C to RT, 2 h, 45 % over 3 steps, e.r. 99:1. g) TIBOH (**11**), PPh_3_, diisopropyl azodicarboxylate (DIAD), THF, 0 °C to RT, overnight, 92 %.

Comparison of the two different routes showed that the stereoselectivity was excellent (e.r. 99:1) in both approaches. One step less is needed in the Myers route (three vs. four) whereby the yields are within the same range. However, 1‐propylboronic acid pinacol ester (**17**) is also commercially available but quite expensive. However, in the case **17** is purchased three steps would be needed in both approaches. One drawback of the Myers route is the use of an auxiliary and the related low atom economy. Another difference is the number of purification steps, with the Myers route requiring three and the assembly line approach requiring two. A practical disadvantage of the assembly line approach is the required very slow addition rate (20 μL min^−1^) of *n*BuLi during the Matteson homologation.

However, with both fragments in hand, the fragment coupling by using lithiation–borylation chemistry was the next step. The obtained boronic ester was directly subjected to an oxidation giving us secondary alcohol **9** in good yield and high diastereoselectivity.^18^ The subsequent two‐step sequence consisting of ozonolysis^19^ and Ley–Griffith oxidation^20^ of the intermediately formed lactol finished the—to the best of our knowledge—shortest total synthesis of (+)‐invictolide (**4**, Scheme 7).

**Scheme 7:**
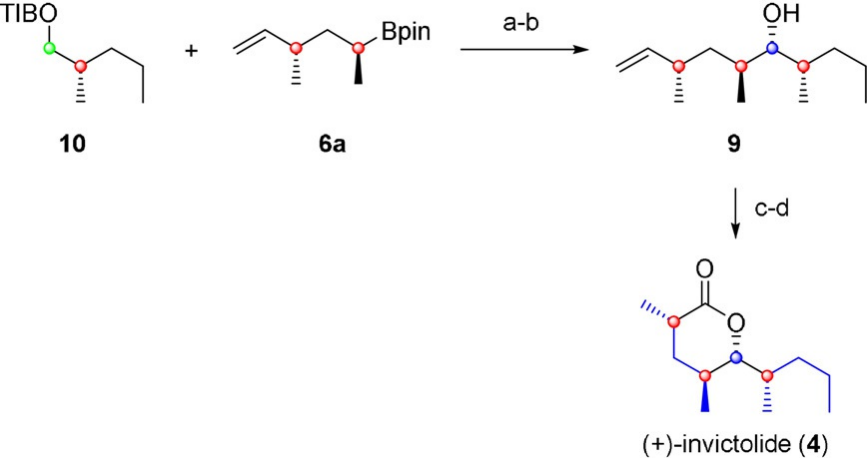
Fragment coupling and endgame in the total synthesis of (+)‐invictolide (**4**). a) *s*BuLi, (−)‐sparteine, Et_2_O, −78 °C, 5 h, then **6 a**, THF, −78 to 40 °C, overnight. b) 2 m NaOH, H_2_O_2_, THF, −20 °C to RT, 2 h, 62 % over 2 steps, d.r. ≥95:5. c) O_3_, PPh_3_, CH_2_Cl_2_, −78 °C to RT, overnight, 87 %. d) Tetrapropylammonium perruthenate (TPAP), *N*‐methylmorpholine *N*‐oxide (NMO), 4 Å molecular sieve (MS), CH_2_Cl_2_, RT, 2.5 h, 80 %.

By using the same strategy we also accomplished the synthesis of serricornin (**20**), the female sex pheromone of the cigarette beetle *Lasioderma serricorne*.^21^ TIB ester **18** was prepared by using established phase‐transfer conditions.^5^, ^22^ Lithiation–borylation chemistry of boronic ester **7 a** with TIB ester **18** 5, 22 and subsequent oxidation gave us secondary alcohol **19** in 70 % yield over two steps in a 10:1 diastereomeric ratio. Finally, ozonolysis^19^ provided serricornin (**20**) as a mixture of its open‐chain and hemiketal form (Scheme 8).^11^

**Scheme 8:**
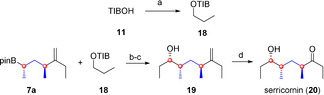
Total synthesis of serricornin (**20**). a) NaOH, 1‐bromopropane, *n*Bu_4_NHSO_4_, H_2_O, CHCl_3_, RT, overnight, ≥95 %. b) *s*BuLi, (+)‐sparteine, Et_2_O, −78 °C, 5 h, then **7 a**, THF, −78 to 40 °C, overnight; c) 2 m NaOH, H_2_O_2_, THF, −20 °C to RT, 2.5 h, 70 % over 2 steps, d.r. 10:1. d) O_3_, PPh_3_, CH_2_Cl_2_, −78 °C to RT, overnight, 74 %, d.r. 18:1 hemiketal form.

In summary, we developed a bidirectional approach to polyketides or polyketide fragments featuring the 1,3‐deoxypropionate motif. The key step in this approach is a desymmetrization by using the mono‐Zweifel olefination. We could show the value of key intermediates **6 a** and **7 a** in the syntheses of (+)‐invictolide (**4**) and serricornin (**20**). Furthermore, a detailed substrate scope showed the general applicability of the developed methodology. Finally, the use of different nucleophiles enables synthetic access to a variety of valuable building blocks.

## Conflict of interest

The authors declare no conflict of interest.

## Supporting information

As a service to our authors and readers, this journal provides supporting information supplied by the authors. Such materials are peer reviewed and may be re‐organized for online delivery, but are not copy‐edited or typeset. Technical support issues arising from supporting information (other than missing files) should be addressed to the authors.

Supplementary
