# Supplementary material for: Desymmetrization of C 2‐Symmetric Bis(Boronic Esters) by Zweifel Olefinations
Source: Chemistry. 2020 Jun 3;26(36):7998–8002. doi: 10.1002/chem.202000599 (PMC7384159; doi:10.1002/chem.202000599)
Supplement: Supplementary file 1 — Supplementary [file CHEM-26-7998-s001.pdf]

Chemistry–A European Journal

Supporting Information

Desymmetrization of C_2-Symmetric Bis(Boronic Esters) by Zweifel Olefinations

Yannick Linne,[a, b] Axel Schönwald,[a] Sebastian Weißbach,[a] and Markus Kalesse*[a, b, c]

Table of Contents

1 General methods 3

2 Optimization of mono-Zweifel olefination 4

3 Experimental procedures 5

3.1 General procedure A: TIB ester 5

3.2 General procedure B: TIB ester 8

3.3 General procedure C: stannane 10

3.4 General procedure D: C_2-symmetric 1,3-bis(boronic ester) 17

3.5 General procedure E: mono-Zweifel olefination 21

3.6 Follow-Up chemistry 29

3.7 Synthesis of (+)-invictolide 31

3.8 Synthesis of serricornin 36

4 References 38

5 NMR Spectra 39

1 General methods

Unless otherwise noted all reactions were carried out under an argon atmosphere using a Drierite™ gas-drying unit. The used glassware was flame dried under high vacuum. Air- and moisture-sensitive liquids and solutions were transferred via syringe flushed with argon prior to use. All reagents were purchased from commercial suppliers and used without further purification unless otherwise noted. Stated temperatures, except room temperature, refer to bath temperatures.

Dry solvents Dichloromethane and all amine bases were distilled under an inert atmosphere over calcium hydride. Tetrahydrofuran, diethyl ether, methanol, toluene and DMF were purchased from Acros Organics over molecular sieves and under inert atmosphere. Benzene was bought from Sigma Aldrich.

Sparteine (+)-Sparteine was purchased from Chem-Impex and (−)-sparteine was bought from TCI. Both were distilled under high vacuum and stored under argon at −25 °C.

Thin layer chromatography All reactions were stirred magnetically and monitored using pre-coated TLC sheets ALUGRAM® Xtra SIL G/UV_{254} (0.2 mm, silica gel, F_{254}, aluminum-backed, MACHEREY-NAGEL) with detection by UV light (λ = 254 nm) and/or by staining with either basic potassium permanganate, acidic ceric ammonium molybdate, acidic anisaldehyde or acidic vanillin stain.

Flash column chromatography was performed using silica gel (0.04-0.063 mm, 240-400 mesh) obtained from MACHEREY-NAGEL. The applied petroleum ether fraction had a bp of 40-60 °C. The eluent is given in volume ratios (v/v).

^{1}H-NMR experiments were recorded in $CDCl_3$ or C_6D_6 using either a DPX 400 (Bruker), an AMX 400 (Bruker), an Ascend 400 Avance III HD (Bruker) or an Ascend 600 MHz (Bruker). The spectra were calibrated using the residual solvent peak: $\delta(CDCl_3)$ = 7.26 ppm, $\delta(C_6D_6)$ = 7.16 ppm. Chemical shift δ is given in parts per million (ppm), coupling constant J in hertz (Hz) and multiplicity as follows: s, singlet; d, doublet; t, triplet; q, quadruplet; p, pentett; sex, sextet; sep, septet; m, multiplet; m_c, centered multiplet; bs, broad signal or combination of these acronyms. NMR spectra were processed using TopSpin (Bruker).

^{13}C-NMR experiments were recorded in $CDCl_3$ or C_6D_6 using either a DPX 400 (Bruker), an AMX 400 (Bruker), an Ascend 400 Avance III HD (Bruker) or an Ascend 600 MHz (Bruker). The spectra were calibrated using the residual solvent peak: $\delta(CDCl_3)$ = 77.16 ppm, $\delta(C_6D_6)$ = 128.06 ppm. Chemical shift δ is given in parts per million (ppm). NMR spectra were processed using TopSpin (Bruker).

High Resolution Mass Spectra (HRMS) were obtained either using a Q-Tof Premier (Waters), a LCT Premier (Waters) or a GC-system Agilent 6890 coupled with an Agilent 5973. Both the masses found and the masses calculated are given.

Optical rotation $[\alpha]_D^{20}$ were measured either on a P3000 polarimeter (A. Krüss Optronic, λ = 589 nm), a Perkin-Elmer 341 (λ = 589 nm) or a Perkin-Elmer 241 MC (λ = 365 nm). The sample concentration (in g/100 mL) is given with every single experiment.

Melting points were determined in °C using an OptiMelt MPA 100 (Stanford Research System).

Chiral HPLC was performed on a Merck/Hitachi L-7150 system with a Merck/Hitachi L-7400 UV-detector using a Daicel Chiralcel® OD-H column (4.6 x 250 mm, 5 µm). Further information can be found in the individual procedure.

Chiral GC was performed on a HP 5890-II system (Hewlett-Packard) with a FID using a MACHEREY-NAGEL Hydrodex-β-6TBDM GC capillary column (25 m x 0.25 mm x 0.25 µm). Further information can be found in the individual procedure.

2 Optimization of mono-Zweifel olefination

To a stirred solution of 1,3-bis(boronic ester) **5a** (1.0 eq.) in solvent (0.2 M) at −78 °C, was added vinylspecies (1.7 eq., 0.5 mL/min) and stirring was continued for 30 min at this temperature. Then the solution was warmed to rt and stirred for further 30 min. After cooling to −78 °C electrophile (4.0 eq.) was added in two portions over a period of 5 min. Anhydrous MeOH (0.5 M, 0.15 mL/min) was added to the dark solution and the reaction mixture was stirred for 30 min. Then a suspension of NaOMe (8.0 eq., 0.5 mL/min) in anhydrous MeOH (1 M) was added and the red reaction mixture was stirred for further 30 min at −78 °C. After warming to rt the black reaction mixture was stirred overnight. MTBE and sat. aq. $Na_2S_2O_3$ were added until the dark color disappeared. The phases were separated and the aqueous phase was extracted with MTBE (3x). The combined organic phases were washed with sat. aq. NaCl and dried over Na_2SO_4, concentrated *in vacuo* and the crude material was purified by flash column chromatography to afford mono-Zweifel product **6a**.

Table 1. Selected optimization results concerning the nucleophile and equivalents.

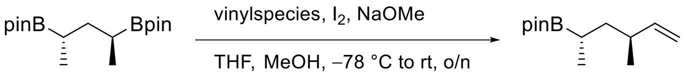

5a **6a**

#	conditions	yield [%][a]
**1**	vinylLi (2.0 eq.)	35[b]
**2**	vinylMgBr (2.0 eq.)	25
**3**	vinylMgBr (1.3 eq.) & elementary I_2 addition[c]	35, 94 brsm
**4**	**vinylMgBr (1.7 eq.) & elementary I_2 addition[c]**	**48, 94 brsm[d]**
**5**	vinylMgBr (2.0 eq.) & elementary I_2 addition[c]	35, 46 brsm
**6**	vinylMgBr (3.0 eq.) & elementary I_2 addition[c]	36

[a] Isolated yield after flash column chromatography. [b] yield dropped to 10% during upscaling. [c] MeOH addition (0.15 mL/min). [d] 35%, 81% brsm at 5.99 mmol scale.

Table 2. Selected optimization results concerning the electrophile.

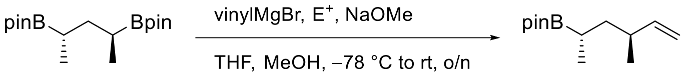

5a **6a**

#	electrophile	yield [%][a]
**1**	**I_2**	**48, 94 brsm[b]**
**2**	NIS	36, 92 brsm
**3**	Br_2	no rxn
**4**	BrCN[c]	traces

[a] Isolated yield after flash column chromatography. [b] 35%, 81% brsm at 5.99 mmol scale. [c] 2.6 eq. in CH_2Cl_2 at 0 °C to 40 °C for 3 h.

Table 3. Selected optimization results concerning the solvent.

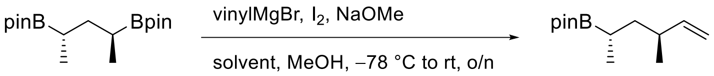

5a **6a**

#	solvent	yield [%][a]
**1**	**THF**	**48, 94 brsm[b]**
**2**	Me-THF	32, 35 brsm
**3**	MTBE	18, 32 brsm
**4**	CPME	23, 64 brsm
**5**	Et_2O	traces
**6**	THF/DMSO (1/1)	traces

[a] Isolated yield after flash column chromatography. [b] 35%, 81% brsm at 5.99 mmol scale.

3 Experimental Procedures

3.1 General procedure A: TIB ester[1]

The required primary alcohol (1.1 eq.) was dissolved in anhydrous THF (0.3 M), PPh_3 (1.0 eq.) and TIBOH (**11**, 1.0 eq.) were added successively. After cooling to 0 °C, DIAD (1.1 eq., 0.12 mL/min) was added, the reaction mixture was slowly warmed to rt and stirred overnight at that temperature. MTBE and sat. aq. $NaHCO_3$ were added and the phases separated. The aqueous phase was extracted with MTBE (3x), the organic layers combined and dried over Na_2SO_4. The solvent was removed *in vacuo* to leave a crude oil, which was then triturated with PE. The white suspension was filtered through a short plug of silica using PE/EtOAc (9:1) as eluent. The solvent was removed under reduced pressure and the crude TIB ester was further purified by flash column chromatography.

TIB ester 21

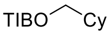

21
$C_{23}H_{36}O_2$
344.54

Using GPA and cyclohexylmethanol (0.82 mL, 759 mg, 6.64 mmol) gave TIB ester **21** (1.67 g, 4.85 mmol, 80%) after purification by flash column chromatography (PE:EtOAc 99:1) as a yellow oil.

^{1}H-NMR (400 MHz, $CDCl_3$): δ = 7.00 (s, 2H), 4.11 (d, J = 6.4 Hz, 2H), 2.95-2.79 (m, 3H), 1.83-1.63 (m, 6H), 1.30-1.15 (m, 21H), 1.09-0.98 (m, 2H) ppm;

^{13}C-NMR (101 MHz, $CDCl_3$): δ = 171.3, 150.1, 144.8, 131.0, 121.0, 70.5, 37.2, 34.6, 31.7, 29.9, 26.5, 25.8, 24.3, 24.1 ppm;

HRMS (ESI): $C_{23}H_{36}O_2Na$ $[M+Na]^+$ calculated: 367.2613, found: 367.2617;

R$_f$ = 0.6 (PE:MTBE 95:5, UV, $KMnO_4$).

TIB ester 22

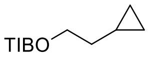

22
$C_{21}H_{32}O_2$
316.49

Using GPA and 2-cyclopropylethan-1-ol (750 mg, 8.71 mmol) gave TIB ester **22** (1.75 g, 5.53 mmol, 70%) after purification by flash column chromatography (PE:EtOAc 99:1) as a colorless oil.

^{1}H-NMR (400 MHz, $CDCl_3$): δ = 7.00 (s, 2H), 4.36 (t, J = 6.9 Hz, 2H), 2.92-2.83 (m, 3H), 1.63 (q, J = 6.9 Hz, 2H), 1.24 (d, J = 6.9 Hz, 18H), 0.84-0.74 (m, 1H), 0.50-0.45 (m, 2H), 0.13-0.10 (m, 2H) ppm;

^{13}C-NMR (101 MHz, $CDCl_3$): δ = 171.2, 150.2, 144.9, 130.8, 121.0, 65.3, 34.6, 33.7, 31.6, 24.3, 24.1, 7.9, 4.4 ppm;

HRMS (ESI): $C_{21}H_{32}O_2Na$ $[M+Na]^+$ calculated: 339.2300, found: 339.2299;

R*f* = 0.5 (PE:MTBE 95:5, UV, $KMnO_4$).

TIB ester 23

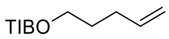

23
$C_{21}H_{32}O_2$
316.49

Using GPA and 4-penten-1-ol (3.0 mL, 2.55 g, 29.6 mmol) gave TIB ester **23** (7.25 g, 22.9 mmol, 85%) after purification by flash column chromatography (PE:EtOAc 99:1) as a yellow oil.

^{1}H-NMR (400 MHz, $CDCl_3$): δ = 7.01 (s, 2H), 5.87-5.77 (m, 1H), 5.08-4.99 (m, 2H), 4.31 (t, J = 6.6 Hz, 2H), 2.93-2.80 (m, 3H), 2.22-2.15 (m, 2H), 1.87-1.79 (m, 2H), 1.247 (d, J = 6.9 Hz) and 1.244 (d, J = 6.9 Hz, 18H) ppm;

^{13}C-NMR (101 MHz, $CDCl_3$): δ = 171.1, 150.2, 144.9, 137.5, 130.8, 121.0, 115.6, 64.4, 34.6, 31.6, 30.3, 28.0, 24.3, 24.1 ppm;

HRMS (ESI): $C_{21}H_{32}O_2Na$ $[M+Na]^+$ calculated: 339.2300, found: 339.2299;

R*f* = 0.6 (PE:MTBE 95:5, UV, $KMnO_4$).

Analytical data are in accordance with the literature.[2]

TIB ester 24

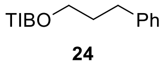

24
$C_{25}H_{34}O_2$
366.55

Using GPA and hydrocinnamyl alcohol (1.2 mL, 1.19 g, 8.72 mmol) gave TIB ester **24** (1.72 g, 4.69 mmol, 59%) after purification by flash column chromatography (PE:EtOAc 99:1) as a yellow oil.

^{1}H-NMR (400 MHz, $CDCl_3$): δ = 7.33-7.27 (m, 2H), 7.24-7.17 (m, 3H), 7.03 (s, 2H), 4.34 (t, J = 6.5 Hz, 2H), 2.96-2.83 (m, 3H), 2.77-2.73 (m, 2H), 2.10-2.03 (m, 2H), 1.269 (d, J = 6.9 Hz) and 1.258 (d, J = 6.9 Hz, 18H) ppm;

^{13}C-NMR (101 MHz, $CDCl_3$): δ = 171.1, 150.3, 144.9, 141.3, 130.7, 128.64, 128.56, 126.2, 121.0, 64.4, 34.6, 32.5, 31.7, 30.5, 24.3, 24.1 ppm;

HRMS (ESI): $C_{25}H_{34}O_2Na$ $[M+Na]^+$ calculated: 389.2457, found: 389.2458;

R$_f$ = 0.5 (PE:MTBE 95:5, UV, $KMnO_4$).

Analytical data are in accordance with the literature.[1]

TIB ester 25

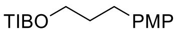

25
$C_{26}H_{36}O_3$
396.57

Using GPA and 3-(4-methoxyphenyl)propan-1-ol (2.00 g, 12.0 mmol) gave TIB ester **25** (4.02 g, 10.1 mmol, 93%) after purification by flash column chromatography (PE:MTBE 98:2) as a yellow oil.

^{1}H-NMR (400 MHz, $CDCl_3$): δ = 7.14-7.08 (m, 2H), 7.02 (s, 2H), 6.87-6.82 (m, 2H), 4.32 (t, J = 6.5 Hz, 2H), 3.79 (s, 3H), 2.94-2.82 (m, 3H), 2.73-2.65 (m, 2H), 2.06-1.97 (m, 2H), 1.262 (d, J = 6.9 Hz) and 1.253 (d, J = 6.9 Hz, 18H) ppm;

^{13}C-NMR (101 MHz, $CDCl_3$): δ = 171.1, 158.1, 150.2, 144.9, 133.3, 130.7, 129.4, 121.0, 114.0, 64.4, 55.4, 34.6, 31.7, 31.5, 30.7, 24.3, 24.1 ppm;

HRMS (ESI): $C_{26}H_{36}O_3Na$ $[M+Na]^+$ calculated: 419.2562, found: 419.2563;

R$_f$ = 0.4 (PE:MTBE 95:5, UV, $KMnO_4$).

Analytical data are in accordance with the literature.[1]

TIB ester 26

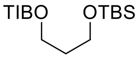

26
$C_{25}H_{44}O_3Si$
420.71

Using GPA and 3-*tert*-butyldimethylsilyloxy-1-propanol (2.00 g, 10.5 mmol) gave TIB ester **26** (3.02 g, 7.18 mmol, 75%) after purification by flash column chromatography (PE:EtOAc 100:1) as a colorless oil.

^{1}H-NMR (400 MHz, $CDCl_3$): δ = 7.00 (s, 2H), 4.40 (t, J = 6.4 Hz, 2H), 3.72 (t, J = 6.2 Hz, 2H), 2.94-2.80 (m, 3H), 1.93 (p, J = 6.3 Hz, 2H), 1.24 (d, J = 6.9 Hz, 18H), 0.90 (s, 9H), 0.05 (s, 6H) ppm;

^{13}C-NMR (101 MHz, $CDCl_3$): δ = 171.1, 150.2, 144.9, 130.8, 121.0, 62.1, 59.6, 34.6, 32.1, 31.6, 26.0, 24.3, 24.1, 18.4, −5.3 ppm;

HRMS (ESI): $C_{25}H_{44}O_3SiNa$ $[M+Na]^+$ calculated: 443.2957, found: 443.2956;

R$_f$ = 0.5 (PE:MTBE 95:5, UV, $KMnO_4$).

Analytical data are in accordance with the literature.[2]

TIB ester 10

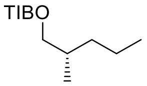

10
$C_{22}H_{36}O_2$
332.53

Using GPA and (*S*)-2-methylpentan-1-ol (**15**, 1.55 g, 15.2 mmol) gave TIB ester **10** (4.23 g, 12.7 mmol, 92%) after purification by flash column chromatography (PE:EtOAc 98:2) as a colorless oil.

^{1}H-NMR (400 MHz, $CDCl_3$): δ = 7.01 (s, 2H), 4.14 (m$_c$, 2H), 2.95-2.79 (m, 3H), 1.94-1.82 (m, 1H), 1.48-1.28 (m, 4H), 1.24 (d, *J* = 7.0 Hz, 18H), 0.97 (d, *J* = 6.7 Hz, 3H), 0.90 (t, *J* = 7.0 Hz, 3H) ppm;

^{13}C-NMR (101 MHz, $CDCl_3$): δ = 171.4, 150.1, 144.9, 131.0, 121.0, 70.1, 35.7, 34.6, 32.4, 31.7, 24.33, 24.32, 24.1, 20.1, 17.1, 14.4 ppm;

HRMS (ESI): $C_{22}H_{36}O_2Na$ $[M+Na]^+$ calculated: 355.2613, found: 355.2612;

R*f* = 0.3 (PE:EtOAc 98:2, UV, $KMnO_4$);

$[\alpha]_D^{20}$ = −3.8 (*c* 1.0, $CHCl_3$).

3.2 General procedure B: TIB ester

Following Beak's procedure[3], a solution of TIBOH (**11**, 1.0 eq.) in $CHCl_3$ (0.2 M) was treated with a solution of NaOH (3.1 eq.) and *n*Bu_4NHSO_4 (8 mol%) in H_2O (0.8 M). After addition of the required alkyl halide (5.0 eq.) the biphasic reaction mixture was stirred overnight at rt. The phases were separated and the aqueous phase was extracted with CH_2Cl_2 (3x). The combined organic phases were dried over Na_2SO_4 and concentrated *in vacuo*. The resulting oil was filtered through a short plug of silica using PE:EtOAc (9:1) as eluent. The solvent was removed under reduced pressure to afford the corresponding TIB ester.

TIB ester 27

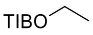

27
$C_{18}H_{28}O_2$
276.42

Using GPB and ethyl bromide (15.0 mL, 21.9 g, 201 mmol) gave TIB ester **27** (10.8 g, 38.9 mmol, ≥ 95%) as a yellow oil.

^{1}H-NMR (400 MHz, $CDCl_3$): δ = 7.00 (s, 2H), 4.37 (q, *J* = 7.1 Hz, 2H), 2.87 (m$_c$, 3H), 1.37 (t, *J* = 7.1 Hz, 3H), 1.250 (d, *J* = 7.0 Hz) and 1.242 (d, *J* = 7.0 Hz, 18H) ppm;

^{13}C-NMR (101 MHz, $CDCl_3$): δ = 171.0, 150.2, 144.9, 130.8, 121.0, 60.9, 34.6, 31.6, 24.3, 24.1, 14.4 ppm;

HRMS (ESI): $C_{18}H_{28}O_2Na$ $[M+Na]^+$ calculated: 299.1987, found: 299.1986;

R*f* = 0.6 (PE:EtOAc 10:1, UV, $KMnO_4$).

Analytical data are in accordance with the literature.[4]

TIB ester 18

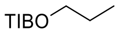

18
$C_{19}H_{30}O_2$
290.45

Using GPB and 1-propyl bromide (9.2 mL, 12.4 g, 101 mmol) gave TIB ester **18** (5.74 g, 19.8 mmol, ≥ 95%) as a yellow oil.

^{1}**H-NMR** (400 MHz, $CDCl_3$): δ = 7.01 (s, 2H), 4.27 (t, J = 6.7 Hz, 2H), 2.94-2.81 (m, 3H), 1.76 (m_c, 2H), 1.247 (d, J = 6.8 Hz) and 1.244 (d, J = 6.9 Hz, 18H), 1.00 (t, J = 7.4 Hz, 3H) ppm;

^{13}**C-NMR** (101 MHz, $CDCl_3$): δ = 171.2, 150.2, 144.9, 130.9, 121.0, 66.8, 34.6, 31.6, 24.3, 24.1, 22.1, 10.7 ppm;

HRMS (ESI): $C_{19}H_{30}O_2Na$ $[M+Na]^+$ calculated: 313.2144, found: 313.2139;

$\mathbf{R_f}$**-Wert** = 0.2 (PE:MTBE 100:1, UV, $KMnO_4$).

Analytical data are in accordance with the literature.[1]

TIB ester 28

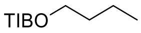

28
$C_{20}H_{32}O_2$
304.47

Using GPB and 1-butyl bromide (4.3 mL, 5.52 g, 40.3 mmol) gave TIB ester **28** (2.38 g, 7.82 mmol, ≥ 95%) as a yellow oil.

^{1}**H-NMR** (400 MHz, $CDCl_3$): δ = 7.00 (s, 2H), 4.30 (t, J = 6.7 Hz, 2H), 2.94-2.80 (m, 3H), 1.76-1.67 (m, 2H), 1.50-1.39 (m, 2H), 1.25 (m_c, 18H), 0.95 (t, J = 7.4 Hz, 3H) ppm;

^{13}**C-NMR** (101 MHz, $CDCl_3$): δ = 171.2, 150.2, 144.8, 130.9, 121.0, 64.9, 34.6, 31.6, 30.8, 24.3, 24.1, 19.4, 13.8 ppm;

HRMS (ESI): $C_{20}H_{32}O_2Na$ $[M+Na]^+$ calculated: 327.2300, found: 327.2300;

$\mathbf{R_f}$ = 0.6 (PE:MTBE 95:5, UV, $KMnO_4$).

TIB ester 29

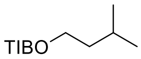

29
$C_{21}H_{34}O_2$
318.50

Using GPB and 1-bromo-3-methylbutane (4.8 mL, 6.08 g, 40.3 mmol) gave TIB ester **29** (2.24 g, 7.03 mmol, 87%) as a yellow oil.

^{1}**H-NMR** (400 MHz, $CDCl_3$): δ = 7.00 (s, 2H), 4.33 (t, J = 6.8 Hz, 2H), 2.86 (m_c, 3H), 1.76 (sep, J = 6.7 Hz, 1H), 1.61 (dt, J = 6.9 Hz, 6.8 Hz, 2H), 1.24 (d, J = 6.9 Hz, 18H), 0.95 (d, J = 6.8 Hz, 6H) ppm;

^{13}**C-NMR** (101 MHz, $CDCl_3$): δ = 171.2, 150.2, 144.8, 130.9, 121.0, 63.6, 37.5, 34.6, 31.6, 25.1, 24.3, 24.1, 22.5 ppm;

HRMS (ESI): $C_{21}H_{34}O_2Na$ $[M+Na]^+$ calculated: 341.2457, found: 341.2459;

$\mathbf{R_f}$ = 0.7 (PE:MTBE 95:5, UV, $KMnO_4$).

Analytical data are in accordance with the literature.[5]

TIB ester 30

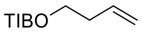

30
$C_{20}H_{30}O_2$
302.46

Using GPB and 4-bromo-1-butene (4.1 mL, 5.44 g, 40.3 mmol) gave TIB ester **30** (2.37 g, 7.84 mmol, ≥ 95%) as a yellow oil.

^{1}H-NMR (400 MHz, $CDCl_3$): *δ* = 7.00 (s, 2H), 5.84 (m_c, 1H), 5.15 (ddt, *J* = 17.2 Hz, 1.7 Hz, 1.6 Hz, 1H), 5.09 (ddt, *J* = 10.3 Hz, 1.6 Hz, 1.2 Hz, 1H), 4.37 (t, *J* = 6.8 Hz, 2H), 2.94-2.80 (m, 3H), 2.50 (m_c, 2H), 1.242 (d, *J* = 7.0 Hz) and 1.240 (d, *J* = 6.9 Hz, 18H) ppm;

^{13}C-NMR (101 MHz, $CDCl_3$): *δ* = 171.0, 150.2, 144.9, 134.2, 130.6, 121.0, 117.5, 64.2, 34.6, 33.2, 31.6, 24.3, 24.1 ppm;

HRMS (ESI): $C_{20}H_{30}O_2Na$ $[M+Na]^+$ calculated: 325.2144, found: 325.2144;

R*f* = 0.7 (PE:MTBE 95:5, UV, $KMnO_4$).

Analytical data are in accordance with the literature.[6]

3.3 General procedure C: stannane[2,4]

The required TIB ester (1.0 eq.) and (+)-sparteine (1.6 eq.) were dissolved in Et_2O (0.2 M). The solution was cooled to −78 °C, *s*BuLi (1.3 M in hexanes, 1.5 eq., 0.5 mL/min , color change: colorless → brown/purple) was added and the reaction mixture stirred for 5 h at this temperature. Then a freshly prepared solution of Me_3SnCl (1.7 eq.) in Et_2O (1.0 M) was added dropwise (color change: brown/purple → yellow/colorless) and the reaction mixture stirred for 1 h at −78 °C before being warmed to rt. After 30 min at rt 5% aq. H_3PO_4 was added and the biphasic mixture was stirred for further 20 min. The organic layer was separated and washed with 5% aq. H_3PO_4 (3x). The combined aqueous layers were extracted with Et_2O (3x). The organic layers were combined, dried over Na_2SO_4 and concentrated *in vacuo* to afford the desired stannane. Racemic samples were prepared by the use of TMEDA instead of (+)-sparteine.
Sparteine-recovery: After adjusting the pH of the combined aqueous phases to 11 by using aq. 2.0 M NaOH, they were extracted with Et_2O (3x). The organic layers were combined, dried over K_2CO_3 and concentrated *in vacuo*. The residue was then distilled under high vacuum over calcium hydride (100 mg/g) at 150 °C to afford the respective enantiomer of sparteine (70-80%) as a colorless oil, which solidified in the freezer.

Stannane 12

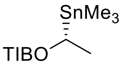

12
$C_{21}H_{36}O_2Sn$
439.23

Following a modified version of GPC using TIB ester **27** (5.00 g, 18.1 mmol, 1.0 eq.), (+)-sparteine (5.4 mL, 5.51 g, 23.5 mmol, 1.3 eq.), *s*BuLi (1.3 M in hexanes, 18.0 mL, 23.5 mmol, 1.3 eq.) and trimethyltin chloride (1.0 M in Et_2O, 24 mL, 23.5 mmol, 1.3 eq.) gave a slightly yellow solid, which was then recrystallized from MeOH (3 mL/g) to give stannane **12** (5.55 g, 12.7 mmol, 70%, *er* 99:1) as colorless needles.

^{1}H-NMR (400 MHz, $CDCl_3$): *δ* = 6.99 (s, 2H), 5.08-5.00 (m, 1H), 2.92-2.80 (m, 3H), 1.68-1.51 (m, 3H), 1.24 (d, *J* = 6.9 Hz, 18H), 0.18 (s, d, *J* = 54.2 Hz and *J* = 51.6 Hz, 9H) ppm;

^{13}C-NMR (101 MHz, $CDCl_3$): *δ* = 171.4, 150.1, 145.0, 130.9, 120.9, 67.2, 34.5, 31.5, 24.5, 24.2, 24.1, 19.4, −9.8 ppm;

HRMS (ESI):$C_{21}H_{36}O_2SnNa$ $[M+Na]^+$ calculated: 463.1635, found: 463.1634;

R_f = 0.9 (PE:EtOAc 95:5);

$[\alpha]_D^{20}$ = –42.0 (*c* 1.0, $CHCl_3$);

Chiral HPLC: (Daicel Chiracel® OD-H column (25 cm), hexanes, 0.7 mL/min, rt, 210 nm): t_R = 6.4 min (*S*), 14.6 min (*R*), *er* 1:99.

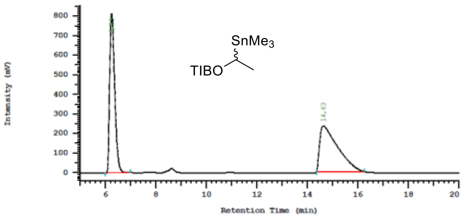

Peak Quantitation: AREA
Calculation Method: AREA%

No.	RT	Area	Conc 1	BC
1	6,25	11209370	49,178	BB
2	14,63	11584068	50,822	BB
		22793438	100,000	

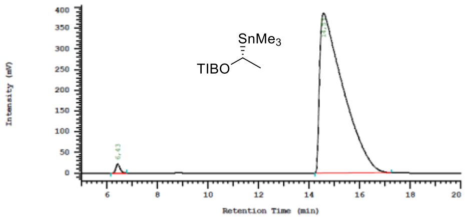

Peak Quantitation: AREA
Calculation Method: AREA%

No.	RT	Area	Conc 1	BC
1	6,43	251399	0,994	BB
2	14,57	25035472	99,006	BB
		25286871	100,000	

Analytical data are in accordance with the literature.[2,4]

Stannane 31

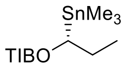

31
$C_{22}H_{38}O_2Sn$
453.25

According to GPC, TIB ester **18** (2.61 g, 8.98 mmol) gave stannane **31** (4.05 g, 8.94 mmol, ≥ 95%, *er* 94:6) as a colorless oil.

^{1}H-NMR (400 MHz, $CDCl_3$): δ = 6.99 (s, 2H), 4.95 (m_c, 1H), 2.86 (m_c, 3H), 1.96 (m_c, 2H), 1.24 (m_c, 18H), 1.01 (t, J = 7.3 Hz, 3H), 0.19 (s, d, J = 54.0 Hz and J = 51.6 Hz, 9H) ppm;

^{13}C-NMR (101 MHz, $CDCl_3$): δ = 171.6, 150.0, 145.0, 131.0, 121.0, 74.2, 34.5, 31.6, 27.0, 24.5, 24.3, 24.1, 12.4, −8.9 ppm;

HRMS (ESI):$C_{22}H_{38}O_2SnNa$ $[M+Na]^+$ calculated: 477.1791, found: 477.1794;

R_f = 0.3 (PE:MTBE 98:2, UV, $KMnO_4$);

$[\alpha]_D^{20}$ = –36.7 (*c* 1.1, $CHCl_3$);

Chiral HPLC: (Daicel Chiracel® OD-H column (25 cm), hexanes, 0.7 mL/min, rt, 210 nm): t_R = 6.1 min (*S*), 14.1 min (*R*), *er* 6:94.

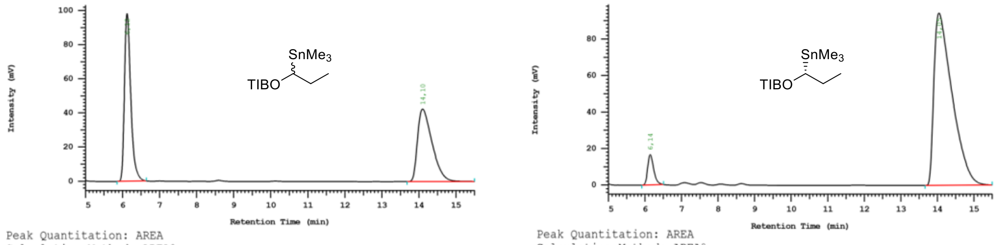

Peak Quantitation: AREA
Calculation Method: AREA%

No.	RT	Area	Conc 1	BC
1	6,12	1167208	50,315	BB
2	14,10	1152599	49,685	BB
		2319807	100,000	

Peak Quantitation: AREA
Calculation Method: AREA%

No.	RT	Area	Conc 1	BC
1	6,14	189324	5,525	BB
2	14,05	3237116	94,475	BB
		3426440	100,000	

Analytical data are in accordance with the literature.[7]

Stannane 32

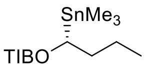

32
$C_{23}H_{40}O_2Sn$
467.28

According to GPC, TIB ester **28** (2.00 g, 6.57 mmol) gave stannane **32** (3.00 g, 6.42 mmol, ≥ 95%, *er* 96:4) as a colorless oil.

^{1}H-NMR (400 MHz, $CDCl_3$): δ = 6.99 (s, 2H), 5.01 (m_c, 1H), 2.94-2.78 (m, 3H), 2.00-1.76 (m, 2H), 1.51-1.35 (m, 2H), 1.24 (m_c, 18H), 0.93 (t, J = 7.4 Hz, 3H), 0.19 (s, d, J = 53.8 Hz and J = 51.6 Hz, 9H) ppm;

^{13}C-NMR (101 MHz, $CDCl_3$): δ = 171.5, 150.0, 145.0, 131.0, 120.9, 72.2, 36.1, 34.5, 31.6, 24.5, 24.3, 24.1, 21.1, 14.0, −9.0 ppm;

HRMS (ESI):$C_{23}H_{40}O_2SnNa$ $[M+Na]^+$ calculated: 491.1948, found: 491.1953;

R_f = 0.4 (PE:MTBE 98:2, UV, $KMnO_4$);

$[\alpha]_D^{20}$ = –33.3 (*c* 0.9, $CHCl_3$);

Chiral HPLC: (Daicel Chiracel® OD-H column (25 cm), hexanes, 0.7 mL/min, rt, 210 nm): t_R = 4.1 min (*S*), 12.7 min (*R*), *er* 4:96.

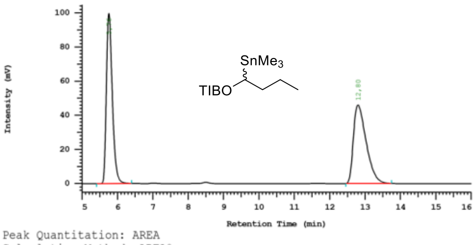

Peak Quantitation: AREA
Calculation Method: AREA%

No.	RT	Area	Conc 1	BC
1	5,76	1157481	50,060	BB
2	12,80	1154711	49,940	BB
		2312192	100,000	

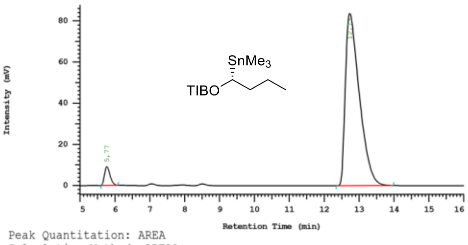

Peak Quantitation: AREA
Calculation Method: AREA%

No.	RT	Area	Conc 1	BC
1	5,77	100054	4,094	BB
2	12,73	2343843	95,906	BB
		2443897	100,000	

Stannane 33

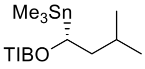

33
$C_{24}H_{42}O_2Sn$
481.31

According to GPC, TIB ester **29** (2.03 g, 6.37 mmol) gave stannane **33** (3.02 g, 6.27 mmol, ≥ 95%, *er* 95:5) as a colorless oil.

^{1}H-NMR (400 MHz, $CDCl_3$): δ = 6.99 (s, 2H), 5.07 (m_c, 1H), 2.93-2.77 (m, 3H), 2.05-1.95 (m, 1H), 1.80-1.69 (m, 1H), 1.49-1.37 (m, 1H), 1.26-1.21 (m, 18H), 0.92 (m_c, 6H), 0.19 (s, d, *J* = 53.9 Hz and *J* = 51.4 Hz, 9H) ppm;

^{13}C-NMR (101 MHz, $CDCl_3$): δ = 171.5, 150.0, 145.0, 131.0, 121.0, 70.1, 42.8, 34.5, 31.6, 25.7, 24.5, 24.4, 24.1, 23.4, 21.3, −9.1 ppm;

HRMS (ESI):$C_{24}H_{42}O_2SnNa$ $[M+Na]^+$ calculated: 505.2104, found: 505.2099;

R_f = 0.4 (PE:MTBE 98:2, UV, $KMnO_4$);

$[\alpha]_D^{20}$ = –43.8 (*c* 0.8, $CHCl_3$);

Chiral HPLC: (Daicel Chiracel® OD-H column (25 cm), hexanes, 0.7 mL/min, rt, 210 nm): t_R = 5.5 min (*S*), 7.3 min (*R*), *er* 5:95.

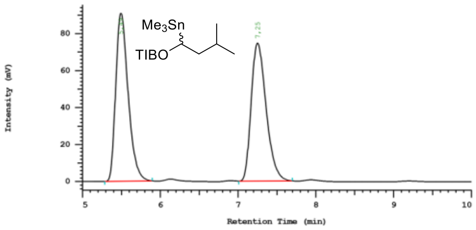

Peak Quantitation: AREA
Calculation Method: AREA%

No.	RT	Area	Conc 1	BC
1	5,49	1006165	50,182	BB
2	7,25	998884	49,818	BB
		2005049	100,000	

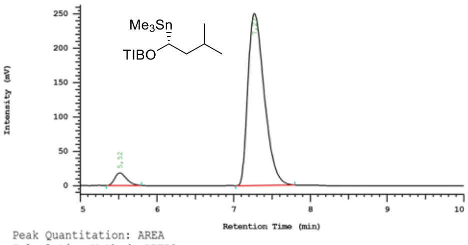

Peak Quantitation: AREA
Calculation Method: AREA%

No.	RT	Area	Conc 1	BC
1	5,52	195146	4,982	BB
2	7,27	3721959	95,018	BB
		3917105	100,000	

Stannane 34

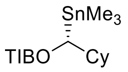

34

$C_{26}H_{44}O_2Sn$

507.35

According to GPC, TIB ester **21** (1.30 g, 3.77 mmol) gave stannane **34** (1.91 g, 3.76 mmol, ≥ 95%, *er* 95:5) as a colorless oil.

^{1}H-NMR (400 MHz, $CDCl_3$): δ = 6.99 (s, 2H), 4.95 (m_c, 1H), 2.93-2.79 (m, 3H), 1.88-1.62 (m, 6H), 1.29-1.04 (m, 23H), 0.21 (s, d, *J* = 53.4 Hz and *J* = 51.2 Hz, 9H) ppm;

^{13}C-NMR (101 MHz, $CDCl_3$): δ = 171.6, 149.9, 145.0, 131.0, 121.0, 78.8, 42.7, 34.5, 32.1, 31.6, 30.5, 26.54, 26.50, 26.4, 24.6, 24.4, 24.1, −7.8 ppm;

HRMS (ESI):$C_{26}H_{44}O_2SnNa$ $[M+Na]^+$ calculated: 531.2261, found: 531.2263;

R_f = 0.4 (PE:MTBE 98:2, UV, $KMnO_4$);

$[\alpha]_D^{20}$ = –29.0 (*c* 0.8, $CHCl_3$);

Chirale HPLC: (Daicel Chiracel® OD-H column (25 cm), hexanes, 0.5 mL/min, rt, 210 nm): t_R = 8.1 min (*S*), 17.1 min (*R*), *er* 5:95.

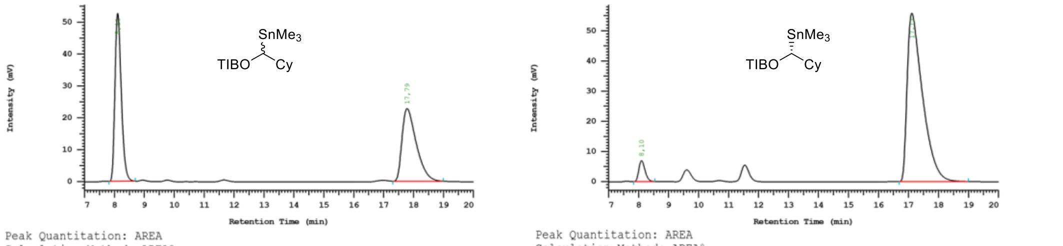

Peak Quantitation: AREA
Calculation Method: AREA%

No.	RT	Area	Conc 1	BC
1	8,11	775492	50,635	BB
2	17,79	756057	49,365	BB
		1531549	100,000	

Peak Quantitation: AREA
Calculation Method: AREA%

No.	RT	Area	Conc 1	BC
1	8,10	99597	4,765	BB
2	17,11	1990699	95,235	BB
		2090296	100,000	

Stannane 35

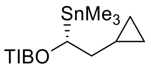

35

$C_{24}H_{40}O_2Sn$

479.29

According to GPC, TIB ester **22** (1.30 g, 4.11 mmol) gave stannane **35** (1.89 g, 3.94 mmol, ≥ 95%, *er* 95:5) as a colorless oil.

^{1}H-NMR (400 MHz, $CDCl_3$): δ = 6.99 (s, 2H), 5.11 (m_c, 1H), 2.93-2.81 (m, 3H), 1.98 (m_c, 1H), 1.73-1.59 (m, 1H), 1.26-1.21 (m, 18H), 0.86-0.76 (m, 1H), 0.51-0.41 (m, 2H), 0.27-0.07 (m, 11H) ppm;

^{13}C-NMR (101 MHz, $CDCl_3$): δ = 171.5, 150.0, 145.0, 131.0, 120.9, 72.4, 38.9, 34.5, 31.5, 24.5, 24.4, 24.1, 9.5, 5.2, 4.7, −8.9 ppm;

HRMS (ESI):$C_{24}H_{40}O_2SnNa$ $[M+Na]^+$ calculated: 503.1948, found: 503.1951;

R_f = 0.4 (PE:MTBE 98:2, UV, $KMnO_4$);

$[\alpha]_D^{20}$ = –34.7 (*c* 1.2, $CHCl_3$);

Chiral HPLC: (Daicel Chiracel® OD-H column (25 cm), hexanes, 0.7 mL/min, rt, 210 nm): t_R = 6.0 min (*S*), 10.2 min (*R*), *er* 5:95.

Stannane 36

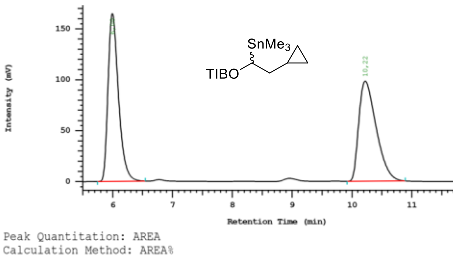

Peak Quantitation: AREA
Calculation Method: AREA%

No.	RT	Area	Conc 1	BC
1	6,00	1990638	50,161	BB
2	10,22	1977870	49,839	BB
		[illegible]	[illegible]	

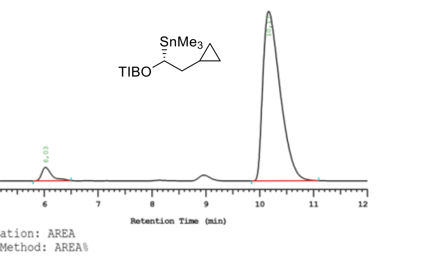

Peak Quantitation: AREA
Calculation Method: AREA%

No.	RT	Area	Conc 1	BC
1	6,03	163038	4,418	BB
2	10,17	3527005	95,582	BB
		[illegible]	[illegible]	

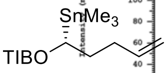

36

$C_{24}H_{40}O_2Sn$
479.29

According to GPC, TIB ester **23** (1.75 g, 5.53 mmol) gave stannane **36** (2.52 g, 5.26 mmol, ≥ 95%, *er* 6:94) as a colorless oil.

^{1}H-NMR (400 MHz, $CDCl_3$): δ = 7.00 (s, 2H), 5.81 (m_c, 1H), 5.06-4.95 (m, 3H), 2.94-2.78 (m, 3H), 2.26-1.88 (m, 4H), 1.24 (m_c, 18H), 0.20 (s, d, *J* = 54.0 Hz and *J* = 51.7 Hz, 9H) ppm;

^{13}C-NMR (101 MHz, $CDCl_3$): δ = 171.5, 150.1, 145.0, 137.9, 130.9, 121.0, 115.3, 71.7, 34.5, 33.3, 32.1, 31.6, 24.5, 24.4, 24.1, −8.9 ppm;

HRMS (ESI):$C_{24}H_{40}O_2SnNa$ $[M+Na]^+$ calculated: 503.1948, found: 503.1955;

R*f* = 0.4 (PE:MTBE 98:2, UV, $KMnO_4$);

$[\alpha]_D^{20}$ = –37.8 (*c* 0.9, $CHCl_3$);

Chiral HPLC: (Daicel Chiracel® OD-H column (25 cm), hexanes, 0.7 mL/min, rt, 210 nm): t_R = 5.9 min (*S*), 13.9 min (*R*), *er* 6:94.

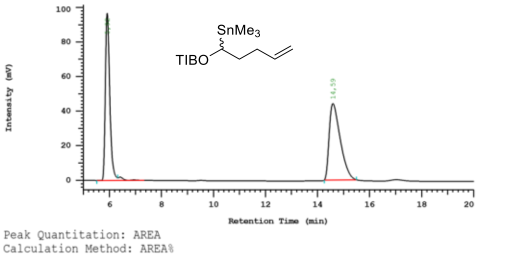

Peak Quantitation: AREA
Calculation Method: AREA%

No.	RT	Area	Conc 1	BC
1	5,92	1129019	46,495	BV
2	14,59	1299264	53,505	BB
		2428283	100,000	

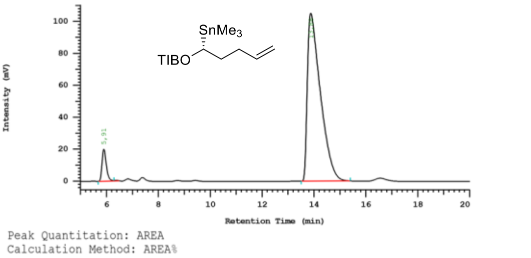

Peak Quantitation: AREA
Calculation Method: AREA%

No.	RT	Area	Conc 1	BC
1	5,91	226507	5,804	BV
2	13,88	3676018	94,196	BB
		3902525	100,000	

Analytical data are in accordance with the literature.[2]

Stannane 37

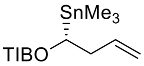

37

$C_{23}H_{38}O_2Sn$
465.27

According to GPC, TIB ester **30** (1.50 g, 4.96 mmol) gave stannane **37** (2.30 g, 4.94 mmol, ≥ 95%, *er* 93:7) as a turbid oil.

^{1}H-NMR (400 MHz, $CDCl_3$): δ = 6.99 (s, 2H), 5.83 (m_c, 1H), 5.16-5.04 (m, 3H), 2.93-2.80 (m, 3H), 2.69 (m_c, 2H), 1.25-1.21 (m, 18H), 0.19 (s, d, *J* = 54.2 Hz and *J* = 52.1 Hz, 9H) ppm;

^{13}C-NMR (101 MHz, $CDCl_3$): δ = 171.5, 150.1, 145.0, 135.8, 130.8, 121.0, 117.3, 71.1, 38.3, 34.5, 31.5, 24.6, 24.3, 24.1, −8.8 ppm;

HRMS (ESI):$C_{23}H_{38}O_2SnNa$ $[M+Na]^+$ calculated: 489.1791, found: 489.1791;

R*f* = 0.4 (PE:MTBE 98:2, UV, $KMnO_4$);

$[\alpha]_D^{20}$ = –19.6 (*c* 1.5, $CHCl_3$);

Chiral HPLC: (Daicel Chiracel® OD-H column (25 cm), hexanes, 0.7 mL/min, rt, 210 nm): t_R = 6.5 min (*S*), 14.9 min (*R*), *er* 7:93.

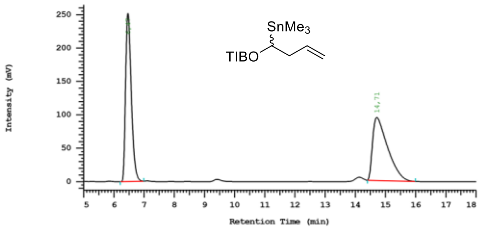

Peak Quantitation: AREA
Calculation Method: AREA%

No.	RT	Area	Conc 1	BC
1	6,47	3297557	50,278	BB
2	14,71	3261065	49,722	BB
		6558622	100,000	

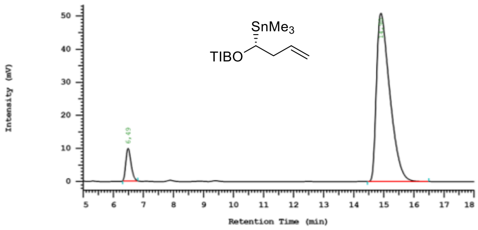

Peak Quantitation: AREA
Calculation Method: AREA%

No.	RT	Area	Conc 1	BC
1	6,49	117730	7,005	BB
2	14,90	1562882	92,995	BB
		1680612	100,000	

Stannane 38

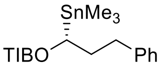

38

$C_{28}H_{42}O_2Sn$

529.35

Following a modified version of GPC using TIB ester **24** (1.62 g, 4.42 mmol, 1.0 eq.), (+)-sparteine (1.3 mL, 1.35 g, 5.75 mmol, 1.3 eq.), *s*BuLi (1.3 M in hexanes, 4.4 mL, 5.75 mmol, 1.3 eq.) and trimethyltin chloride (1.0 M in Et_2O, 5.8 mL, 5.75 mmol, 1.3 eq.) gave stannane **38** (2.32 g, 4.38 mmol, ≥ 95%, *er* 95:5) as a colorless oil.

^{1}H-NMR (400 MHz, $CDCl_3$): *δ* = 7.31-7.26 (m, 2H), 7.22-7.15 (m, 3H), 7.02 (s, 2H), 5.00 (m_c, 1H), 2.95-2.76 (m, 4H), 2.72-2.63 (m, 1H), 2.30-2.09 (m, 2H), 1.262 (d, *J* = 6.8 Hz) and 1.255 (d, *J* = 6.9 Hz, 18H), 0.22 (s, d, *J* = 54.0 Hz and *J* = 51.5 Hz, 9H) ppm;

^{13}C-NMR (101 MHz, $CDCl_3$): *δ* = 171.5, 150.1, 145.1, 141.8, 130.8, 128.6, 128.5, 126.1, 121.0, 72.0, 36.1, 34.6, 34.5, 31.7, 24.6, 24.4, 24.1, −8.8 ppm;

HRMS (ESI):$C_{28}H_{42}O_2SnNa$ $[M+Na]^+$ calculated: 553.2104, found: 553.2103;

R_f = 0.4 (PE:MTBE 98:2, UV, $KMnO_4$);

$[\alpha]_D^{20}$ = –41.1 (*c* 0.9, $CHCl_3$);

Chiral HPLC: (Daicel Chiracel® OD-H column (25 cm), hexanes, 0.7 mL/min, rt, 210 nm): t_R = 6.7 min (*S*), 15.2 min (*R*), *er* 5:95.

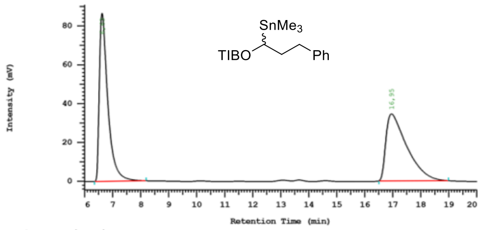

Peak Quantitation: AREA
Calculation Method: AREA%

No.	RT	Area	Conc 1	BC
1	6,63	1820490	50,331	BB
2	16,95	1796577	49,669	BB
		3617067	100,000	

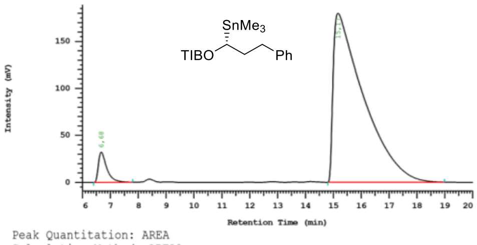

Peak Quantitation: AREA
Calculation Method: AREA%

No.	RT	Area	Conc 1	BC
1	6,68	673341	4,697	BB
2	15,17	13663620	95,303	BB
		14336961	100,000	

Analytical data are in accordance with the literature.[2]

Stannane 39

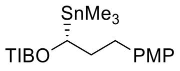

39

$C_{29}H_{44}O_3Sn$

559.38

Following a modified version of GPC using TIB ester **25** (2.00 g, 5.04 mmol, 1.0 eq.), (+)-sparteine (1.5 mL, 1.54 g, 6.56 mmol, 1.3 eq.), *s*BuLi (1.3 M in hexanes, 5.0 mL, 6.56 mmol, 1.3 eq.) and trimethyltin chloride (1.0 M in Et_2O, 7.5 mL, 7.56 mmol, 1.5 eq.) gave stannane **39** (1.95 g, 3.49 mmol, 69%, *er* 94:6) after flash column chromatography (PE:MTBE 98:2) as a colorless oil.

^{1}H-NMR (400 MHz, $CDCl_3$): δ = 7.12-7.06 (m, 2H), 7.01 (s, 2H), 6.86-6.81 (m, 2H), 4.99 (m_c, 1H), 3.79 (s, 3H), 2.94-2.82 (m, 3H), 2.79-2.69 (m, 1H), 2.67-2.57 (m, 1H), 2.27-2.05 (m, 2H), 1.258 (d, *J* = 6.7 Hz) and 1.253 (d, *J* = 7.0 Hz, 18H), 0.21 (s, d, *J* = 53.9 Hz and *J* = 51.7 Hz, 9H) ppm;

^{13}C-NMR (101 MHz, $CDCl_3$): δ = 171.5, 158.0, 150.1, 145.0, 133.8, 130.8, 129.4, 121.0, 114.0, 72.0, 55.4, 36.3, 34.5, 33.6, 31.7, 24.6, 24.4, 24.1, −8.8 ppm;

HRMS (ESI):$C_{29}H_{44}O_3SnNa$ $[M+Na]^+$ calculated: 583.2210, found: 583.2209;

R*f* = 0.2 (PE:MTBE 98:2, UV, $KMnO_4$);

$[\alpha]_D^{20}$ = –37.7 (*c* 1.2, $CHCl_3$);

Chiral HPLC: (Daicel Chiracel® OD-H column (25 cm), hexanes, 1.0 mL/min, rt, 210 nm): t_R = 18.1 min (*S*), 28.6 min (*R*), *er* 6:94.

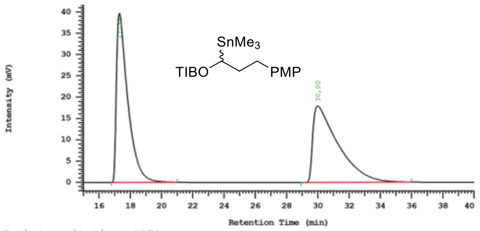

Peak Quantitation: AREA
Calculation Method: AREA%

No.	RT	Area	Conc 1	BC
1	17,31	2000140	49,679	BB
2	30,00	2026014	50,321	BB
		4026154	100,000	

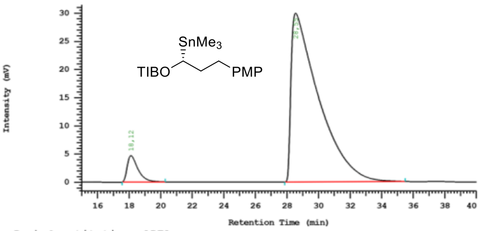

Peak Quantitation: AREA
Calculation Method: AREA%

No.	RT	Area	Conc 1	BC
1	18,12	222083	5,734	BB
2	28,55	3650847	94,266	BB
		3872930	100,000	

Stannane 40

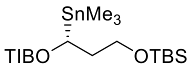

40

$C_{28}H_{52}O_3SiSn$

583.52

According to GPC, TIB ester **26** (2.00 g, 4.75 mmol) gave stannane **40** (2.75 g, 4.71 mmol, ≥ 95%, *er* 95:5) as a colorless oil.

^{1}H-NMR (600 MHz, $CDCl_3$): δ = 6.99 (s, 2H), 5.07 (m_c, 1H), 3.74-3.66 (m, 2H), 2.88 (sep, *J* = 6.9 Hz, 1H), 2.82 (sep, *J* = 6.8 Hz, 2H), 2.19-1.98 (m, 2H), 1.242 (d, *J* = 6.9 Hz) and 1.234 (d, *J* = 6.8 Hz, 18H), 0.89 (s, 9H), 0.20 (s, d, *J* = 54.1 Hz and *J* = 51.7 Hz, 9H) 0.044 (s, 3H), 0.040 (s, 3H) ppm;

^{13}C-NMR (151 MHz, $CDCl_3$): δ = 171.4, 150.1, 145.0, 130.9, 121.0, 68.7, 61.1, 37.0, 34.5, 31.6, 26.1, 24.5, 24.4, 24.1, 18.5, −5.09, −5.11, −8.9 ppm;

HRMS (ESI):$C_{28}H_{52}O_3SiSnNa$ $[M+Na]^+$ calculated: 607.2605, found: 607.2605;

R$_f$ = 0.4 (PE:MTBE 98:2, UV, $KMnO_4$);

$[\alpha]_D^{20}$ = –35.7 (*c* 1.4, $CHCl_3$);

Chiral HPLC: (Daicel Chiracel® OD-H column (25 cm), hexanes, 0.7 mL/min, rt, 210 nm): t_R = 5.6 min (*S*), 12.9 min (*R*), *er* 5:95.

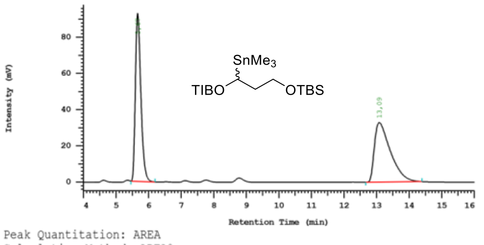

Peak Quantitation: AREA
Calculation Method: AREA%

No.	RT	Area	Conc 1	BC
1	5,67	1106833	49,676	BB
2	13,09	1121278	50,324	BB
		2228111	100,000	

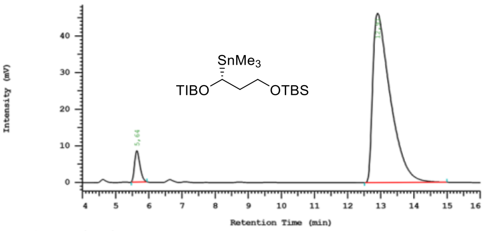

Peak Quantitation: AREA
Calculation Method: AREA%

No.	RT	Area	Conc 1	BC
1	5,64	97315	5,409	BB
2	12,91	1701728	94,591	BB
		1799043	100,000	

Analytical data are in accordance with the literature.[2]

3.4 General procedure D: C_2-symmetric 1,3-bis(boronic ester)

Following Aggarwal's procedure[2], a stirred solution of stannane (2.05 eq.) in Et_2O (0.2 M) at −78 °C was treated with *n*BuLi (1.6 M in hexanes, 2.0 eq., 0.5 mL/min). The reaction mixture was stirred for 1.5 h at this temperature. Then a solution of $pinBCH_2Bpin$[8] (1.0 eq., 0.5 mL/min) in Et_2O (0.5 M) was added. After 2.5 h at this temperature, the reaction mixture was warmed to rt and stirred overnight. H_2O and Et_2O were added and the organic layer was separated. The aqueous phase was extracted with Et_2O (3x), the organic layers were combined and dried over Na_2SO_4. The solvent was removed *in vacuo* and the crude material purified by flash column chromatography to afford C_2-symmetric 1,3-bis(boronic ester).

1,3-bis(boronic ester) 5a

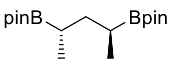

5a
$C_{17}H_{34}B_2O_4$
324.08

Following GPD, using stannane **12** (5.00 g, 11.4 mmol, 2.05 eq.) gave 1,3-bis(boronic ester) **5a** (1.47 g, 4.54 mmol, 82%, *dr* ≥ 95:5) after purification by flash column chromatography (PE:EtOAc 98:2) as a colorless oil.

^{1}H-NMR (400 MHz, $CDCl_3$): δ = 1.42 (t, *J* = 7.8 Hz, 2H), 1.23 (s, 24H), 1.12-1.03 (m, 2H), 0.93 (d, *J* = 7.4 Hz, 6H) ppm;

^{13}C-NMR (101 MHz, $CDCl_3$): δ = 82.9, 36.1, 24.90, 24.89, 15.5 ppm (carbon attached to boron not observed);

HRMS (ESI): $C_{17}H_{34}B_2O_4Na$ $[M+Na]^+$ calculated: 347.2541, found: 347.2542;

R$_f$ = 0.3 (PE:EtOAc 9:1, CAN);

$[\alpha]_D^{20}$ = +10.5 (*c* 1.0, $CHCl_3$);

Analytical data are in accordance with the literature.[2]

1,3-bis(boronic ester) 5b

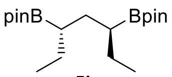

5b
$C_{19}H_{38}B_2O_4$
352.13

Following GPD, using stannane **31** (1.50 g, 3.31 mmol, 2.05 eq.) gave 1,3-bis(boronic ester) **5b** (0.49 g, 1.39 mmol, 86%, *dr* ≥ 95:5) after purification by flash column chromatography (PE:EtOAc 97:3) as a colorless oil.

^{1}H-NMR (400 MHz, $CDCl_3$): δ = 1.46-1.35 (m, 6H), 1.23 (s, 24H), 0.96-0.85 (m, 8H) ppm;

^{13}C-NMR (101 MHz, $CDCl_3$): δ = 82.9, 32.6, 25.0, 24.9, 13.8 ppm (carbon attached to boron not observed);

HRMS (ESI): $C_{19}H_{38}B_2O_4Na$ $[M+Na]^+$ calculated: 375.2854, found: 375.2852;

R$_f$ = 0.3 (PE:MTBE 95:5, CAN);

$[\alpha]_D^{20}$ = +12.5 (*c* 0.8, $CHCl_3$).

1,3-bis(boronic ester) 5c

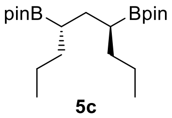

5c
$C_{21}H_{42}B_2O_4$
380.18

Following GPD, using stannane **32** (2.00 g, 4.28 mmol, 2.05 eq.) gave 1,3-bis(boronic ester) **5c** (0.45 g, 1.18 mmol, 56%, *dr* ≥ 95:5) after purification by flash column chromatography (PE:EtOAc 98:2) as a colorless oil.

^{1}H-NMR (400 MHz, $CDCl_3$): δ = 1.44-1.37 (m, 2H), 1.37-1.25 (m, 8H), 1.23 (s, 24H), 1.03-0.93 (m, 2H), 0.90-0.83 (m, 6H) ppm;

^{13}C-NMR (101 MHz, $CDCl_3$): δ = 82.8, 34.5, 33.2, 24.99, 24.95, 22.5, 14.6 ppm (carbon attached to boron not observed);

HRMS (ESI): $C_{21}H_{42}B_2O_4Na$ $[M+Na]^+$ calculated: 403.3167, found: 403.3164;

R$_f$ = 0.4 (PE:MTBE 95:5, CAN);

$[\alpha]_D^{20}$ = +11.8 (*c* 1.1, $CHCl_3$).

1,3-bis(boronic ester) 5d

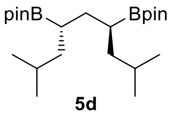

5d
$C_{23}H_{46}B_2O_4$
408.24

Following GPD, using stannane **33** (2.00 g, 4.16 mmol, 2.05 eq.) gave 1,3-bis(boronic ester) **5d** (0.52 g, 1.27 mmol, 63%, *dr* ≥ 95:5) after purification by flash column chromatography (PE:EtOAc 98:2) as a colorless solid.

^{1}H-NMR (400 MHz, $CDCl_3$): δ = 1.63-1.52 (m, 2H), 1.37 (t, *J* = 7.8 Hz, 2H), 1.32-1.25 (m, 2H), 1.23 (s, 24H), 1.20-1.11 (m, 2H), 1.10-1.01 (m, 2H), 0.85 (d, *J* = 6.6 Hz, 12H) ppm;

^{13}C-NMR (101 MHz, $CDCl_3$): δ = 82.8, 41.5, 33.7, 27.3, 25.00, 24.96, 23.2, 22.8 ppm (carbon attached to boron not observed);

HRMS (ESI): $C_{23}H_{46}B_2O_4Na$ $[M+Na]^+$ calculated: 431.3480, found: 431.3477;

R$_f$ = 0.5 (PE:MTBE 95:5, CAN);

$[\alpha]_D^{20}$ = +12.4 (*c* 0.7, $CHCl_3$);

Melting point: 52 °C.

1,3-bis(boronic ester) 5e

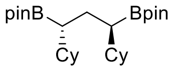

5e
$C_{27}H_{50}B_2O_4$
460.31

Following GPD, using stannane **34** (1.50 g, 2.96 mmol, 2.05 eq.) gave 1,3-bis(boronic ester) **5e** (0.11 g, 0.25 mmol, 17%, *dr* ≥ 95:5) after purification by flash column chromatography (PE:MTBE 98:2) as a colorless solid.

^{1}H-NMR (400 MHz, $CDCl_3$): δ = 1.78-1.57 (m, 10H), 1.49 (m_c, 2H), 1.40-1.29 (m, 2H), 1.24 (s, 12H), 1.23 (s, 12H), 1.21-0.93 (m, 10H), 0.77 (m_c, 2H) ppm;

^{13}C-NMR (101 MHz, $CDCl_3$): δ = 82.9, 40.9, 33.1, 32.6, 28.5, 27.04, 26.98, 26.9, 25.3, 25.0 ppm (carbon attached to boron not observed);

HRMS (ESI): $C_{27}H_{50}B_2O_4Na$ $[M+Na]^+$ calculated: 483.3793, found: 483.3788;

R_f = 0.3 (PE:MTBE 95:5, CAN);

$[\alpha]_D^{20}$ = +11.3 (*c* 0.5, $CHCl_3$);

Melting point: 78 °C.

1,3-bis(boronic ester) 5f

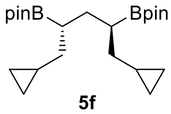

5f
$C_{23}H_{42}B_2O_4$
404.21

Following GPD, using stannane **35** (1.50 g, 3.13 mmol, 2.05 eq.) gave 1,3-bis(boronic ester) **5f** (0.41 g, 1.01 mmol, 66%, *dr* ≥ 95:5) after purification by flash column chromatography (PE:MTBE 98:2) as a colorless oil.

^{1}H-NMR (400 MHz, $CDCl_3$): δ = 1.49 (t, J = 7.8 Hz, 2H), 1.33-1.08 (m, 30H), 0.74-0.64 (m, 2H), 0.42-0.33 (m, 4H), 0.08-0.06 (m, 4H) ppm;

^{13}C-NMR (101 MHz, $CDCl_3$): δ = 82.9, 37.3, 33.4, 25.1, 25.0, 10.8, 5.3, 5.0 ppm (carbon attached to boron not observed);

HRMS (ESI): $C_{23}H_{42}B_2O_4Na$ $[M+Na]^+$ calculated: 427.3167, found: 427.3167;

R_f = 0.3 (PE:MTBE 95:5, CAN);

$[\alpha]_D^{20}$ = +7.4 (*c* 0.9, $CHCl_3$).

1,3-bis(boronic ester) 5g

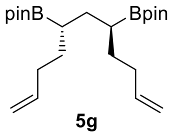

5g
$C_{23}H_{42}B_2O_4$
404.21

Following GPD, using stannane **36** (1.00 g, 2.09 mmol, 2.05 eq.) gave 1,3-bis(boronic ester) **5g** (0.32 g, 0.79 mmol, 77%, *dr* ≥ 95:5) after purification by flash column chromatography (PE:EtOAc 98:2) as a colorless oil.

^{1}H-NMR (400 MHz, $CDCl_3$): δ = 5.81 (m_c, 2H), 4.98 (m_c, 2H), 4.90 (m_c, 2H), 2.12-1.96 (m, 4H), 1.53-1.36 (m, 6H), 1.23 (s, 24H), 1.08-0.97 (m, 2H) ppm;

^{13}C-NMR (101 MHz, $CDCl_3$): δ = 139.5, 114.2, 82.9, 33.6, 33.0, 31.5, 24.99, 24.98 ppm (carbon attached to boron not observed);

HRMS (ESI): $C_{23}H_{42}B_2O_4Na$ $[M+Na]^+$ calculated: 427.3167, found: 427.3169;

R_f = 0.4 (PE:MTBE 95:5, CAN);

$[\alpha]_D^{20}$ = +7.9 (*c* 0.9, $CHCl_3$);

Analytical data are in accordance with the literature.[2]

1,3-bis(boronic ester) 5h

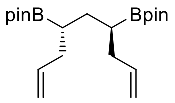

5h
$C_{21}H_{38}B_2O_4$
376.15

Following GPD, using stannane **37** (1.50 g, 3.22 mmol, 2.05 eq.) gave 1,3-bis(boronic ester) **5h** (0.30 g, 0.80 mmol, 51%, *dr* ≥ 95:5) after purification by flash column chromatography (PE:MTBE 98:2) as a colorless oil.

^{1}H-NMR (400 MHz, $CDCl_3$): δ = 5.85-5.74 (m, 2H), 5.00 (m_c, 2H), 4.91 (m_c, 2H), 2.12 (m_c, 4H), 1.49-1.37 (m, 2H), 1.22 (s, 24H), 1.16-1.06 (m, 2H) ppm;

^{13}C-NMR (101 MHz, $CDCl_3$): δ = 138.7, 114.9, 83.1, 36.3, 32.5, 25.0 ppm (carbon attached to boron not observed);

HRMS (ESI): $C_{21}H_{38}B_2O_4Na$ $[M+Na]^+$ calculated: 399.2854, found: 399.2852;

R_f = 0.3 (PE:MTBE 95:5, CAN);

$[\alpha]_D^{20}$ = +18.2 (*c* 1.1, $CHCl_3$).

1,3-bis(boronic ester) 5i

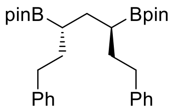

5i
$C_{31}H_{46}B_2O_4$
504.33

Following GPD, using stannane **38** (2.00 g, 3.78 mmol, 2.05 eq.) gave 1,3-bis(boronic ester) **5i** (0.36 g, 0.71 mmol, 39%, *dr* 9:1) after purification by flash column chromatography (PE:EtOAc 98:2) as a colorless oil.

^{1}H-NMR (400 MHz, $CDCl_3$): δ = 7.29-7.11 (m, 10H), 2.66-2.53 (m, 4H), 1.77-1.60 (m, 4H), 1.59-1.51 (m, 2H), 1.23 (s, 24H), 1.14-1.03 (m, 2H) ppm;

^{13}C-NMR (101 MHz, $CDCl_3$): δ = 143.3, 128.6, 128.3, 125.6, 83.0, 35.7, 34.3, 33.2, 25.00, 24.99 ppm (carbon attached to boron not observed);

HRMS (ESI): $C_{31}H_{46}B_2O_4Na$ $[M+Na]^+$ calculated: 527.3480, found: 527.3489;

R$_f$ = 0.3 (PE:MTBE 95:5, UV, CAN);

$[\alpha]_D^{20}$ = −7.6 (*c* 1.1, $CHCl_3$).

1,3-bis(boronic ester) 5j

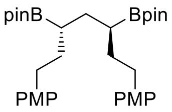

5j
$C_{33}H_{50}B_2O_6$
564.38

Following a modified version of GPD using stannane **39** (1.75 g, 3.13 mmol, 1.1 eq.), *n*BuLi (1.6 M in hexanes, 1.8 mL, 2.84 mmol, 1.0 eq.) and $pinBCH_2Bpin$ (1.14 g, 4.27 mmol, 1.5 eq.) gave 1,3-bis(boronic ester) **5j** (0.38 g, 0.67 mmol, 47%, *dr* 9:1) after purification by flash column chromatography (PE:MTBE 99:1 → 93:7) as a colorless oil.

^{1}H-NMR (600 MHz, $CDCl_3$): δ = 7.11-7.05 (m, 4H), 6.82-6.77 (m, 4H), 3.77 (s, 6H), 2.59-2.47 (m, 4H), 1.69-1.57 (m, 4H), 1.52 (t, *J* = 8.1 Hz, 2H), 1.25-1.21 (m, 24H), 1.05 (m_c, 2H) ppm;

^{13}C-NMR (151 MHz, $CDCl_3$): δ = 157.7, 135.4, 129.4, 113.8, 83.0, 55.4, 34.8, 34.5, 33.2, 25.01, 24.99 ppm (carbon attached to boron not observed);

HRMS (ESI): $C_{33}H_{50}B_2O_6Na$ $[M+Na]^+$ calculated: 587.3691, found: 587.3708;

R$_f$ = 0.3 (PE:MTBE 9:1, UV, CAN);

$[\alpha]_D^{20}$ = −8.5 (*c* 0.9, $CHCl_3$).

Analytical data are in accordance with the literature.[2]

1,3-bis(boronic ester) 5k

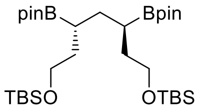

5k
$C_{31}H_{66}B_2O_6Si_2$
612.65

Following GPD, using stannane **40** (1.50 g, 2.57 mmol, 2.05 eq.) gave 1,3-bis(boronic ester) **5k** (0.37 g, 0.60 mmol, 48%, *dr* ≥ 95:5) after purification by flash column chromatography (PE:EtOAc 98:2) as a colorless oil.

^{1}H-NMR (400 MHz, $CDCl_3$): δ = 3.58 (m_c, 4H), 1.63-1.56 (m, 4H), 1.45 (t, *J* = 7.9 Hz, 2H), 1.22 (s, 24H), 1.06-0.97 (m, 2H), 0.88 (s, 18H), 0.03 (s, 12H) ppm;

^{13}C-NMR (101 MHz, $CDCl_3$): δ = 83.0, 63.3, 35.2, 33.2, 26.2, 25.0, 18.5, −5.0, −5.1 ppm (carbon attached to boron not observed);

HRMS (ESI): $C_{31}H_{66}B_2O_6Si_2Na$ $[M+Na]^+$ calculated: 635.4482, found: 635.4485;

R$_f$ = 0.3 (PE:MTBE 95:5, CAN);

$[\alpha]_{365}^{20}$ = +7.5 (*c* 0.9, $CHCl_3$)[9].

Analytical data are in accordance with the literature.[2]

3.5 General procedure E: mono-Zweifel olefination

To a stirred solution of 1,3-bis(boronic ester, 1.0 eq.) in THF (0.2 M) at −78 °C, was added VinylMgBr (1.0 M in THF, 1.7 eq., 0.5 mL/min) and stirring was continued for 30 min at this temperature. Then the solution was warmed to rt and stirred for further 30 min. After cooling to −78 °C iodine (4.0 eq.) was added in two portions over a period of 5 min. MeOH (0.5 M, 0.15 mL/min) was added to the dark solution and the reaction mixture was stirred for 30 min. Then a suspension of NaOMe (8.0 eq., 0.5 mL/min) in MeOH (1 M) was added and the red reaction mixture was stirred for further 30 min at −78 °C. After warming to rt the black reaction mixture was stirred overnight. MTBE and sat. aq. $Na_2S_2O_3$ were added until the dark color disappeared. The phases were separated and the aqueous phase was extracted with MTBE (3x). The combined organic phases were washed with sat. aq. NaCl and dried over Na_2SO_4, concentrated *in vacuo* and the crude material was purified by flash column chromatography to afford mono-Zweifel product.

Mono-Zweifel product 6a

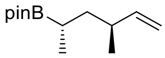

6a
$C_{13}H_{25}BO_2$
224.15

According to GPE 1,3-bis(boronic ester) **5a** (100 mg, 0.31 mmol, 1.0 eq.) gave mono-Zweifel product **6a** (33 mg, 0.15 mmol, 48%, 94% brsm[a, b]) after purification by flash column chromatography (PE:MTBE 98:2 → 9:1) as a yellow and reisolated 1,3-bis(boronic ester) **5a** (48 mg, 0.15 mmol) as a slightly yellow oil.

[a] At a 5.99 mmol scale (1.94 g) of 1,3-bis(boronic ester) **5a** mono-Zweifel product **6a** was obtained in 35% (470 mg, 2.10 mmol, 81% brsm).
[b] After three cycles mono-Zweifel product **6a** was obtained in 62% (831 mg, 3.71 mmol).

^{1}H-NMR (400 MHz, $CDCl_3$): δ = 5.68 (m_c, 1H), 4.98-4.87 (m, 2H), 2.23-2.12 (m, 1H), 1.49-1.41 (m, 1H), 1.30-1.25 (m, 1H), 1.24 (s, 12H), 1.12-1.03 (m, 1H), 0.98-0.92 (m, 6H) ppm;

^{13}C-NMR (101 MHz, $CDCl_3$): δ = 145.2, 112.6, 82.9, 40.4, 37.2, 24.90, 24.85, 20.3, 15.8 ppm (carbon attached to boron not observed);

HRMS (EI): $C_{13}H_{25}BO_2$ $[M]^{\cdot+}$ calculated: 224.1948, found: 224.1947;

R_f = 0.4 (PE:MTBE 97:3, $KMnO_4$);

$[\alpha]_D^{20}$ = +14.5 (*c* 1.2, $CHCl_3$).

Mono-Zweifel product 6b

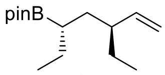

6b
$C_{15}H_{29}BO_2$
252.21

According to GPE 1,3-bis(boronic ester) **5b** (100 mg, 0.28 mmol, 1.0 eq.) gave mono-Zweifel product **6b** (27 mg, 0.11 mmol, 39%, 85% brsm) after purification by flash column chromatography (PE:MTBE 98:2 → 9:1) as a yellow and reisolated 1,3-bis(boronic ester) **5b** (52 mg, 0.15 mmol) as a colorless oil.

^{1}H-NMR (400 MHz, $CDCl_3$): δ = 5.55-5.42 (m, 1H), 5.00-4.89 (m, 2H), 1.93-1.79 (m, 1H), 1.57-1.48 (m, 1H), 1.47-1.29 (m, 3H), 1.29-1.15 (m, 14H), 1.04-0.94 (m, 1H), 0.92-0.78 (m, 6H) ppm;

^{13}C-NMR (101 MHz, $CDCl_3$): δ = 143.4, 114.7, 82.9, 45.9, 36.7, 28.4, 25.1, 25.02, 24.96, 13.8, 11.9 ppm (carbon attached to boron not observed);

HRMS (EI): $C_{15}H_{29}BO_2$ $[M]^{\bullet+}$ calculated: 252.2261, found: 252.2270;

R*f* = 0.3 (PE:MTBE 98:2, $KMnO_4$);

$[\alpha]_D^{20}$ = +7.3 (*c* 0.6, $CHCl_3$).

Mono-Zweifel product 6c

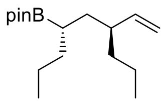

6c
$C_{17}H_{33}BO_2$
280.26

According to GPE 1,3-bis(boronic ester) **5c** (180 mg, 0.47 mmol, 1.0 eq.) gave mono-Zweifel product **6c** (40 mg, 0.14 mmol, 30%, 67% brsm) after purification by flash column chromatography (PE:MTBE 98:2 → 9:1) as a yellow and reisolated 1,3-bis(boronic ester) **5c** (100 mg, 0.26 mmol) as a colorless oil.

^{1}H-NMR (400 MHz, $CDCl_3$): δ = 5.47 (m_c, 1H), 4.98-4.89 (m, 2H), 1.95 (m_c, 1H), 1.50 (m_c, 1H), 1.41-1.15 (m, 21H), 1.11-1.01 (m, 1H), 0.91-0.82 (m, 6H) ppm;

^{13}C-NMR (101 MHz, $CDCl_3$): δ = 143.7, 114.5, 82.9, 43.8, 37.9, 37.2, 34.5, 25.02, 24.96, 22.4, 20.4, 14.6, 14.2 ppm (carbon attached to boron not observed);

HRMS (EI): $C_{17}H_{33}BO_2$ $[M]^{\bullet+}$ calculated: 280.2574, found: 280.2573;

R*f* = 0.5 (PE:MTBE 98:2, $KMnO_4$);

$[\alpha]_D^{20}$ = +8.3 (*c* 0.6, $CHCl_3$).

Mono-Zweifel product 6d

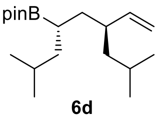

6d
$C_{19}H_{37}BO_2$
308.31

According to GPE 1,3-bis(boronic ester) **5d** (200 mg, 0.49 mmol, 1.0 eq.) gave mono-Zweifel product **6d** (45 mg, 0.15 mmol, 31%, 68% brsm) after purification by flash column chromatography (PE:MTBE 105:1 → 9:1) as a yellow oil and reisolated 1,3-bis(boronic ester) **5d** (111 mg, 0.27 mmol) as a colorless solid.

^{1}H-NMR (400 MHz, $CDCl_3$): δ = 5.52-5.35 (m, 1H), 5.00-4.88 (m, 2H), 2.10-1.95 (m, 1H), 1.66-1.49 (m, 2H), 1.47-1.27 (m, 2H), 1.26-1.21 (m, 12H), 1.21-1.06 (m, 5H), 0.89-0.79 (m, 12H) ppm;

^{13}C-NMR (101 MHz, $CDCl_3$): δ = 143.6, 114.5, 82.9, 45.3, 42.0, 41.4, 37.6, 27.3, 25.4, 25.03, 24.99, 23.6, 23.1, 22.9, 22.0 ppm (carbon attached to boron not observed);

HRMS (ESI): $C_{19}H_{38}BO_2$ $[M+H]^+$ calculated: 309.2965, found: 309.2965;

R*f* = 0.5 (PE:MTBE 98:2, $KMnO_4$);

$[\alpha]_D^{20}$ = +5.7 (*c* 0.7, $CHCl_3$).

Mono-Zweifel product 6e

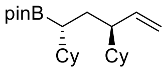

6e

$C_{23}H_{41}BO_2$

360.39

According to GPE 1,3-bis(boronic ester) **5e** (39 mg, 84.7 µmol, 1.0 eq.) gave mono-Zweifel product **6e** (8 mg, 22.2 µmol, 26%, 93% brsm) after purification by flash column chromatography (PE:MTBE 100:1 → 9:1) as a yellow oil and reisolated 1,3-bis(boronic ester) **5e** (28 mg, 60.8 µmol) as a colorless solid.

^{1}H-NMR (400 MHz, $CDCl_3$): δ = 5.47 (m_c, 1H), 4.99 (m_c, 1H), 4.88 (m_c, 1H), 1.78-1.56 (m, 12H), 1.38-0.79 (m, 26H) ppm;

^{13}C-NMR (101 MHz, $CDCl_3$): δ = 141.7, 115.5, 82.9, 50.4, 42.8, 40.7, 33.1, 32.8, 31.2, 31.1, 30.1, 27.00, 26.98, 26.9, 26.8, 25.3, 25.0 ppm (carbon attached to boron not observed);

HRMS (ESI): $C_{23}H_{41}BO_2Na$ [M+Na]$^+$ calculated: 383.3097, found: 383.3097;

R$_f$ = 0.6 (PE:MTBE 98:2, $KMnO_4$);

$[\alpha]_D^{20}$ = +12.6 (*c* 0.5, $CHCl_3$).

Mono-Zweifel product 6f

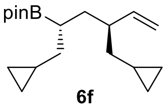

6f

$C_{19}H_{33}BO_2$

304.28

According to GPE 1,3-bis(boronic ester) **5f** (140 mg, 346 µmol, 1.0 eq.) gave mono-Zweifel product **6f** (22 mg, 72.3 µmol, 21%, 77% brsm, *dr* 6:1) after purification by flash column chromatography (PE:MTBE 100:1 → 9:1) as a yellow and reisolated 1,3-bis(boronic ester) **5f** (102 mg, 252 µmol) as a colorless oil.

^{1}H-NMR (400 MHz, $CDCl_3$): δ = 5.72-5.52 (m, 1H), 5.03-4.89 (m, 2H), 2.19-2.04 (m, 1H), 1.64-1.53 (m, 1H), 1.52-1.34 (m, 1H), 1.34-1.16 (m, 16H), 1.14-1.04 (m, 1H), 0.76-0.60 (m, 2H), 0.44-0.32 (m, 4H), 0.07-−0.07 (m, 4H) ppm;

^{13}C-NMR (101 MHz, $CDCl_3$): δ = 143.6, 114.4, 82.9, 44.7, 41.2, 37.4, 37.0, 25.2, 25.0, 10.8, 9.1, 5.2, 5.0, 4.9, 4.7 ppm (carbon attached to boron not observed);

HRMS (ESI): $C_{19}H_{33}BO_2Na$ [M+Na]$^+$ calculated: 327.2471, found: 327.2470;

R$_f$ = 0.4 (PE:MTBE 98:2, $KMnO_4$);

$[\alpha]_D^{20}$ = +3.3 (*c* 0.7, $CHCl_3$).

Mono-Zweifel product 6g

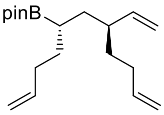

6g
$C_{19}H_{33}BO_2$
304.28

According to GPE 1,3-bis(boronic ester) **5g** (100 mg, 0.25 mmol, 1.0 eq.) gave mono-Zweifel product **6g** (30 mg, 0.10 mmol, 40%, 77% brsm) after purification by flash column chromatography (PE:MTBE 98:2 → 9:1) as a yellow and reisolated 1,3-bis(boronic ester) **5g** (48 mg, 0.12 mmol) as a colorless oil.

^{1}H-NMR (400 MHz, $CDCl_3$): δ = 5.87-5.73 (m, 2H), 5.46 (m_c, 1H), 5.02-4.88 (m, 6H), 2.12-1.92 (m, 5H), 1.53-1.26 (m, 6H), 1.24 (s, 12H), 1.12-1.02 (m, 1H) ppm;

^{13}C-NMR (101 MHz, $CDCl_3$): δ = 143.1, 139.4, 139.2, 115.2, 114.35, 114.32, 83.0, 43.5, 37.1, 34.8, 33.5, 31.5, 31.4, 25.01, 24.99 ppm (carbon attached to boron not observed);

HRMS (ESI): $C_{19}H_{33}BO_2Na$ $[M+Na]^+$ calculated: 327.2471, found: 327.2468;

R_f = 0.4 (PE:MTBE 98:2, $KMnO_4$);

$[\alpha]_D^{20}$ = +6.9 (*c* 0.7, $CHCl_3$).

Mono-Zweifel product 6h

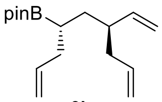

6h
$C_{17}H_{29}BO_2$
276.23

According to GPE 1,3-bis(boronic ester) **5h** (160 mg, 0.43 mmol, 1.0 eq.) gave mono-Zweifel product **6h** (37 mg, 0.13 mmol, 30%, 72% brsm, *dr* 9:1) after purification by flash column chromatography (PE:MTBE 100:1 → 9:1) as a yellow and reisolated 1,3-bis(boronic ester) **5h** (93 mg, 0.25 mmol) as a colorless oil.

^{1}H-NMR (400 MHz, $CDCl_3$): δ = 5.85-5.69 (m, 2H), 5.63-5.46 (m, 1H), 5.05-4.89 (m, 6H), 2.22-2.00 (m, 5H), 1.60-1.52 (m, 1H), 1.47-1.13 (m, 14H) ppm;

^{13}C-NMR (101 MHz, $CDCl_3$): δ = 142.6, 138.6, 137.3, 115.6, 115.04, 114.97, 83.1, 43.7, 40.2, 36.3, 36.0, 25.04, 24.97 ppm (carbon attached to boron not observed);

HRMS (ESI): $C_{17}H_{29}BO_2Na$ $[M+Na]^+$ calculated: 299.2158, found: 299.2160;

R_f = 0.4 (PE:MTBE 98:2, $KMnO_4$);

$[\alpha]_D^{20}$ = +14.3 (*c* 0.3, $CHCl_3$).

Mono-Zweifel product 6i

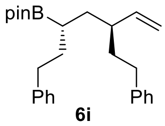

6i

$C_{27}H_{37}BO_2$

404.40

According to GPE 1,3-bis(boronic ester) **5i** (190 mg, 0.38 mmol, 1.0 eq.) gave mono-Zweifel product **6i** (56 mg, 0.14 mmol, 37%, 88% brsm, *dr* 3:1) after purification by flash column chromatography (PE:MTBE 98:2 → 9:1) as a yellow and reisolated 1,3-bis(boronic ester) **5i** (110 mg, 0.22 mmol) as a colorless oil.

^{1}H-NMR (400 MHz, $CDCl_3$): δ = 7.29-7.11 (m, 10H), 5.62-5.46 (m, 1H), 5.09-4.89 (m, 2H), 2.70-2.39 (m, 4H), 2.10-1.95 (m, 1H), 1.76-1.54 (m, 4H), 1.53-1.28 (m, 2H), 1.28-1.17 (m, 12H), 1.17-1.10 (m, 1H) ppm;

^{13}C-NMR (101 MHz, $CDCl_3$): δ = 143.2, 142.94, 142.89, 128.6, 128.5, 128.359, 128.355, 125.69, 125.66, 115.6, 83.0, 43.5, 37.3, 37.2, 35.8, 34.2, 33.5, 25.0, 24.9 ppm (carbon attached to boron not observed);

HRMS (ESI): $C_{27}H_{37}BO_2Na$ $[M+Na]^+$ calculated: 427.2784, found: 427.2784;

R_f = 0.3 (PE:MTBE 98:2, UV, $KMnO_4$);

$[\alpha]_{365}^{20}$ = −10.1 (*c* 0.7, $CHCl_3$).

Mono-Zweifel product 6j

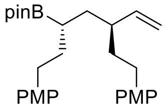

6j

$C_{29}H_{41}BO_4$

464.45

According to GPE 1,3-bis(boronic ester) **5j** (175 mg, 310 µmol, 1.0 eq.) gave mono-Zweifel product **6j** (17 mg, 36.6 µmol, 12%, 34% brsm, *dr* 3:1) after purification by flash column chromatography (PE:MTBE 95:5 → 9:1) as a yellow and reisolated 1,3-bis(boronic ester) **5j** (114 mg, 202 µmol) as a colorless oil.

^{1}H-NMR (600 MHz, $CDCl_3$): δ = 7.11-7.04 (m, 4H), 6.84-6.78 (m, 4H), 5.60-5.49 (m, 1H), 5.06-4.94 (m, 2H), 3.81-3.75 (m, 6H), 2.63-2.42 (m, 4H), 2.09-1.94 (m, 1H), 1.70-1.41 (m, 5H), 1.41-1.19 (m, 13H), 1.17-1.06 (m, 1H) ppm;

^{13}C-NMR (151 MHz, $CDCl_3$): δ = 157.74, 157.73, 143.0, 135.3, 135.0, 129.5, 129.3, 115.5, 113.8, 83.0, 55.4, 43.3, 37.5, 37.2, 34.8, 34.4, 32.6, 25.0, 24.9 ppm (carbon attached to boron not observed);

HRMS (ESI): $C_{29}H_{41}BO_4Na$ $[M+Na]^+$ calculated: 487.2996, found: 487.3002;

R_f = 0.5 (PE:MTBE 9:1, UV, $KMnO_4$);

$[\alpha]_D^{20}$ = −5.8 (*c* 1.2, $CHCl_3$).

Mono-Zweifel product 6k

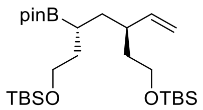

6k
$C_{27}H_{57}BO_4Si_2$
512.73

According to GPE 1,3-bis(boronic ester) **5k** (150 mg, 245 µmol, 1.0 eq.) gave mono-Zweifel product **6k** (33 mg, 64.4 µmol, 26%, 53% brsm) after purification by flash column chromatography (PE:MTBE 98:2 → 9:1) as a colorless and reisolated 1,3-bis(boronic ester) **5k** (76 mg, 124 µmol) as a colorless oil.

^{1}H-NMR (400 MHz, $CDCl_3$): δ = 5.52-5.39 (m, 1H), 5.00-4.90 (m, 2H), 3.64-3.50 (m, 4H), 2.13 (m_c, 1H), 1.68-1.47 (m, 4H), 1.46-1.32 (m, 1H), 1.30-1.20 (m, 13H), 1.13-1.03 (m, 1H), 0.88 (s, 18H), 0.03 (s, 6H), 0.02 (s, 6H) ppm;

^{13}C-NMR (101 MHz, $CDCl_3$): δ = 142.7, 115.3, 83.0, 63.1, 61.4, 40.5, 38.6, 37.2, 35.0, 26.2, 26.1, 25.00, 24.98, 18.53, 18.45, −5.05, −5.06, −5.12, −5.13 ppm (carbon attached to boron not observed);

HRMS (ESI): $C_{27}H_{57}BO_4Si_2Na$ $[M+Na]^+$ calculated: 535.3786, found: 535.3785;

R_f = 0.2 (PE:MTBE 98:2, $KMnO_4$);

$[\boldsymbol{\alpha}]_{365}^{20}$ = −5.1 (*c* 0.8, $CHCl_3$).

Mono-Zweifel product 7a

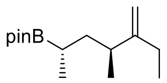

7a
$C_{15}H_{29}BO_2$
252.21

Magnesium turnings were activated using aq. 1.0 M HCl, washed with H_2O (2x) and acetone (2x). The shiny turnings were then flame dried under high vacuum and purged with argon. After addition of THF (1.0 M) 2-bromobutene was added slowly and the reaction mixture was heated to 45 °C until most of the magnesium had reacted (30 min). The concentration of the Grignard reagent was determined by titration with menthol in THF using 1,10-phenanthroline as an indicator (double determination).[10]
To a stirred solution of 1,3-bis(boronic ester) **5a** (275 mg, 0.85 mmol, 1.0 eq.) in THF (4.3 mL) at −78 °C, was added the freshly prepared Grignard solution (0.8 M in THF, 1.8 mL, 1.44 mmol, 1.7 eq., 0.5 mL/min) and stirring was continued for 30 min at this temperature. Then the solution was warmed to rt and stirred for further 30 min. After cooling to −78 °C iodine (861 mg, 3.39 mmol, 4.0 eq.) was added in four portions over a period of 10 min. MeOH (7.0 mL, 0.15 mL/min) was added to the dark solution and the reaction mixture was stirred for 30 min. Then a suspension of NaOMe (367 mg, 6.79 mmol, 8.0 eq., 0.5 mL/min) in MeOH (7.0 mL) was added and the red reaction mixture was stirred for further 30 min at −78 °C. After warming to rt the black reaction mixture was stirred overnight. MTBE and sat. aq. $Na_2S_2O_3$ were added until the dark color disappeared. The phases were separated and the aqueous phase was extracted with MTBE (3x). The combined organic phases were washed with sat. aq. NaCl and dried over Na_2SO_4, concentrated *in vacuo* and the crude material was purified by flash column chromatography (PE:MTBE 98:2 → 9:1) to afford mono-Zweifel product **7a** (57 mg, 0.23 mmol, 27%, 72% brsm[c]) as a yellow oil and reisolated 1,3-bis(boronic ester) **5a** (172 mg, 0.53 mmol) as a slightly yellow oil.
[c] At a 5.96 mmol scale (1.93 g) of 1,3-bis(boronic ester) **5a** mono-Zweifel product **7a** was obtained in 21% (318 mg, 1.26 mmol, 50% brsm).

^{1}H-NMR (400 MHz, $CDCl_3$): δ = 4.73-4.67 (m, 2H), 2.20 (sex, *J* = 7.1 Hz, 1H), 2.06-1.91 (m, 2H), 1.48-1.30 (m, 2H), 1.23 (s, 12H), 1.05-0.98 (m, 7H), 0.96-0.92 (m, 3H) ppm;

^{13}C-NMR (101 MHz, $CDCl_3$): *δ* = 156.6, 106.6, 82.9, 39.6, 39.5, 25.9, 24.91, 24.85, 20.3, 15.9, 12.5 ppm (carbon attached to boron not observed);

HRMS (ESI): $C_{15}H_{29}BO_2Na$ $[M+Na]^+$ calculated: 275.2158, found: 275.2159;

R*f* = 0.4 (PE:MTBE 98:2, $KMnO_4$);

$[\alpha]_D^{20}$ = +4.2 (*c* 0.8, $CHCl_3$).

Mono-Zweifel product 7b

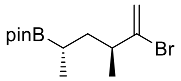

7b
$C_{13}H_{24}BBrO_2$
303.05

Di*iso*propylamine (0.10 mL, 70 mg, 0.69 mmol, 1.5 eq.) was dissolved in THF (0.30 mL). After cooling to −78 °C *n*BuLi (1.6 M in hexanes, 0.46 mL, 0.75 mmol, 1.6 eq., 0.5 mL/min) was added and the reaction mixture was stirred for 15 min at −78 °C and then for 5 min at rt. Simultaneously 1,3-bis(boronic ester) **5a** (150 mg, 0.46 mmol, 1.0 eq.) was dissolved in THF (2.3 mL) and cooled to −95 °C (MeOH/$N_{2(l)}$). Vinyl bromide (1.0 M in THF, 0.60 mL, 0.60 mmol, 1.3 eq.) was added and then the freshly prepared LDA solution was added (60 µL/min, rinsed once with 0.25 mL). After stirring for 1 h at this temperature iodine (470 mg, 1.85 mmol, 4.0 eq.) was added in three portions over a period of 10 min. The reaction mixture was then warmed to −78 °C and MeOH (3.8 mL, 0.15 mL/min) was added to the dark red solution and the reaction mixture was stirred for 30 min. Then a suspension of NaOMe (200 mg, 3.70 mmol, 8.0 eq., 0.5 mL/min) in MeOH (3.8 mL) was added and the red reaction mixture was stirred for further 30 min at −78 °C. After warming to rt the deep red reaction mixture was stirred overnight. MTBE and sat. aq. $Na_2S_2O_3$ were added until the deep red color disappeared. The phases were separated and the aqueous phase was extracted with MTBE (3x). The combined organic phases were washed with sat. aq. NaCl and dried over Na_2SO_4, concentrated *in vacuo* and the crude material was purified by flash column chromatography (PE:MTBE 98:2) to afford mono-Zweifel product **7b** (65 mg, 0.21 mmol, 46%) as a yellow oil.

^{1}H-NMR (400 MHz, $CDCl_3$): *δ* = 5.60-5.56 (m, 1H), 5.37-5.34 (m, 1H), 2.44 (sex, *J* = 6.8 Hz, 1H), 1.43 (t, *J* = 7.2 Hz, 2H), 1.23 (s, 12H), 1.08-0.95 (m, 7H) ppm;

^{13}C-NMR (101 MHz, $CDCl_3$): *δ* = 142.3, 115.7, 83.0, 43.5, 38.9, 24.9, 24.8, 20.5, 16.1 ppm (carbon attached to boron not observed);

HRMS (EI): $C_{12}H_{21}BBrO_2$ $[M-Me]^+$ calculated: 287.0818, found: 287.0813;

R*f* = 0.4 (PE:MTBE 98:2, $KMnO_4$);

$[\alpha]_D^{20}$ = +14.1 (*c* 0.9, $CHCl_3$).

Mono-Zweifel product 7c

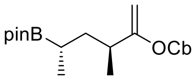

7c
$C_{20}H_{38}BNO_4$
367.34

Di*iso*propylamine (0.17 mL, 122 mg, 1.20 mmol, 1.3 eq.) was dissolved in THF (0.50 mL). After cooling to −78 °C *n*BuLi (1.6 M in hexanes, 0.81 mL, 1.30 mmol, 1.4 eq., 0.5 mL/min) was added and the reaction mixture was stirred for 15 min at −78 °C and then for 5 min at rt. The freshly prepared LDA solution was then added to a stirred solution of vinyl carbamate[11] (174 mg, 1.02 mmol, 1.1 eq.) in THF (2.5 mL) at −78 °C (0.2 mL/min, rinsed once with 0.50 mL). After stirring for 10 min at this temperature 1,3-bis(boronic ester) **5a** (300 mg, 0.93 mmol, 1.0 eq.) in THF (4.6 mL, 0.3 mL/min) was added. The reaction mixture was stirred for 30 min at −78 °C before being warmed to 0 °C and stirring was continued for 30 min at this temperature. After cooling the slightly yellow

reaction mixture to −78 °C iodine (940 mg, 3.70 mmol, 4.0 eq.) was added in four portions over a period of 15 min. MeOH (7.5 mL, 0.15 mL/min) was added to the dark solution and the reaction mixture was stirred for 30 min. Then a suspension of NaOMe (400 mg, 7.41 mmol, 8.0 eq., 0.5 mL/min) in MeOH (7.5 mL) was added and the red reaction mixture was stirred for further 30 min at −78 °C. After warming to rt the deep red reaction mixture was stirred overnight. MTBE and sat. aq. $Na_2S_2O_3$ were added until the deep red color disappeared. The phases were separated and the aqueous phase was extracted with MTBE (3x). The combined organic phases were washed with sat. aq. NaCl and dried over Na_2SO_4, concentrated *in vacuo* and the crude material was purified by flash column chromatography (PE:MTBE 95:5 → 9:1) to afford mono-Zweifel product **7c** (250 mg, 0.68 mmol, 73%) as a slightly yellow oil and the double olefination product (30 mg, 0.07mmol, 8%) as a yellow oil.
Mono olefination product **7c**:

^{1}H-NMR (400 MHz, $CDCl_3$): δ = 4.65 (s, 2H), 4.13-3.72 (brm, 2H), 2.40 (sex, *J* = 7.0 Hz, 1H), 1.52-1.37 (m, 2H) 1.25-1.21 (m, 24H), 1.14-1.04 (m, 4H), 0.94 (d, *J* = 7.3 Hz, 3H) ppm;

^{13}C-NMR (101 MHz, $CDCl_3$): δ = 160.6, 153.7, 98.8, 82.9, 46.5 (br), 46.0 (br), 38.0, 37.5, 24.9, 24.8, 21.6 (br), 20.6 (br), 18.3, 15.6 ppm (carbon attached to boron not observed);

HRMS (ESI): $C_{20}H_{38}BNO_4Na$ $[M+Na]^+$ calculated: 390.2792, found: 390.2794;

R$_f$ = 0.4 (PE:MTBE 5:1, vanillin);

$[\alpha]_D^{20}$ = +10.6 (*c* 1.9, $CHCl_3$).

Double olefination product (*dr* 9:1):

^{1}H-NMR (400 MHz, $CDCl_3$): δ = 4.69 (m_c, 4H), 4.09-3.76 (brm, 4H), 2.49 (sex, *J* = 7.0 Hz, 2H), 1.48 (t, *J* = 7.0 Hz, 2H), 1.24 (d, *J* = 6.9 Hz, 24H), 1.07 (d, *J* = 7.0 Hz, 6H) ppm;

^{13}C-NMR (101 MHz, $CDCl_3$): δ = 159.8, 153.7, 99.4, 46.6 (br), 46.0 (br), 39.1, 36.5, 21.6 (br), 20.6 (br), 18.7 ppm;

HRMS (ESI): $C_{23}H_{42}N_2O_4Na$ $[M+Na]^+$ calculated: 433.3042, found: 433.3046;

R$_f$ = 0.1 (PE:MTBE 9:1, vanillin);

$[\alpha]_{365}^{20}$ = +15.9 (*c* 0.9, $CHCl_3$).

3.6 Follow-Up Chemistry

Alkyne 8

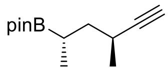

8
$C_{13}H_{23}BO_2$
222.14

Di*iso*propylamine (48 µL, 34 mg, 340 µmol, 2.5 eq.) was dissolved in THF (0.14 mL). After cooling to −78 °C *n*BuLi (1.6 M in hexanes, 0.22 mL, 354 µmol, 2.6 eq., 0.5 mL/min) was added and the reaction mixture stirred for 15 min at −78 °C and then for 5 min at rt. Simultaneously mono-Zweifel product **7c** (50 mg, 136 µmol, 1.0 eq.) was dissolved in Et_2O (1.4 mL) and cooled to −78 °C. Then the prepared LDA solution was added slowly (0.3 mL/min, rinsed once with 0.25 mL). After warming to 0 °C the slightly yellow and cloudy reaction mixture was stirred for 1.5 h at this temperature. Sat. aq. NH_4Cl and Et_2O were added and the organic layer was separated. The aqueous layer was extracted with Et_2O (3x) The combined organic layers were washed with sat. aq. NaCl and dried over Na_2SO_4. The solvent was removed *in vacuo* and the crude material was purified by flash column chromatography (PE:MTBE 15:1) to afford alkyne **8** (22 mg, 99.0 µmol, 73%) as a colorless oil.

^{1}H-NMR (400 MHz, $CDCl_3$): δ = 2.56-2.46 (m, 1H), 2.01 (d, J = 2.5 Hz, 1H), 1.61-1.53 (m, 1H), 1.47-1.37 (m, 1H), 1.33-1.25 (m, 1H), 1.23 (s, 12H), 1.16 (d, J = 6.7 Hz, 3H), 0.98 (d, J = 7.4 Hz, 3H) ppm;

^{13}C-NMR (101 MHz, $CDCl_3$): δ = 89.7, 83.1, 68.2, 40.5, 25.1, 24.9, 24.8, 21.2, 15.9 ppm (carbon attached to boron not observed);

HRMS (EI): $C_{12}H_{20}BO_2$ [M-Me]$^+$ calculated: 207.1556, found: 207.1555;

R*f* = 0.6 (PE:MTBE 15:1, vanillin);

$[\alpha]_D^{20}$ = +40.8 (*c* 0.8, $CHCl_3$).

Alkyne 8

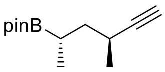

8
$C_{13}H_{23}BO_2$
222.14

To a stirred solution of mono-Zweifel product **7c** (60 mg, 163 µmol, 1.0 eq.) in Et_2O (1.6 mL) was added *t*BuLi (1.9 M in pentane, 0.21 mL, 408 µmol, 2.5 eq.) at −78 °C. After warming to 0 °C the reaction mixture was stirred for 2.5 h at this temperature. Sat. aq. NH_4Cl and Et_2O were added and the organic layer was separated. The aqueous layer was extracted with Et_2O (3x). The combined organic layers were washed with sat. aq. NaCl and dried over Na_2SO_4. The solvent was removed *in vacuo* and the crude material was purified by flash column chromatography (PE:MTBE 15:1) to afford alkyne **8** (15 mg, 67.5 µmol, 41%) as a colorless oil.

The analytical data were in accordance with those observed via the elimination using LDA.

3.7 Synthesis of (+)-invictolide

(*S*)-*N*-((1*R*,2*R*)-1-hydroxy-1-phenylpropan-2-yl)-*N*,2-dimethylpentanamide 41

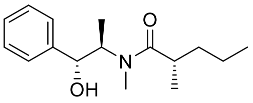

41
$C_{16}H_{25}NO_2$
263.38

Lithium chloride (17.7 g, 416 mmol, 13.5 eq.) was flame dried under high vacuum and purged with argon. Then THF (130 mL) and di*iso*propylamine (17.5 mL, 12.5 g, 123 mmol, 4.0 eq.) were added. After cooling to −78 °C *n*BuLi (1.6 M in hexanes, 73 mL, 117 mmol, 3.8 eq., 0.5 mL/min) was added and the reaction mixture stirred for 10 min at −78 °C and then for 5 min at 0 °C. Then a solution of Myers auxiliary **14**[12] (13.6 g, 61.6 mmol, 2.0 eq.) in THF (170 mL, 2.0 mL/min) was added at −78 °C. The reaction mixture was stirred for 1 h at this temperature before being warmed to 0 °C and stirred for further 30 min. Finally the reaction mixture was warmed to rt and stirred for another 5 min. After cooling to 0 °C a solution of 1-propyliodide (**16**, 3.0 mL, 5.23 g, 30.8 mmol, 1.0 eq.) in THF (31 mL, 1.0 mL/min) was added. The reaction mixture was slowly warmed to rt and stirred overnight at this temperature. Sat. aq. NH_4Cl and EtOAc were added at 0 °C and the organic layer was separated. The aqueous layer was extracted with EtOAc (3x). The combined organic layers were washed with sat. aq. NaCl and dried over Na_2SO_4. The solvent was removed *in vacuo* and the crude material was purified by flash column chromatography (PE:EtOAc 1:1) to afford (*S*)-*N*-((1*R*,2*R*)-1-hydroxy-1-phenylpropan-2-yl)-*N*,2-dimethylpentanamide **41** as a colorless oil (mixture of amide bond rotamers, 5:1, NMR), which was directly used in the next step without further characterization.

HRMS (ESI):$C_{16}H_{25}NO_2Na$ $[M+Na]^+$ calculated: 286.1783, found: 286.1782;

$\mathbf{R_f}$ = 0.36 (PE:EtOAc 1:1, UV, $KMnO_4$).

(*S*)-2-methylpentan-1-ol (15)

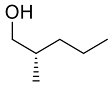

15
$C_6H_{14}O$
102.18

To a stirred solution of di*iso*propylamine (18.5 mL, 13.4 g, 132 mmol, 4.3 eq.) in THF (120 mL) was added *n*BuLi (1.6 M in hexanes, 49 mL, 124 mmol, 4.0 eq.) at −78 °C. After stirring for 10 min at this temperature, the solution was warmed to 0 °C and stirred for further 10 min. Then borane-ammonia complex (4.29 g, 139 mmol, 4.5 eq.) was added in three portions over a period of 5 min and the reaction mixture was allowed to stir for 15 min at 0 °C before being warmed to rt and stirred for further 15 min. After cooling to 0 °C a solution of the obtained amide **41** (1.0 eq.) in THF (160 mL) was added. The reaction mixture was slowly warmed to rt and stirred for 5 h at that temperature before being cooled to 0 °C. Sat. aq. NH_4Cl and MTBE were added. The organic layer was separated and the aqueous one was extracted with MTBE (3x). The combined organic layers were washed with sat. aq. NH_4Cl (1x) and sat. aq. NaCl (1x), dried over Na_2SO_4 and the solvent was removed under slightly reduced pressure (≥ 150 mbar). Purification by vacuum distillation (8 mbar, bp.: 43 °C) afforded (*S*)-2-methylpentan-1-ol (**15**, 1.67 g, 16.4 mmol, 53% o2s) as a colorless liquid.

^{1}H-NMR (400 MHz, $CDCl_3$): δ = 3.51 (dd, J = 10.5 Hz, 5.8 Hz, 1H), 3.41 (dd, J = 10.5 Hz, 6.5 Hz, 1H), 1.63 (m_c, 1H), 1.43-1.23 (m, 4H), 1.16-1.06 (m, 1H), 0.94-0.87 (m, 6H) ppm;

^{13}C-NMR (101 MHz, $CDCl_3$): δ = 68.6, 35.6, 35.5, 20.2, 16.7, 14.5 ppm;

HRMS (EI): C_6H_{12} $[M-H_2O]^{\bullet+}$ calculated: 84.0939, found: 84.0940;

R_f = 0.4 (PE:MTBE 2:1, vanillin);

$[\alpha]_D^{20}$ = −15.0 (*c* 1.0, $CHCl_3$).

Chiral GC: (Hydrodex®-*β*-6TBDM (25 m x 0.25 mm x 0.25 µm), N_2, 1.1 mL/min, 0.1 °C/min, 45-60 °C) t_R = 43.5 min (*R*), 46.4 min (*S*), *er* 1:99.

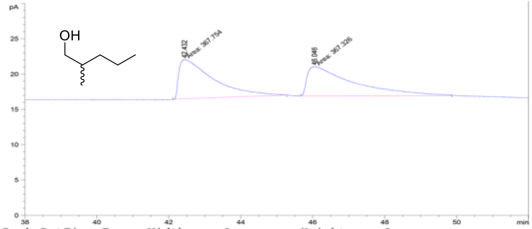

Peak #	RetTime [min]	Type	Width [min]	Area [pA*s]	Height [pA]	Area %
1	42.432	MM	1.1021	367.75449	5.56136	50.02914
2	46.046	MM	1.4752	367.32608	4.14997	49.97086
Totals :				735.08057	9.71133	

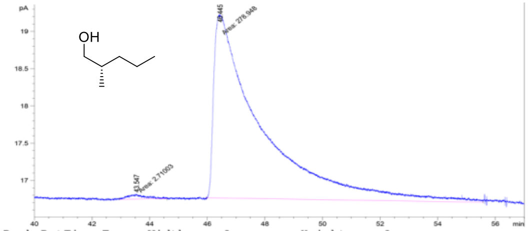

Peak #	RetTime [min]	Type	Width [min]	Area [pA*s]	Height [pA]	Area %
1	43.547	MM	0.6293	2.71003	7.17765e-2	0.96217
2	46.445	MM	1.8818	278.94843	2.47057	99.03783
Totals :				281.65846	2.54235	

Analytical data are in accordance with the literature.[13]

1-propylboronic acid pinacol ester (17)

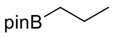

17
$C_9H_{19}BO_2$
170.06

A solution of 1-propyliodide (**16**, 1.00 mL, 1.73 g, 10.2 mmol, 1.0 eq.) and pinBO*i*Pr (2.5 mL, 2.28 g, 12.3 mmol, 1.2 eq.) in Et_2O (60 mL) was cooled to −105 °C. After the dropwise addition of *t*BuLi (1.9 M in pentane, 11.0 mL, 20.4 mmol, 2.0 eq.) the reaction mixture was stirred for 10 min at that temperature, before being warmed to rt. The reaction mixture was treated with sat. aq. NH_4Cl and the phases were separated. The organic layer was washed with sat. aq. NaCl (2x) and the combined aqueous layers were extracted with Et_2O (3x). The combined organic layers were dried over Na_2SO_4 and concentrated under reduced pressure. The crude product was purified by flash column chromatography (PE:MTBE 50:1) to give 1-propylboronic acid pinacol ester (**17**, 1.52 g, 8.94 mmol, 88%) as a colorless liquid.

^{1}H-NMR (400 MHz, $CDCl_3$): *δ* = 1.44 (sex, *J* = 7.4 Hz, 2H), 1.24 (s, 12H), 0.92 (t, *J* = 7.4 Hz, 3H), 0.77 (t, *J* = 7.4 Hz, 2H) ppm;

^{13}C-NMR (101 MHz, $CDCl_3$): *δ* = 83.0, 25.0, 17.6, 17.0 ppm (carbon attached to boron not observed);

HRMS (EI): $C_8H_{16}BO_2$ [M-Me]$^+$ calculated: 155.1243, found: 155.1243;

R_f = 0.3 (PE:MTBE 30:1, CAN).

Analytical data are in accordance with the literature.[14]

(*S*)-2-methylpentan-1-ol (15)

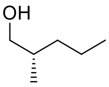

15
$C_6H_{14}O$
102.18

A stirred solution of stannane **12** (1.00 g, 2.28 mmol, 1.3 eq.) in Et_2O (11.5 mL) at −78 °C was treated with *n*BuLi (1.6 M in hexanes, 1.4 mL, 2.28 mmol, 1.3 eq., 0.5 mL/min). The reaction mixture was stirred for 1.5 h at this temperature. Then a solution of 1-

propylboronic acid pinacol ester (**17**, 298 mg, 1.75 mmol, 1.0 eq., 0.5 mL/min) in Et_2O (3.5 mL) was added. After 2.5 h at this temperature, the reaction mixture was warmed to rt and stirred overnight. H_2O and Et_2O were added and the organic layer was separated. The aqueous phase was extracted with Et_2O (3x), the organic layers were combined and dried over Na_2SO_4. The obtained boronic ester (R_f = 0.4, PE:MTBE 30:1, CAN) and bromochloromethane (0.34 mL, 0.68 g, 5.25 mmol, 3.0 eq.) were dissolved in Et_2O (8.8 mL) and cooled to −78 °C. Then *n*BuLi (1.6 M in hexanes, 2.7 mL, 4.38 mmol, 2.5 eq., 20 µL/min) was added and the reaction mixture was stirred for 20 min at that temperature. After warming to rt the cloudy reaction mixture was stirred overnight. The reaction mixture was then filtered through a short plug of silica using Et_2O as eluent. The solvent was removed *in vacuo*, the residue was dissolved in THF (9.0 mL) and cooled to −20 °C. A premixed solution of NaOH (2.0 M)/H_2O_2 (35%, 2/1 v/v, 7.0 mL) was added dropwise. The reaction mixture was stirred for 2 h at rt before being quenched by the addition of sat. aq. $Na_2S_2O_3$ at 0 °C. The solution was diluted with MTBE, the phases were separated and the aqueous phase was extracted with MTBE (3x). The combined organic layers were dried over Na_2SO_4 and concentrated *in vacuo*. The crude product was purified by flash column chromatography (PE:MTBE 6:1) to afford alcohol **15** (81 mg, 0.79 mmol, 45% o3s) as a colorless oil.

The analytical data were in accordance with those observed via the Myers route.

Chiral GC: (Hydrodex®-*β*-6TBDM (25 m x 0.25 mm x 0.25 µm), N_2, 1.1 mL/min, 0.1 °C/min, 45-60 °C) t_R = 43.5 min (*R*), 46.3 min (*S*), *er* 1:99.

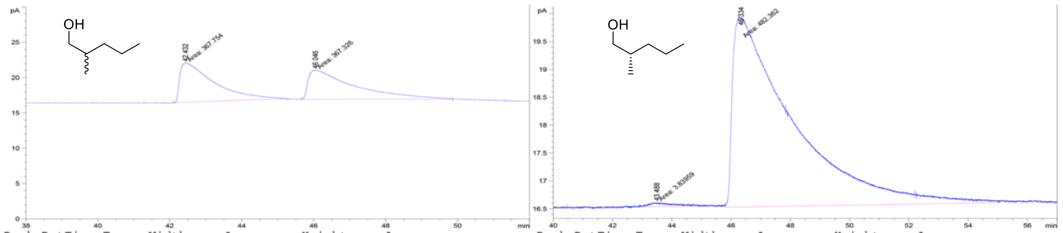

Peak #	RetTime [min]	Type	Width [min]	Area [pA*s]	Height [pA]	Area %
1	42.432	MM	1.1021	367.75449	5.56136	50.02914
2	46.046	MM	1.4752	367.32608	4.14997	49.97086
Totals :				735.08057	9.71133	

Peak #	RetTime [min]	Type	Width [min]	Area [pA*s]	Height [pA]	Area %
1	43.488	MM	0.8548	3.83959	7.48671e-2	0.78971
2	46.334	MM	2.3552	482.36154	3.41343	99.21029
Totals :				486.20113	3.48830	

Alcohol 9

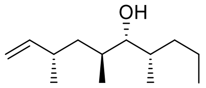

9
$C_{13}H_{26}O$
198.35

To a stirred solution of TIB ester **10** (405 mg, 1.22 mmol, 1.0 eq.) and (−)-sparteine (0.45 mL, 457 mg, 1.95 mmol, 1.6 eq.) in Et_2O (4.9 mL) at −78 °C was added *s*BuLi (1.3 M in hexanes, 1.4 mL, 1.83 mmol, 1.5 eq., 0.5 mL/min). The brown reaction mixture was stirred for 5 h at that temperature before a solution of mono-Zweifel product **6a** (383 mg, 1.71 mmol, 1.4 eq.) in THF (3.4 mL, 0.5 mL/min) was added. After stirring for further 2 h at −78 °C, the yellow reaction mixture was warmed to 40 °C and stirred overnight. The reaction mixture was cooled to rt and aq. 5% H_3PO_4 was added and the biphasic mixture was stirred for 20 min. The phases were separated, the organic layer was washed with aq. 5% H_3PO_4 (3x) and the combined aqueous phases were extracted with MTBE (3x). The combined organic phases were dried over Na_2SO_4 and concentrated *in vacuo*. The orange residue was dissolved in THF (6.0 mL) and cooled to −20 °C. A premixed solution of NaOH (2.0 M)/H_2O_2 (35%, 2/1 v/v, 5.0 mL) was added dropwise. The reaction mixture was stirred for 2 h at rt before being quenched by the addition of sat. aq. $Na_2S_2O_3$ at 0 °C. The solution was diluted with MTBE, the phases were separated and the aqueous phase was extracted with MTBE (3x). The combined organic layers were dried over Na_2SO_4 and concentrated *in vacuo*. The crude product was purified by flash column chromatography (PE:MTBE 98:2) to afford alcohol **9** (150 mg, 0.76 mmol, 62% o2s, *dr* ≥ 95:5) as a colorless oil.

^{1}H-NMR (400 MHz, $CDCl_3$): δ = 5.79 (m_c, 1H), 5.01-4.86 (m, 2H), 3.16 (m_c, 1H), 2.25 (m_c, 1H), 1.70-1.60 (m, 2H), 1.58-1.50 (m, 1H), 1.41-1.11 (m, 6H), 0.97 (d, J = 6.8 Hz, 3H), 0.90 (t, J = 7.0 Hz, 3H), 0.86 (d, J = 6.8 Hz, 6H) ppm;

^{13}C-NMR (101 MHz, $CDCl_3$): δ = 146.0, 111.9, 79.8, 39.4, 36.6, 35.3, 34.7, 33.8, 20.4, 19.3, 16.5, 14.5, 13.2 ppm;

HRMS (EI): C_8H_{13} [M-C_5H_{13}O]$^+$ calculated: 109.1017, found: 109.1017;

R$_f$ = 0.3 (PE:MTBE 95:5, vanillin);

$[\alpha]_D^{20}$ = –31.1 (c 1.2, $CHCl_3$).

Lactol 42

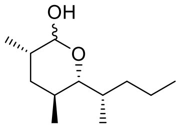

42
$C_{12}H_{24}O_2$
200.32

A solution of alcohol **9** (94 mg, 0.47 mmol, 1.0 eq.) in CH_2Cl_2 (4.8 mL) was treated with a dilute stream of ozone at −78 °C until the light blue color persisted. The solution was then purged with oxygen until the blue color disappeared and treated with PPh_3 (249 mg, 0.95 mmol, 2.0 eq.). The reaction mixture was allowed to slowly reach rt overnight (with cooling bath). Silica gel was added, the solvent was removed *in vacuo* and the crude product was purified by flash column chromatography (PE:MTBE 10:1 → 8:1) to afford lactol **42** (83 mg, 0.41 mmol, 87%, mixture of diastereoisomers, *dr* 1.7:1) as a colorless oil. Lactol **42** was used in the next step without detailed characterization.

^{1}H-NMR (400 MHz, $CDCl_3$): δ = 4.92 (s) and 4.74 (dd, J = 6.1 Hz, 2.4 Hz, 1H), 3.51 (dd, J = 10.1 Hz, 2.4 Hz) and 2.98 (dd, J = 10.1 Hz, 2.2 Hz, 1H), 2.68 (d, J = 5.9 Hz) and 2.27 (d, J = 2.9 Hz, 1H), 1.93-1.60 (m, 4H), 1.43-1.21 (m, 5H), 1.04 (d, J = 7.2 Hz) and 0.99 (d, J = 6.9 Hz, 3H), 0.92-0.84 (m, 6H), 0.78 (d, J = 6.5 Hz) and 0.74 (d, J = 6.6 Hz, 3H) ppm;

HRMS (ESI): $C_{12}H_{24}O_2Na$ [M+Na]$^+$ calculated: 223.1674, found: 223.1676;

R$_f$ = 0.3 (PE:MTBE 9:1, vanillin).

(+)-invictolide (4)

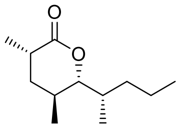

(+)-invictolide (**4**)
$C_{12}H_{22}O_2$
198.31

Predried Molecular Sieve (4 Å, 300 mg) was flame dried under high vacuum and purged with argon. Then CH_2Cl_2 (3.5 mL), lactol **42** (70 mg, 0.35 mmol, 1.0 eq.) and NMO (123 mg, 1.05 mmol, 3.0 eq.) were added successively. The reaction mixture was stirred for 30 min before TPAP (6 mg, 17.1 µmol, 0.05 eq.) was added. After stirring for further 2 h the solvent was removed *in vacuo*. The crude product was purified by flash column chromatography (PE:MTBE 8:1) providing (+)-invictolide (**4**, 55 mg, 0.28 mmol, 80%) as a colorless oil which solidified in the freezer (mp ≤ 28 °C).

^{1}H-NMR (400 MHz, C_6D_6): δ = 3.44 (d, J = 10.2 Hz, 1H), 2.04 (m_c, 1H), 1.50 (m_c, 1H), 1.45-1.29 (m, 3H), 1.28-1.09 (m, 3H), 1.07 (d, J = 6.9 Hz, 3H), 1.02-0.94 (m, 1H), 0.87 (t, J = 7.2 Hz, 3H), 0.81 (d, J = 6.3 Hz, 3H), 0.45 (d, J = 6.8 Hz, 3H) ppm;

^{1}H-NMR (400 MHz, $CDCl_3$): δ = 3.90 (dd, J = 10.2 Hz, 2.1 Hz, 1H), 2.64 (m_c, 1H), 1.98 (m_c, 1H), 1.75-1.64 (m, 3H), 1.48-1.25 (m, 4H), 1.21 (d, J = 6.9 Hz, 3H), 0.97 (d, J = 6.8 Hz, 3H), 0.92-0.87 (m, 6H) ppm;

^{13}C-NMR (101 MHz, C_6D_6): δ = 174.7, 84.5, 36.6, 35.4, 34.0, 32.3, 28.6, 20.8, 17.3, 16.7, 14.4, 12.4 ppm;

^{13}C-NMR (101 MHz, $CDCl_3$): δ = 176.9, 85.8, 36.2, 35.5, 33.7, 32.7, 28.5, 20.6, 17.8, 16.7, 14.3, 12.4 ppm;

HRMS (ESI): $C_{12}H_{22}O_2Na$ $[M+Na]^+$ calculated: 221.1517, found: 221.1519;

R_f = 0.2 (PE:MTBE 9:1, vanillin, anisaldehyde);

$[\boldsymbol{\alpha}]_{\mathbf{D}}^{\mathbf{20}}$ = +98.4 (c 0.6, $CHCl_3$).

Analytical data are in accordance with the literature.[15]

3.8 Synthesis of serricornin

Alcohol 19

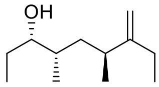

19
$C_{12}H_{24}O$
184.32

To a stirred solution of TIB ester **18** (250 mg, 0.86 mmol, 1.0 eq.) and (+)-sparteine (0.32 mL, 323 mg, 1.38 mmol, 1.6 eq.) in Et_2O (3.4 mL) at −78 °C was added *s*BuLi (1.3 M in hexanes, 0.99 mL, 1.29 mmol, 1.5 eq., 0.5 mL/min). The brown reaction mixture was stirred for 5 h at that temperature before a solution of mono-Zweifel product **7a** (304 mg, 1.21 mmol, 1.4 eq.) in THF (2.4 mL, 0.5 mL/min) was added. After stirring for further 2 h at −78 °C, the yellow reaction mixture was warmed to 40 °C and stirred overnight. The reaction mixture was cooled to rt and aq. 5% H_3PO_4 was added and the biphasic mixture was stirred for 20 min. The phases were separated, the organic layer was washed with aq. 5% H_3PO_4 (3x) and the combined aqueous phases were extracted with MTBE (3x). The combined organic phases were dried over Na_2SO_4 and concentrated *in vacuo*. The slightly orange residue was dissolved in THF (4.4 mL) and cooled to −20 °C. A premixed solution of NaOH (2.0 M)/H_2O_2 (35%, 2/1 v/v, 3.4 mL) was added dropwise. The reaction mixture was stirred for 2.5 h at rt before being quenched by the addition of sat. aq. $Na_2S_2O_3$ at 0 °C. The solution was diluted with MTBE, the phases were separated and the aqueous phase was extracted with MTBE (3x). The combined organic layers were dried over Na_2SO_4 and concentrated *in vacuo*. The crude product was purified by flash column chromatography (PE:MTBE 98:2 → 95:5) to afford alcohol **19** (110 mg, 0.60 mmol, 70% o2s, *dr* 10:1) as a colorless oil.

^{1}H-NMR (600 MHz, $CDCl_3$): δ = 4.75-4.69 (m, 2H), 3.43 (m_c, 1H), 2.24 (sex, *J* = 7.0 Hz, 1H), 2.06-1.96 (m, 2H), 1.63-1.28 (m, 5H), 1.20 (br, 1H), 1.04 (t, *J* = 7.4 Hz, 3H), 1.00 (d, *J* = 6.9 Hz, 3H), 0.94 (t, *J* = 7.4 Hz, 3H), 0.85 (d, *J* = 6.8 Hz, 3H) ppm;

^{13}C-NMR (151 MHz, $CDCl_3$): δ = 156.8, 106.6, 76.6, 39.7, 37.6, 35.3, 27.6, 26.2, 20.0, 13.6, 12.6, 10.8 ppm;

HRMS (EI): $C_{10}H_{19}O$ $[M–Et]^+$ calculated: 155.1436, found: 155.1447;

R*f* = 0.2 (PE:MTBE 95:5, vanillin);

$[\alpha]_D^{20}$ = –18.2 (*c* 1.0, $CHCl_3$).

Analytical data are in accordance with the literature.[10]

Serricornin (20)

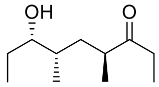

serricornin (**20**)
$C_{11}H_{22}O_2$
186.30

A solution of alcohol **19** (50 mg, 0.27 mmol, 1.0 eq.) in CH_2Cl_2 (2.7 mL) was treated with a dilute stream of ozone at −78 °C until the light blue color persisted. The solution was then purged with oxygen until the blue color disappeared and treated with PPh_3 (142 mg, 0.54 mmol, 2.0 eq.). The reaction mixture was allowed to slowly reach rt overnight (with cooling bath). Silica gel was added, the solvent was removed *in vacuo* and the crude product was purified by flash column chromatography (PE:MTBE 9:1) to afford serricornin (**20**) (37 mg, 0.20 mmol, 74%, mixture of open chain **a** and hemiketal form **b** 19:81, *dr* 18:1 hemiketal form) as a colorless oil.

^{1}H-NMR (400 MHz, C_6D_6): δ = 3.81 (m_c, 0.81H, **b**), 3.10 (br, 0.19H, **a**), 2.39-0.70 (m, 21H) ppm;

^{13}C-NMR (101 MHz, C_6D_6, assigned to **a**): *δ* = 213.1, 76.2, 43.7, 36.8, 35.8, 33.9, 27.5, 16.4, 13.6, 10.8, 8.1 ppm;

^{13}C-NMR (101 MHz, C_6D_6, assigned to **b**): *δ* = 98.5, 72.6, 36.1, 33.0, 31.3, 30.3, 26.2, 16.8, 11.7, 10.7, 7.3 ppm;

HRMS (EI): $C_{11}H_{20}O$ [M-H_2O]$^{\bullet+}$ calculated: 168.1514, found: 168.1512;

R_f = 0.3 (PE:MTBE 9:1, vanillin);

$[\alpha]_D^{20}$ = –38.0 (*c* 0.7, MeOH).

Analytical data are in accordance with the literature.[16]

4 References

[1] R. C. Mykura, S. Veth, A. Varela, L. Dewis, J. J. Farndon, E. L. Myers, V. K. Aggarwal, *J. Am. Chem. Soc.* **2018**, *140*, 14677.

[2] D. J. Blair, D. Tanini, J. M. Bateman, H. K. Scott, E. L. Myers, V. K. Aggarwal, *Chem. Sci.* **2017**, *8*, 2898.

[3] P. Beak, L. G. Carter, *J. Org. Chem.* **1981**, *46*, 2363.

[4] M. Burns, S. Essafi, J. R. Bame, S. P. Bull, M. P. Webster, S. Balieu, J. W. Dale, C. P. Butts, J. N. Harvey, V. K. Aggarwal, *Nature* **2014**, *513*, 183.

[5] J. L.-Y. Chen, H. K. Scott, M. J. Hesse, C. L. Willis, V. K. Aggarwal, *J. Am. Chem. Soc.* **2013**, *135*, 5316.

[6] G. Casoni, M. Kucukdisli, J. M. Fordham, M. Burns, E. L. Myers, V. K. Aggarwal, *J. Am. Chem. Soc.* **2017**, *139*, 11877.

[7] A. Noble, S. Roesner, V. K. Aggarwal, *Angew. Chem. Int. Ed.* **2016**, *55*, 15920; *Angew. Chem.* **2016**, *128*, 16152.

[8] pinBCH$_2$Bpin can be purchased from Allychem or prepared on multigram scale; see K. Hong, X. Liu, J. P. Morken, *J. Am. Chem. Soc.* **2014**, *136*, 10581.

[9] Due to variations at λ = 589 nm, the optical rotation was measured at λ = 365 nm.

[10] D. S. Matteson, R. P. Singh, B. Schafman, J.-j. Yang, *J. Org. Chem.* **1998**, *63*, 4466.

[11] Prepared on multigrain scale according to a) A. M. Fournier, J. Clayden, *Org. Lett.* **2012**, *14*, 142; b) N. J. Webb, S. P. Marsden, S. A. Raw, *Org. Lett.* **2014**, *16*, 4718.

[12] a) A. G. Myers, B. H. Yang, H. Chen, L. McKinstry, D. J. Kopecky, J. L. Gleason, *J. Am. Chem. Soc.* **1997**, *119*, 6496; b) A. Myers, B. Yang, H. Chen, D. Kopecky, *Synlett* **1997**, *5*, 457.

[13] J. Bergmann, C. Löfstedt, V. D. Ivanov, W. Francke, *Eur. J. Org. Chem.* **2001**, *2001*, 3175.

[14] Di Wang, X.-S. Xue, K. N. Houk, Z. Shi, *Angew. Chem. Int. Ed.* **2018**, *57*, 16861; *Angew. Chem.* **2018**, *130*, 17103.

[15] a) J. Rocca, J. Tumlinson, B. Glancey, C. Lofgren, *Tetrahedron Lett.* **1983**, *24*, 1893; b) F. E. Ziegler, W. T. Cain, A. Kneisley, E. P. Stirchak, R. T. Wester, *J. Am. Chem. Soc.* **1988**, *110*, 5442.

[16] K. Mori, H. Watanabe, *Tetrahedron* **1985**, *41*, 3423.

5 Spectra

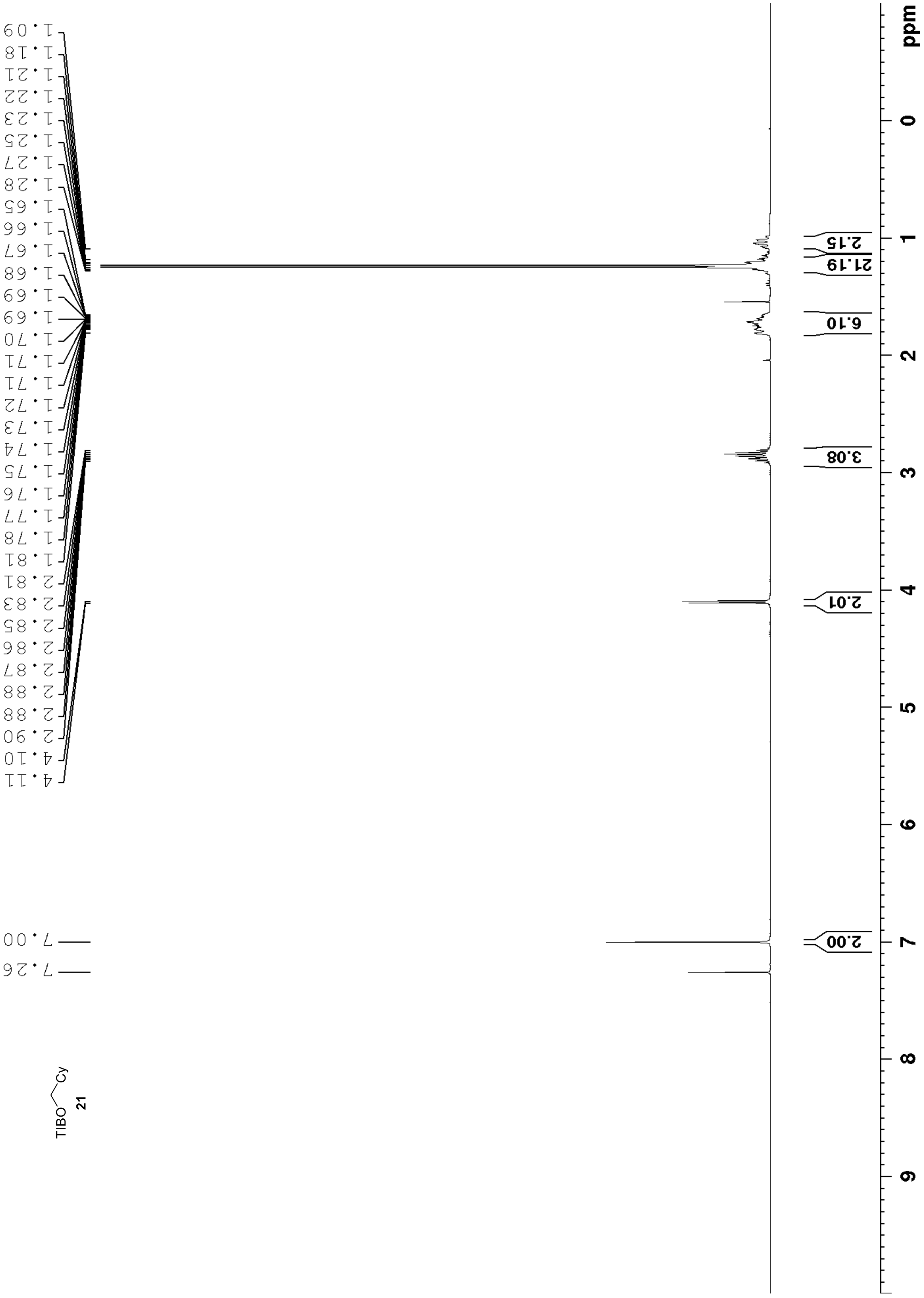
1.09
1.18
1.21
1.22
1.23
1.25
1.27
1.28
1.65
1.66
1.67
1.68
1.69
1.69
1.70
1.71
1.71
1.72
1.73
1.74
1.75
1.76
1.77
1.78
1.81
2.81
2.83
2.85
2.86
2.87
2.88
2.88
2.90
4.10
4.11
7.00
7.26
2.15
21.19
6.10
3.08
2.01
2.00
ppm
0
1
2
3
4
5
6
7
8
9
TIBO
Cy
21

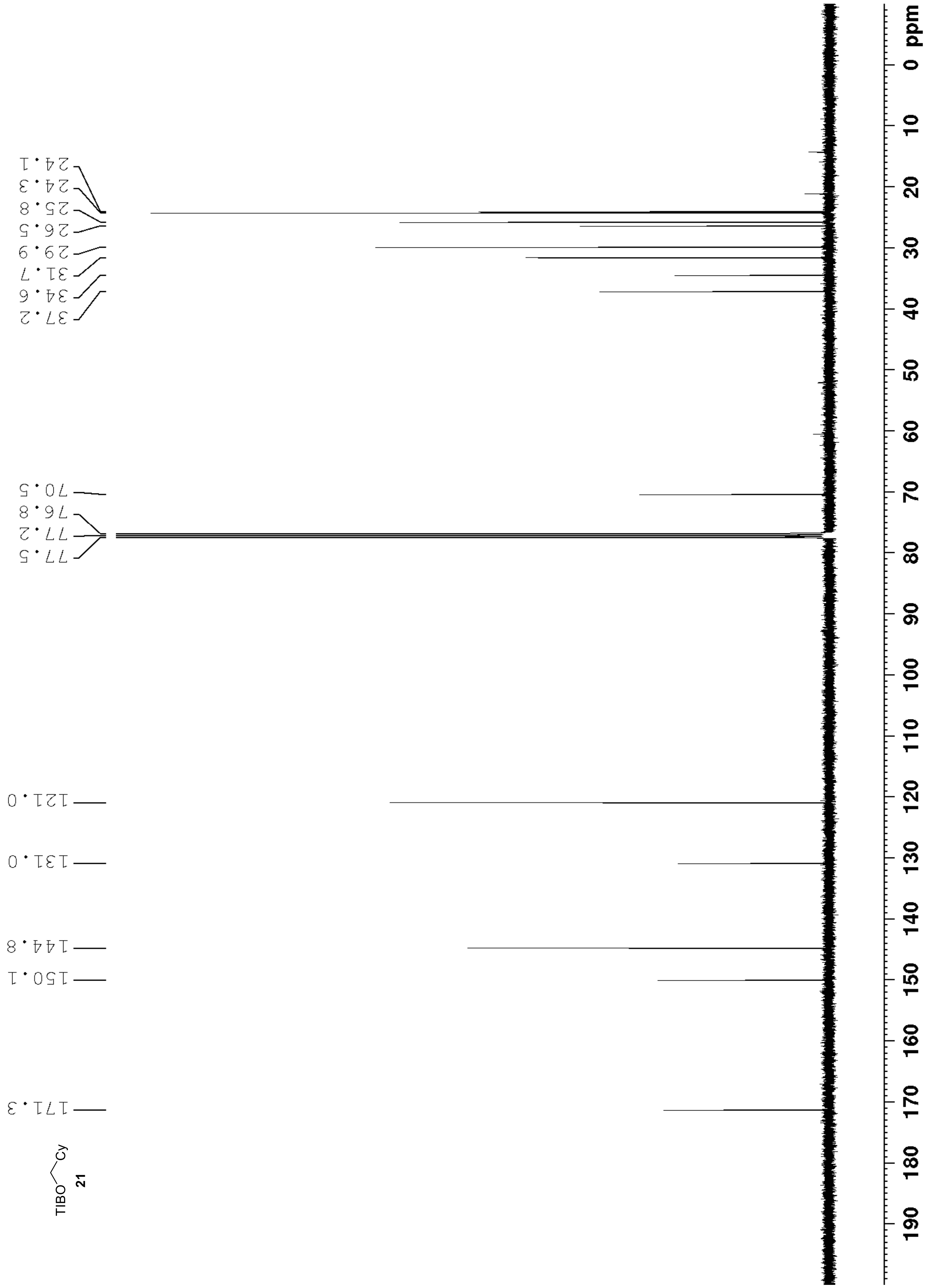
24.1
24.3
25.8
26.5
29.9
31.7
34.6
37.2
70.5
76.8
77.2
77.5
121.0
131.0
144.8
150.1
171.3
TIBO Cy
21
0 ppm
10
20
30
40
50
60
70
80
90
100
110
120
130
140
150
160
170
180
190

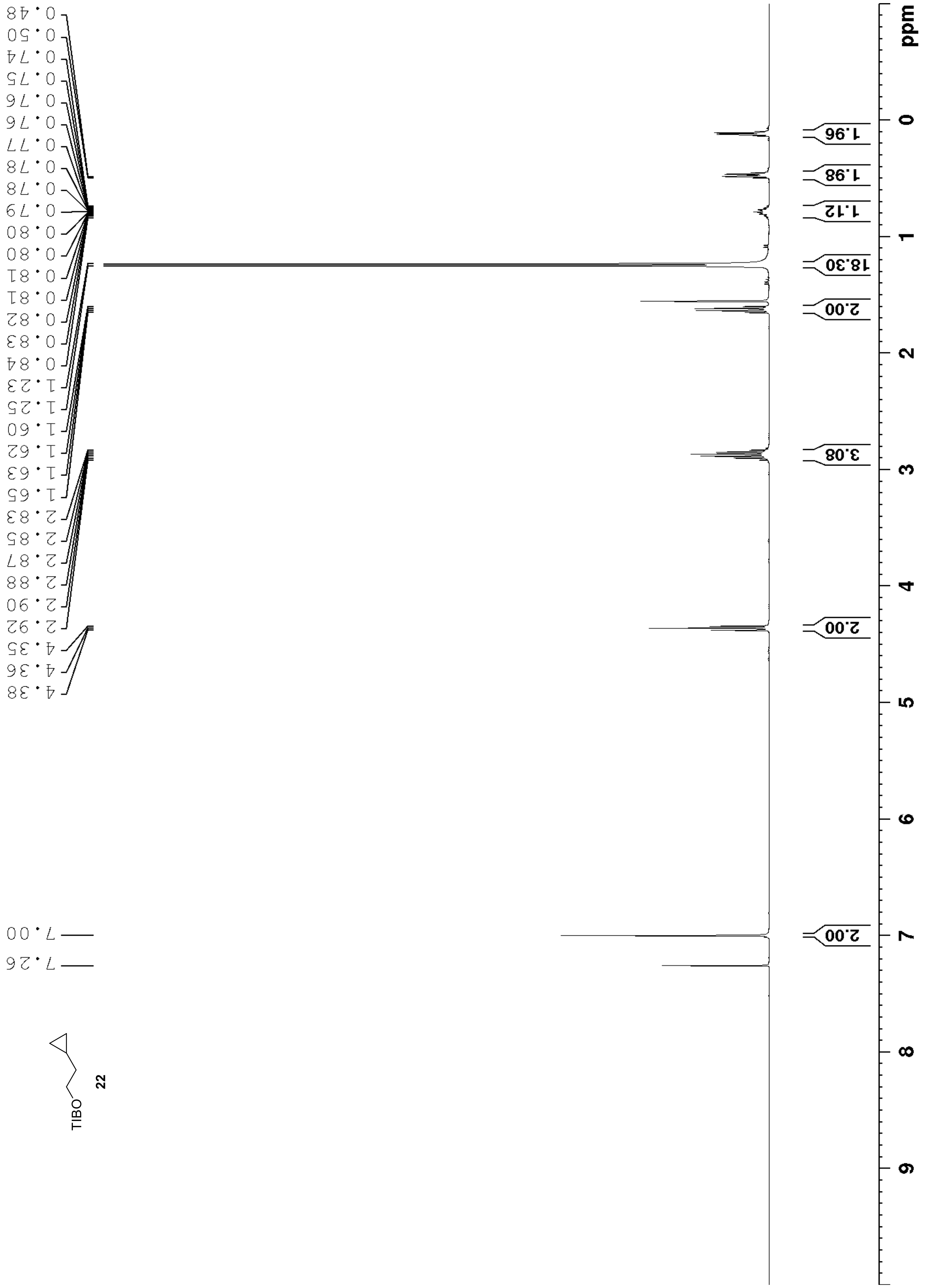
ppm
1.96
1.98
1.12
18.30
2.00
3.08
2.00
2.00
TIBO
22

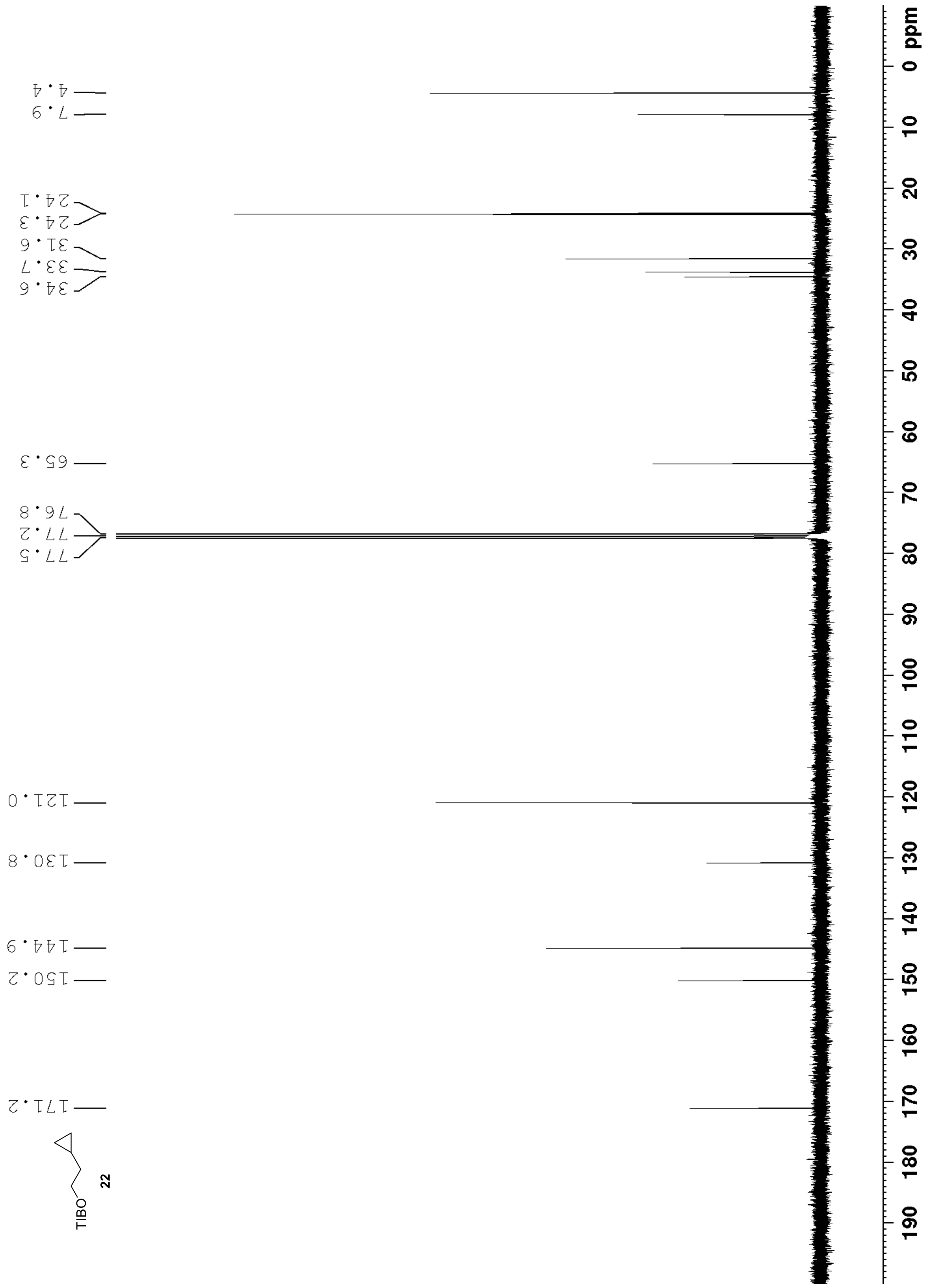
4.4
7.9
24.1
24.3
31.6
33.7
34.6
65.3
76.8
77.2
77.5
121.0
130.8
144.9
150.2
171.2
TIBO
22
0 ppm
10
20
30
40
50
60
70
80
90
100
110
120
130
140
150
160
170
180
190

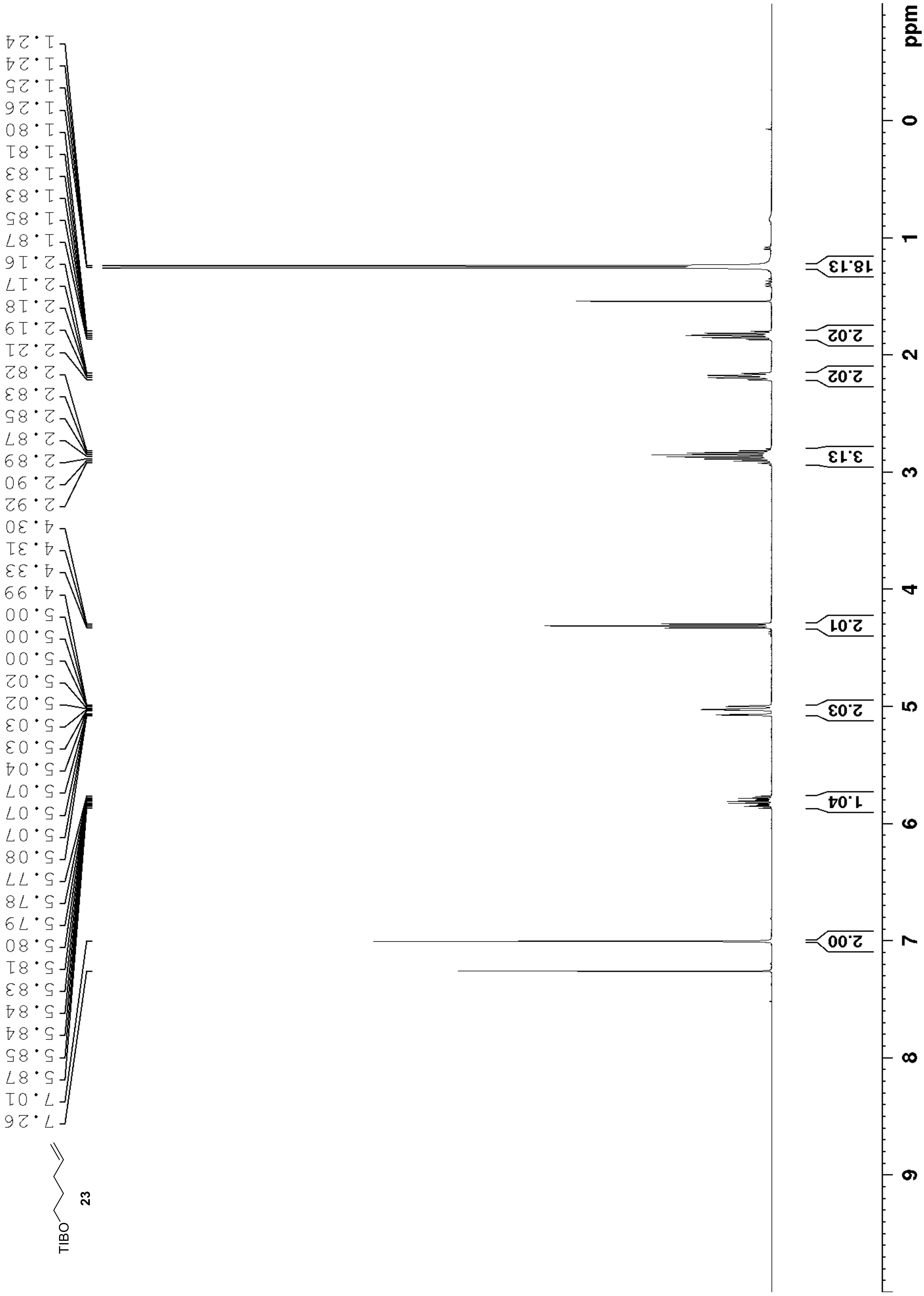
1.24
1.24
1.25
1.26
1.80
1.81
1.83
1.83
1.85
1.87
2.16
2.17
2.18
2.19
2.21
2.82
2.83
2.85
2.87
2.89
2.90
2.92
4.30
4.31
4.33
4.99
5.00
5.00
5.00
5.02
5.02
5.03
5.03
5.04
5.07
5.07
5.07
5.08
5.77
5.78
5.79
5.80
5.81
5.83
5.84
5.84
5.85
5.87
7.01
7.26
TIBO
23
18.13
2.02
2.02
3.13
2.01
2.03
1.04
2.00
ppm
0
1
2
3
4
5
6
7
8
9

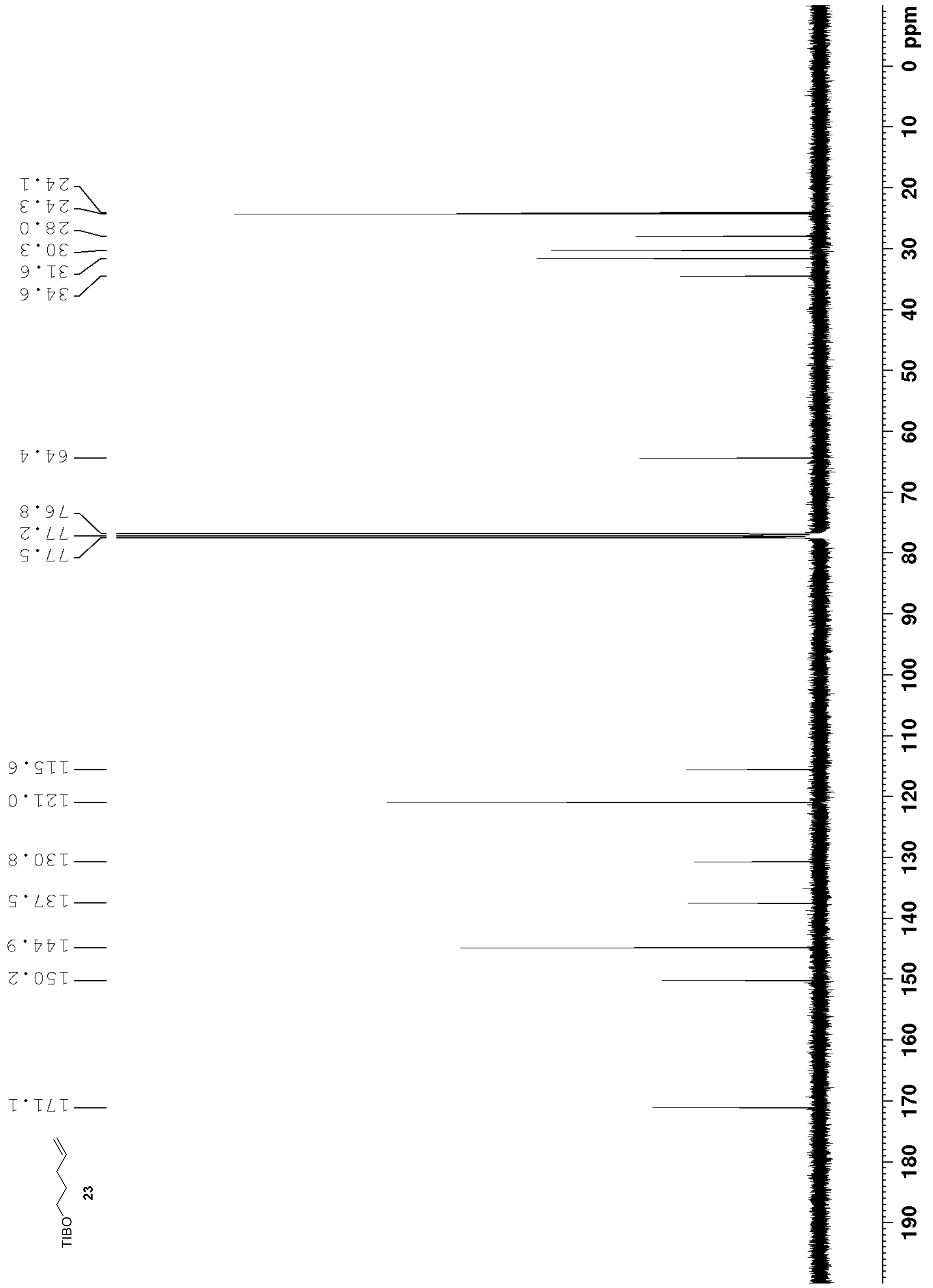
24.1
24.3
28.0
30.3
31.6
34.6
64.4
76.8
77.2
77.5
115.6
121.0
130.8
137.5
144.9
150.2
171.1
TIBO
23
ppm

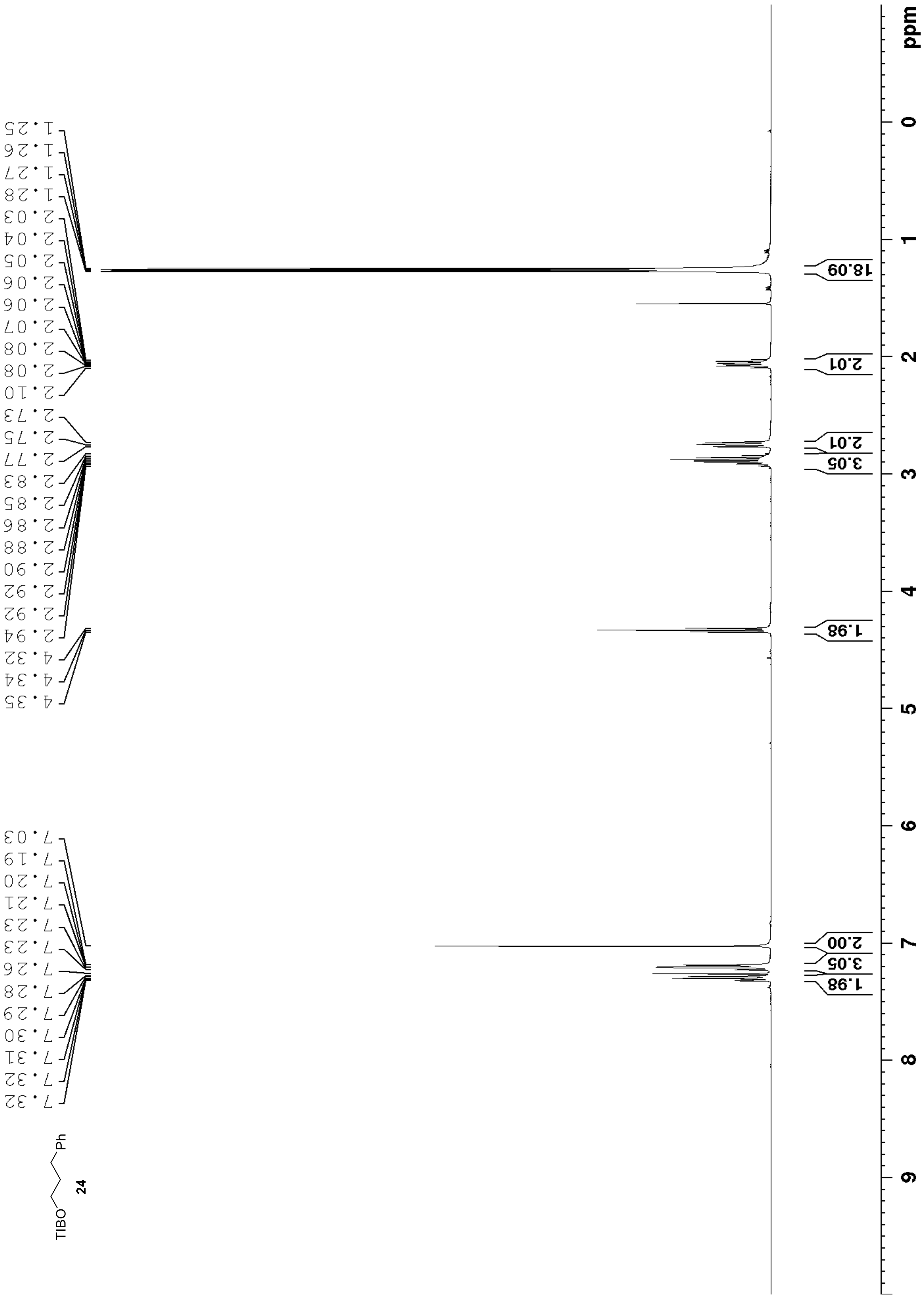
1.25
1.26
1.27
1.28
2.03
2.04
2.05
2.06
2.06
2.07
2.08
2.08
2.10
2.73
2.75
2.77
2.83
2.85
2.86
2.88
2.90
2.92
2.92
2.94
4.32
4.34
4.35
7.03
7.19
7.20
7.21
7.23
7.23
7.26
7.28
7.29
7.30
7.31
7.32
7.32
TIBO
Ph
24
18.09
2.01
2.01
3.05
1.98
2.00
3.05
1.98
ppm
0
1
2
3
4
5
6
7
8
9

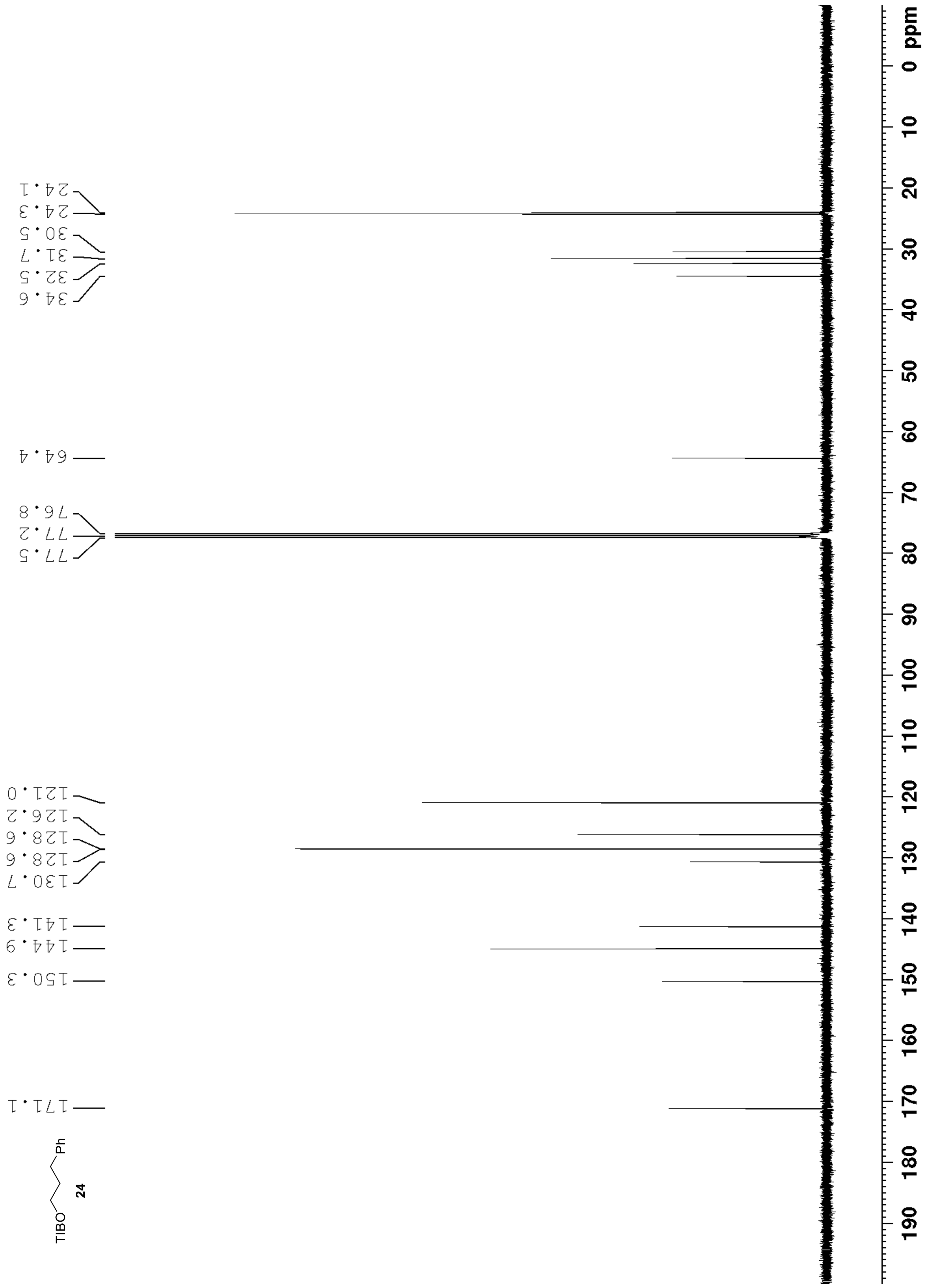
24.1
24.3
30.5
31.7
32.5
34.6
64.4
76.8
77.2
77.5
121.0
126.2
128.6
128.6
130.7
141.3
144.9
150.3
171.1
TIBO
Ph
24
ppm

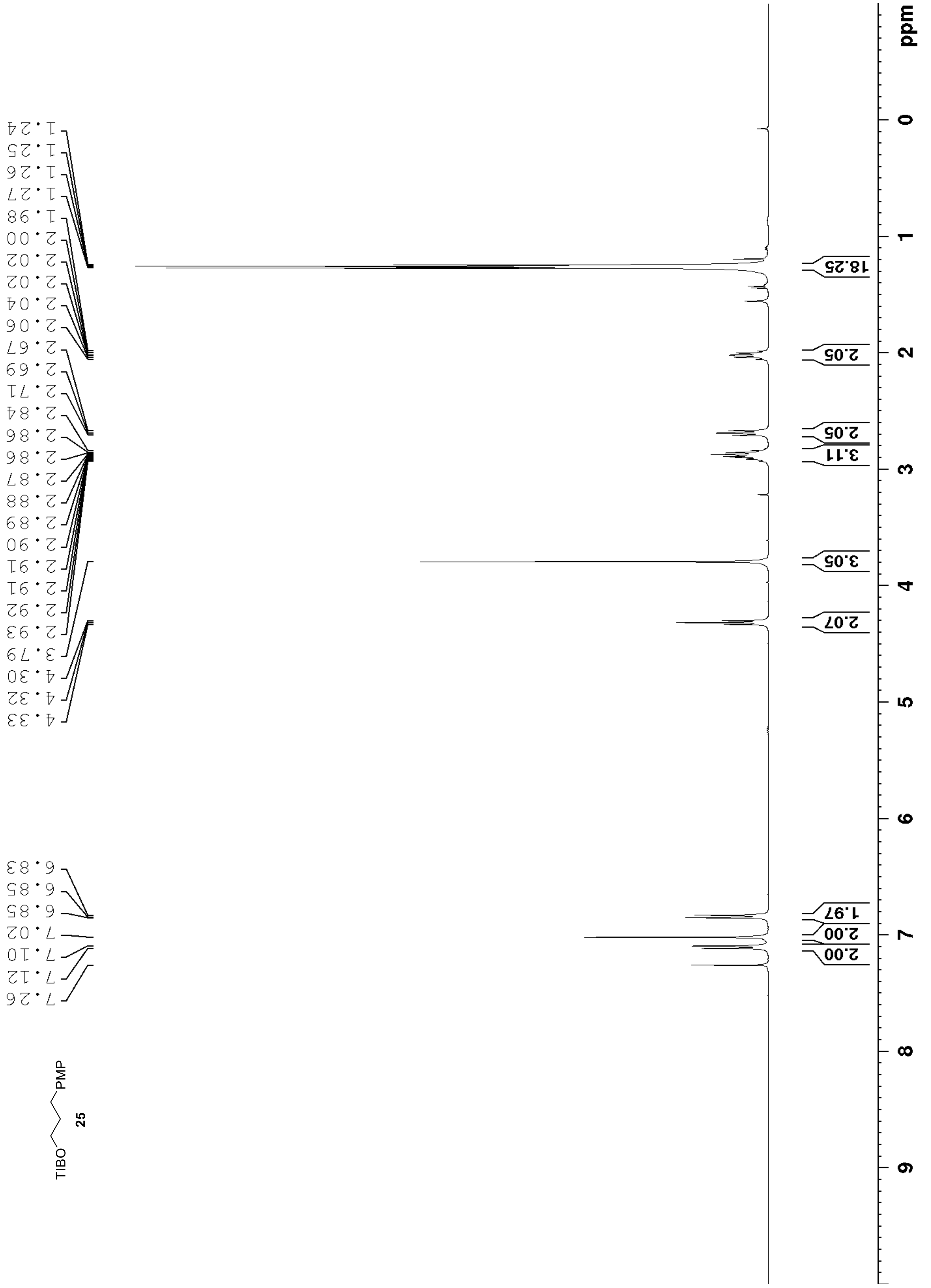
TIBO
PMP
25
1.24
1.25
1.26
1.27
1.98
2.00
2.02
2.02
2.04
2.06
2.67
2.69
2.71
2.84
2.86
2.86
2.87
2.88
2.89
2.90
2.91
2.91
2.92
2.93
3.79
4.30
4.32
4.33
6.83
6.85
6.85
7.02
7.10
7.12
7.26
18.25
2.05
2.05
3.11
3.05
2.07
1.97
2.00
2.00
ppm
0
1
2
3
4
5
6
7
8
9

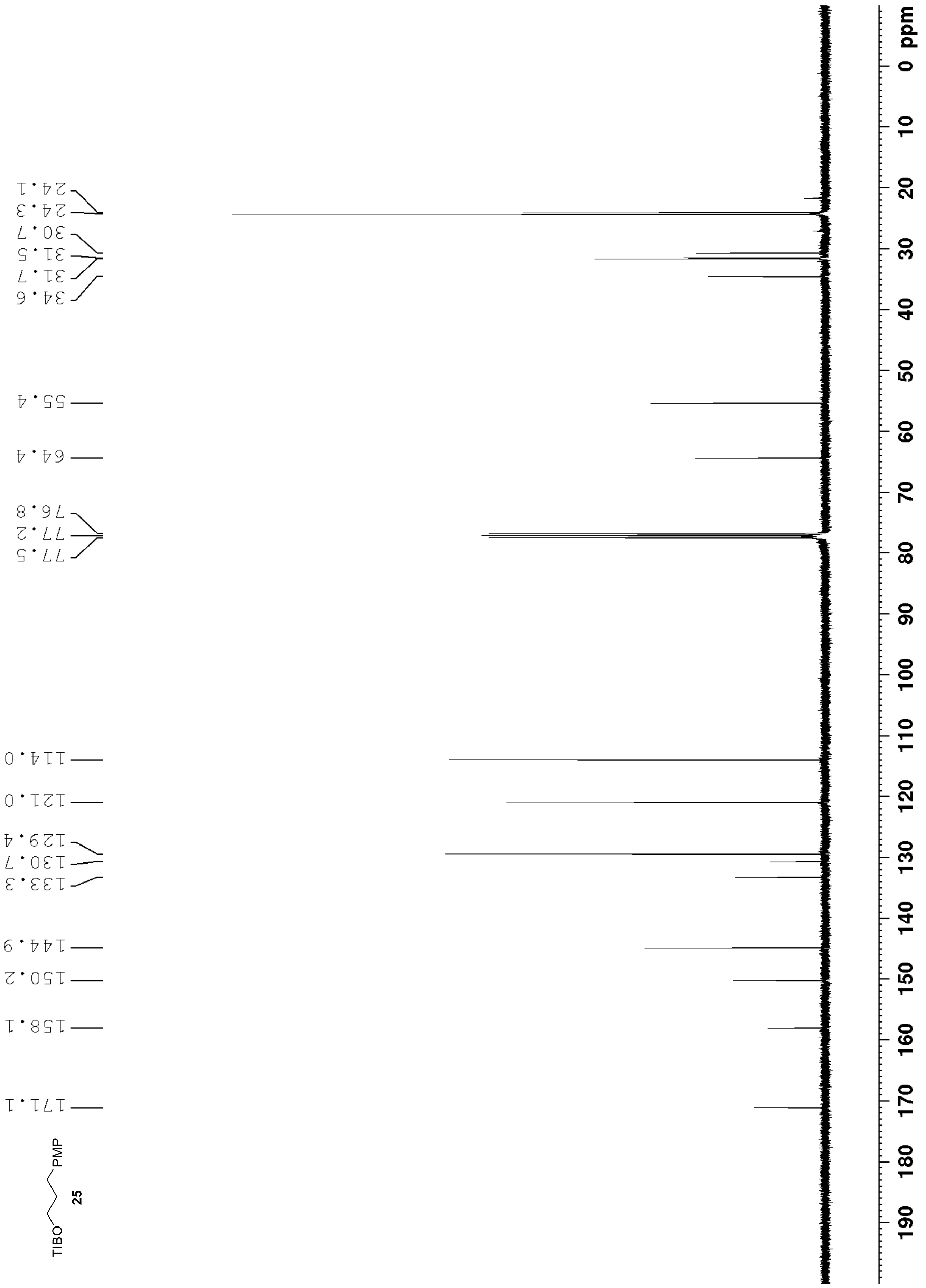
24.1
24.3
30.7
31.5
31.7
34.6
55.4
64.4
76.8
77.2
77.5
114.0
121.0
129.4
130.7
133.3
144.9
150.2
158.1
171.1
TIBO
PMP
25
ppm
0
10
20
30
40
50
60
70
80
90
100
110
120
130
140
150
160
170
180
190

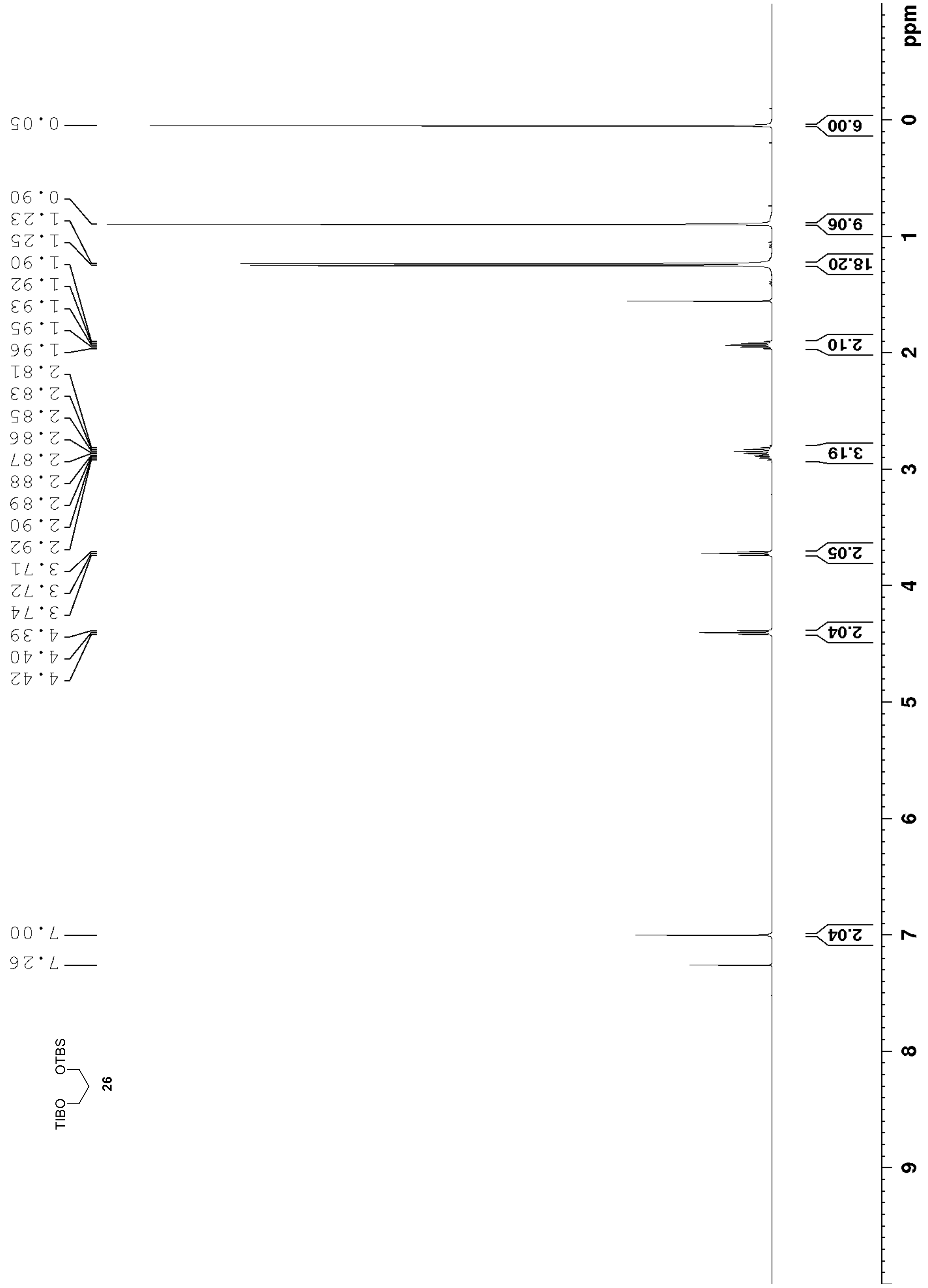
0.05
0.90
1.23
1.25
1.90
1.92
1.93
1.95
1.96
2.81
2.83
2.85
2.86
2.87
2.88
2.89
2.90
2.92
3.71
3.72
3.74
4.39
4.40
4.42
7.00
7.26
6.00
9.06
18.20
2.10
3.19
2.05
2.04
2.04
ppm
TIBO
OTBS
26

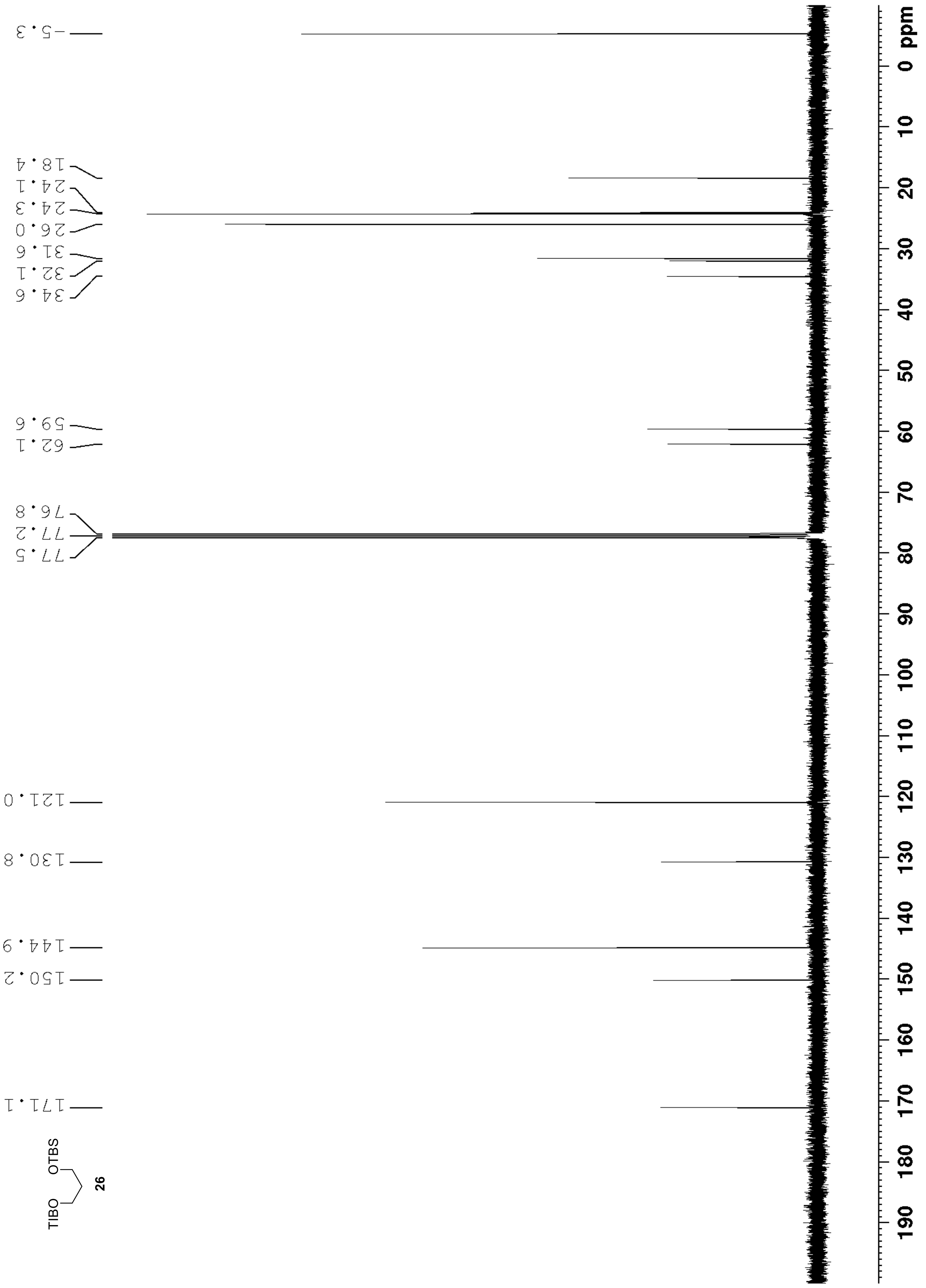
-5.3
18.4
24.1
24.3
26.0
31.6
32.1
34.6
59.6
62.1
76.8
77.2
77.5
121.0
130.8
144.9
150.2
171.1
TIBO
OTBS
26
ppm
0
10
20
30
40
50
60
70
80
90
100
110
120
130
140
150
160
170
180
190

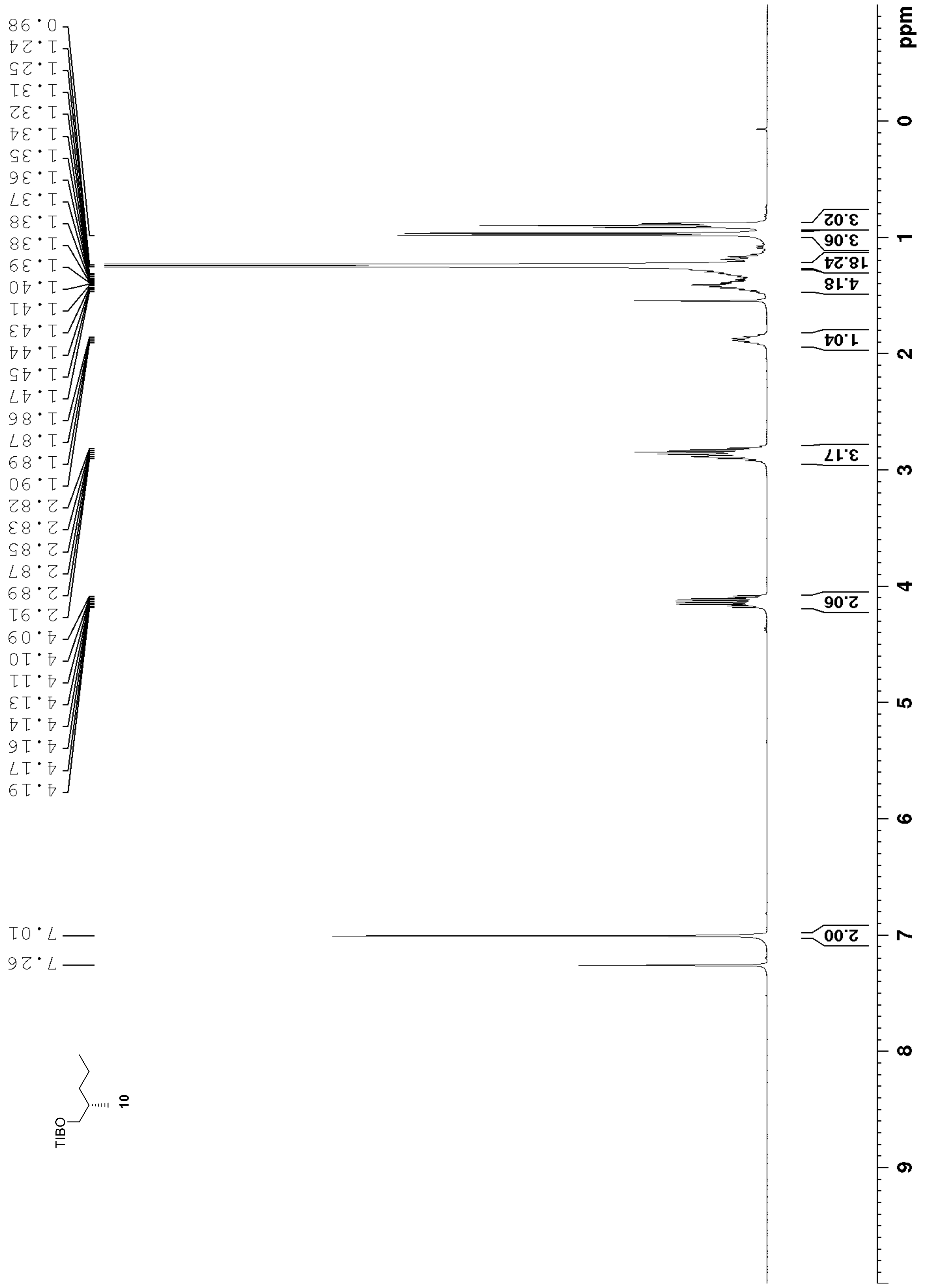
0.98
1.24
1.25
1.31
1.32
1.34
1.35
1.36
1.37
1.38
1.38
1.39
1.40
1.41
1.43
1.44
1.45
1.47
1.86
1.87
1.89
1.90
2.82
2.83
2.85
2.87
2.89
2.91
4.09
4.10
4.11
4.13
4.14
4.16
4.17
4.19
7.01
7.26
3.02
3.06
18.24
4.18
1.04
3.17
2.06
2.00
ppm
TIBO
10

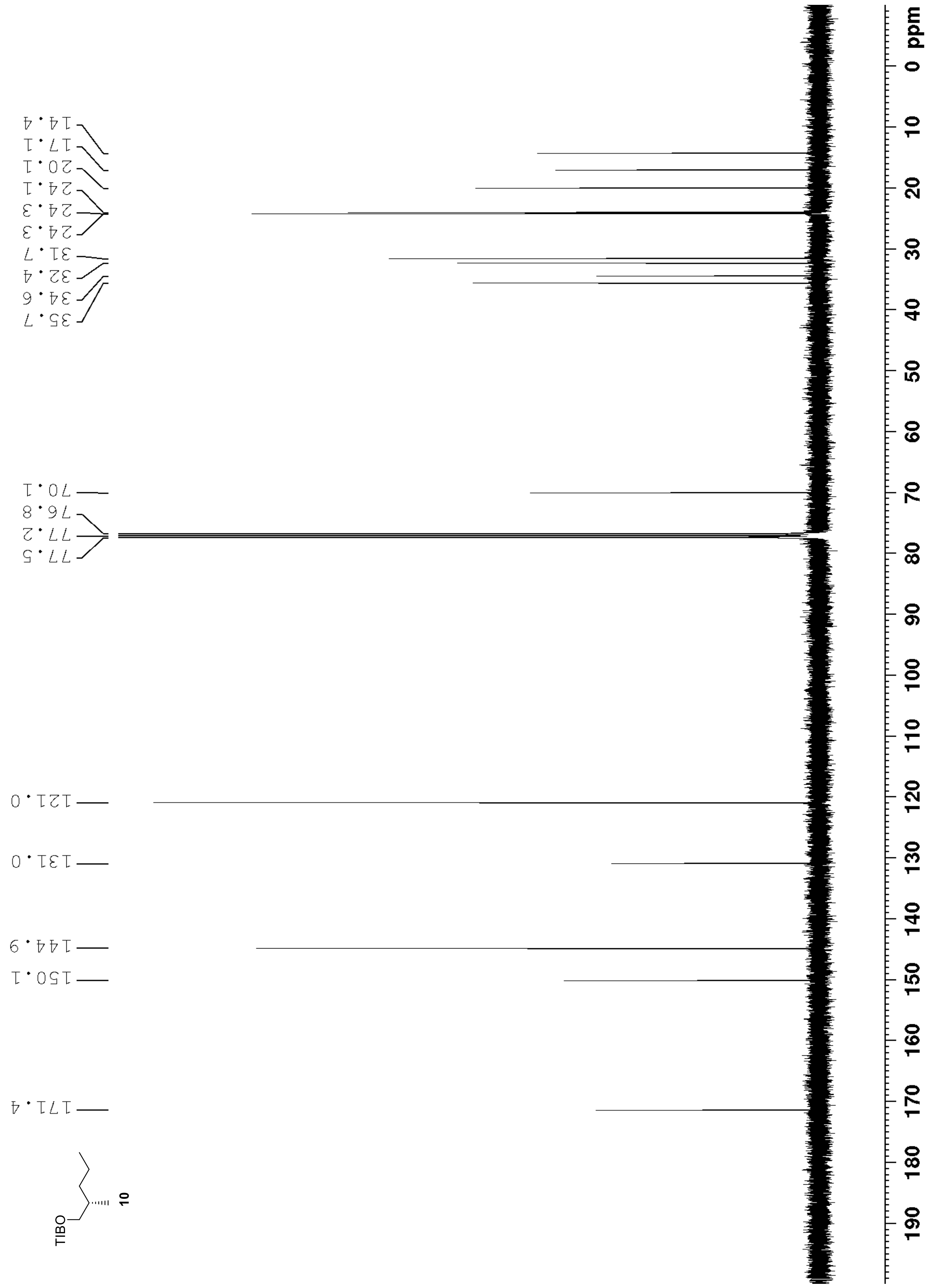
14.4
17.1
20.1
24.1
24.3
24.3
31.7
32.4
34.6
35.7
70.1
76.8
77.2
77.5
121.0
131.0
144.9
150.1
171.4
TIBO
10
ppm

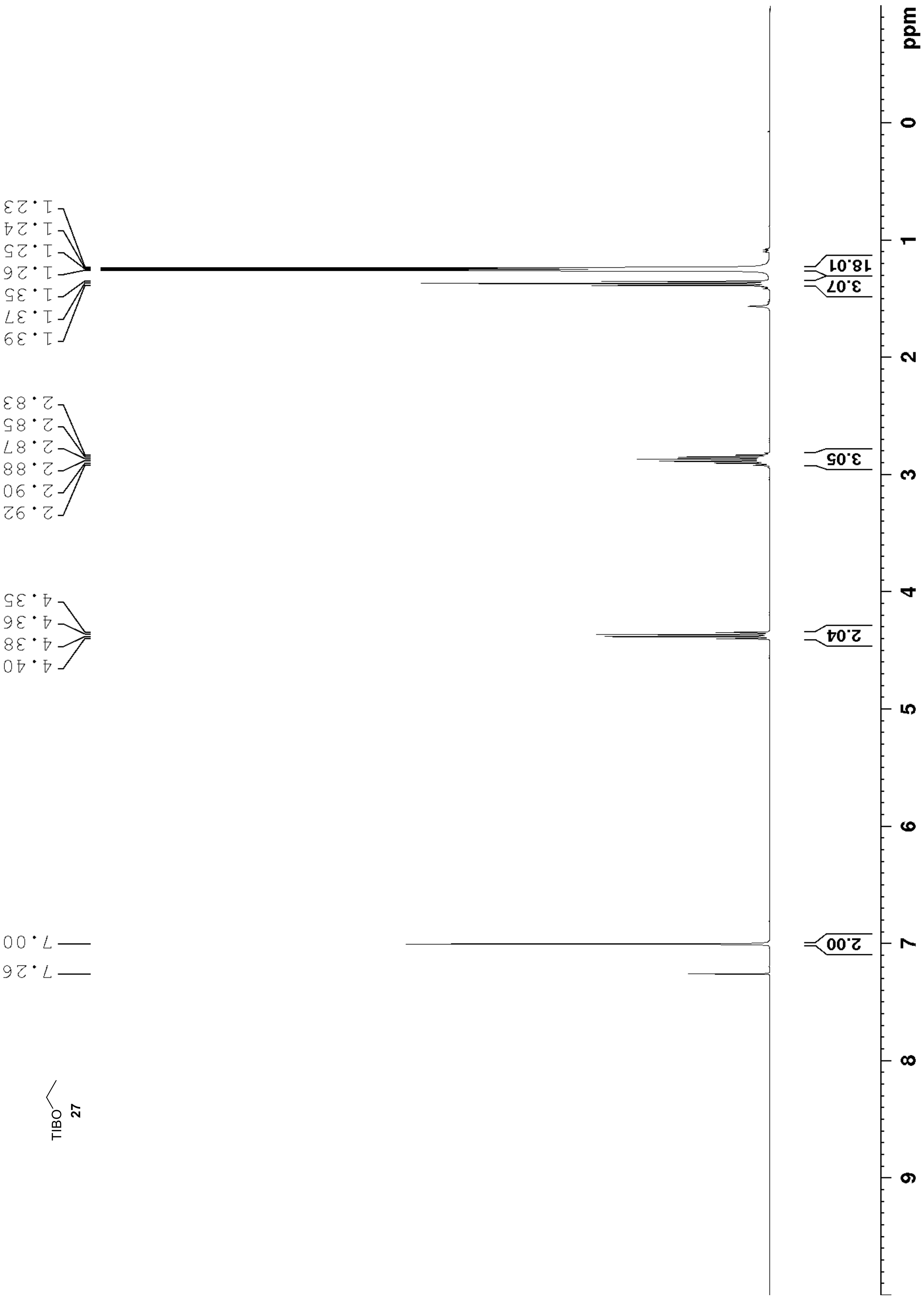
1.23
1.24
1.25
1.26
1.35
1.37
1.39
2.83
2.85
2.87
2.88
2.90
2.92
4.35
4.36
4.38
4.40
7.00
7.26
TIBO
27
18.01
3.07
3.05
2.04
2.00
ppm

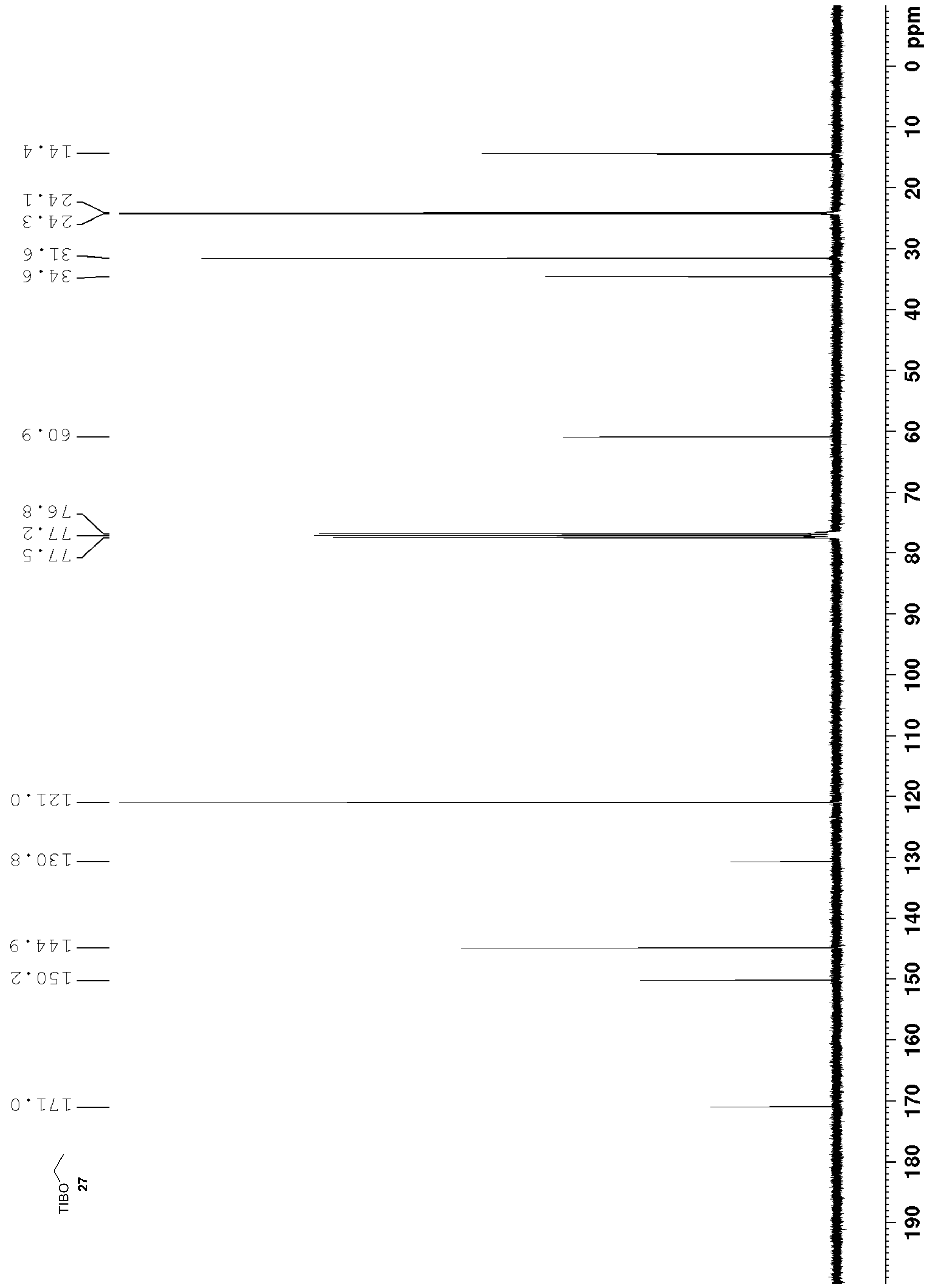
14.4
24.1
24.3
31.6
34.6
60.9
76.8
77.2
77.5
121.0
130.8
144.9
150.2
171.0
TIBO
27
0 ppm
10
20
30
40
50
60
70
80
90
100
110
120
130
140
150
160
170
180
190

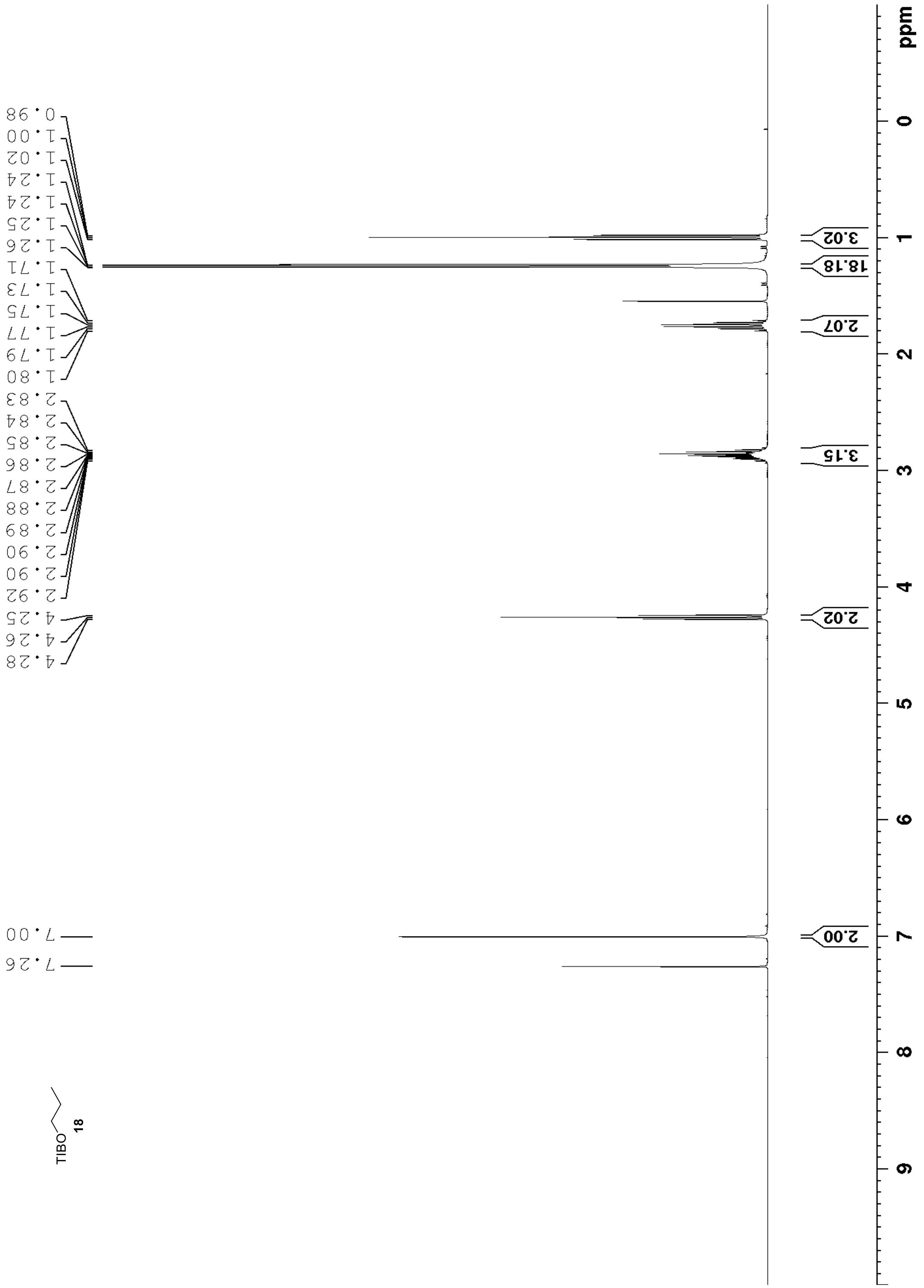
0.98
1.00
1.02
1.24
1.24
1.25
1.26
1.71
1.73
1.75
1.77
1.79
1.80
2.83
2.84
2.85
2.86
2.87
2.88
2.89
2.90
2.90
2.92
4.25
4.26
4.28
7.00
7.26
3.02
18.18
2.07
3.15
2.02
2.00
TIBO
18
ppm
0
1
2
3
4
5
6
7
8
9

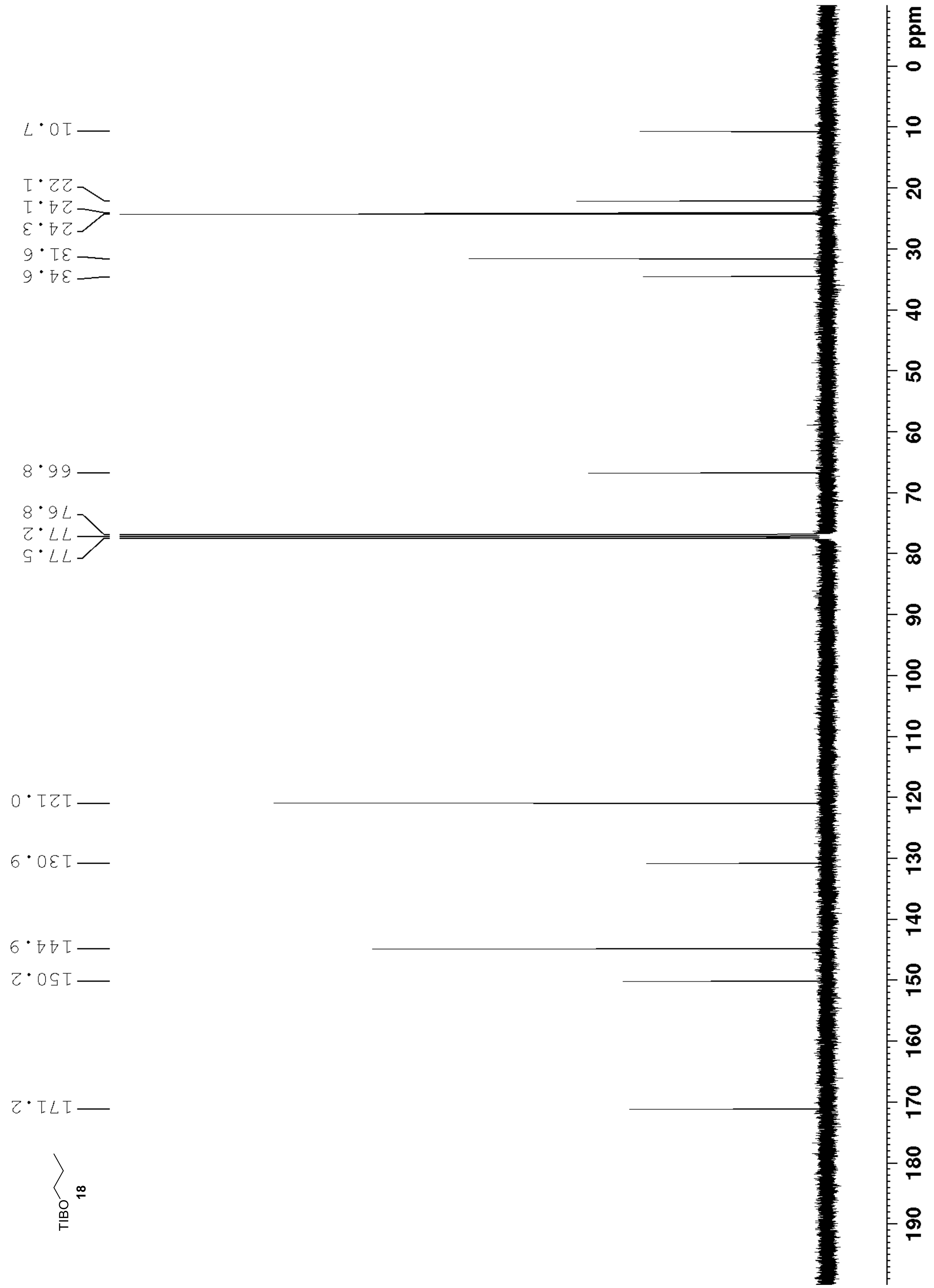
10.7
22.1
24.1
24.3
31.6
34.6
66.8
76.8
77.2
77.5
121.0
130.9
144.9
150.2
171.2
TIBO
18
ppm

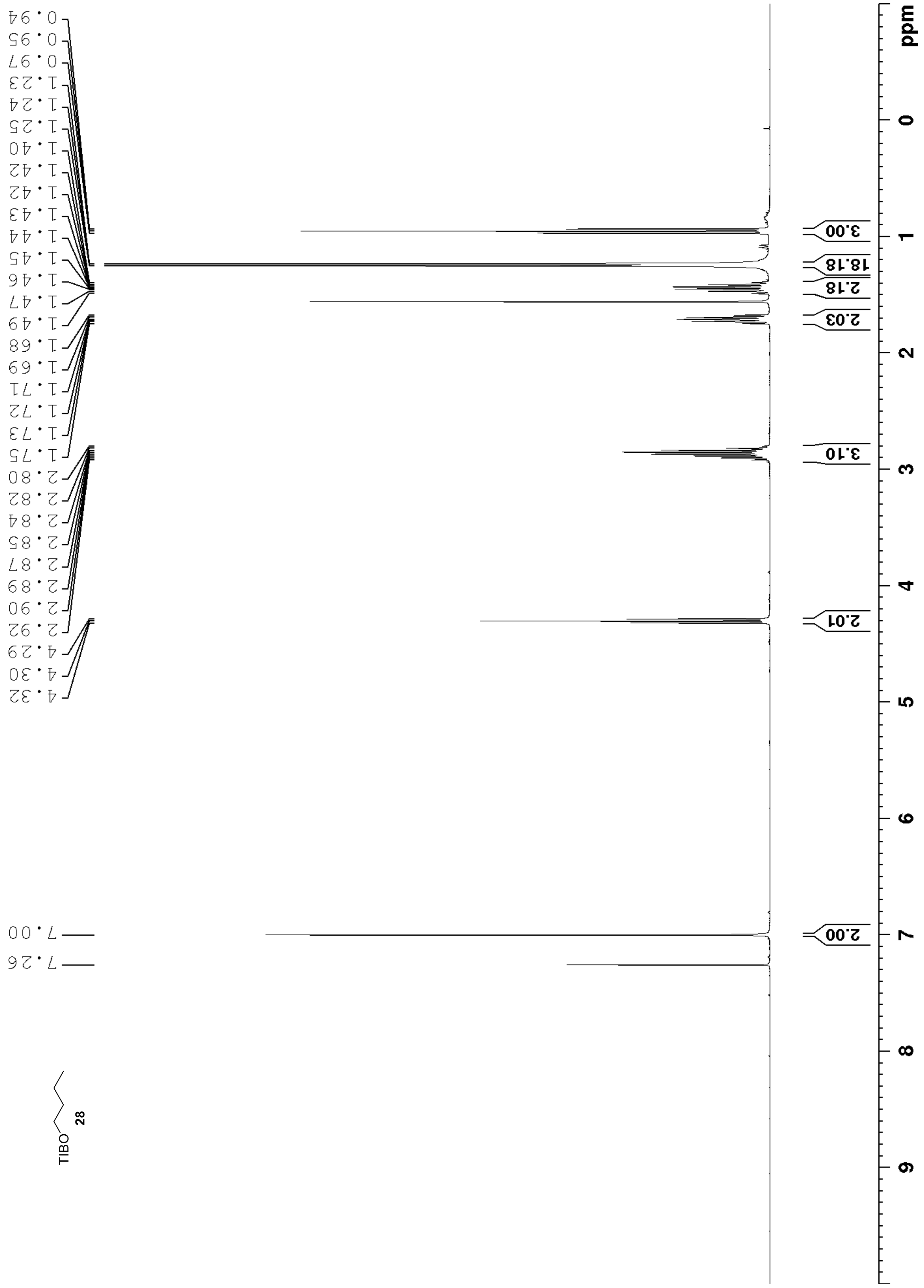
0.94
0.95
0.97
1.23
1.24
1.25
1.40
1.42
1.42
1.43
1.44
1.45
1.46
1.47
1.49
1.68
1.69
1.71
1.72
1.73
1.75
2.80
2.82
2.84
2.85
2.87
2.89
2.90
2.92
4.29
4.30
4.32
7.00
7.26
3.00
18.18
2.18
2.03
3.10
2.01
2.00
TIBO
28
ppm
0
1
2
3
4
5
6
7
8
9

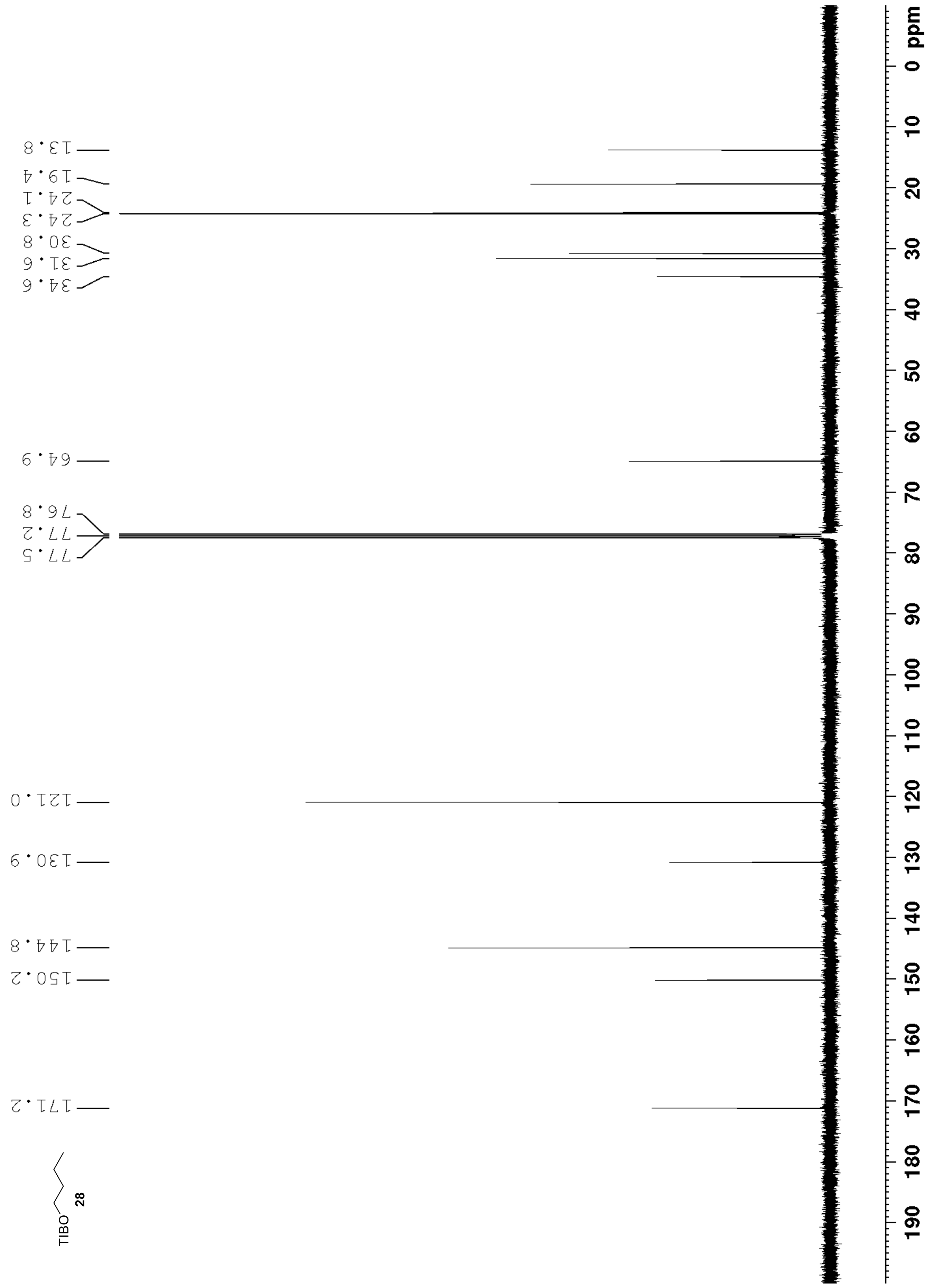
13.8
19.4
24.1
24.3
30.8
31.6
34.6
64.9
76.8
77.2
77.5
121.0
130.9
144.8
150.2
171.2
TIBO
28
ppm

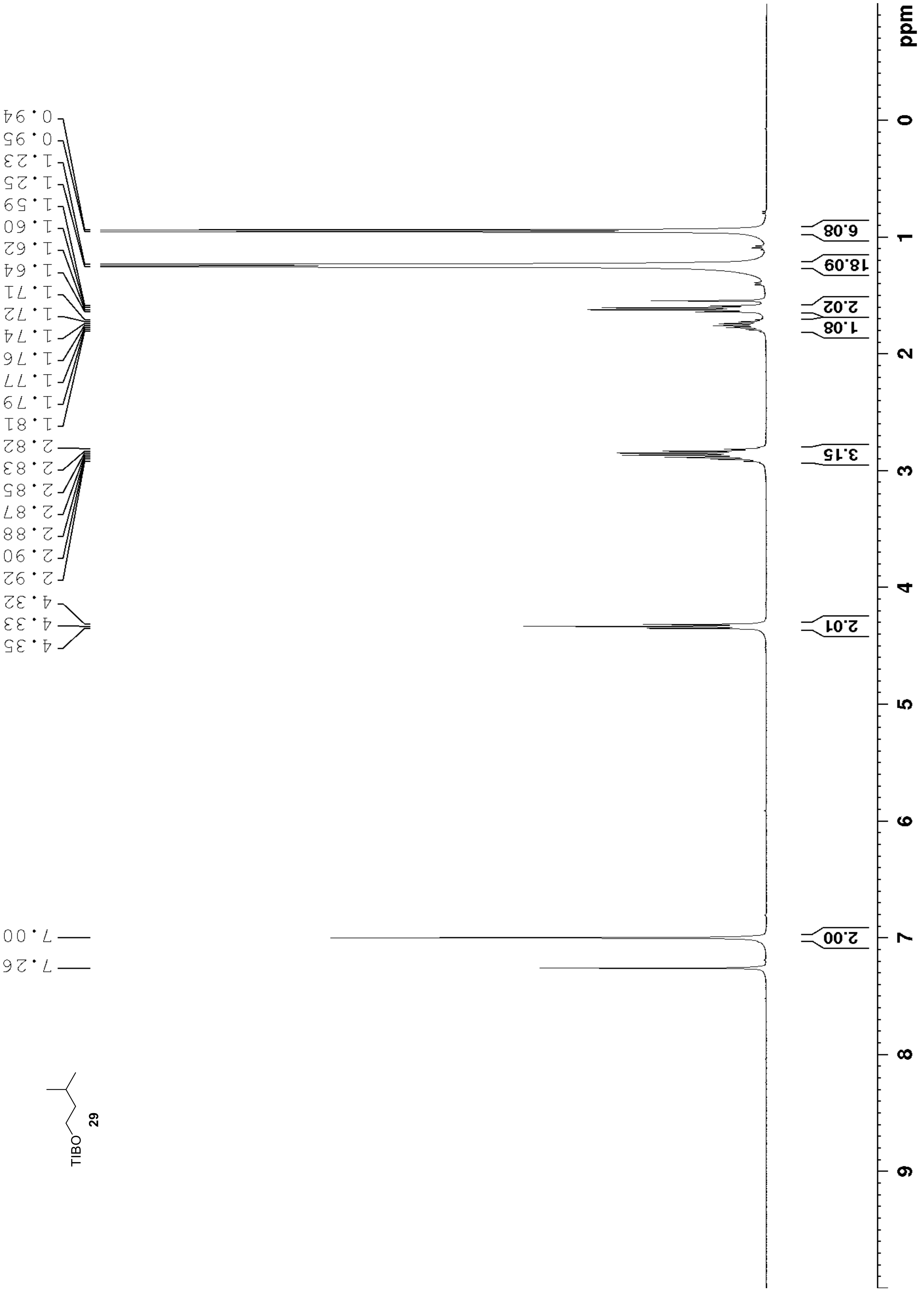
0.94
0.95
1.23
1.25
1.59
1.60
1.62
1.64
1.71
1.72
1.74
1.76
1.77
1.79
1.81
2.82
2.83
2.85
2.87
2.88
2.90
2.92
4.32
4.33
4.35
7.00
7.26
6.08
18.09
2.02
1.08
3.15
2.01
2.00
ppm
TIBO
29

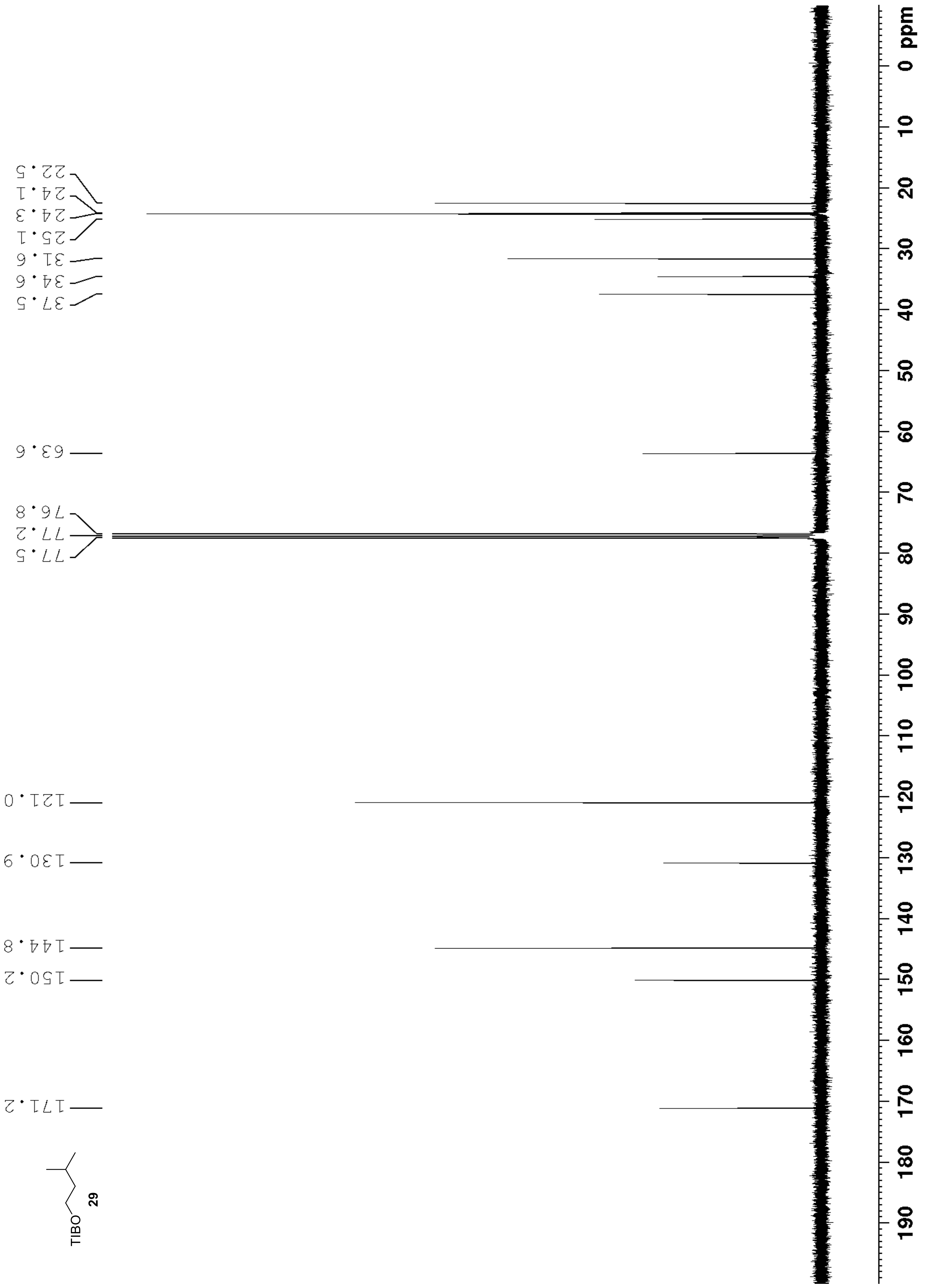
22.5
24.1
24.3
25.1
31.6
34.6
37.5
63.6
76.8
77.2
77.5
121.0
130.9
144.8
150.2
171.2
TIBO
29
ppm
0
10
20
30
40
50
60
70
80
90
100
110
120
130
140
150
160
170
180
190

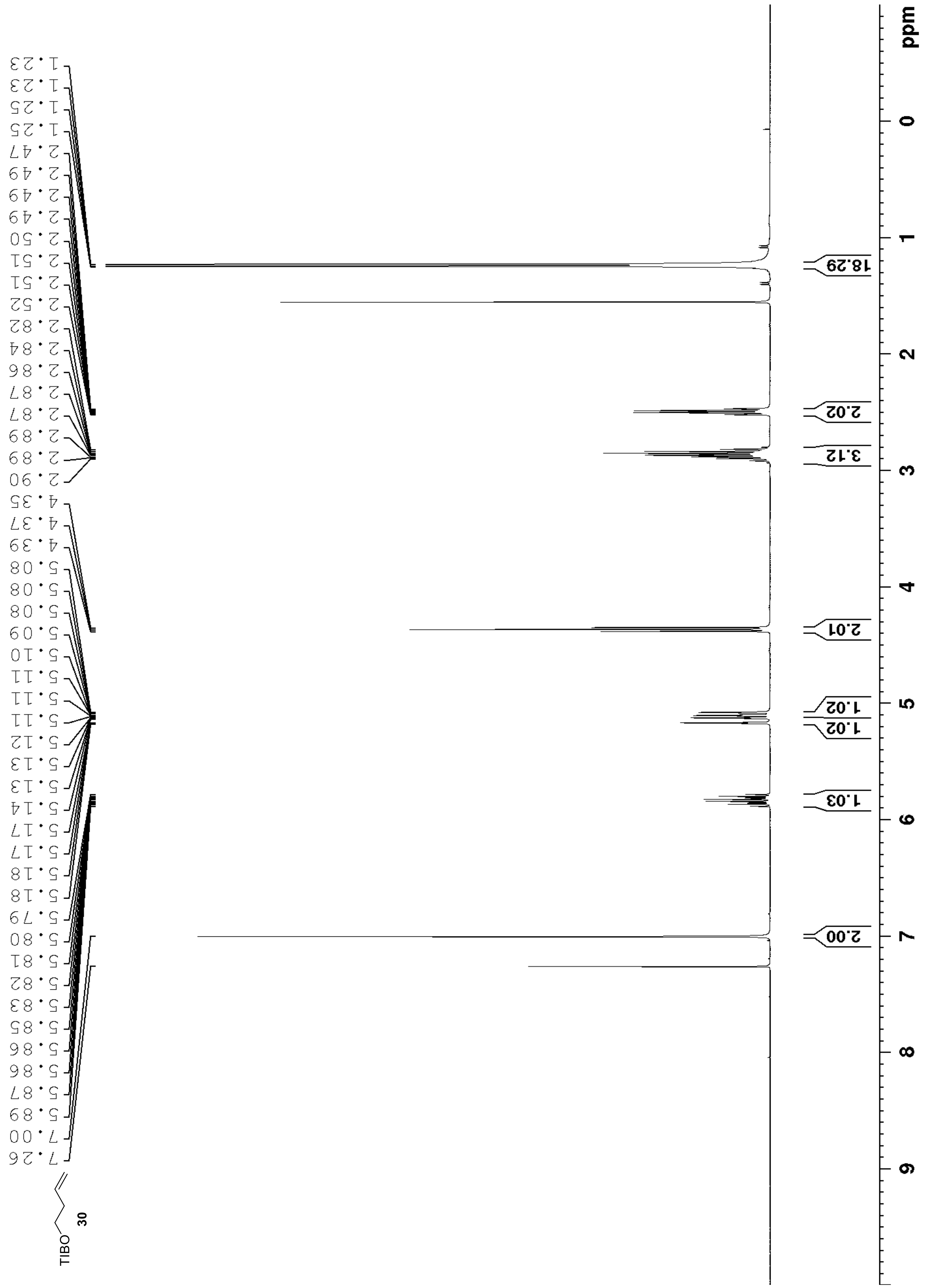
1.23
1.23
1.25
1.25
2.47
2.49
2.49
2.49
2.50
2.51
2.51
2.52
2.82
2.84
2.86
2.87
2.87
2.89
2.89
2.90
4.35
4.37
4.39
5.08
5.08
5.08
5.09
5.10
5.11
5.11
5.11
5.12
5.13
5.13
5.14
5.17
5.17
5.18
5.18
5.79
5.80
5.81
5.82
5.83
5.85
5.86
5.86
5.87
5.89
7.00
7.26
TIBO
30
18.29
2.02
3.12
2.01
1.02
1.02
1.03
2.00
ppm
0
1
2
3
4
5
6
7
8
9

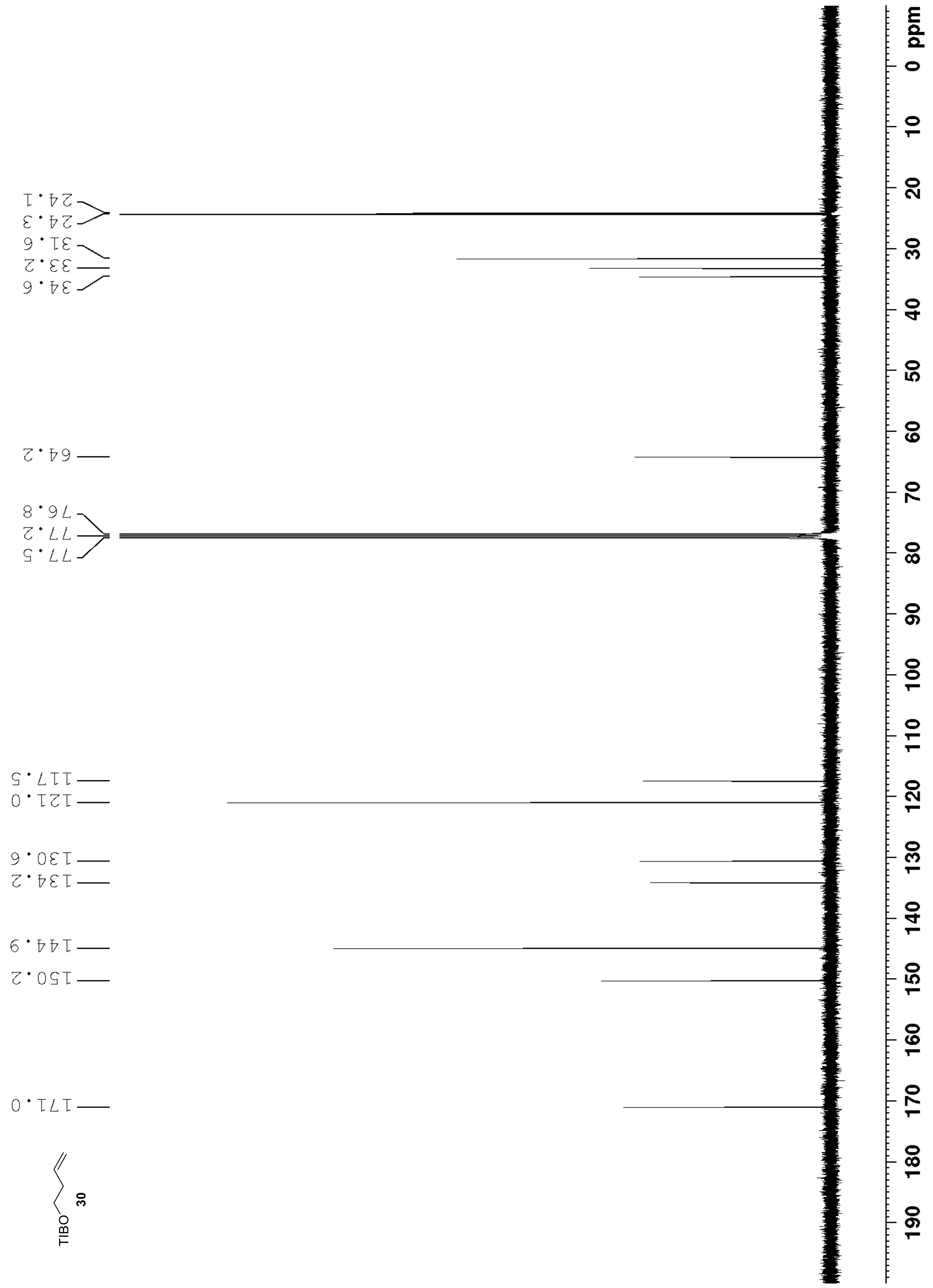
24.1
24.3
31.6
33.2
34.6
64.2
76.8
77.2
77.5
117.5
121.0
130.6
134.2
144.9
150.2
171.0
TIBO
30
ppm
0
10
20
30
40
50
60
70
80
90
100
110
120
130
140
150
160
170
180
190

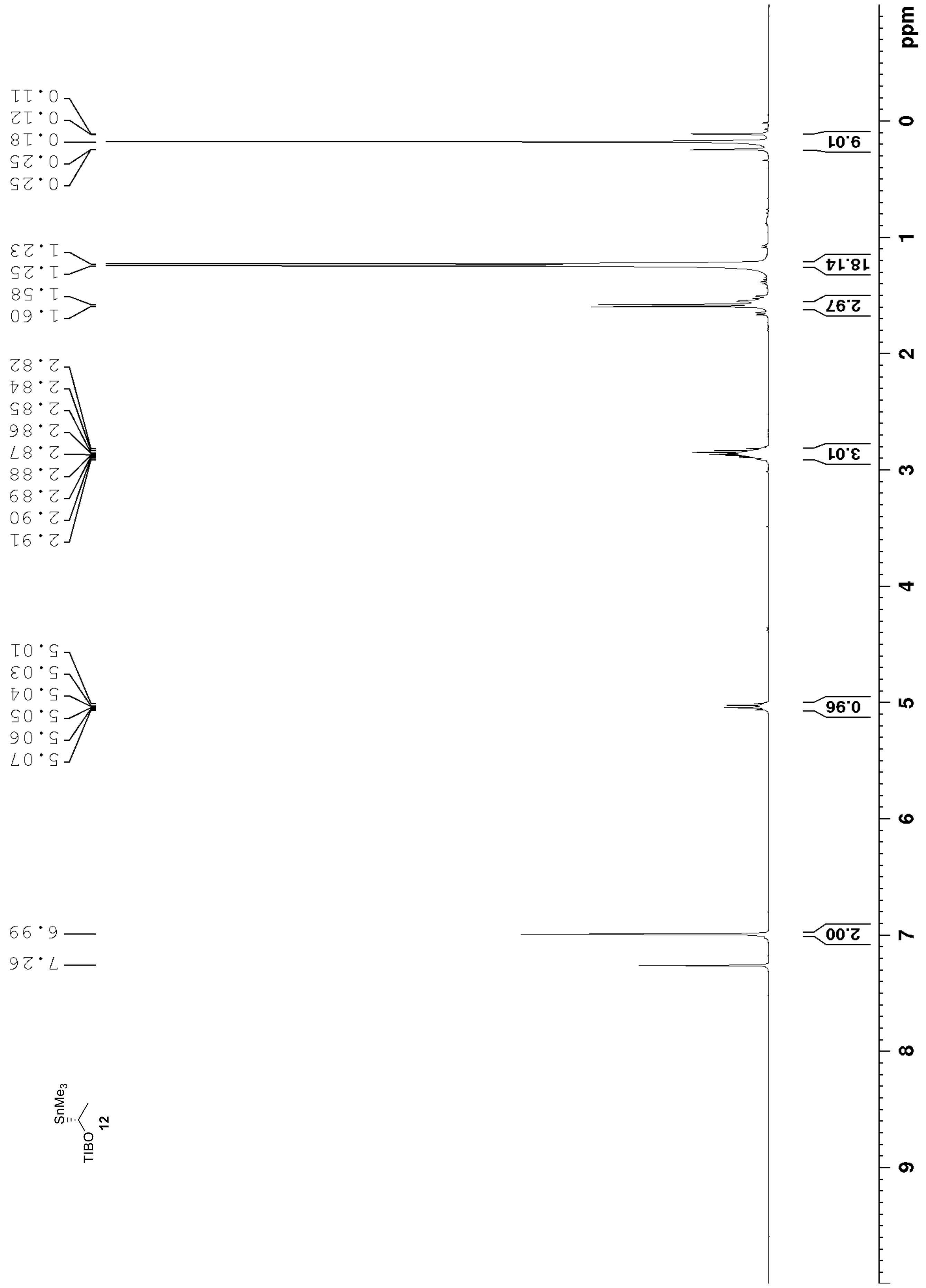
0.11
0.12
0.18
0.25
0.25
1.23
1.25
1.58
1.60
2.82
2.84
2.85
2.86
2.87
2.88
2.89
2.90
2.91
5.01
5.03
5.04
5.05
5.06
5.07
6.99
7.26
9.01
18.14
2.97
3.01
0.96
2.00
ppm
0
1
2
3
4
5
6
7
8
9
TIBO
SnMe3
12

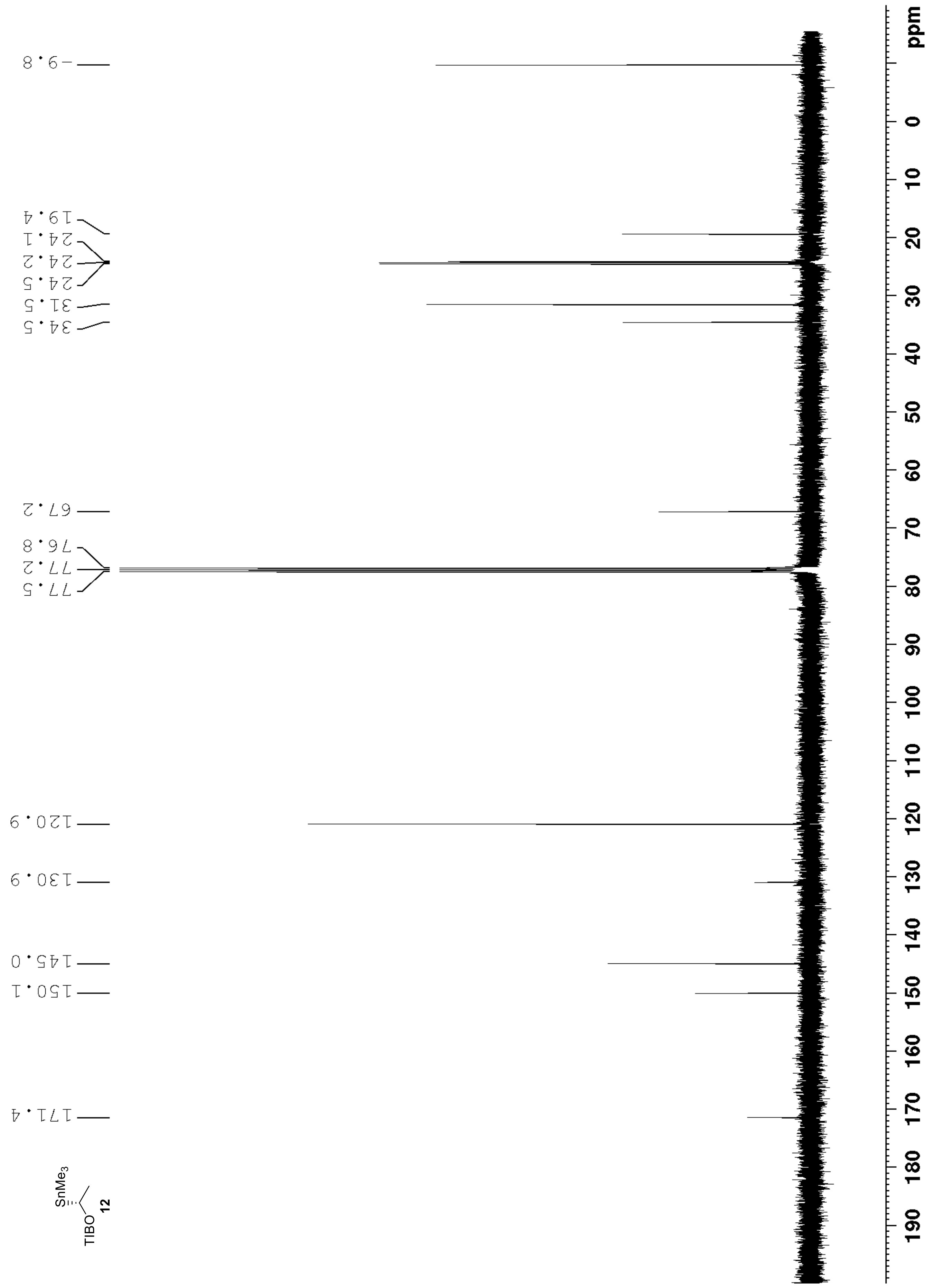
-9.8
19.4
24.1
24.2
24.5
31.5
34.5
67.2
76.8
77.2
77.5
120.9
130.9
145.0
150.1
171.4
TIBO
SnMe3
12
ppm
0
10
20
30
40
50
60
70
80
90
100
110
120
130
140
150
160
170
180
190

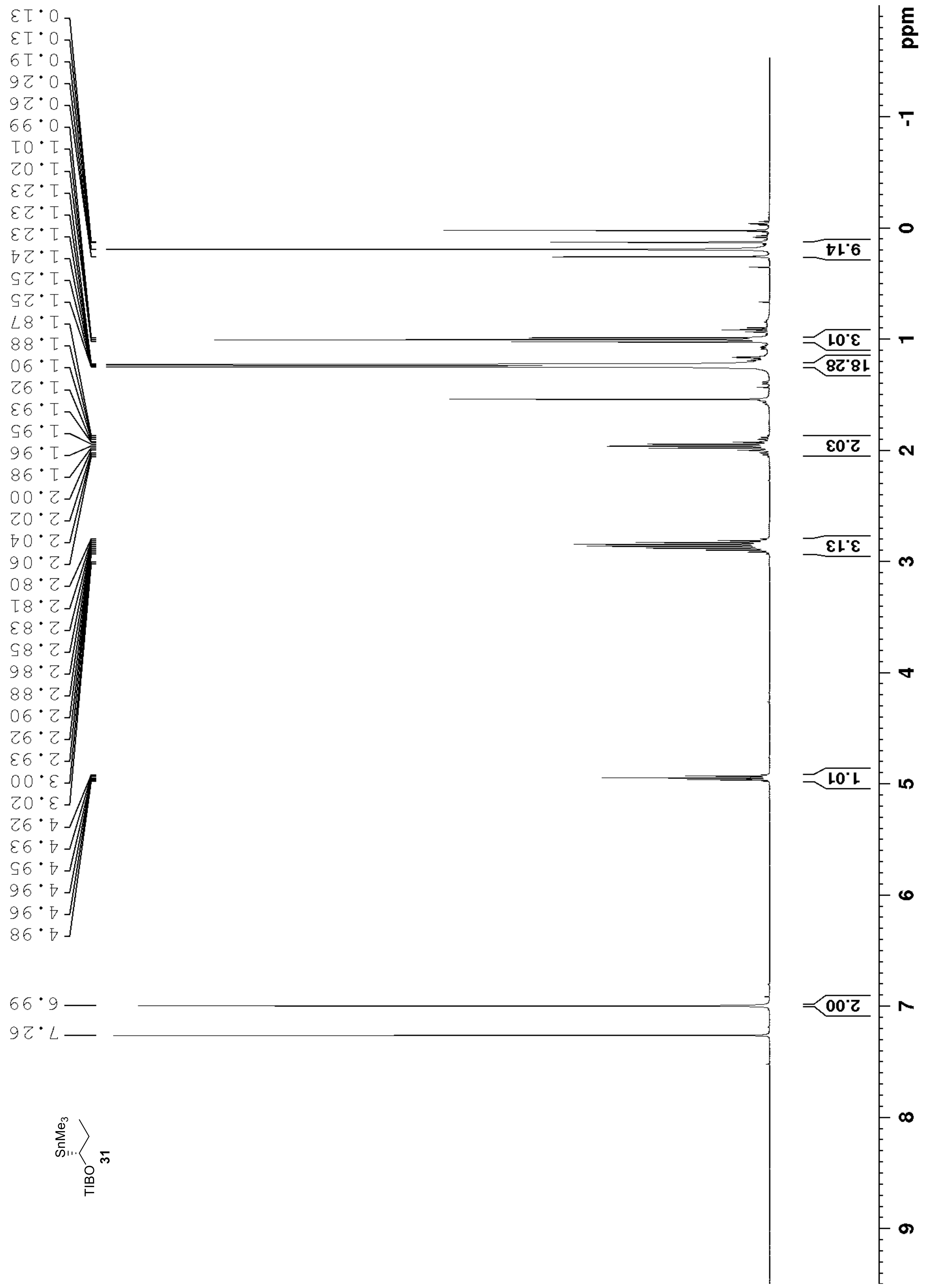
0.13
0.13
0.19
0.26
0.26
0.99
1.01
1.02
1.23
1.23
1.23
1.24
1.25
1.25
1.87
1.88
1.90
1.92
1.93
1.95
1.96
1.98
2.00
2.02
2.04
2.06
2.80
2.81
2.83
2.85
2.86
2.88
2.90
2.92
2.93
3.00
3.02
4.92
4.93
4.95
4.96
4.96
4.98
6.99
7.26
9.14
3.01
18.28
2.03
3.13
1.01
2.00
ppm
-1
0
1
2
3
4
5
6
7
8
9
TIBO
SnMe3
31

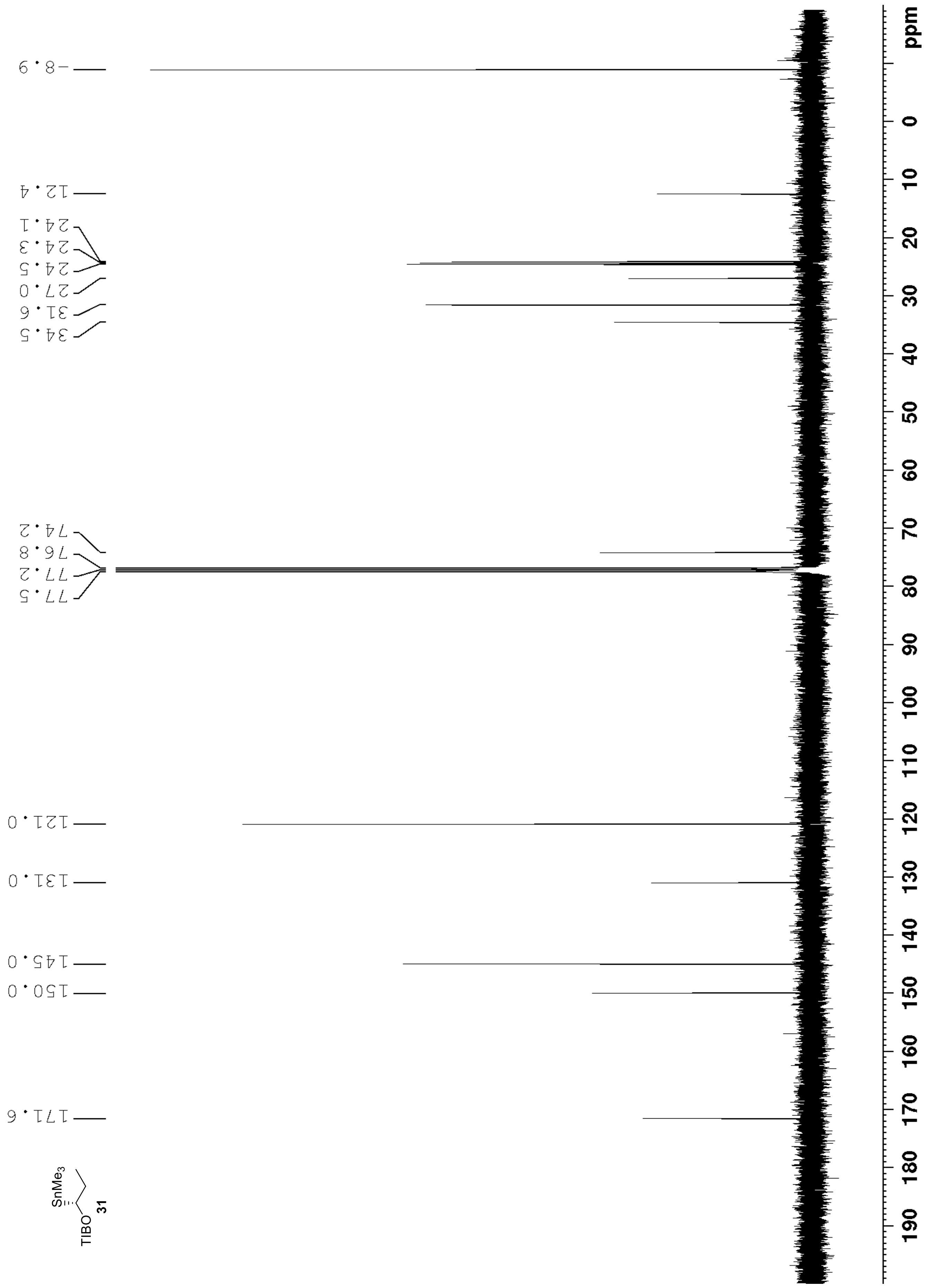
-8.9
12.4
24.1
24.3
24.5
27.0
31.6
34.5
74.2
76.8
77.2
77.5
121.0
131.0
145.0
150.0
171.6
SnMe3
TIBO
31
ppm

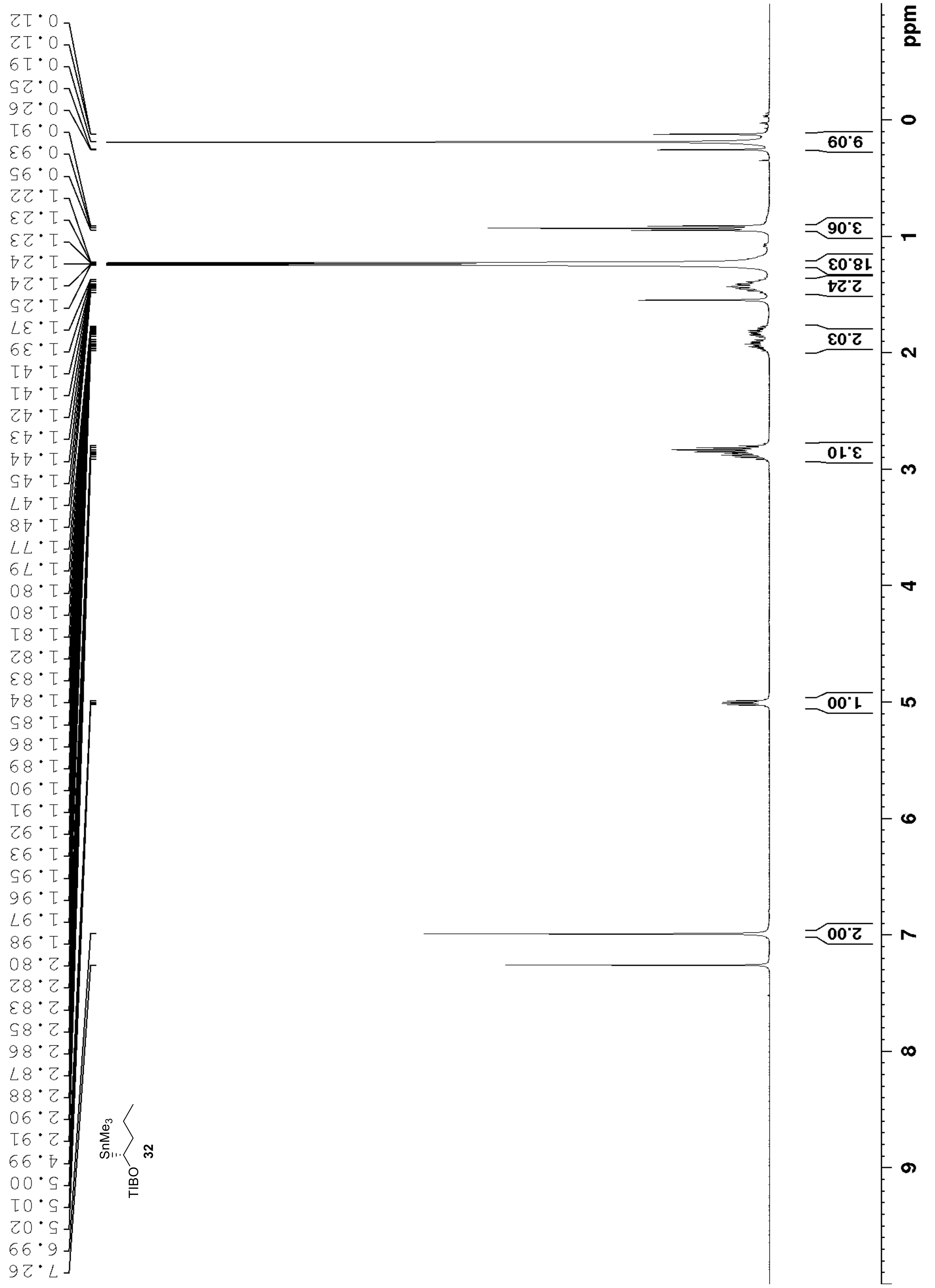
0.12
0.12
0.19
0.25
0.26
0.91
0.93
0.95
1.22
1.23
1.23
1.24
1.24
1.25
1.37
1.39
1.41
1.41
1.42
1.43
1.44
1.45
1.47
1.48
1.77
1.79
1.80
1.80
1.81
1.82
1.83
1.84
1.85
1.86
1.89
1.90
1.91
1.92
1.93
1.95
1.96
1.97
1.98
2.80
2.82
2.83
2.85
2.86
2.87
2.88
2.90
2.91
4.99
5.00
5.01
5.02
6.99
7.26
9.09
3.06
18.03
2.24
2.03
3.10
1.00
2.00
ppm
0
1
2
3
4
5
6
7
8
9
TIBO
SnMe3
32

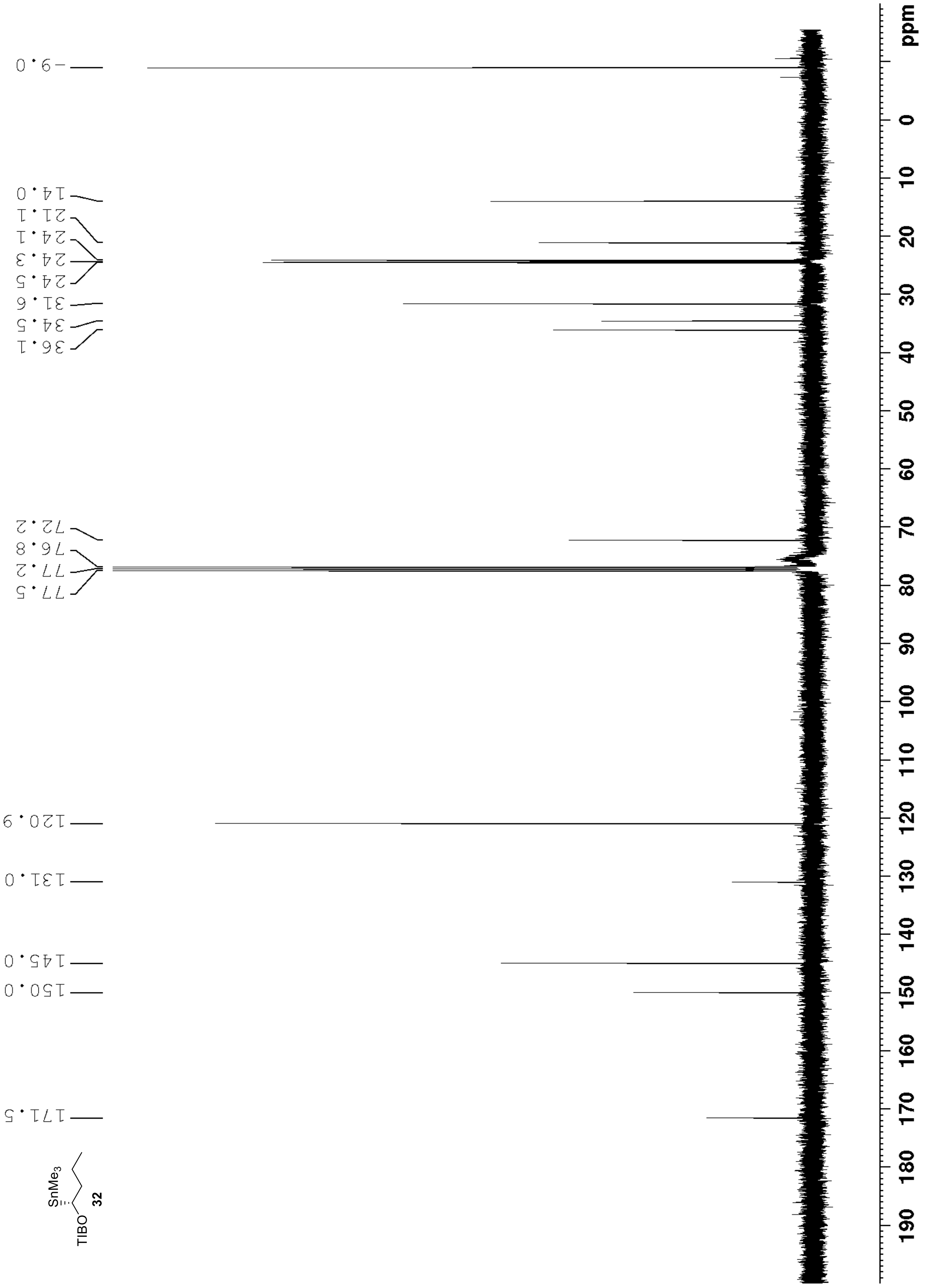
-9.0
14.0
21.1
24.1
24.3
24.5
31.6
34.5
36.1
72.2
76.8
77.2
77.5
120.9
131.0
145.0
150.0
171.5
TIBO
SnMe3
32
ppm
0
10
20
30
40
50
60
70
80
90
100
110
120
130
140
150
160
170
180
190

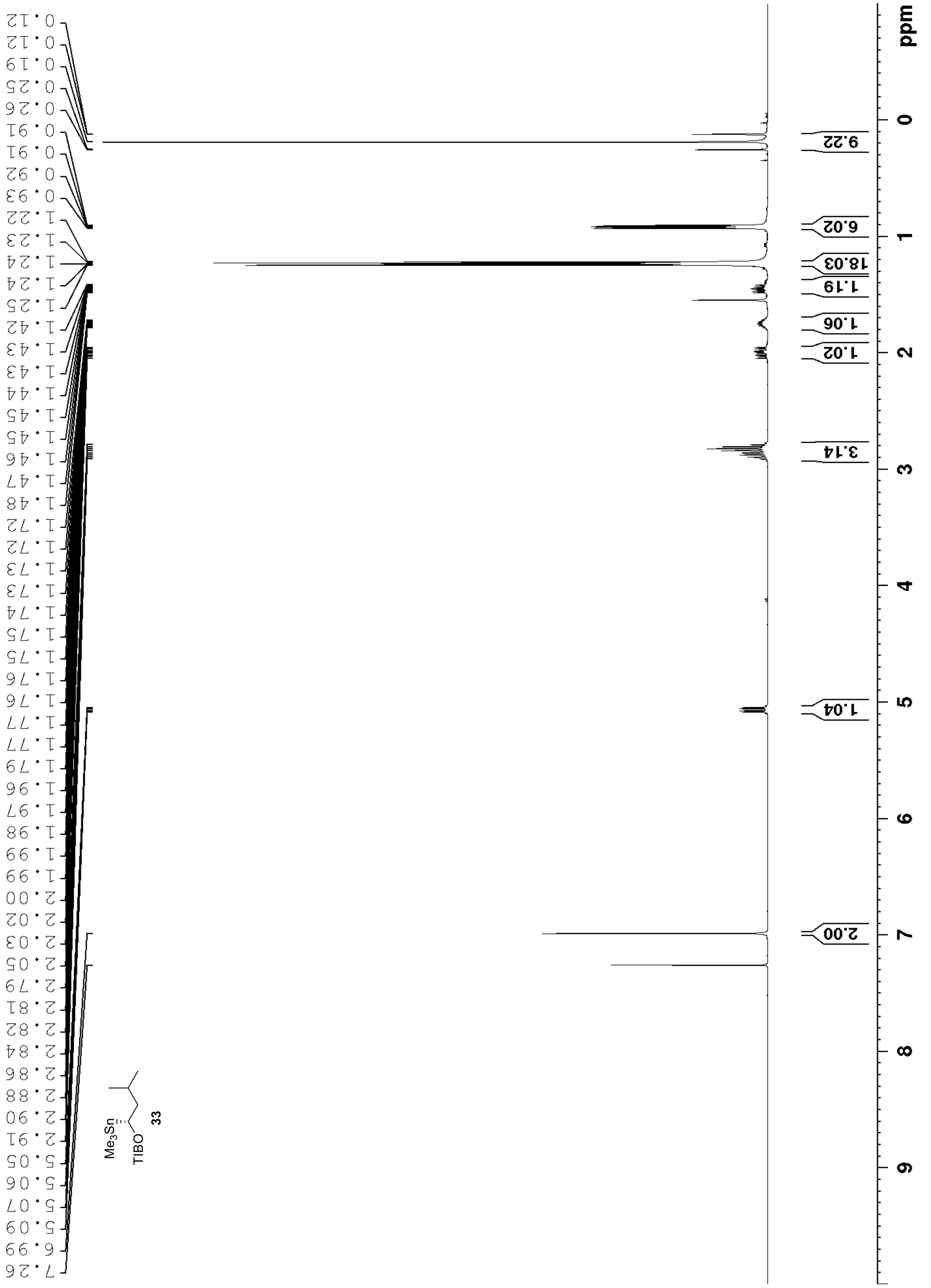
0.12
0.12
0.19
0.25
0.26
0.91
0.91
0.92
0.93
1.22
1.23
1.24
1.24
1.25
1.42
1.43
1.43
1.44
1.45
1.45
1.46
1.47
1.48
1.72
1.72
1.73
1.73
1.74
1.75
1.75
1.76
1.76
1.77
1.77
1.79
1.96
1.97
1.98
1.99
1.99
2.00
2.02
2.03
2.05
2.79
2.81
2.82
2.84
2.86
2.88
2.90
2.91
5.05
5.06
5.07
5.09
6.99
7.26
Me3Sn
TIBO
33
9.22
6.02
18.03
1.19
1.06
1.02
3.14
1.04
2.00
ppm
0
1
2
3
4
5
6
7
8
9

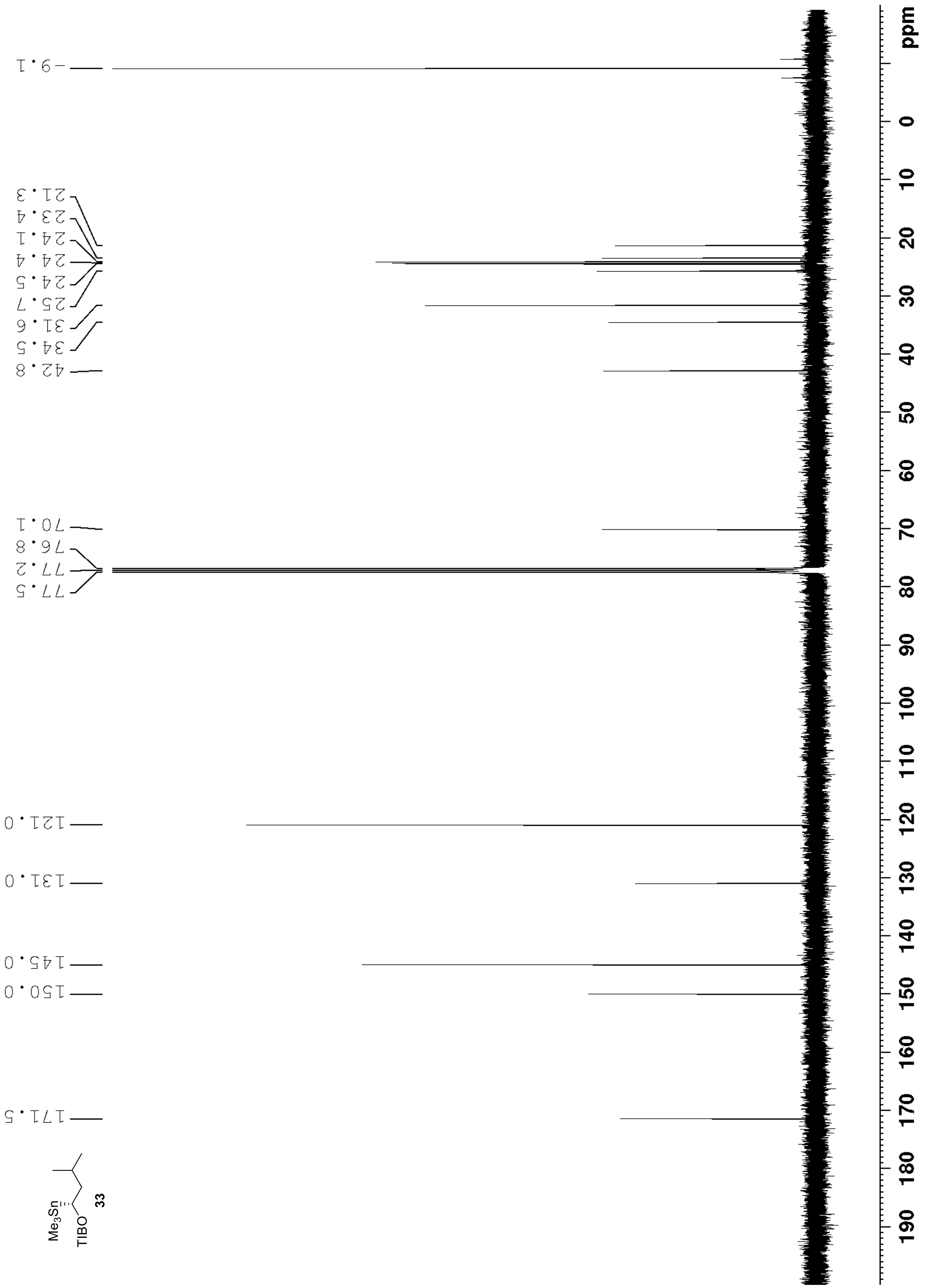
-9.1
21.3
23.4
24.1
24.4
24.5
25.7
31.6
34.5
42.8
70.1
76.8
77.2
77.5
121.0
131.0
145.0
150.0
171.5
Me3Sn
TIBO
33
ppm

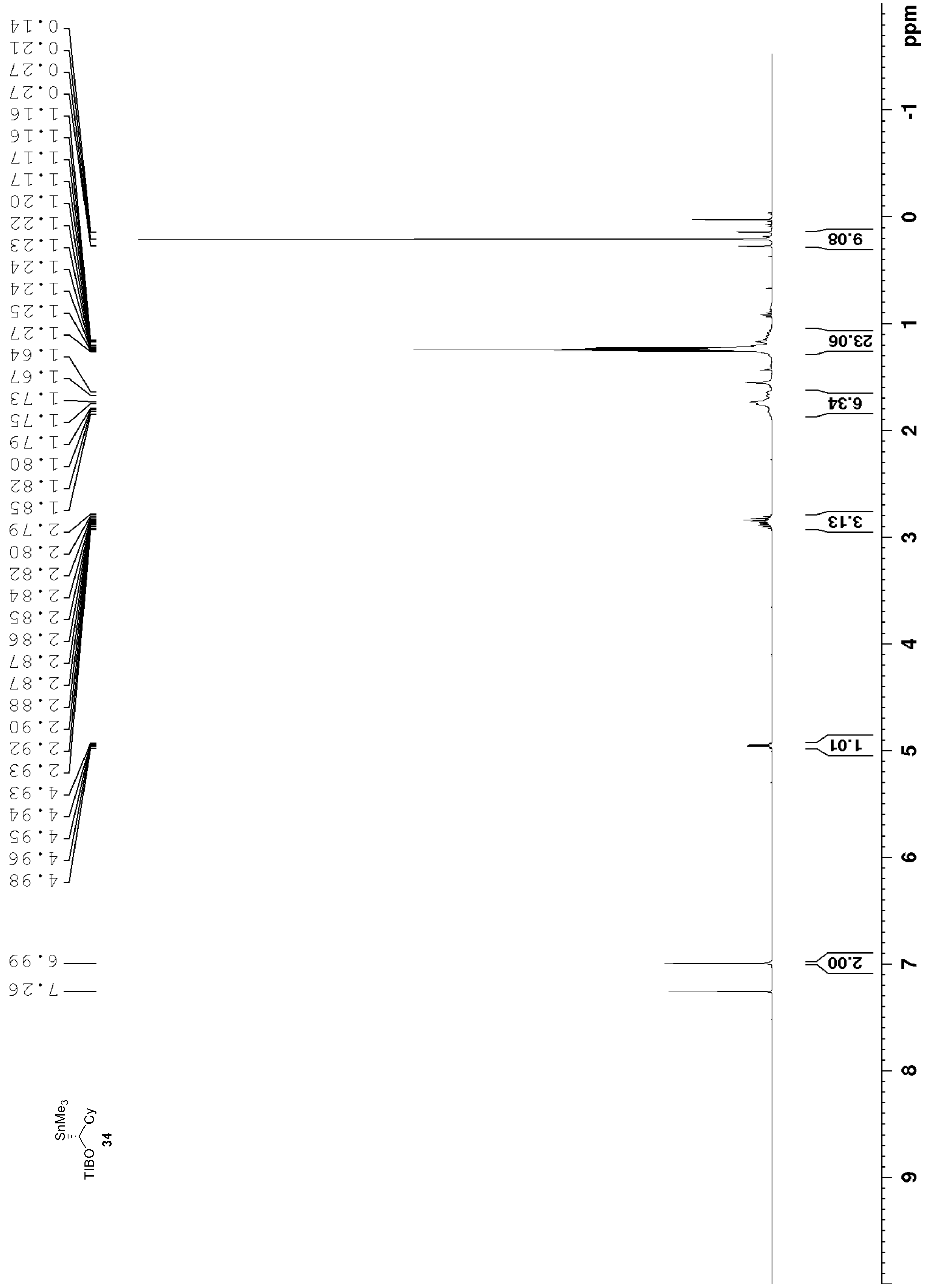

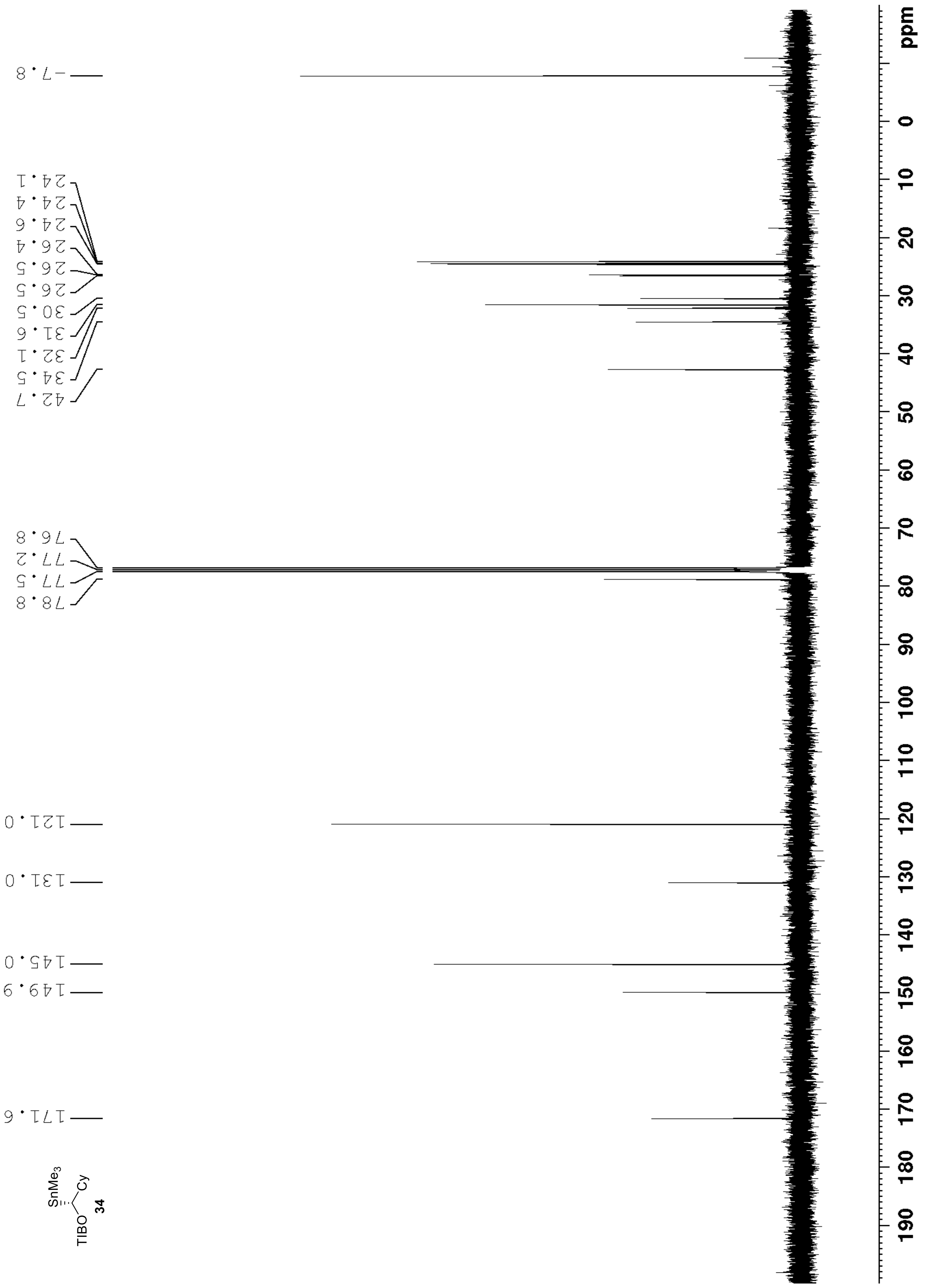
-7.8
24.1
24.4
24.6
26.4
26.5
26.5
30.5
31.6
32.1
34.5
42.7
76.8
77.2
77.5
78.8
121.0
131.0
145.0
149.9
171.6
TIBO
$SnMe_3$
Cy
34
ppm

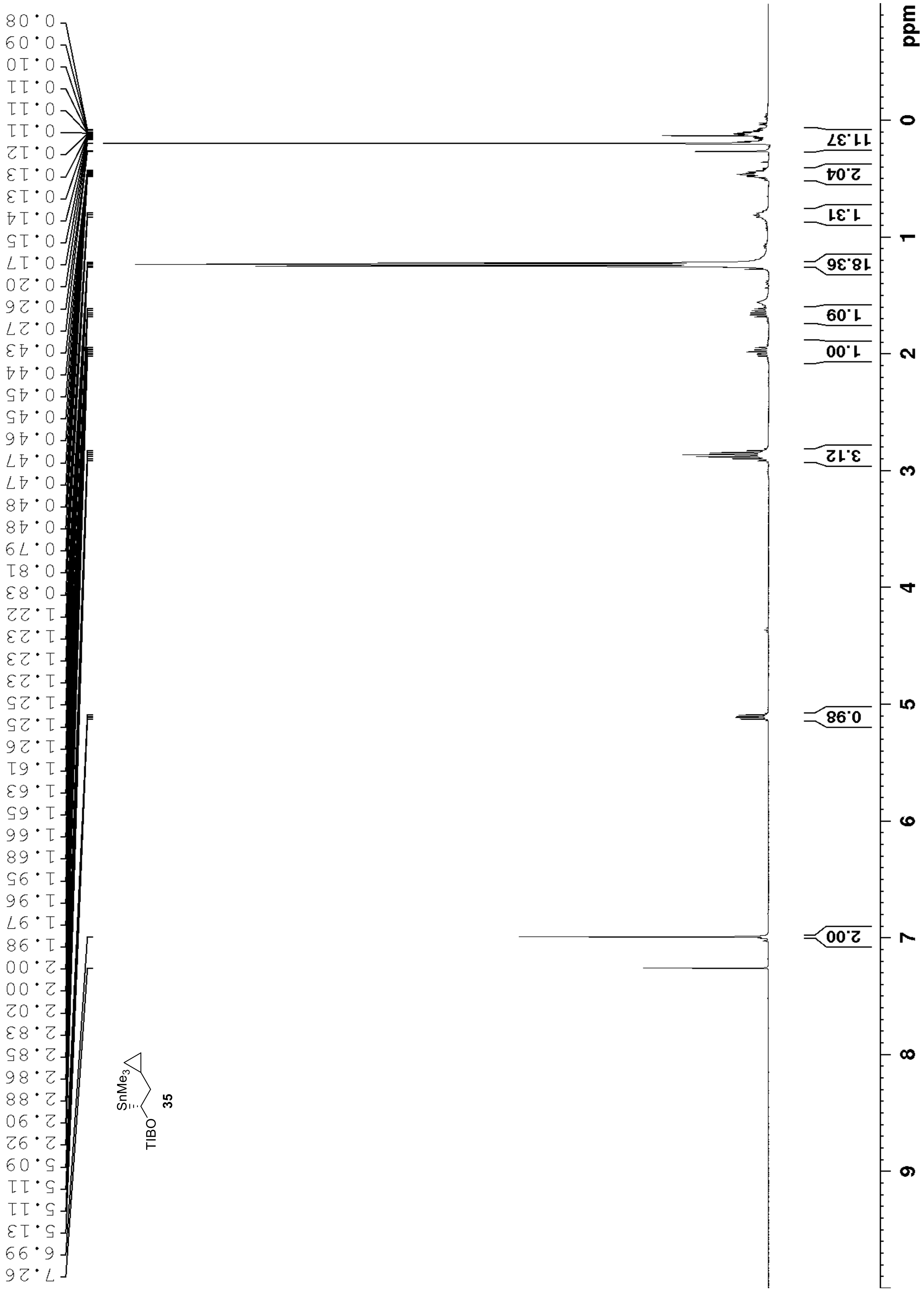
0.08
0.09
0.10
0.11
0.11
0.11
0.12
0.13
0.13
0.14
0.15
0.17
0.20
0.26
0.27
0.43
0.44
0.45
0.45
0.46
0.47
0.47
0.48
0.48
0.79
0.81
0.83
1.22
1.23
1.23
1.23
1.25
1.25
1.26
1.61
1.63
1.65
1.66
1.68
1.95
1.96
1.97
1.98
2.00
2.00
2.02
2.83
2.85
2.86
2.88
2.90
2.92
5.09
5.11
5.11
5.13
6.99
7.26
SnMe3
TIBO
35
11.37
2.04
1.31
18.36
1.09
1.00
3.12
0.98
2.00
ppm

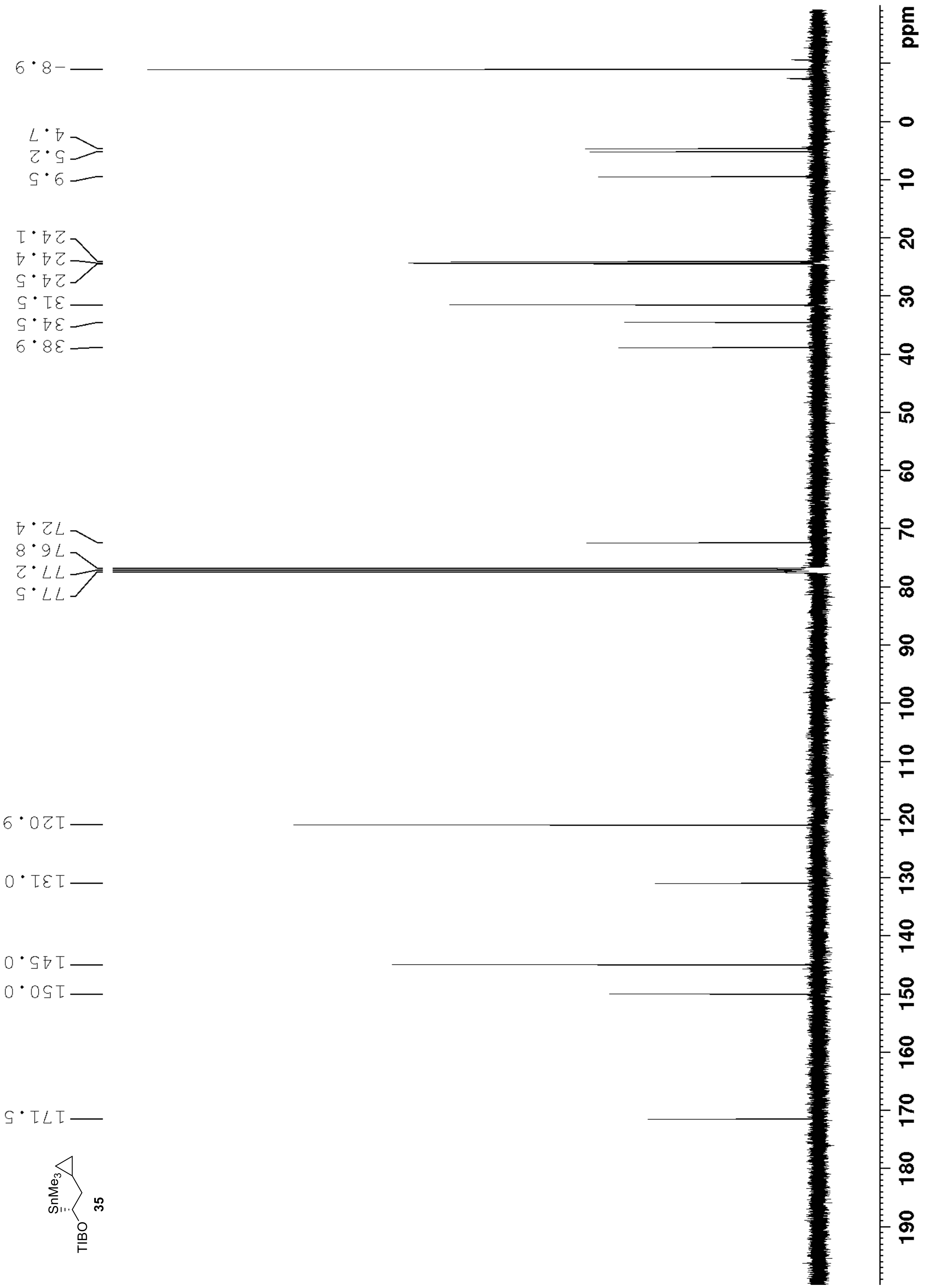
-8.9
4.7
5.2
9.5
24.1
24.4
24.5
31.5
34.5
38.9
72.4
76.8
77.2
77.5
120.9
131.0
145.0
150.0
171.5
ppm
TIBO
SnMe3
35

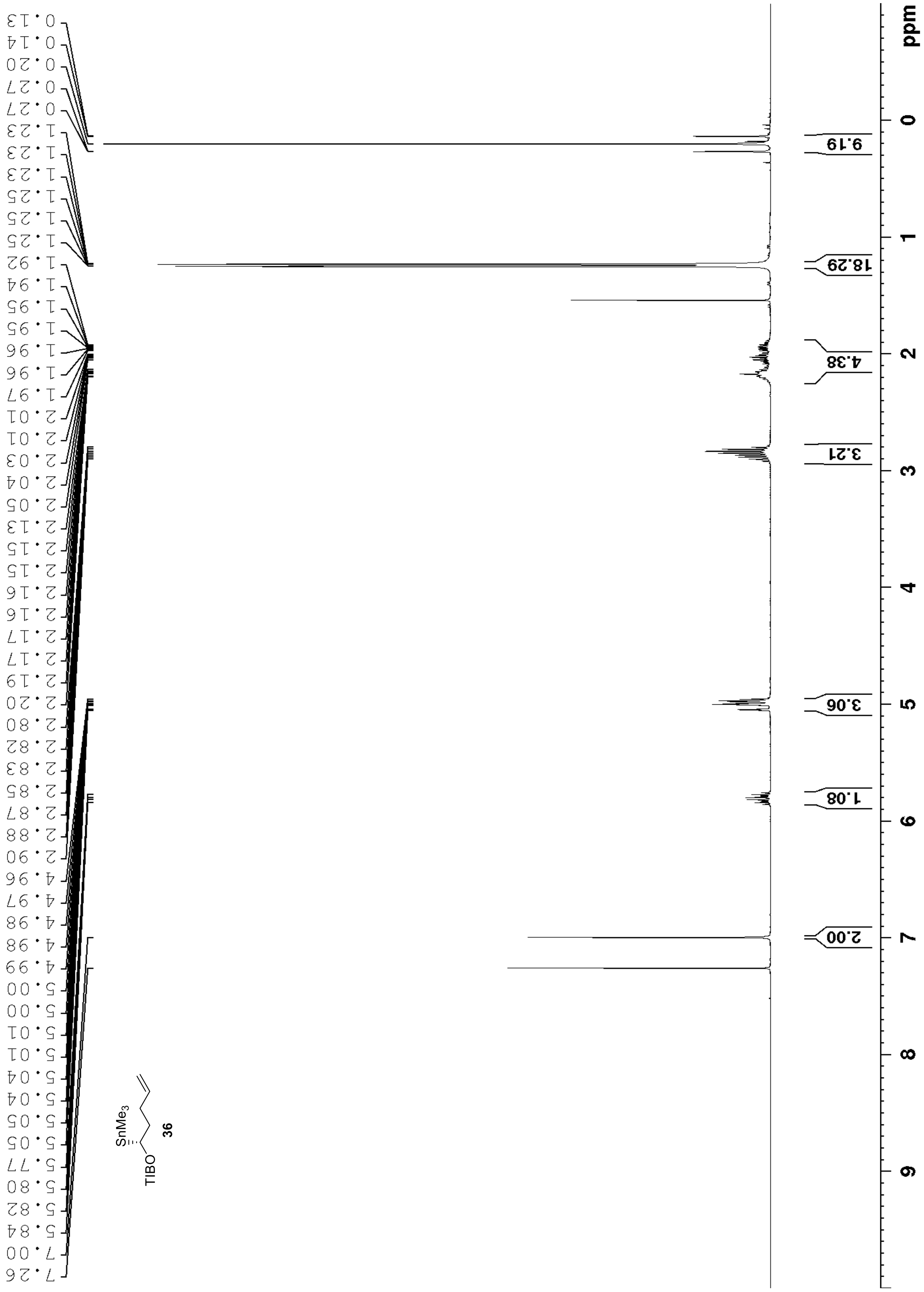
0.13
0.14
0.20
0.27
0.27
1.23
1.23
1.23
1.25
1.25
1.25
1.92
1.94
1.95
1.95
1.96
1.96
1.97
2.01
2.01
2.03
2.04
2.05
2.13
2.15
2.15
2.16
2.16
2.17
2.17
2.19
2.20
2.80
2.82
2.83
2.85
2.87
2.88
2.90
4.96
4.97
4.98
4.98
4.99
5.00
5.00
5.01
5.01
5.04
5.04
5.05
5.05
5.77
5.80
5.82
5.84
7.00
7.26
TIBO
SnMe3
36
9.19
18.29
4.38
3.21
3.06
1.08
2.00
ppm

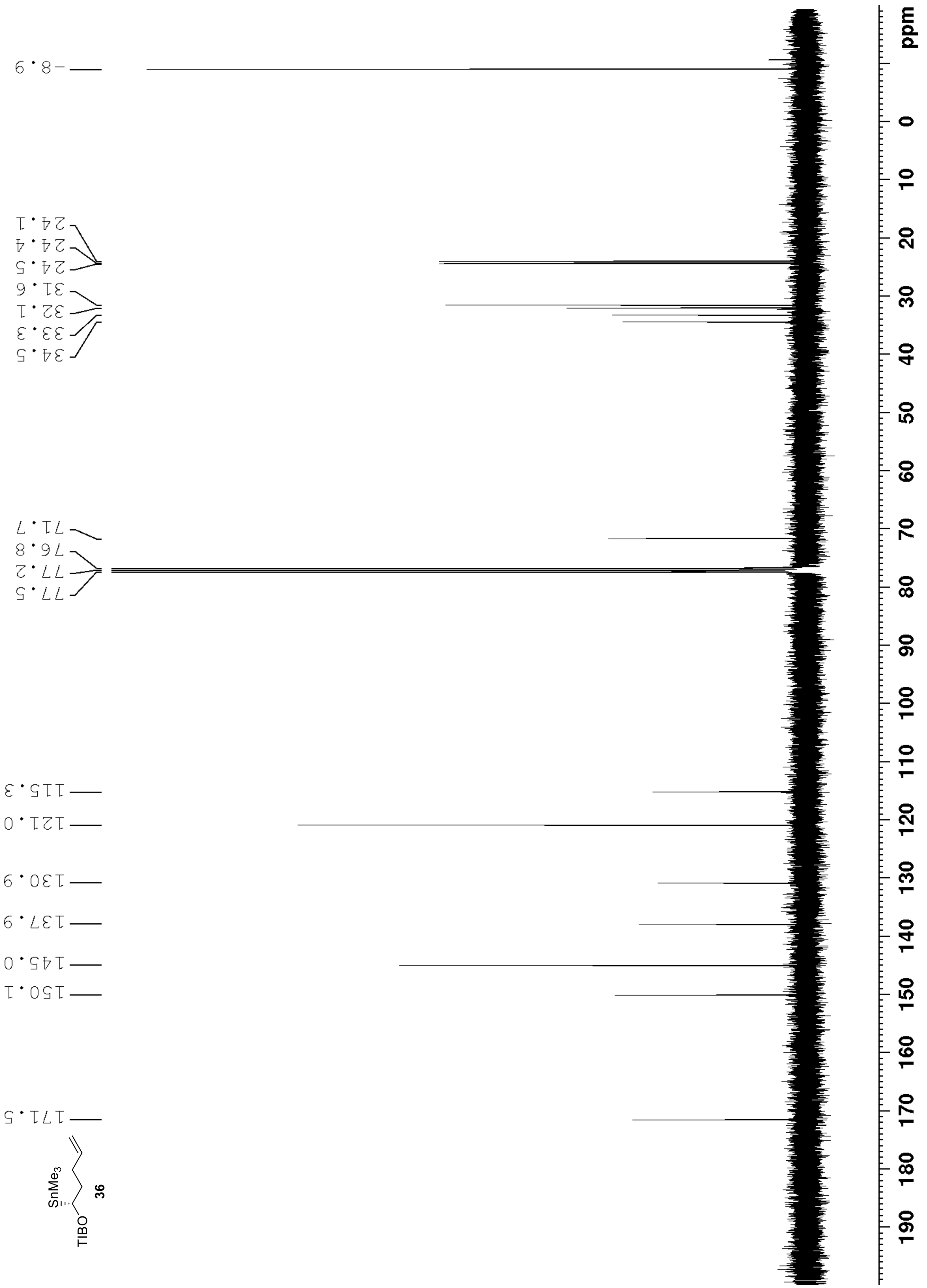
-8.9
24.1
24.4
24.5
31.6
32.1
33.3
34.5
71.7
76.8
77.2
77.5
115.3
121.0
130.9
137.9
145.0
150.1
171.5
SnMe3
TIBO
36
ppm
0
10
20
30
40
50
60
70
80
90
100
110
120
130
140
150
160
170
180
190

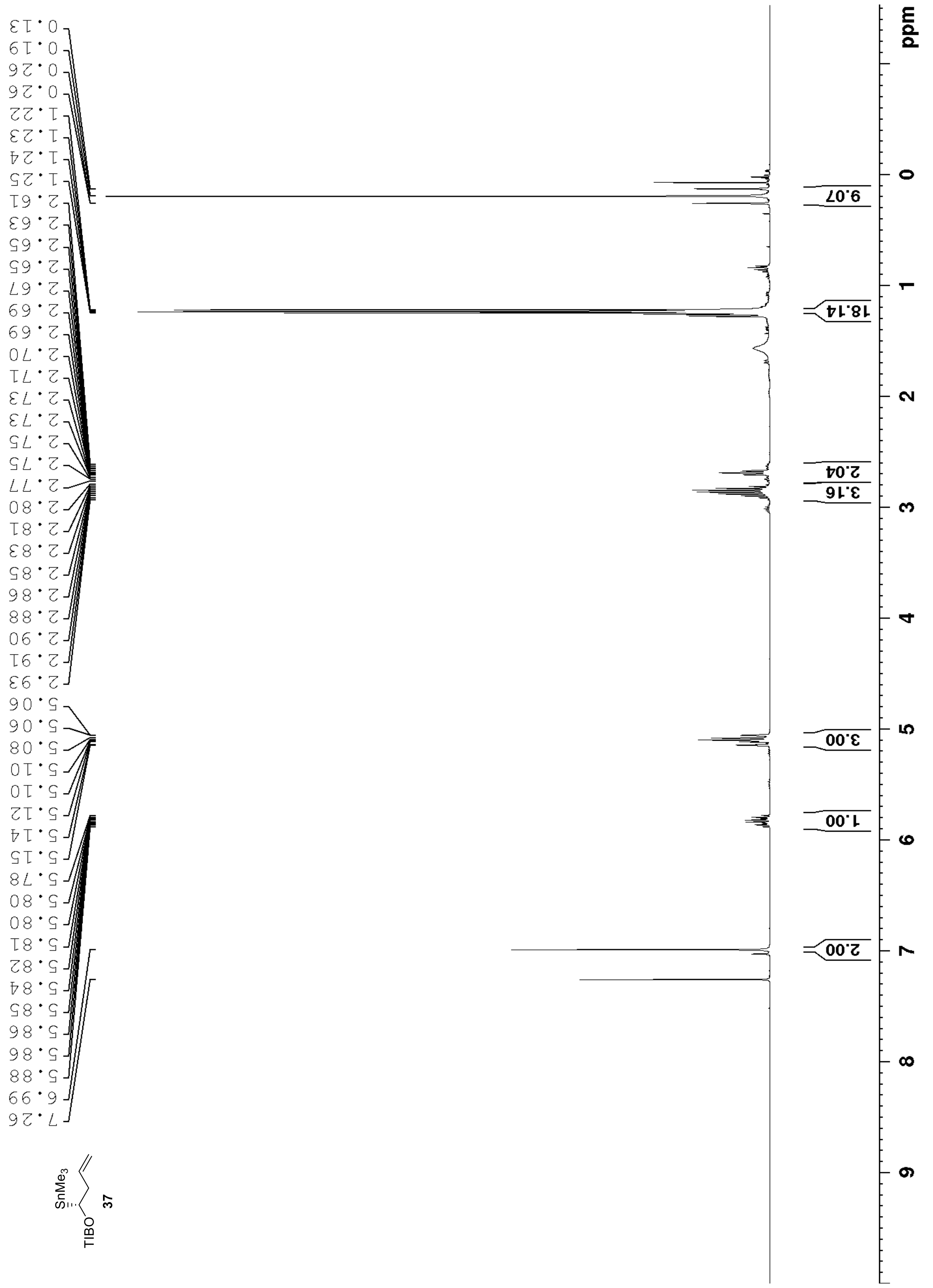
0.13
0.19
0.26
0.26
1.22
1.23
1.24
1.25
2.61
2.63
2.65
2.65
2.67
2.69
2.69
2.70
2.71
2.73
2.73
2.75
2.75
2.77
2.80
2.81
2.83
2.85
2.86
2.88
2.90
2.91
2.93
5.06
5.06
5.08
5.10
5.10
5.12
5.14
5.15
5.78
5.80
5.80
5.81
5.82
5.84
5.85
5.86
5.86
5.88
6.99
7.26
TIBO
SnMe3
37
9.07
18.14
2.04
3.16
3.00
1.00
2.00
ppm
0
1
2
3
4
5
6
7
8
9

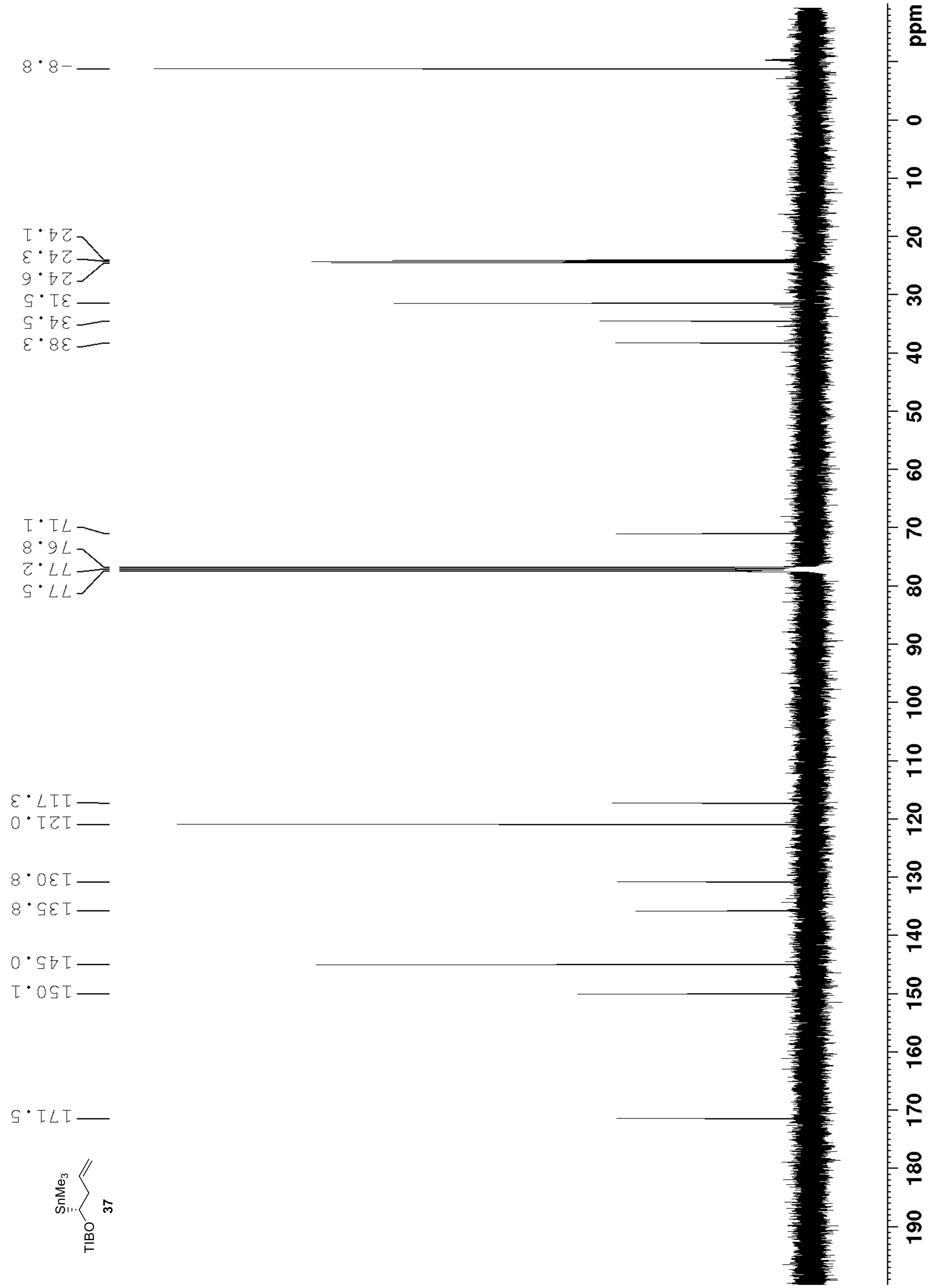
-8.8
24.1
24.3
24.6
31.5
34.5
38.3
71.1
76.8
77.2
77.5
117.3
121.0
130.8
135.8
145.0
150.1
171.5
TIBO
SnMe3
37
ppm

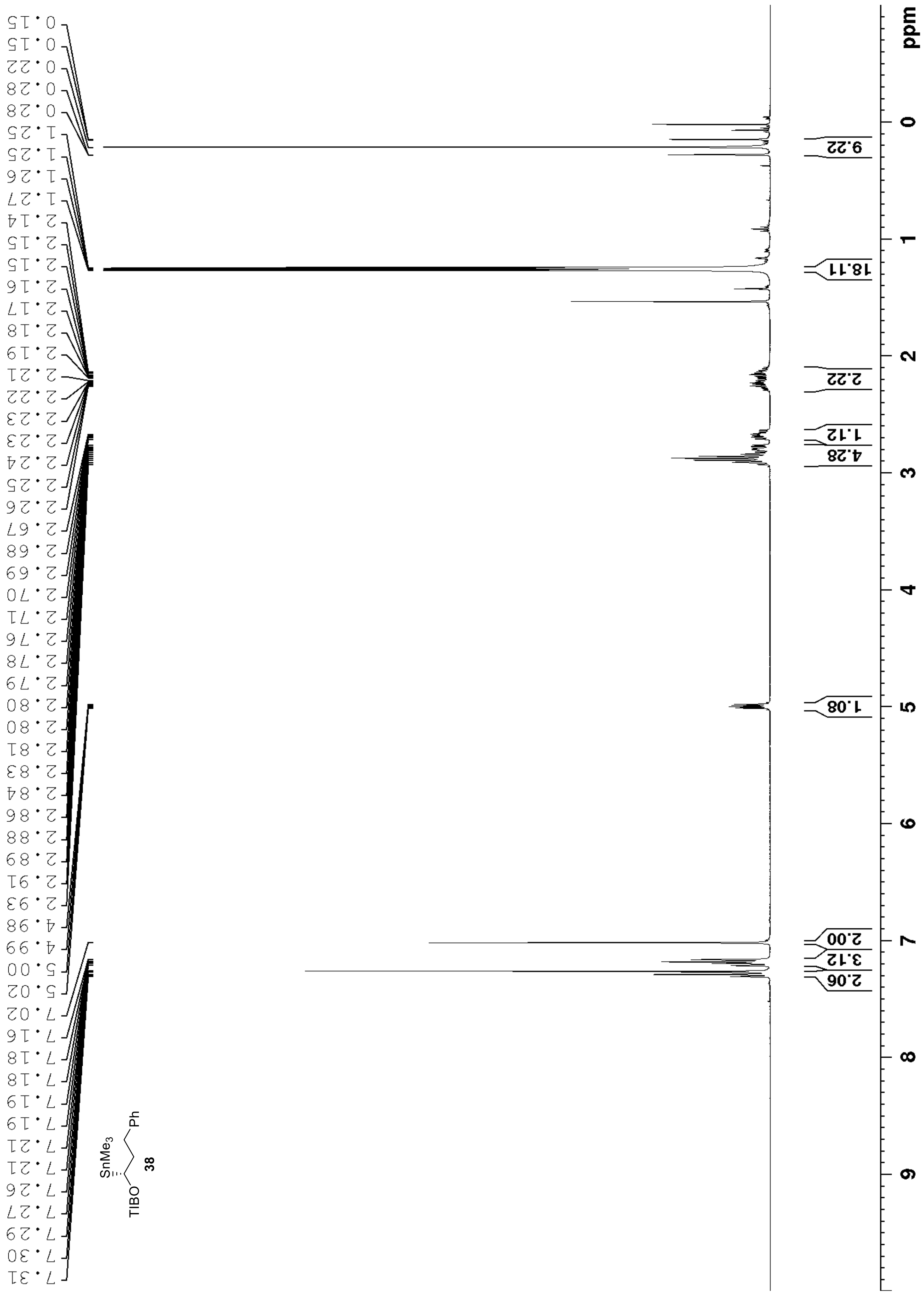
0.15
0.15
0.22
0.28
0.28
1.25
1.25
1.26
1.27
2.14
2.15
2.15
2.16
2.17
2.18
2.19
2.21
2.22
2.23
2.23
2.24
2.25
2.26
2.67
2.68
2.69
2.70
2.71
2.76
2.78
2.79
2.80
2.80
2.81
2.83
2.84
2.86
2.88
2.89
2.91
2.93
4.98
4.99
5.00
5.02
7.02
7.16
7.18
7.18
7.19
7.19
7.21
7.21
7.26
7.27
7.29
7.30
7.31
TIBO
SnMe3
Ph
38
9.22
18.11
2.22
1.12
4.28
1.08
2.00
3.12
2.06
ppm
0
1
2
3
4
5
6
7
8
9

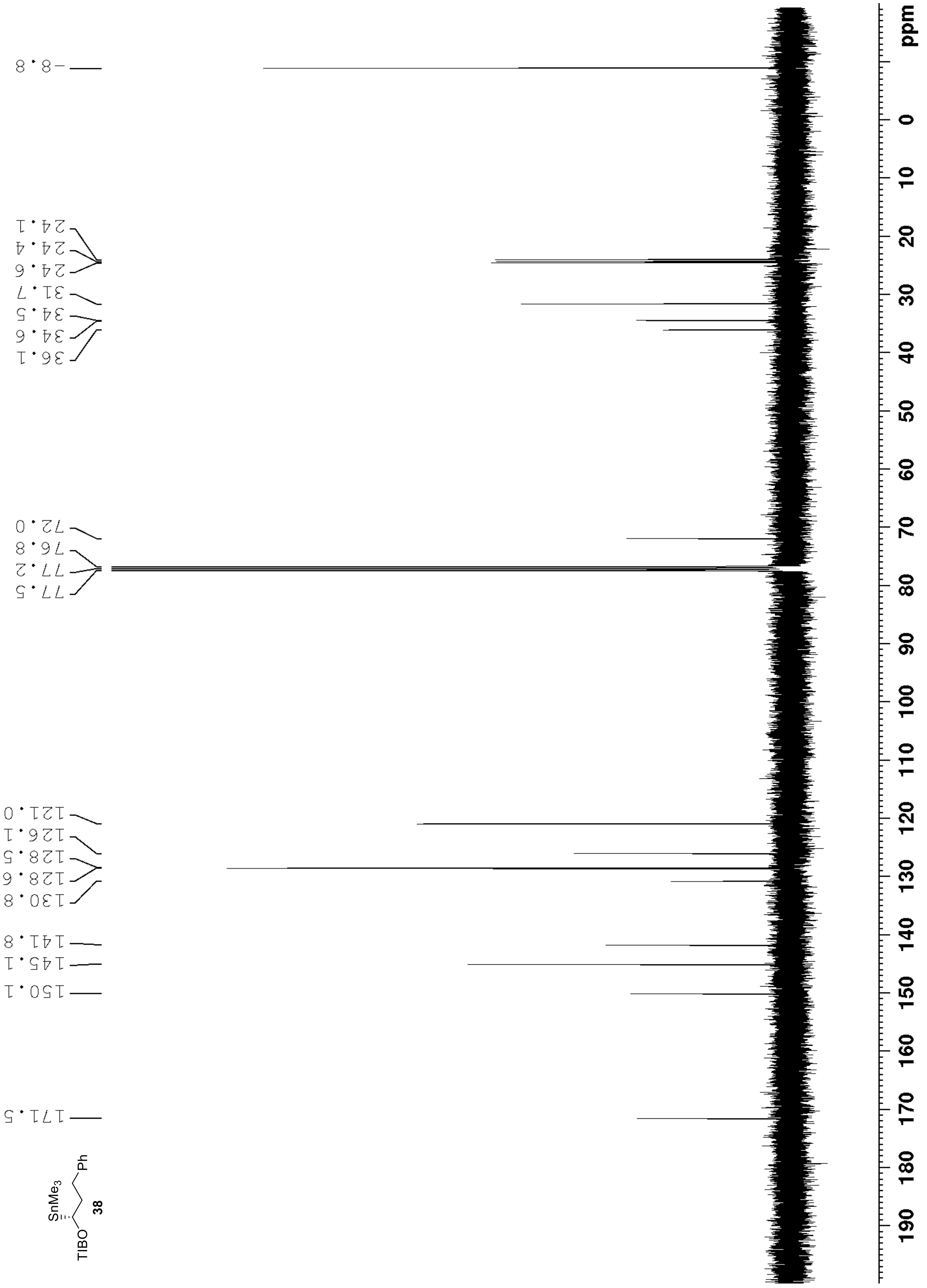
-8.8
24.1
24.4
24.6
31.7
34.5
34.6
36.1
72.0
76.8
77.2
77.5
121.0
126.1
128.5
128.6
130.8
141.8
145.1
150.1
171.5
TIBO
SnMe3
Ph
38
ppm

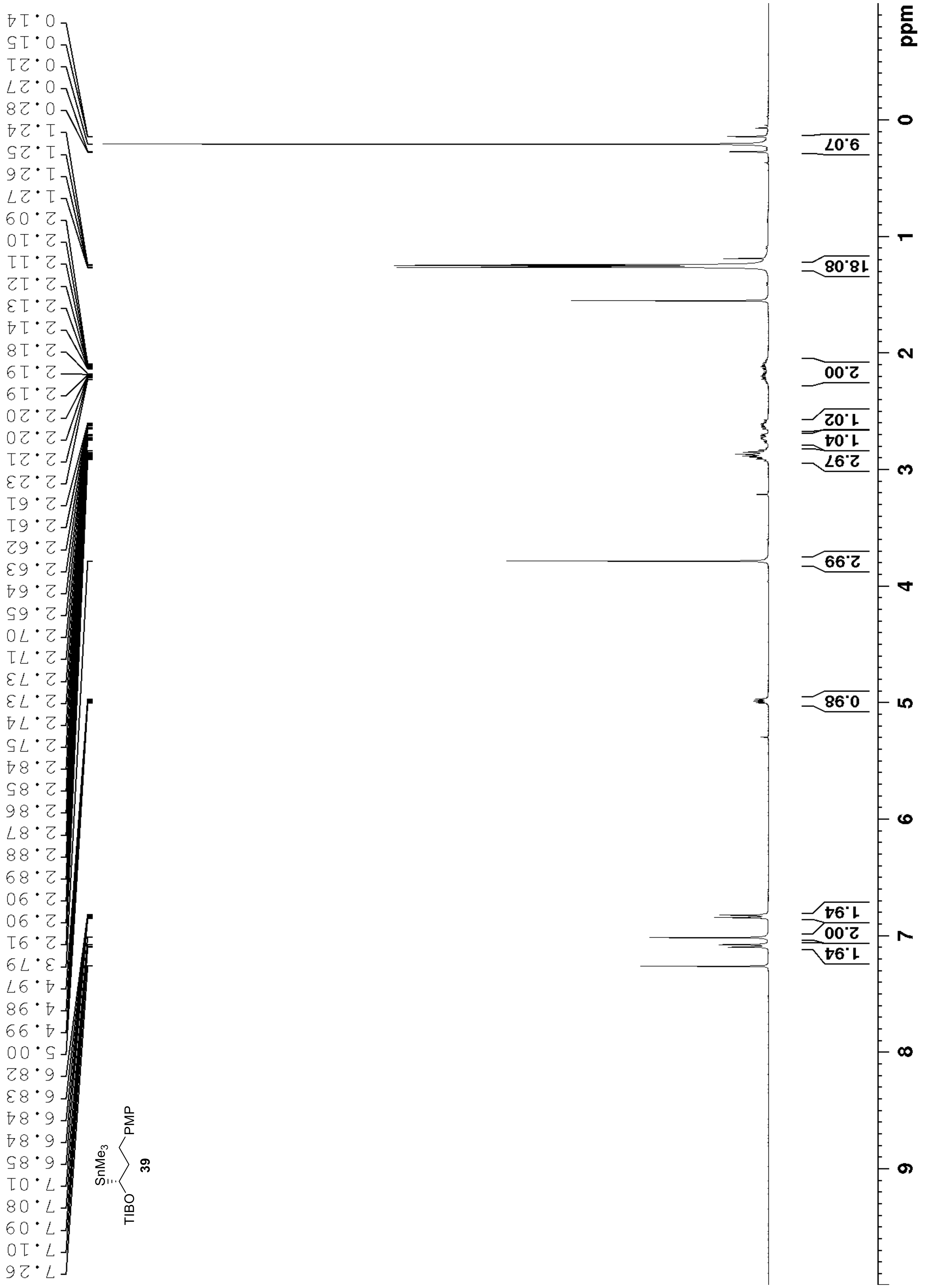
0.14
0.15
0.21
0.27
0.28
1.24
1.25
1.26
1.27
2.09
2.10
2.11
2.12
2.13
2.14
2.18
2.19
2.19
2.20
2.20
2.21
2.23
2.61
2.61
2.62
2.63
2.64
2.65
2.70
2.71
2.73
2.73
2.74
2.75
2.84
2.85
2.86
2.87
2.88
2.89
2.90
2.90
2.91
3.79
4.97
4.98
4.99
5.00
6.82
6.83
6.84
6.84
6.85
7.01
7.08
7.09
7.10
7.26
9.07
18.08
2.00
1.02
1.04
2.97
2.99
0.98
1.94
2.00
1.94
ppm
TIBO
SnMe3
PMP
39

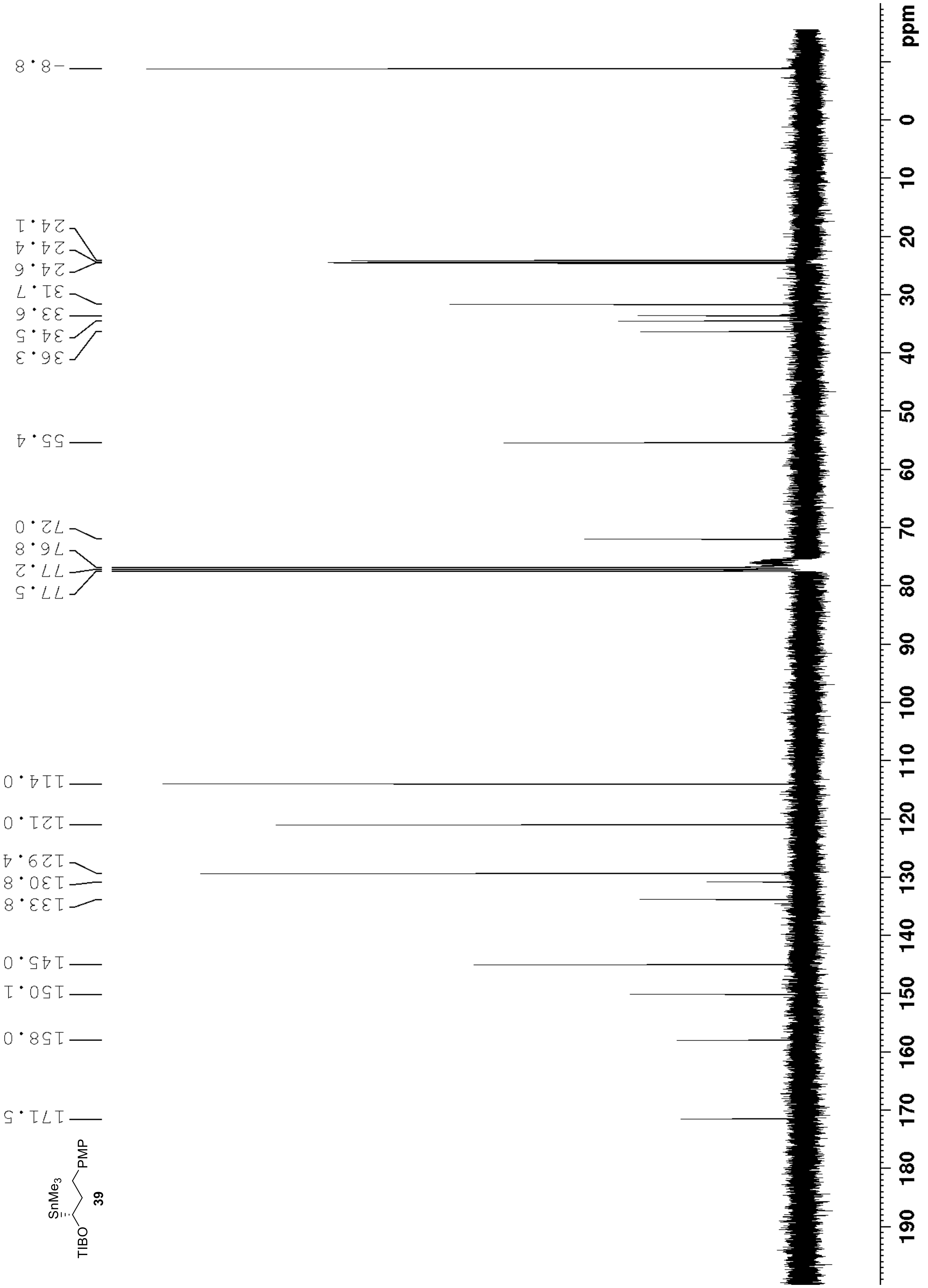
-8.8
24.1
24.4
24.6
31.7
33.6
34.5
36.3
55.4
72.0
76.8
77.2
77.5
114.0
121.0
129.4
130.8
133.8
145.0
150.1
158.0
171.5
TIBO
SnMe3
PMP
39
ppm
0
10
20
30
40
50
60
70
80
90
100
110
120
130
140
150
160
170
180
190

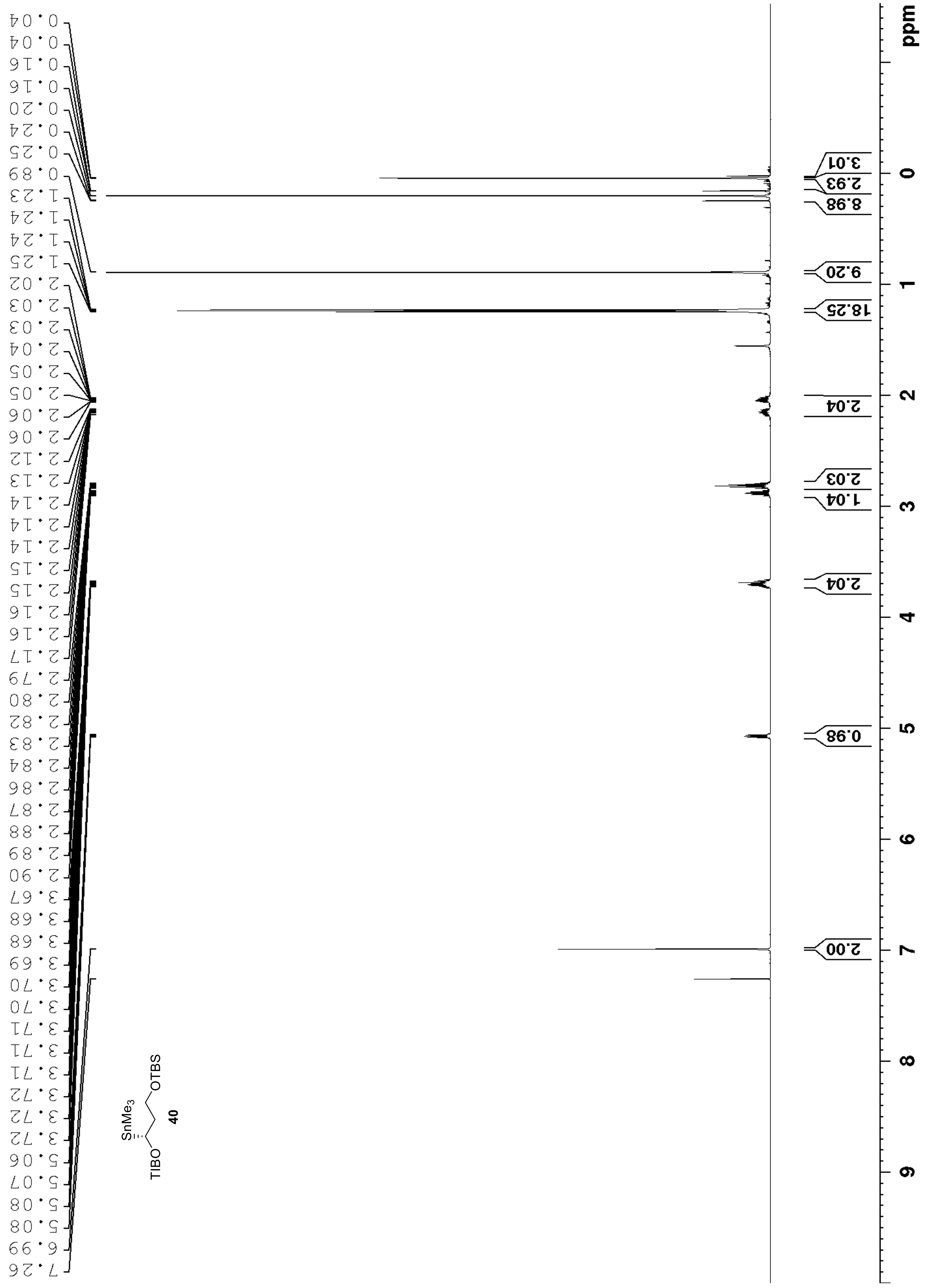
0.04
0.04
0.16
0.16
0.20
0.24
0.25
0.89
1.23
1.24
1.24
1.25
2.02
2.03
2.03
2.04
2.05
2.05
2.06
2.06
2.12
2.13
2.14
2.14
2.14
2.15
2.15
2.16
2.16
2.17
2.79
2.80
2.82
2.83
2.84
2.86
2.87
2.88
2.89
2.90
3.67
3.68
3.68
3.69
3.70
3.70
3.71
3.71
3.71
3.72
3.72
3.72
5.06
5.07
5.08
5.08
6.99
7.26
3.01
2.93
8.98
9.20
18.25
2.04
2.03
1.04
2.04
0.98
2.00
ppm
0
1
2
3
4
5
6
7
8
9
SnMe3
TIBO
OTBS
40

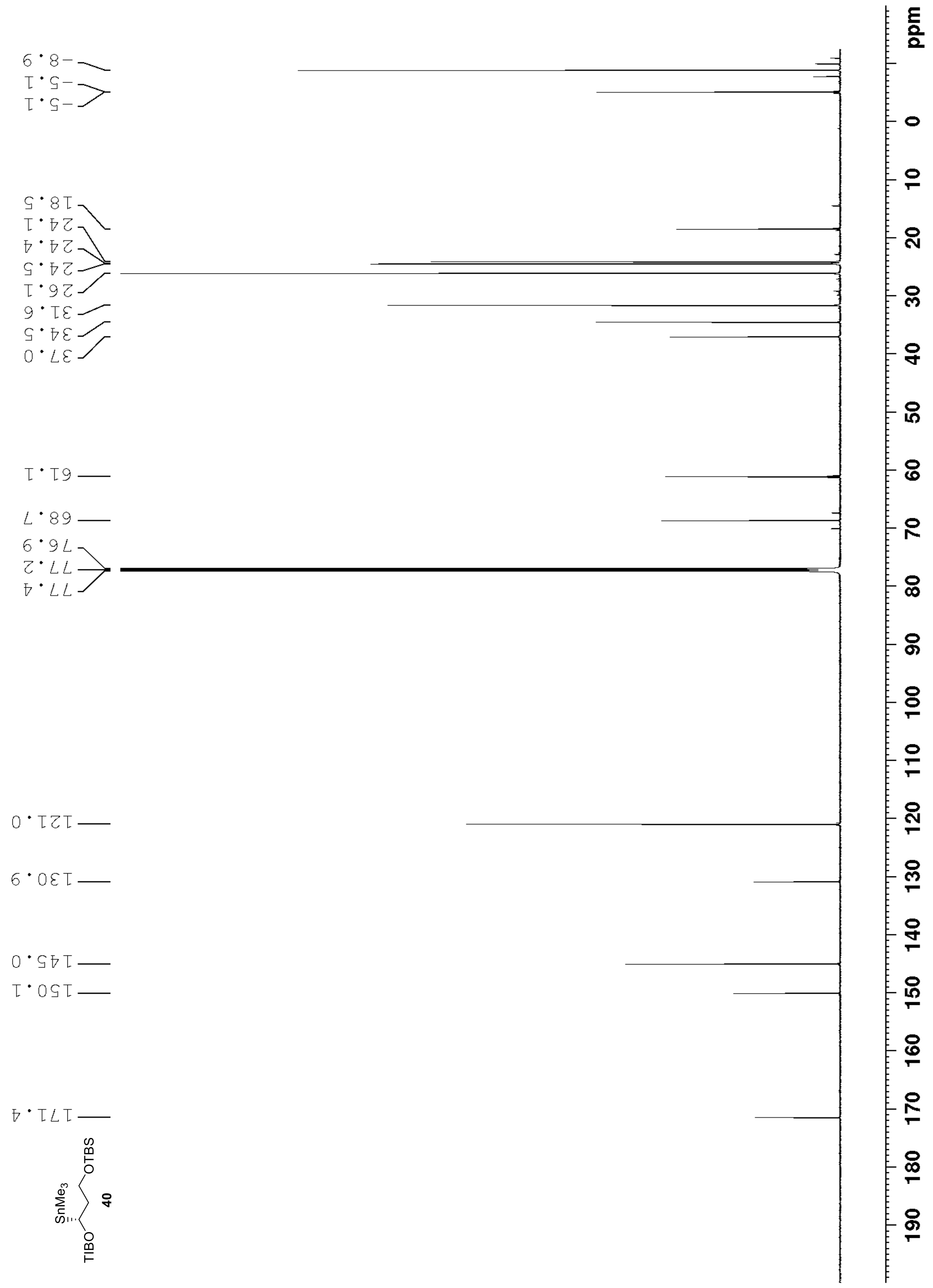
-8.9
-5.1
-5.1
18.5
24.1
24.4
24.5
26.1
31.6
34.5
37.0
61.1
68.7
76.9
77.2
77.4
121.0
130.9
145.0
150.1
171.4
SnMe3
TIBO
OTBS
40
ppm
0
10
20
30
40
50
60
70
80
90
100
110
120
130
140
150
160
170
180
190

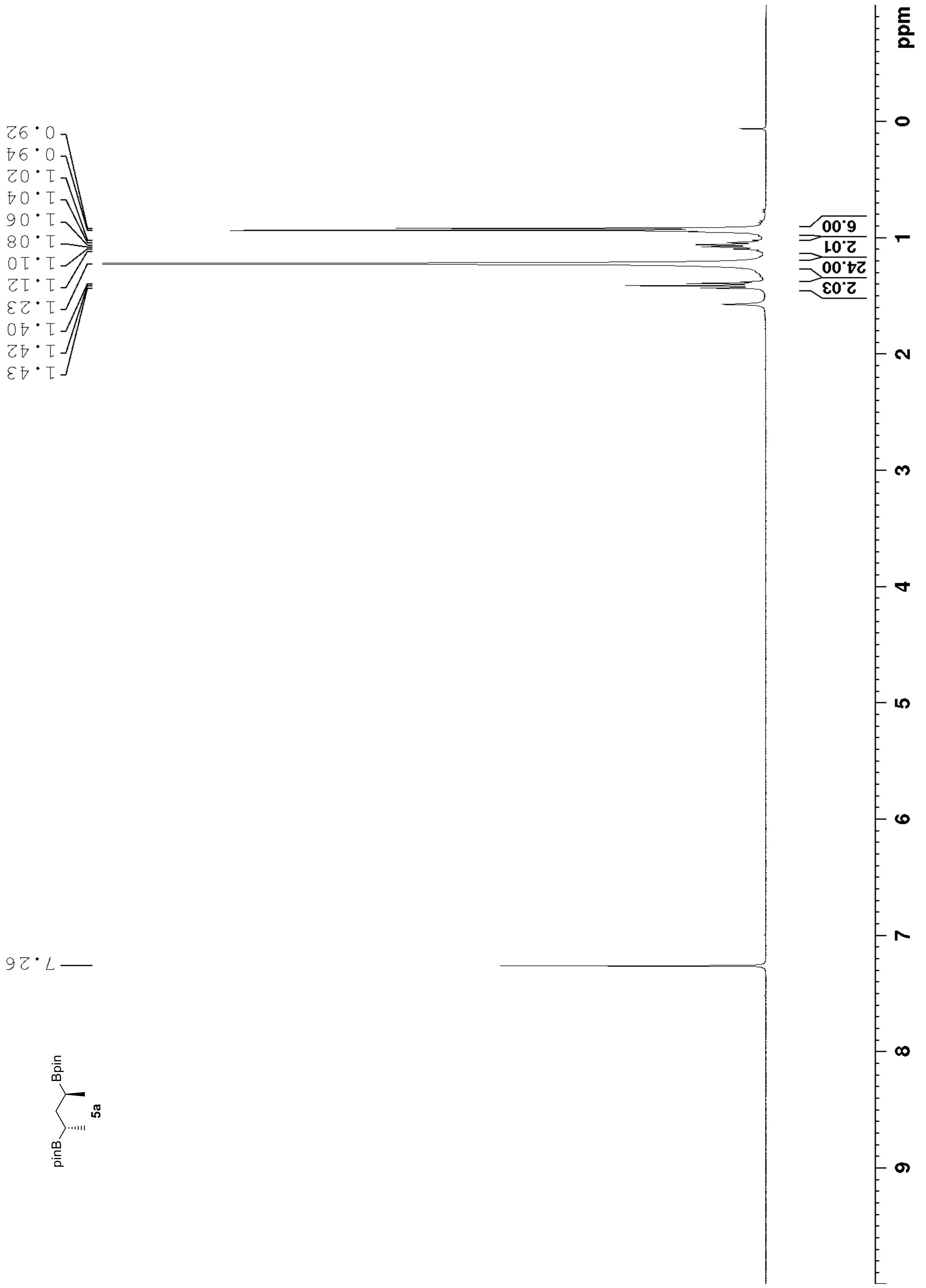
0.92
0.94
1.02
1.04
1.06
1.08
1.10
1.12
1.23
1.40
1.42
1.43
7.26
6.00
2.01
24.00
2.03
ppm
pinB
Bpin
5a

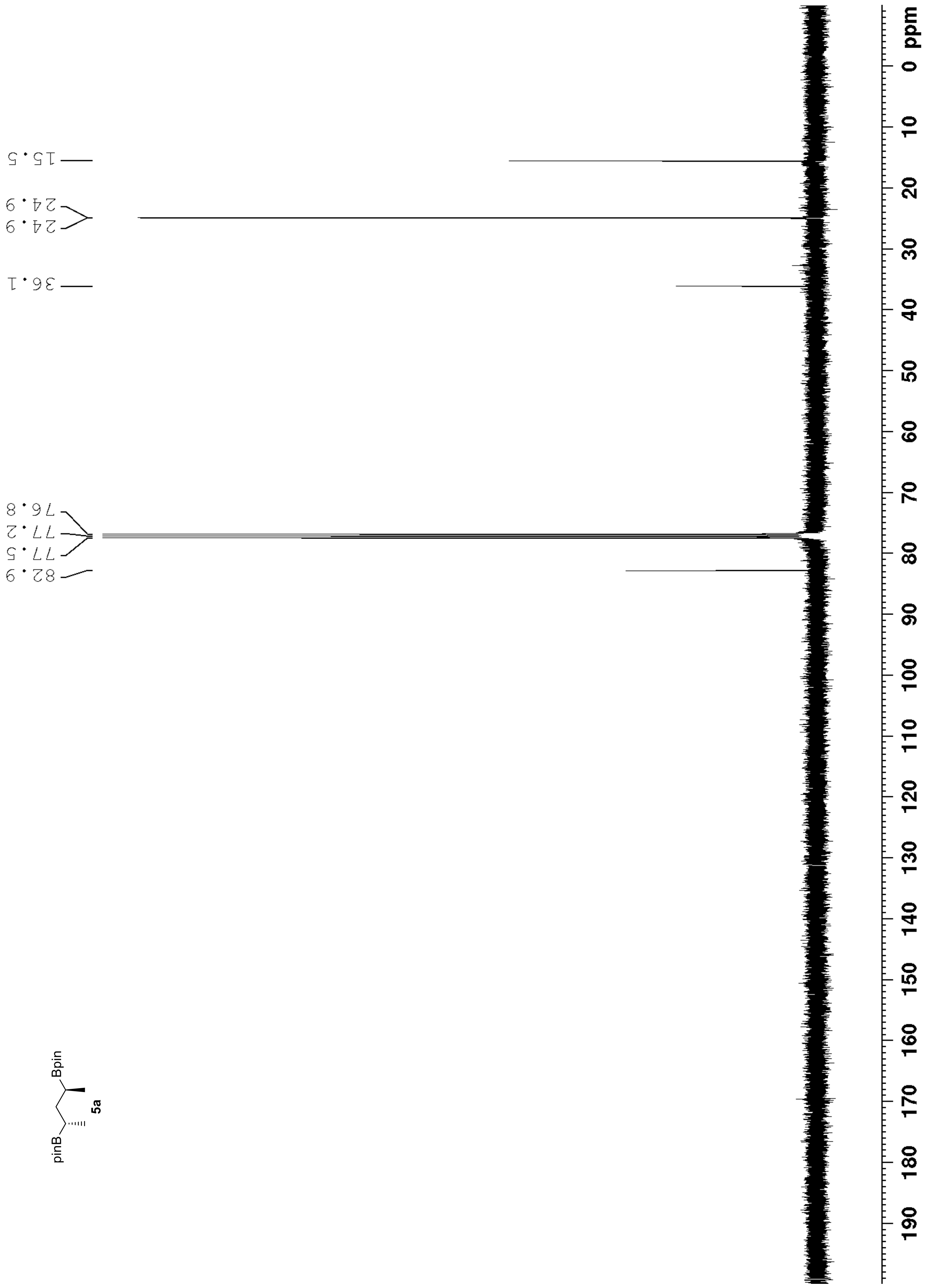
15.5
24.9
24.9
36.1
76.8
77.2
77.5
82.9
pinB
Bpin
5a
ppm
0
10
20
30
40
50
60
70
80
90
100
110
120
130
140
150
160
170
180
190

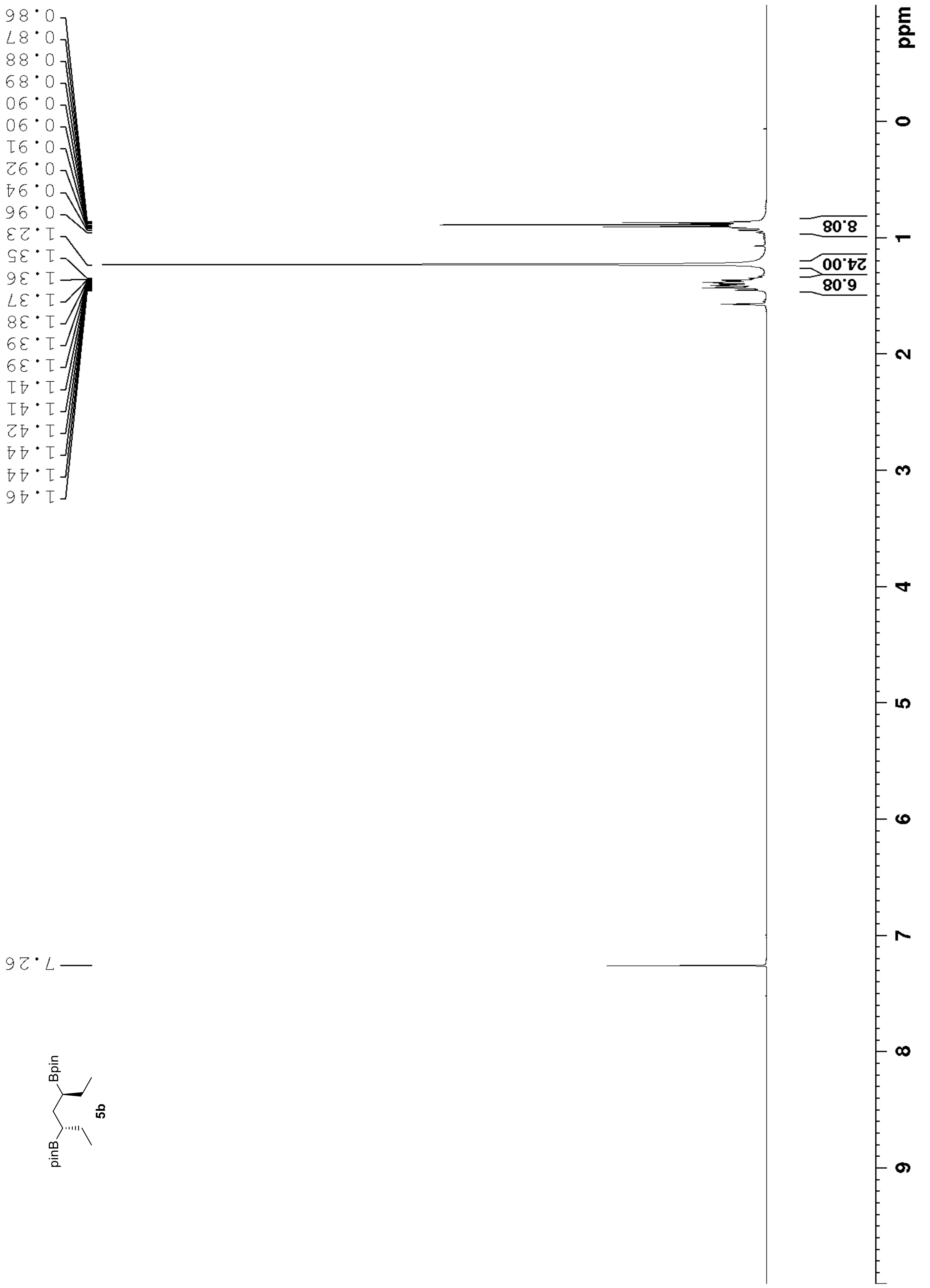
0.86
0.87
0.88
0.89
0.90
0.90
0.91
0.92
0.94
0.96
1.23
1.35
1.36
1.37
1.38
1.39
1.39
1.41
1.41
1.42
1.44
1.44
1.46
7.26
8.08
24.00
6.08
ppm
0
1
2
3
4
5
6
7
8
9
Bpin
pinB
5b

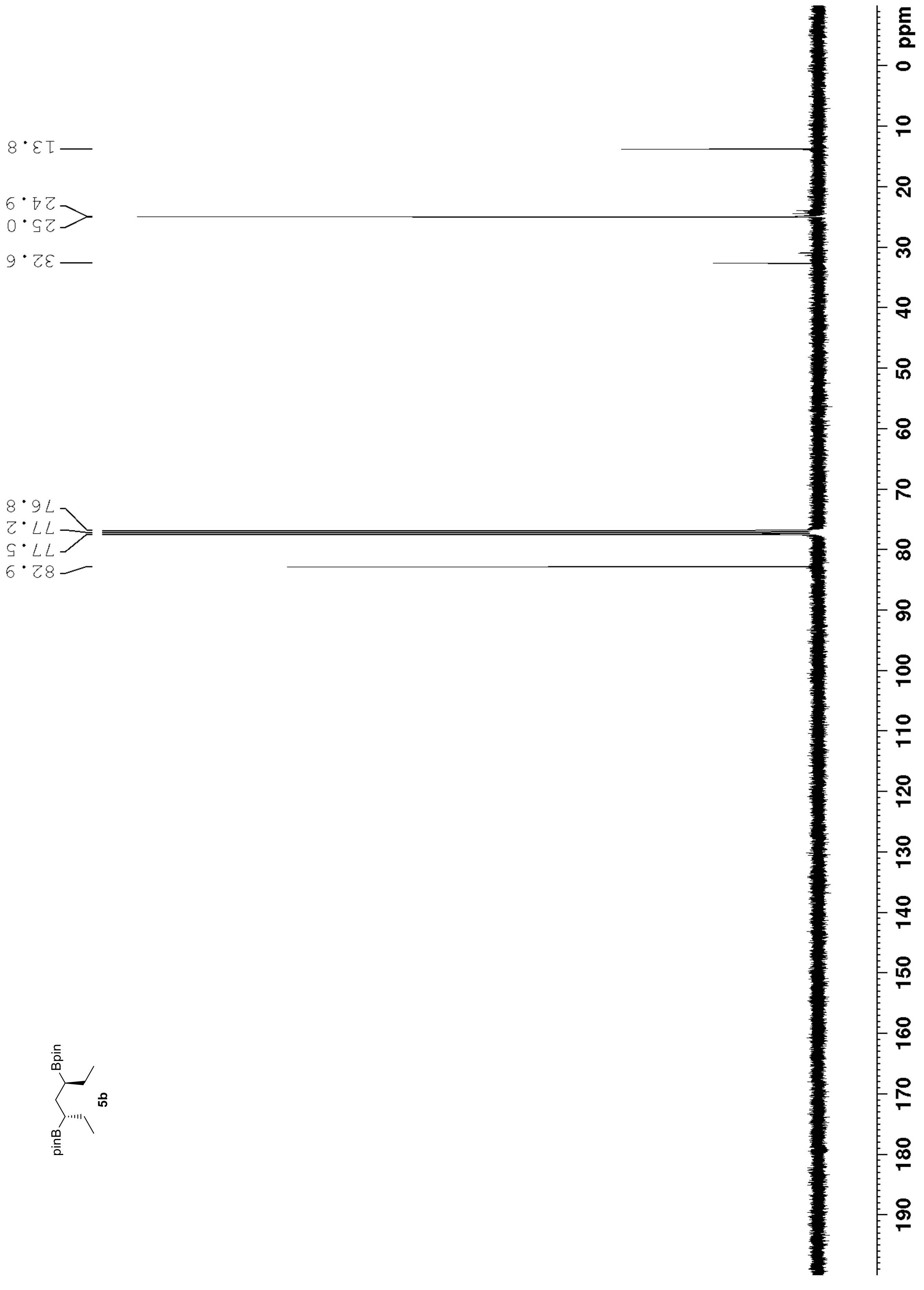
13.8
24.9
25.0
32.6
76.8
77.2
77.5
82.9
pinB
Bpin
5b
ppm

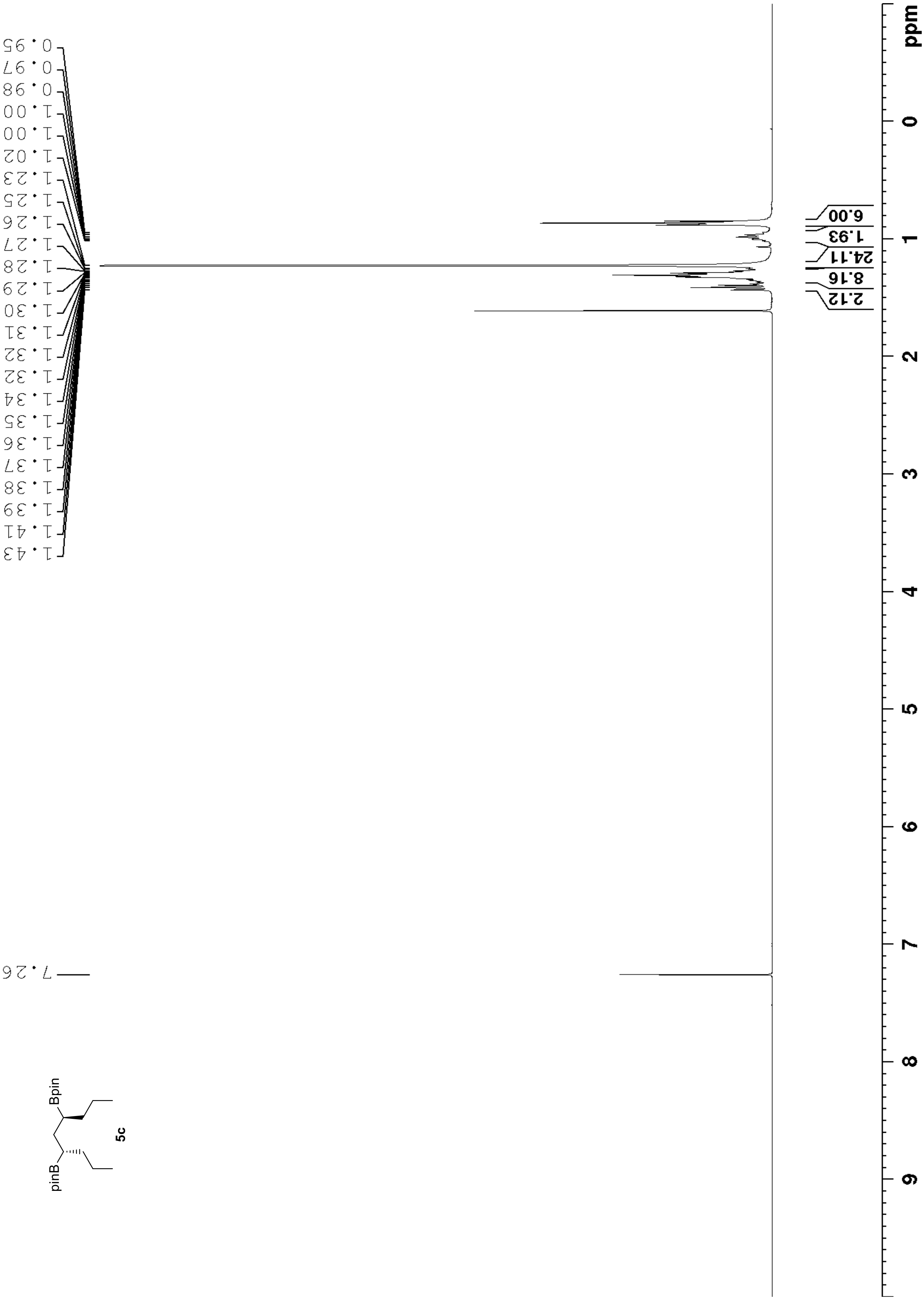
0.95
0.97
0.98
1.00
1.00
1.02
1.23
1.25
1.26
1.27
1.28
1.29
1.30
1.31
1.32
1.32
1.34
1.35
1.36
1.37
1.38
1.39
1.41
1.43
7.26
6.00
1.93
24.11
8.16
2.12
ppm
pinB
Bpin
5c

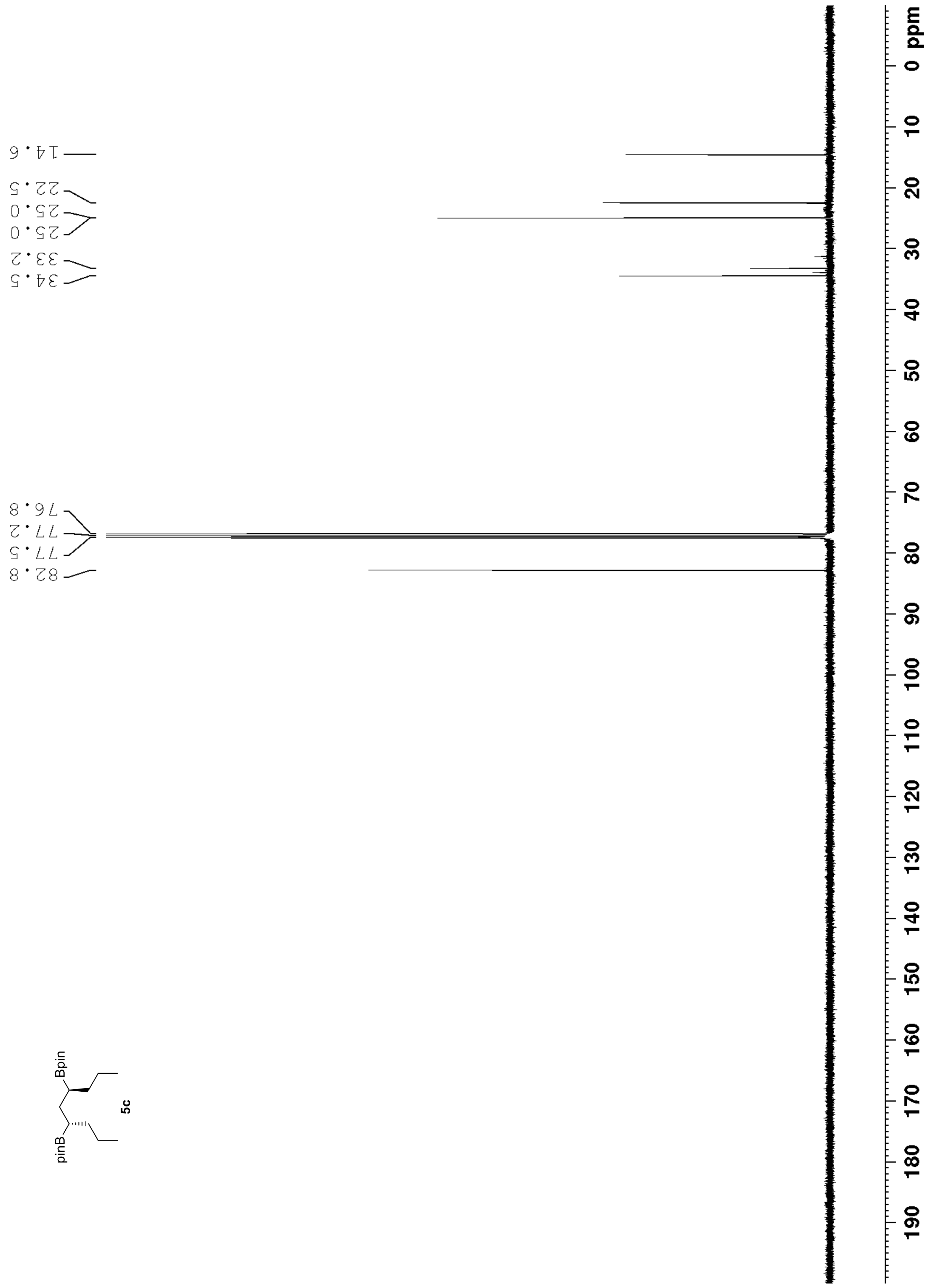
14.6
22.5
25.0
25.0
33.2
34.5
76.8
77.2
77.5
82.8
ppm
pinB
Bpin
5c

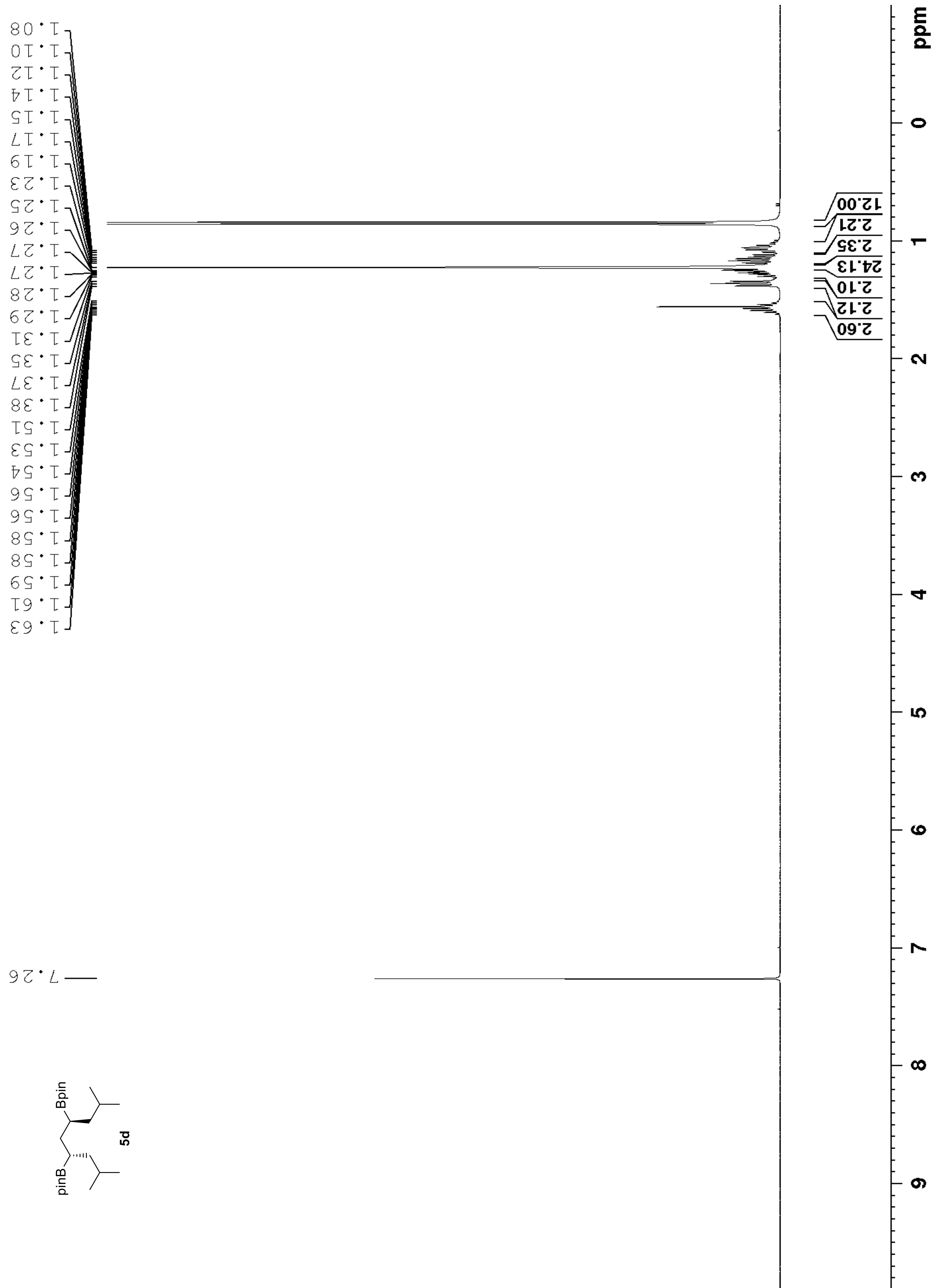
1.08
1.10
1.12
1.14
1.15
1.17
1.19
1.23
1.25
1.26
1.27
1.27
1.28
1.29
1.31
1.35
1.37
1.38
1.51
1.53
1.54
1.56
1.56
1.58
1.58
1.59
1.61
1.63
7.26
12.00
2.21
2.35
24.13
2.10
2.12
2.60
ppm
0
1
2
3
4
5
6
7
8
9
pinB
Bpin
5d

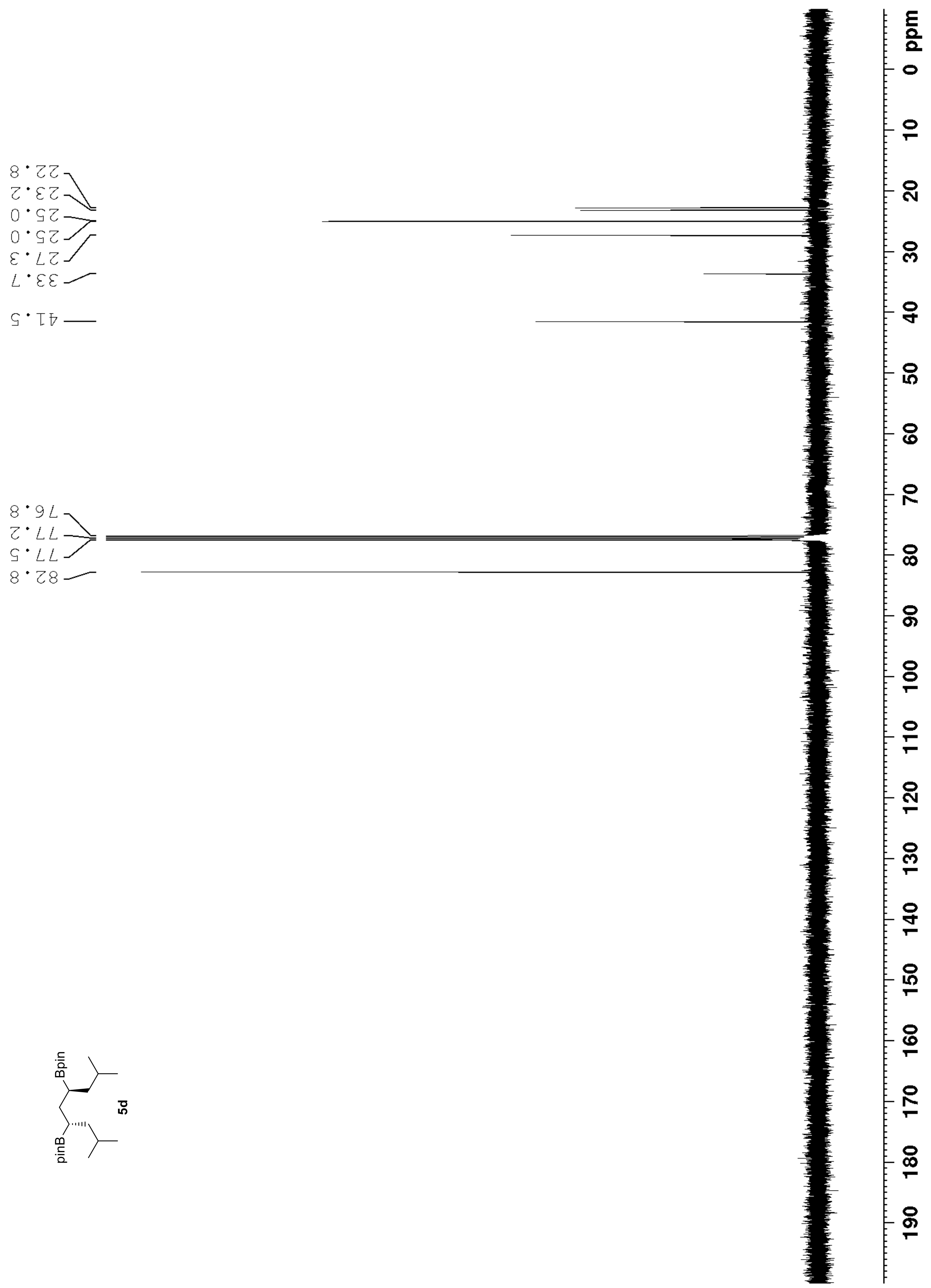
22.8
23.2
25.0
25.0
27.3
33.7
41.5
76.8
77.2
77.5
82.8
ppm
pinB
Bpin
5d

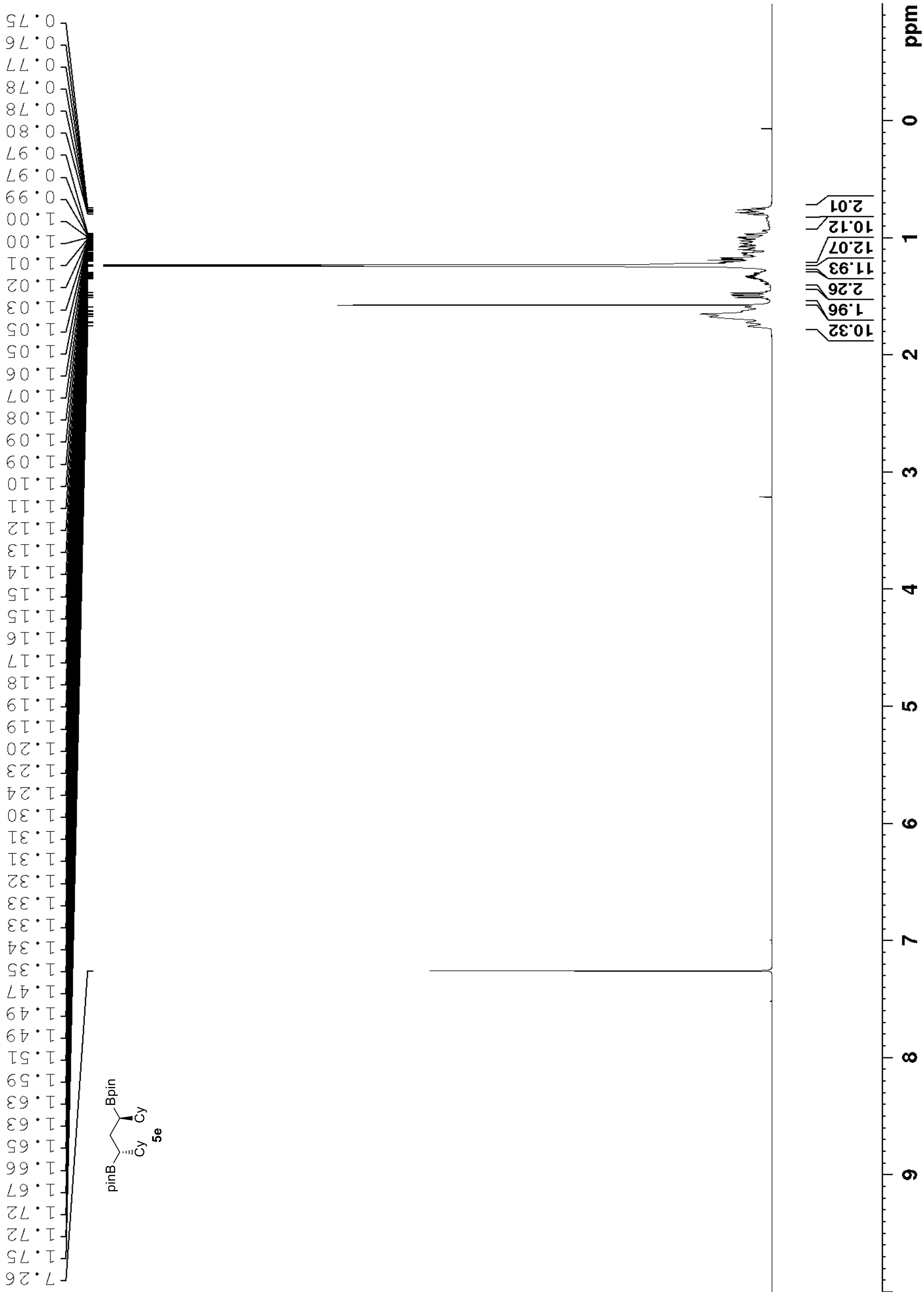
0.75
0.76
0.77
0.78
0.78
0.80
0.97
0.97
0.99
1.00
1.00
1.01
1.02
1.03
1.05
1.05
1.06
1.07
1.08
1.09
1.09
1.10
1.11
1.12
1.13
1.14
1.15
1.15
1.16
1.17
1.18
1.19
1.19
1.20
1.23
1.24
1.30
1.31
1.31
1.32
1.33
1.33
1.34
1.35
1.47
1.49
1.49
1.51
1.59
1.63
1.63
1.65
1.66
1.67
1.72
1.72
1.75
7.26
2.01
10.12
12.07
11.93
2.26
1.96
10.32
ppm
0
1
2
3
4
5
6
7
8
9
pinB
Cy
Cy
Bpin
5e

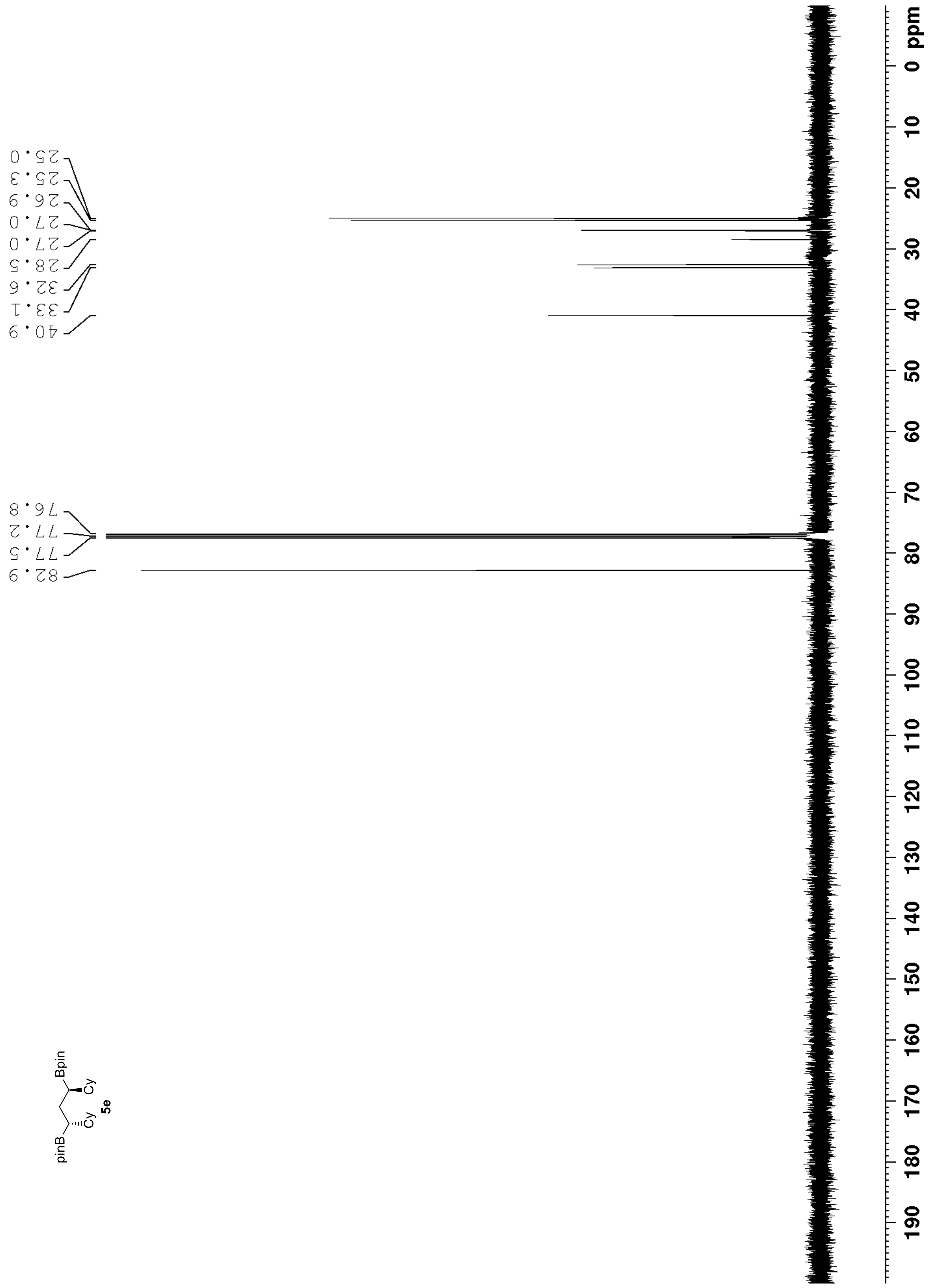
25.0
25.3
26.9
27.0
27.0
28.5
32.6
33.1
40.9
76.8
77.2
77.5
82.9
pinB
Bpin
Cy
Cy
5e
ppm

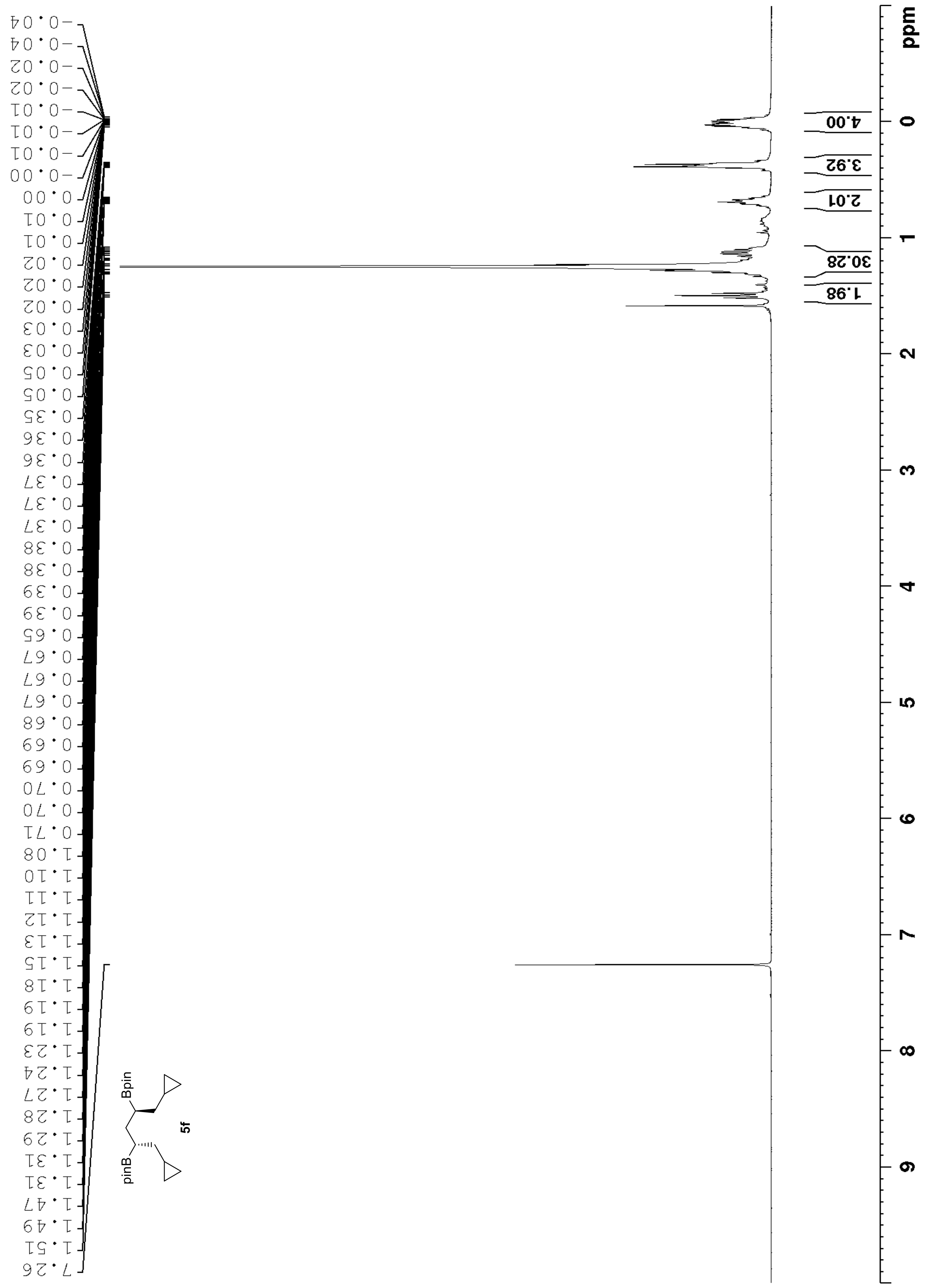
-0.04
-0.04
-0.02
-0.02
-0.01
-0.01
-0.01
-0.00
0.00
0.01
0.01
0.02
0.02
0.02
0.03
0.03
0.05
0.05
0.35
0.36
0.36
0.37
0.37
0.37
0.38
0.38
0.39
0.39
0.65
0.67
0.67
0.67
0.68
0.69
0.69
0.70
0.70
0.71
1.08
1.10
1.11
1.12
1.13
1.15
1.18
1.19
1.19
1.23
1.24
1.27
1.28
1.29
1.31
1.31
1.47
1.49
1.51
7.26
Bpin
pinB
5f
4.00
3.92
2.01
30.28
1.98
ppm
0
1
2
3
4
5
6
7
8
9

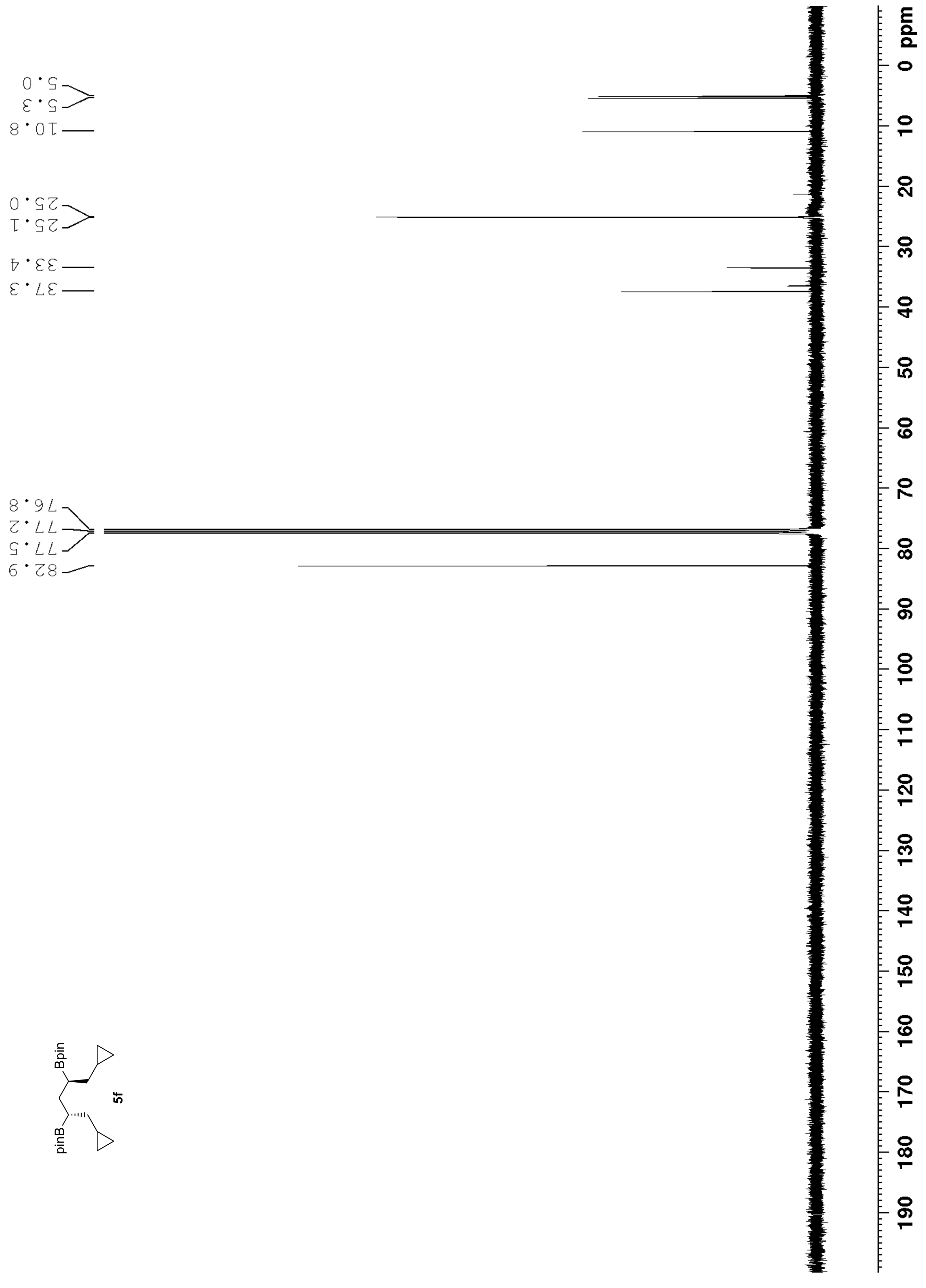
5.0
5.3
10.8
25.0
25.1
33.4
37.3
76.8
77.2
77.5
82.9
ppm
Bpin
pinB
5f

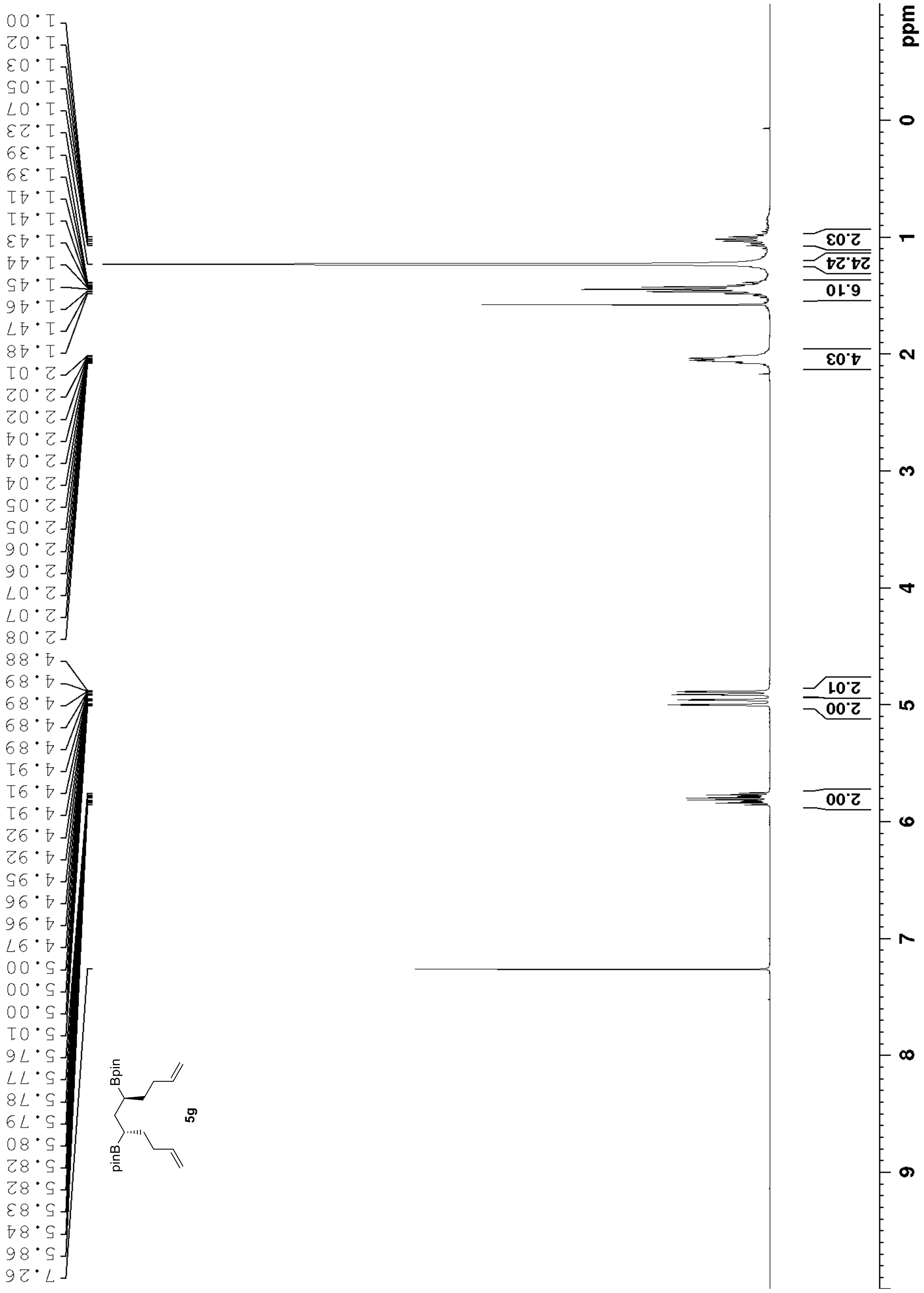
1.00
1.02
1.03
1.05
1.07
1.23
1.39
1.39
1.41
1.41
1.43
1.44
1.45
1.46
1.47
1.48
2.01
2.02
2.02
2.04
2.04
2.04
2.05
2.05
2.06
2.06
2.07
2.07
2.08
4.88
4.89
4.89
4.89
4.89
4.91
4.91
4.91
4.92
4.92
4.95
4.96
4.96
4.97
5.00
5.00
5.00
5.01
5.76
5.77
5.78
5.79
5.80
5.82
5.82
5.83
5.84
5.86
7.26
Bpin
pinB
5g
2.03
24.24
6.10
4.03
2.01
2.00
2.00
ppm
0
1
2
3
4
5
6
7
8
9

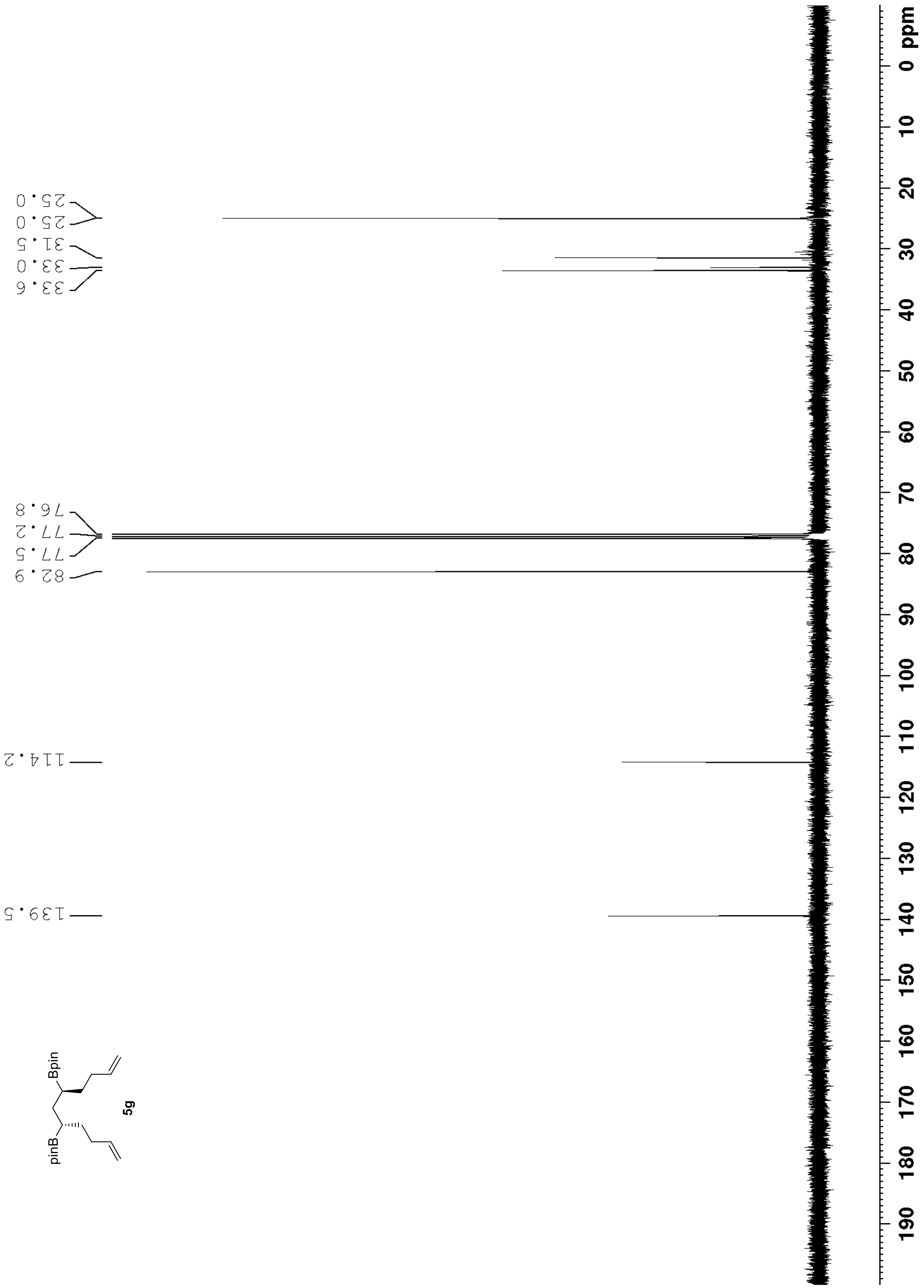
25.0
25.0
31.5
33.0
33.6
76.8
77.2
77.5
82.9
114.2
139.5
Bpin
pinB
5g
ppm

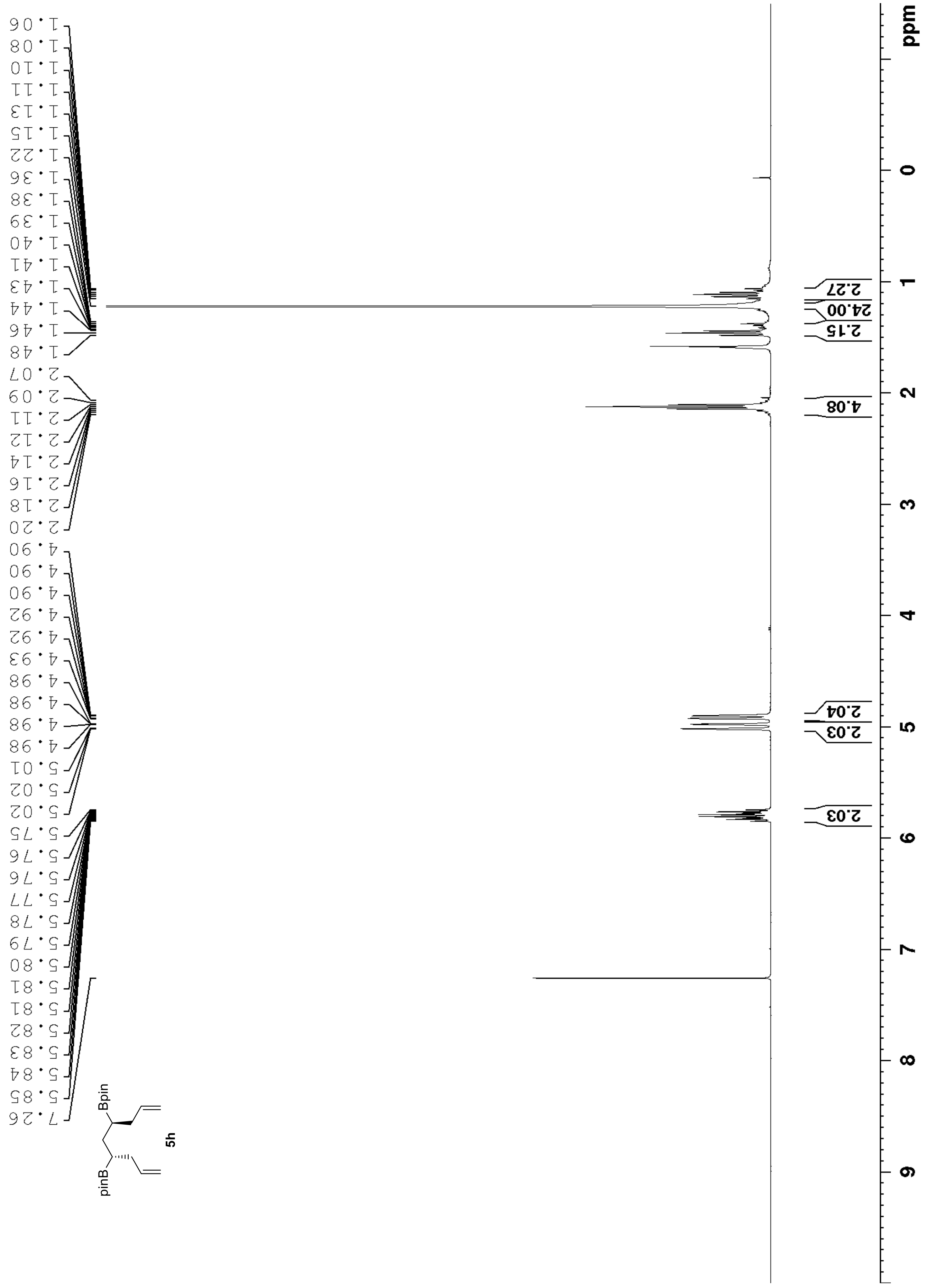
pinB
Bpin
5h
ppm

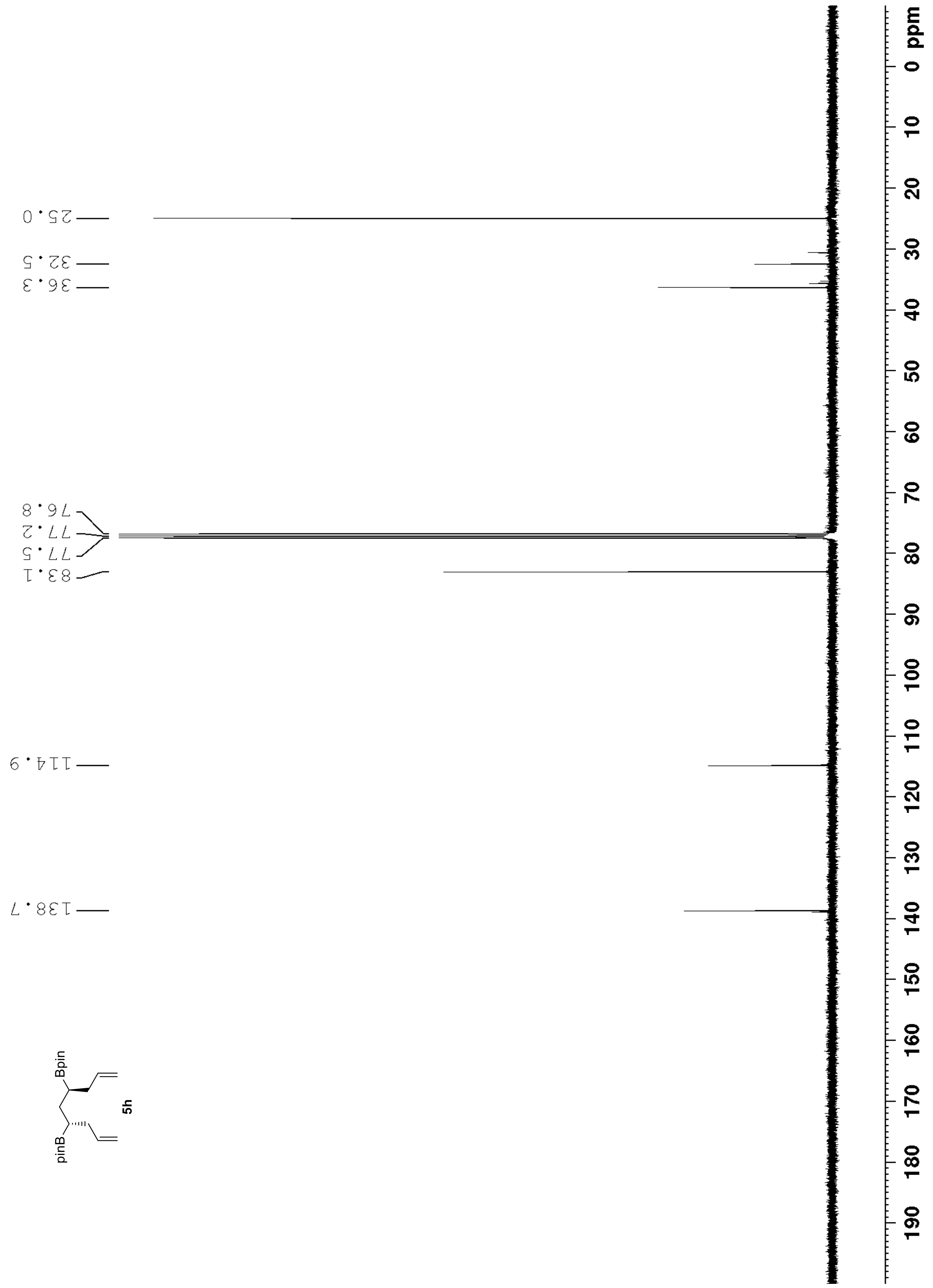
25.0
32.5
36.3
76.8
77.2
77.5
83.1
114.9
138.7
ppm
Bpin
pinB
5h

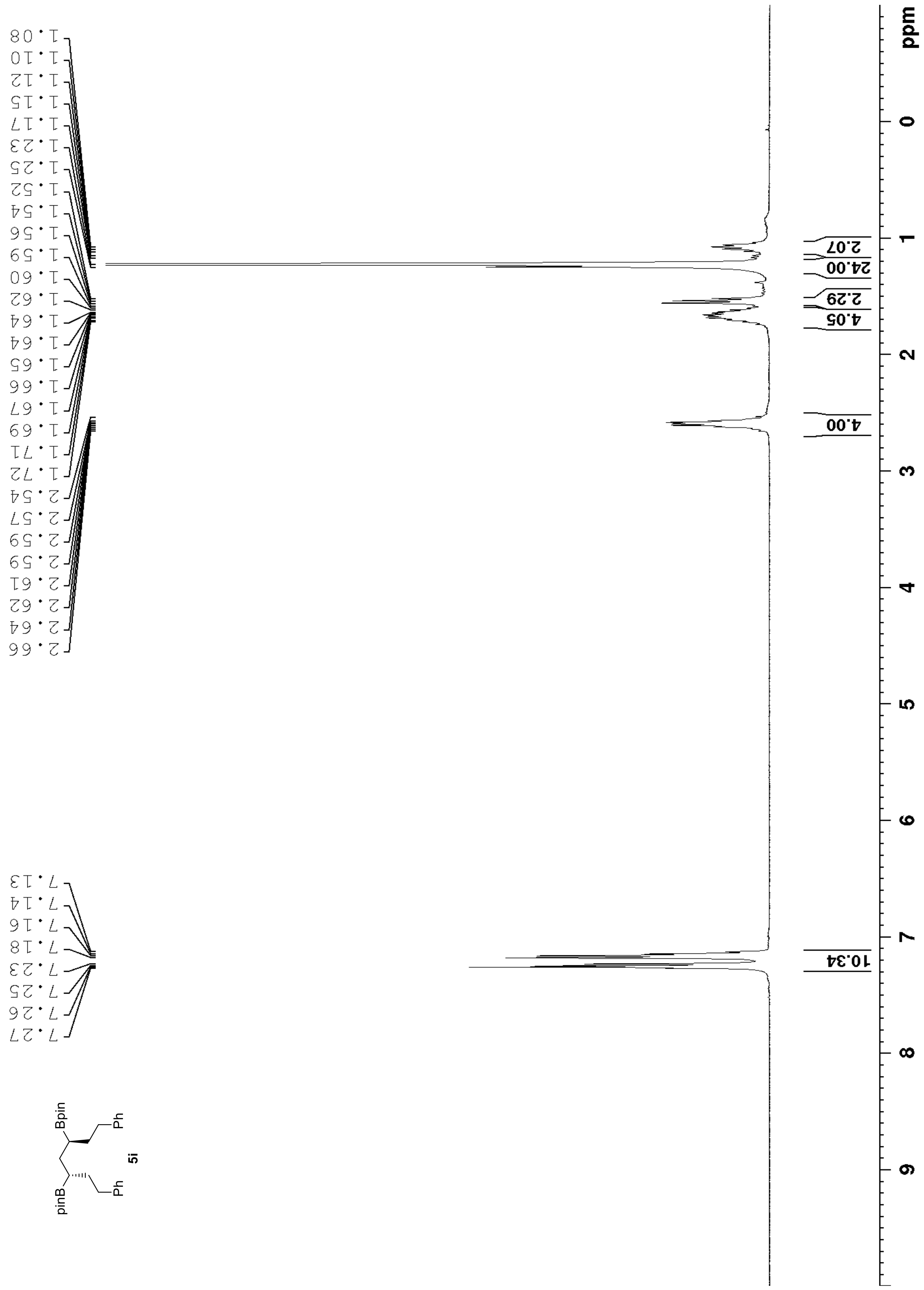
1.08
1.10
1.12
1.15
1.17
1.23
1.25
1.52
1.54
1.56
1.59
1.60
1.62
1.64
1.64
1.65
1.66
1.67
1.69
1.71
1.72
2.54
2.57
2.59
2.59
2.61
2.62
2.64
2.66
7.13
7.14
7.16
7.18
7.23
7.25
7.26
7.27
2.07
24.00
2.29
4.05
4.00
10.34
ppm
0
1
2
3
4
5
6
7
8
9
Bpin
pinB
Ph
Ph
5i

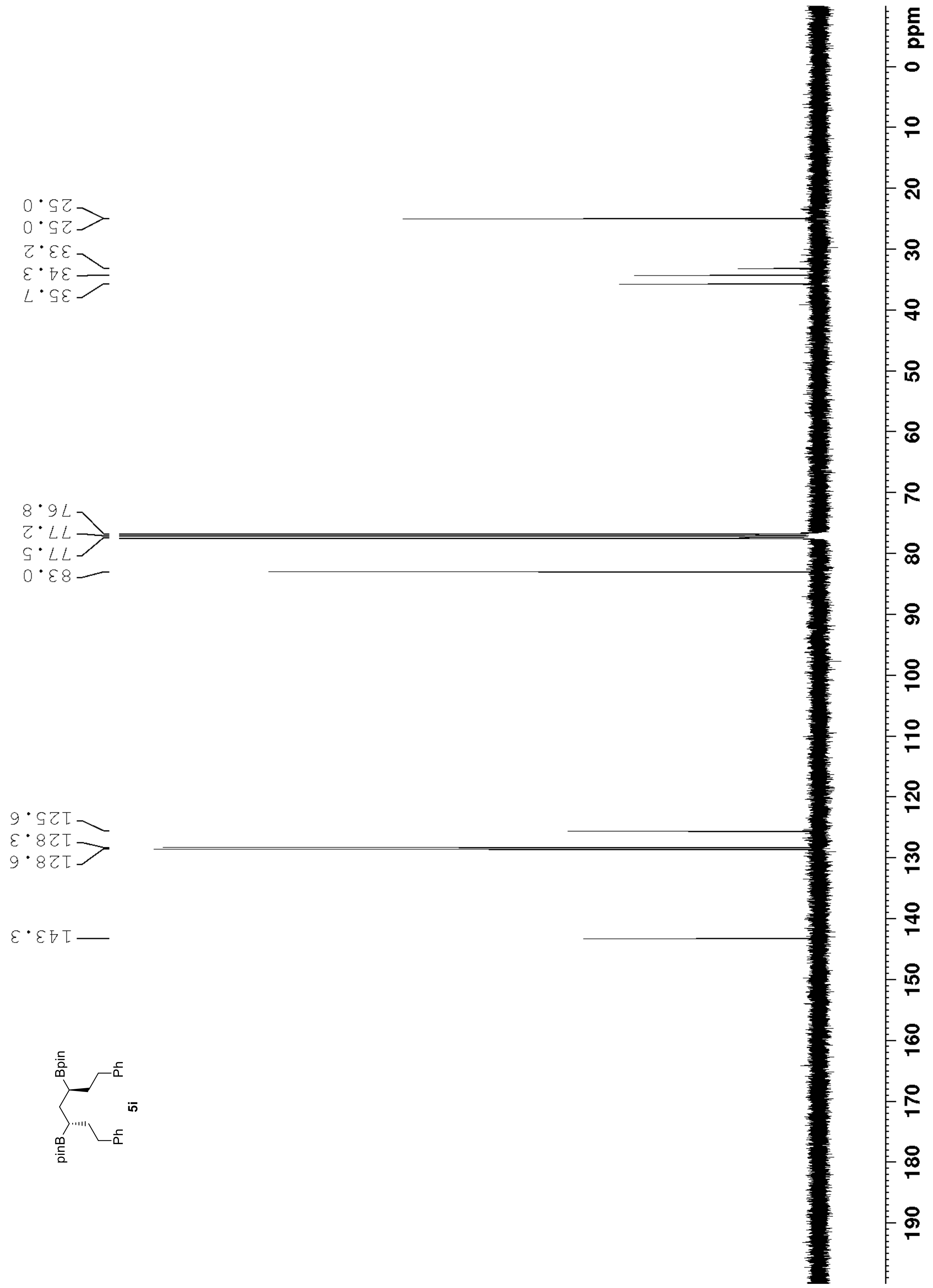
25.0
25.0
33.2
34.3
35.7
76.8
77.2
77.5
83.0
125.6
128.3
128.6
143.3
pinB
Bpin
Ph
Ph
5i
ppm
0
10
20
30
40
50
60
70
80
90
100
110
120
130
140
150
160
170
180
190

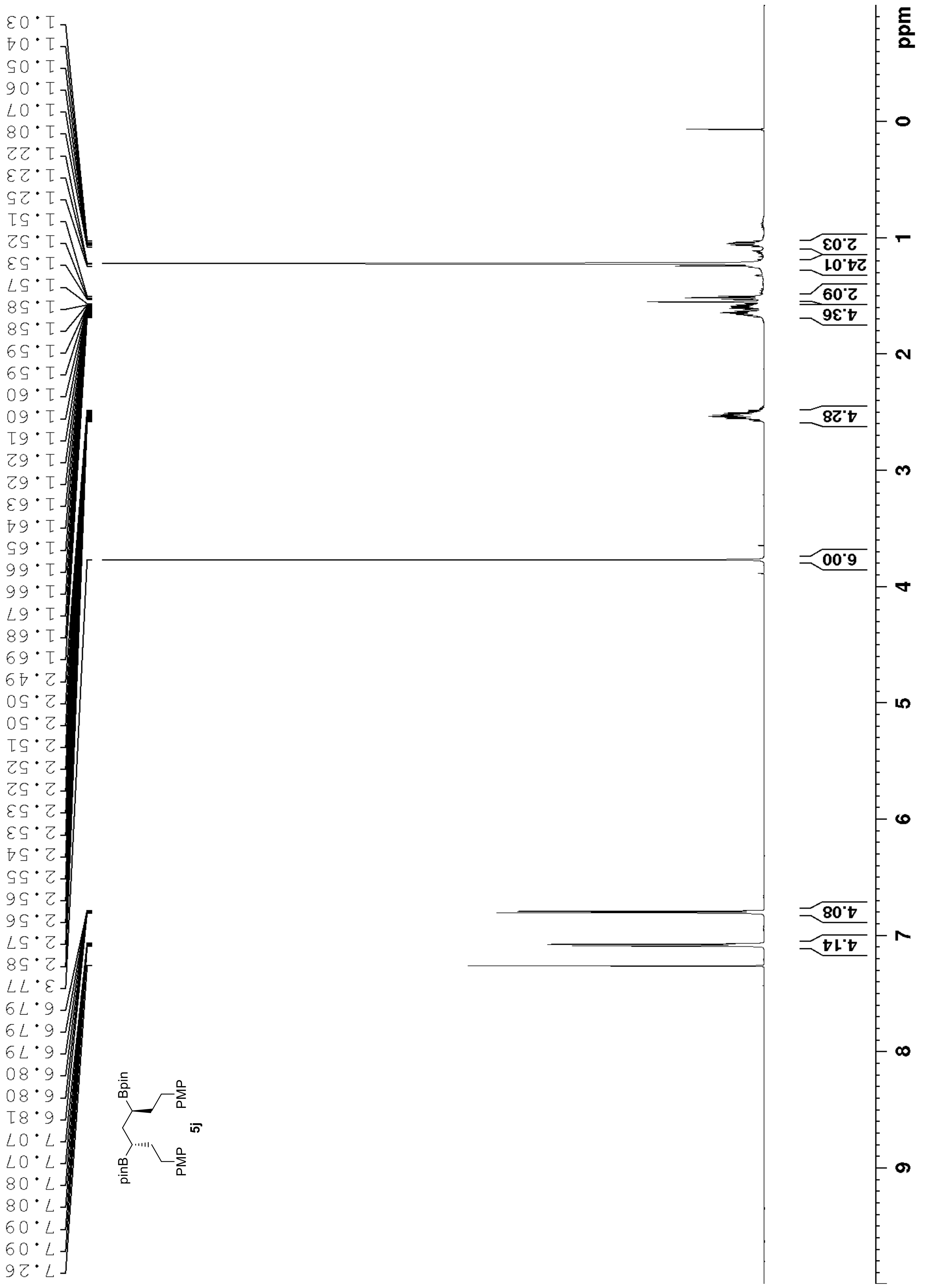
1.03
1.04
1.05
1.06
1.07
1.08
1.22
1.23
1.25
1.51
1.52
1.53
1.57
1.58
1.58
1.59
1.59
1.60
1.60
1.61
1.62
1.62
1.63
1.64
1.65
1.66
1.66
1.67
1.68
1.69
2.49
2.50
2.50
2.51
2.52
2.52
2.53
2.53
2.54
2.55
2.56
2.56
2.57
2.58
3.77
6.79
6.79
6.79
6.80
6.80
6.81
7.07
7.07
7.08
7.08
7.09
7.09
7.26
2.03
24.01
2.09
4.36
4.28
6.00
4.08
4.14
ppm
0
1
2
3
4
5
6
7
8
9
Bpin
PMP
pinB
PMP
5j

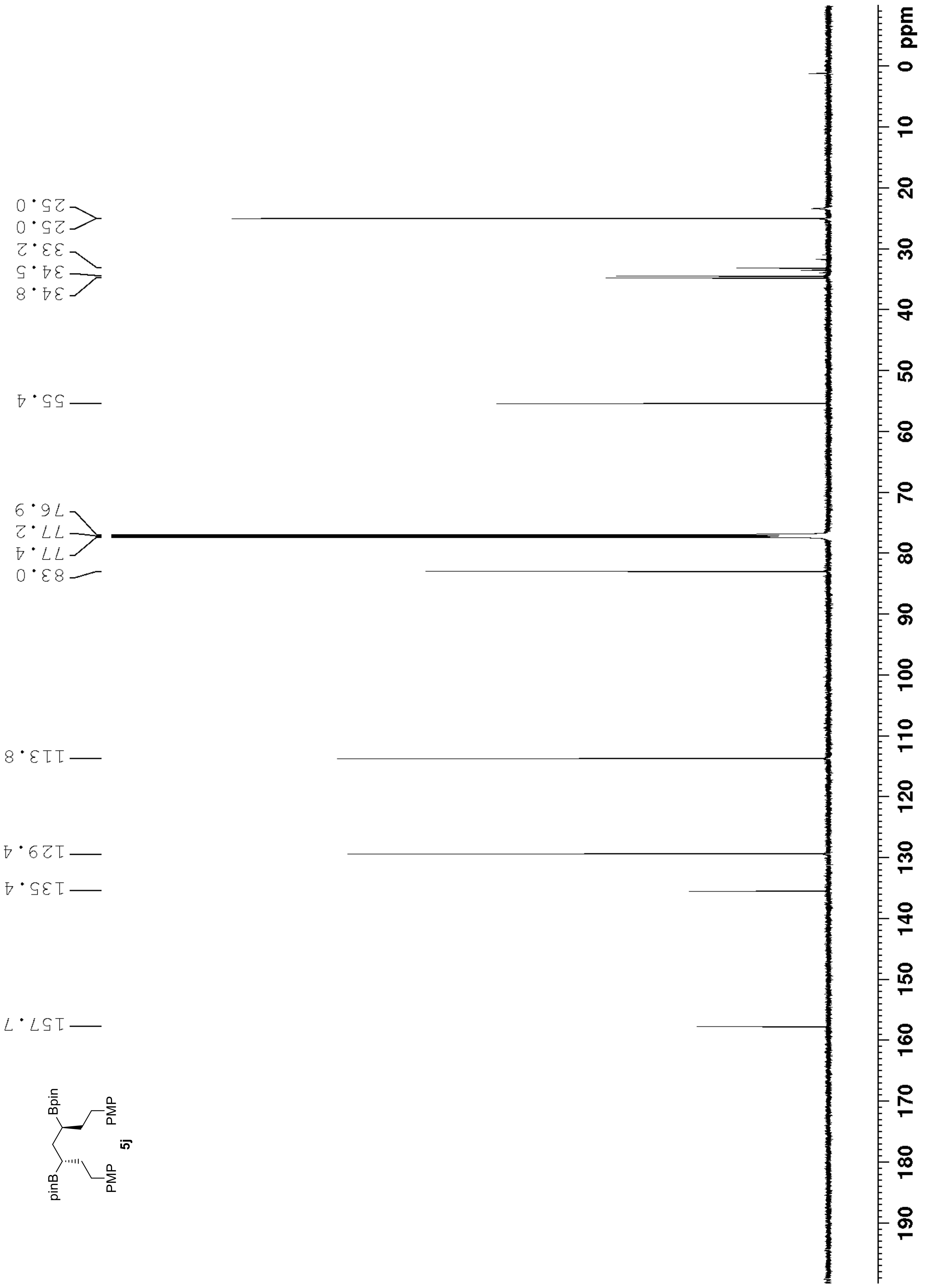
25.0
25.0
33.2
34.5
34.8
55.4
76.9
77.2
77.4
83.0
113.8
129.4
135.4
157.7
Bpin
pinB
PMP
PMP
5j
ppm
0
10
20
30
40
50
60
70
80
90
100
110
120
130
140
150
160
170
180
190

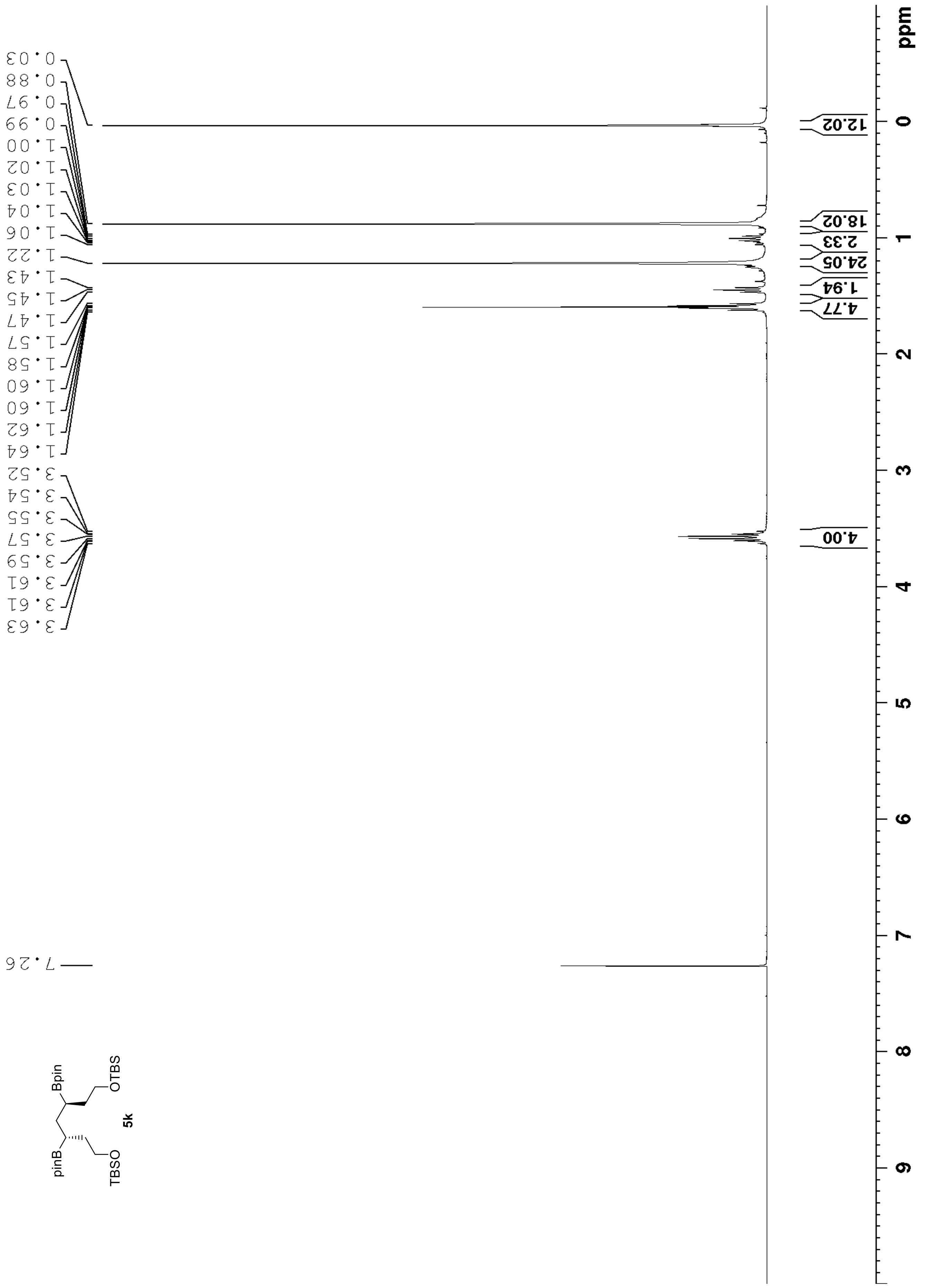
0.03
0.88
0.97
0.99
1.00
1.02
1.03
1.04
1.06
1.22
1.43
1.45
1.47
1.57
1.58
1.60
1.60
1.62
1.64
3.52
3.54
3.55
3.57
3.59
3.61
3.61
3.63
7.26
12.02
18.02
2.33
24.05
1.94
4.77
4.00
ppm
pinB
Bpin
TBSO
OTBS
5k

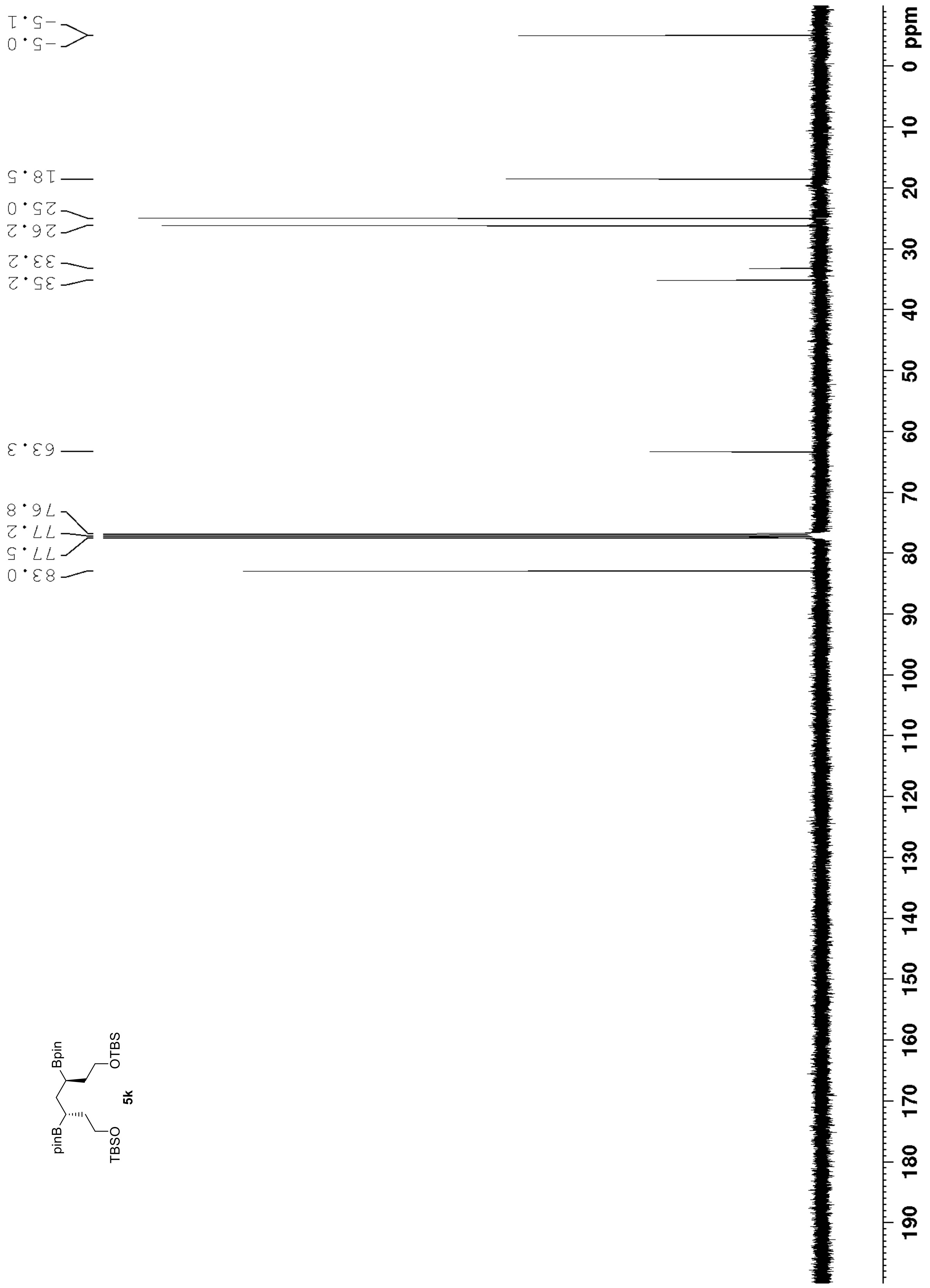
-5.1
-5.0
18.5
25.0
26.2
33.2
35.2
63.3
76.8
77.2
77.5
83.0
ppm
Bpin
OTBS
pinB
TBSO
5k

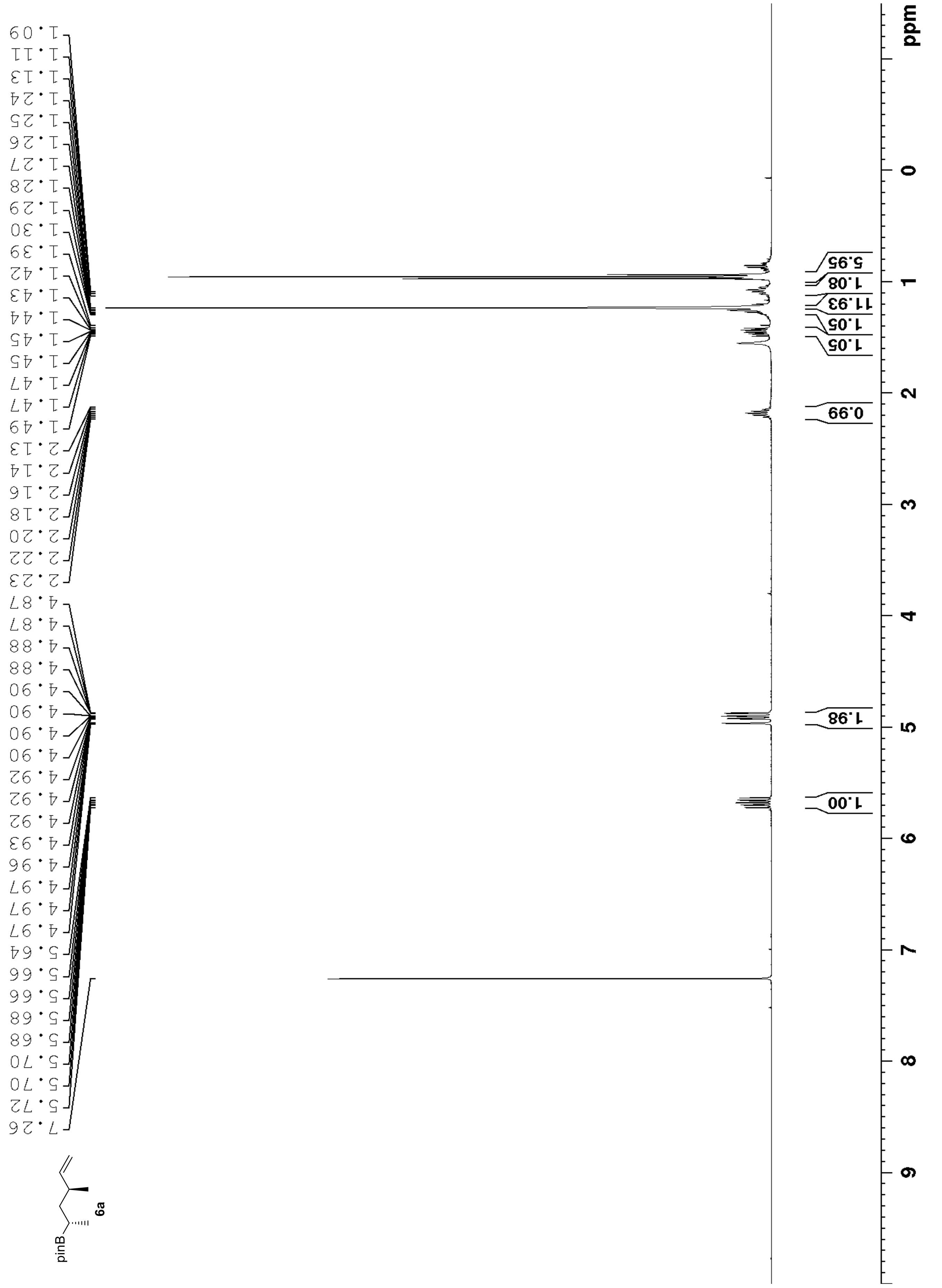
1.09
1.11
1.13
1.24
1.25
1.26
1.27
1.28
1.29
1.30
1.39
1.42
1.43
1.44
1.45
1.45
1.47
1.47
1.49
2.13
2.14
2.16
2.18
2.20
2.22
2.23
4.87
4.87
4.88
4.88
4.90
4.90
4.90
4.90
4.92
4.92
4.92
4.93
4.96
4.97
4.97
4.97
5.64
5.66
5.66
5.68
5.68
5.70
5.70
5.72
7.26
5.95
1.08
11.93
1.05
1.05
0.99
1.98
1.00
ppm
0
1
2
3
4
5
6
7
8
9
pinB
6a

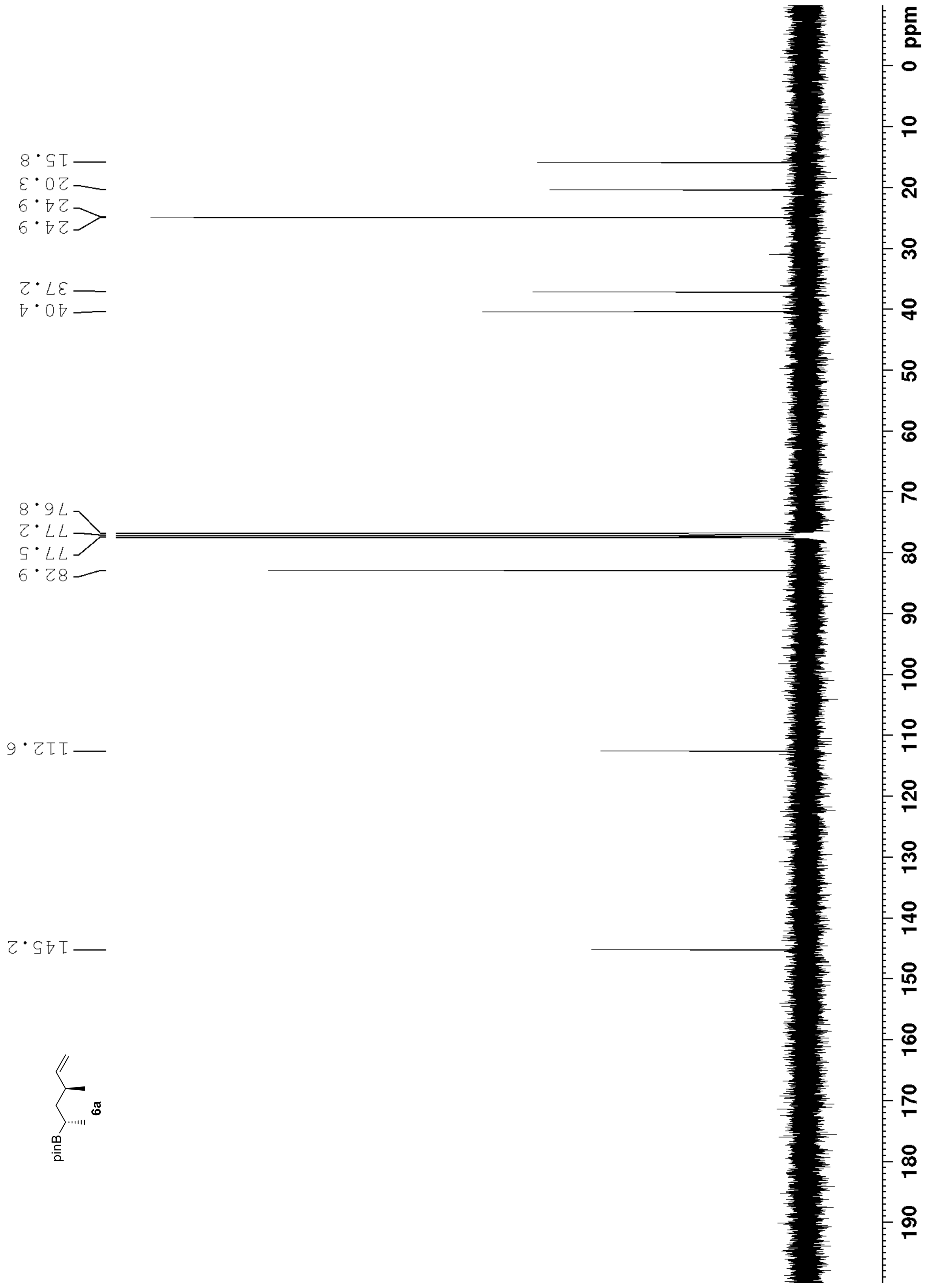
15.8
20.3
24.9
24.9
37.2
40.4
76.8
77.2
77.5
82.9
112.6
145.2
pinB
6a
ppm
0
10
20
30
40
50
60
70
80
90
100
110
120
130
140
150
160
170
180
190

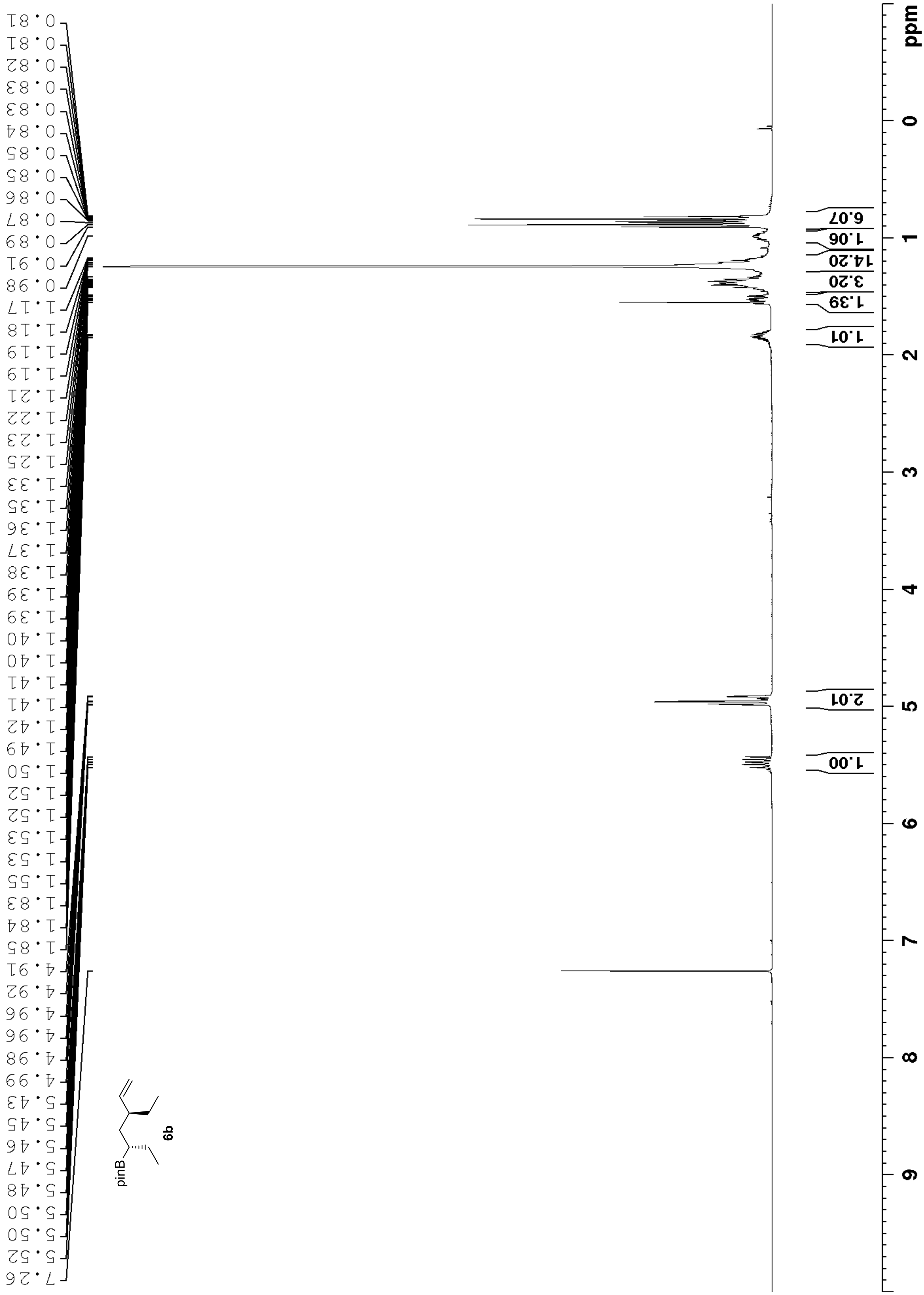
0.81
0.81
0.82
0.83
0.83
0.84
0.85
0.85
0.86
0.87
0.89
0.91
0.98
1.17
1.18
1.19
1.19
1.21
1.22
1.23
1.25
1.33
1.35
1.36
1.37
1.38
1.39
1.39
1.40
1.40
1.41
1.41
1.42
1.49
1.50
1.52
1.52
1.53
1.53
1.55
1.83
1.84
1.85
4.91
4.92
4.96
4.96
4.98
4.99
5.43
5.45
5.46
5.47
5.48
5.50
5.50
5.52
7.26
pinB
6b
6.07
1.06
14.20
3.20
1.39
1.01
2.01
1.00
ppm
0
1
2
3
4
5
6
7
8
9

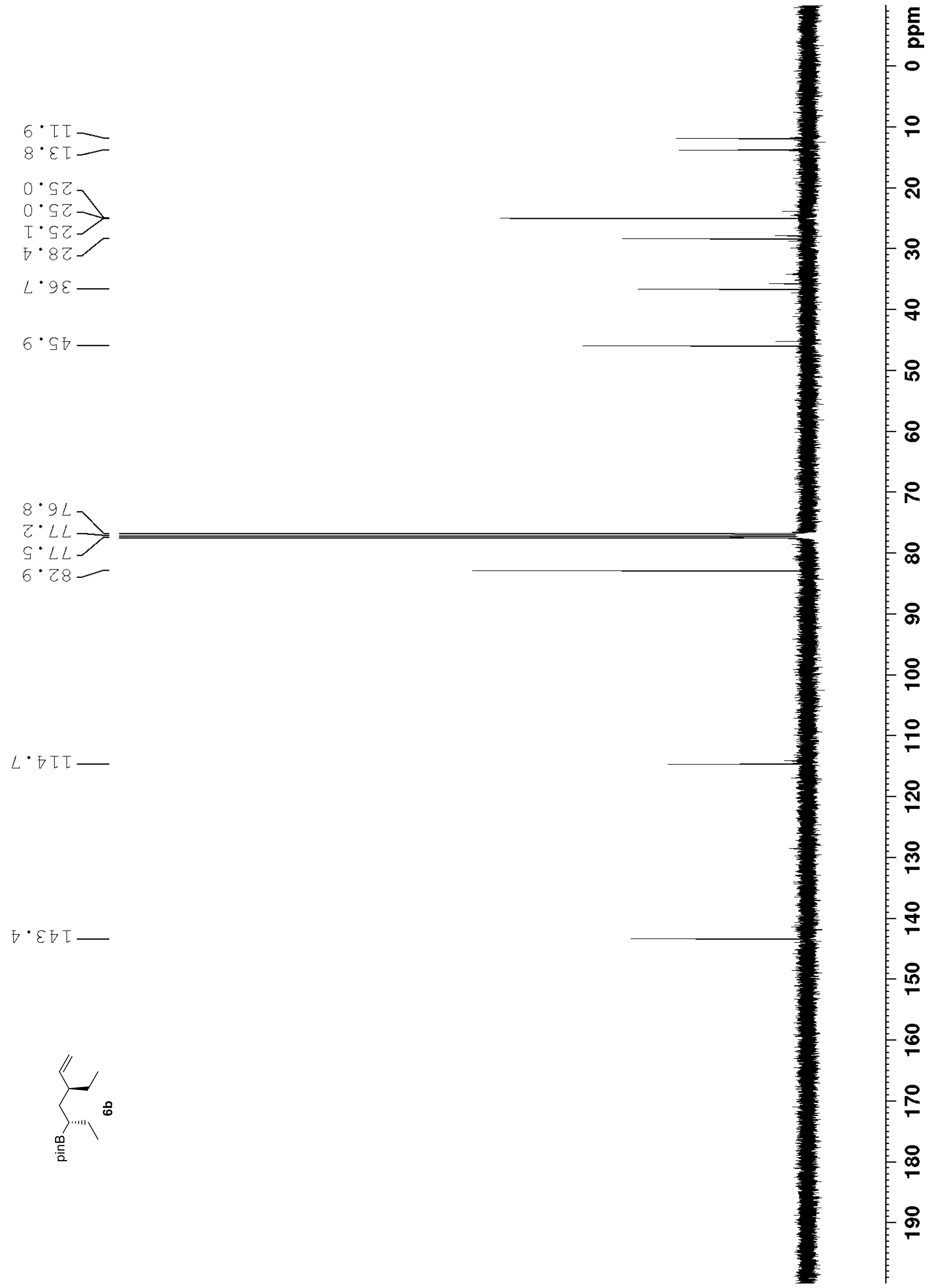
11.9
13.8
25.0
25.0
25.1
28.4
36.7
45.9
76.8
77.2
77.5
82.9
114.7
143.4
pinB
6b
ppm

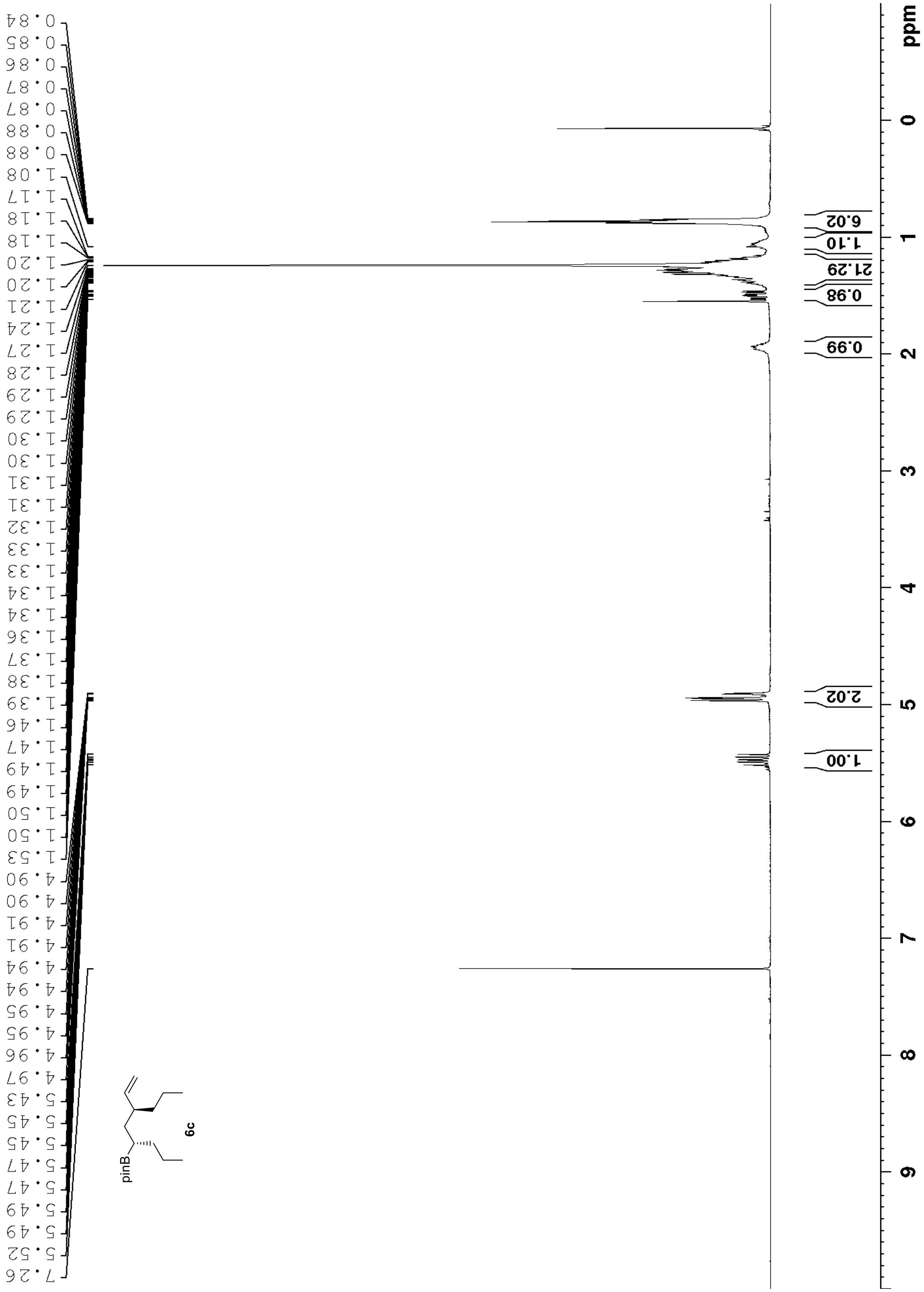
0.84
0.85
0.86
0.87
0.87
0.88
0.88
1.08
1.17
1.18
1.18
1.20
1.20
1.21
1.24
1.27
1.28
1.29
1.29
1.30
1.30
1.31
1.31
1.32
1.33
1.33
1.34
1.34
1.36
1.37
1.38
1.39
1.46
1.47
1.49
1.49
1.50
1.50
1.53
4.90
4.90
4.91
4.91
4.94
4.94
4.95
4.95
4.96
4.97
5.43
5.45
5.45
5.47
5.47
5.49
5.49
5.52
7.26
6.02
1.10
21.29
0.98
0.99
2.02
1.00
ppm
0
1
2
3
4
5
6
7
8
9
pinB
6c

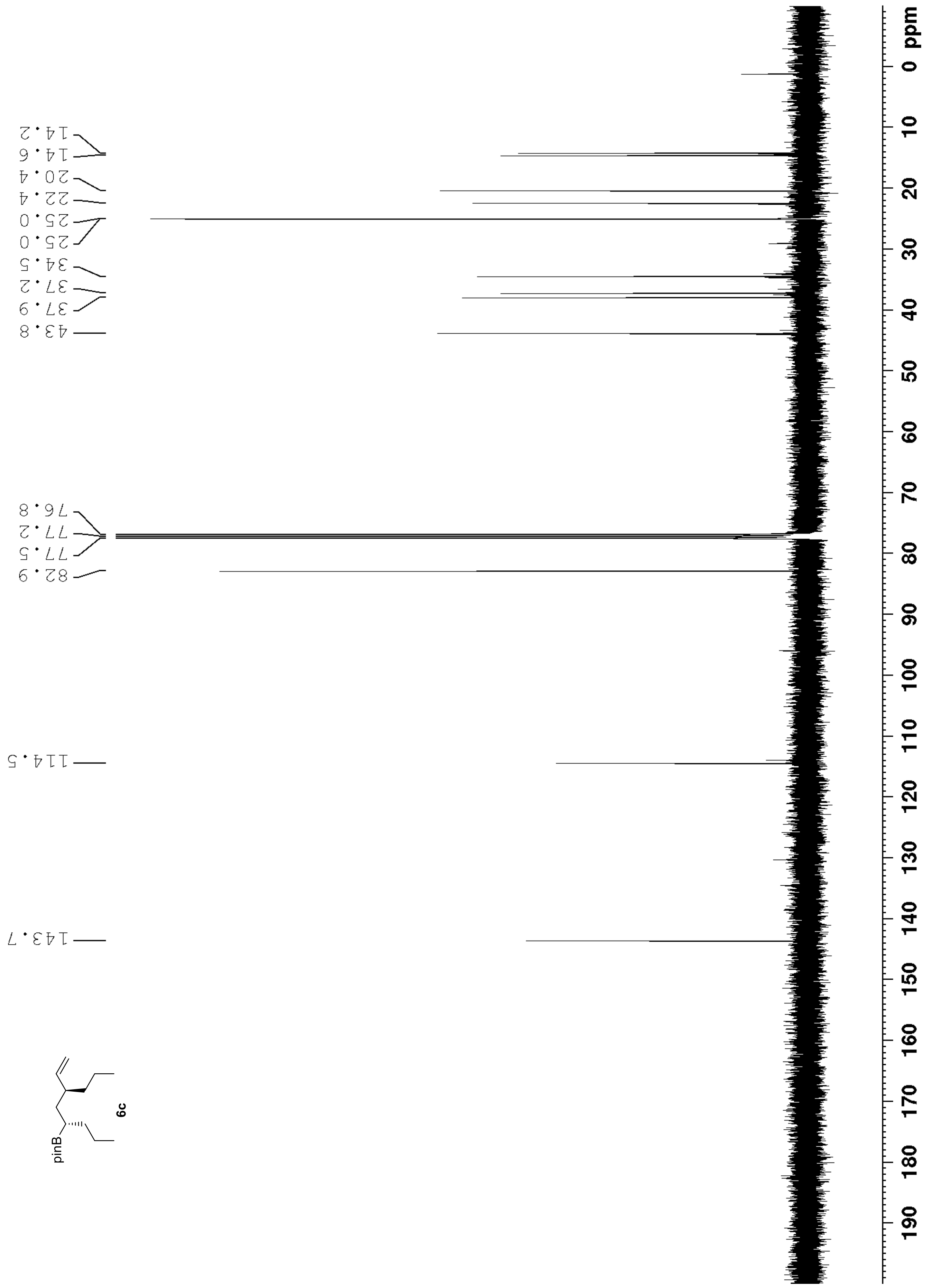
14.2
14.6
20.4
22.4
25.0
25.0
34.5
37.2
37.9
43.8
76.8
77.2
77.5
82.9
114.5
143.7
pinB
6c
ppm

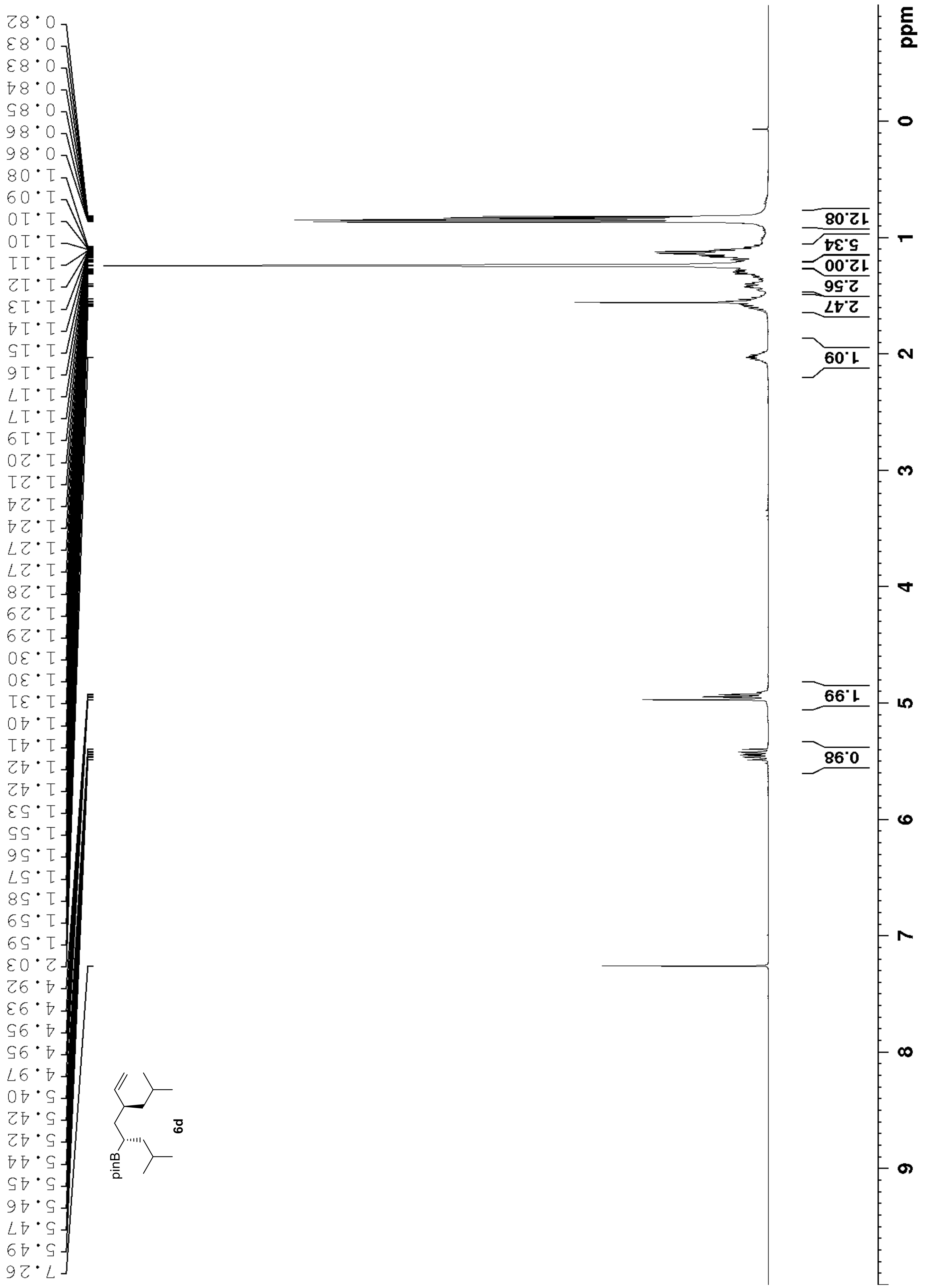
0.82
0.83
0.83
0.84
0.85
0.86
0.86
1.08
1.09
1.10
1.10
1.11
1.12
1.13
1.14
1.15
1.16
1.17
1.17
1.19
1.20
1.21
1.24
1.24
1.27
1.27
1.28
1.29
1.29
1.30
1.30
1.31
1.40
1.41
1.42
1.42
1.53
1.55
1.56
1.57
1.58
1.59
1.59
2.03
4.92
4.93
4.95
4.95
4.97
5.40
5.42
5.42
5.44
5.45
5.46
5.47
5.49
7.26
12.08
5.34
12.00
2.56
2.47
1.09
1.99
0.98
ppm
pinB
6d

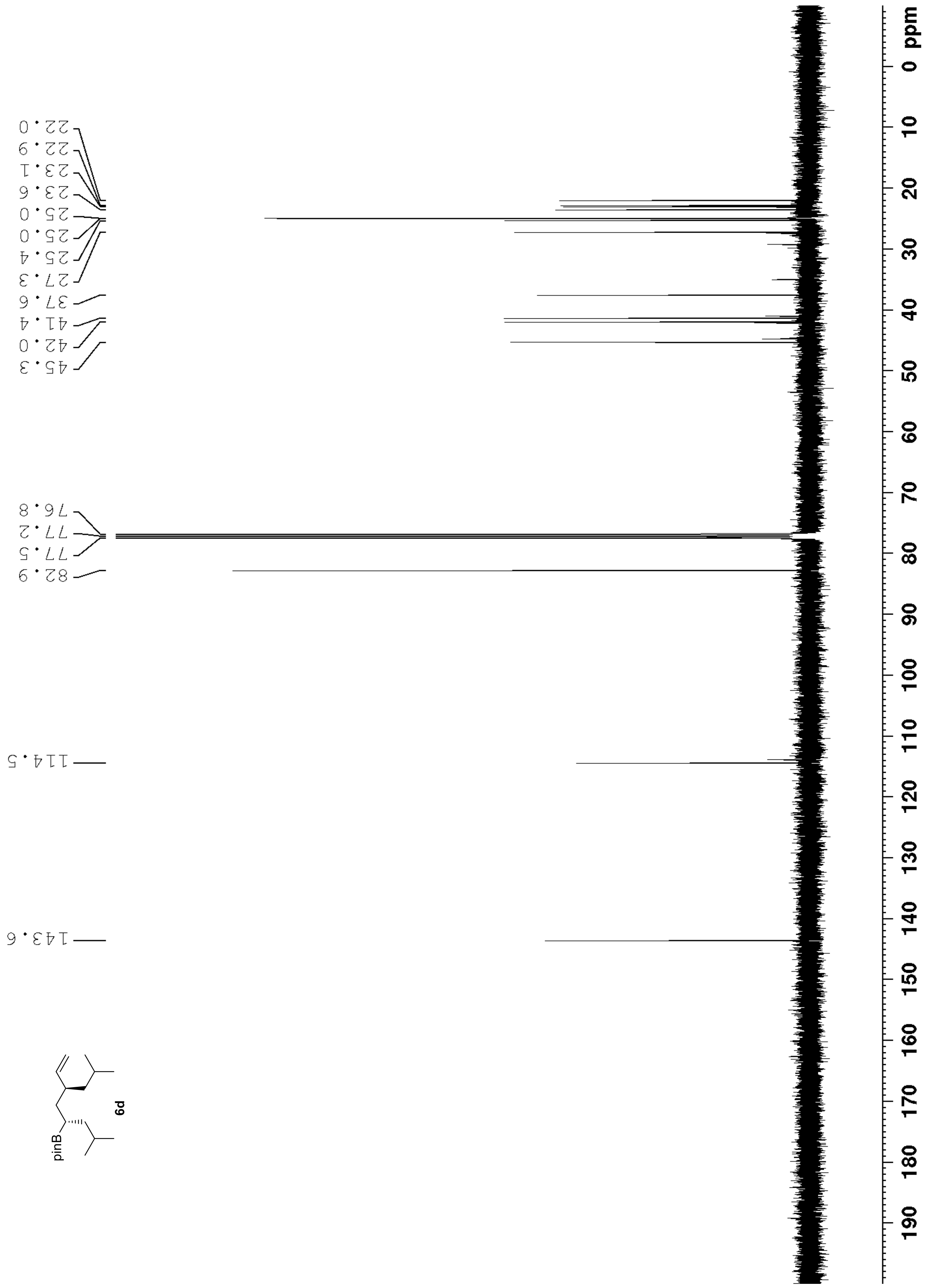
22.0
22.9
23.1
23.6
25.0
25.0
25.4
27.3
37.6
41.4
42.0
45.3
76.8
77.2
77.5
82.9
114.5
143.6
pinB
6d
ppm

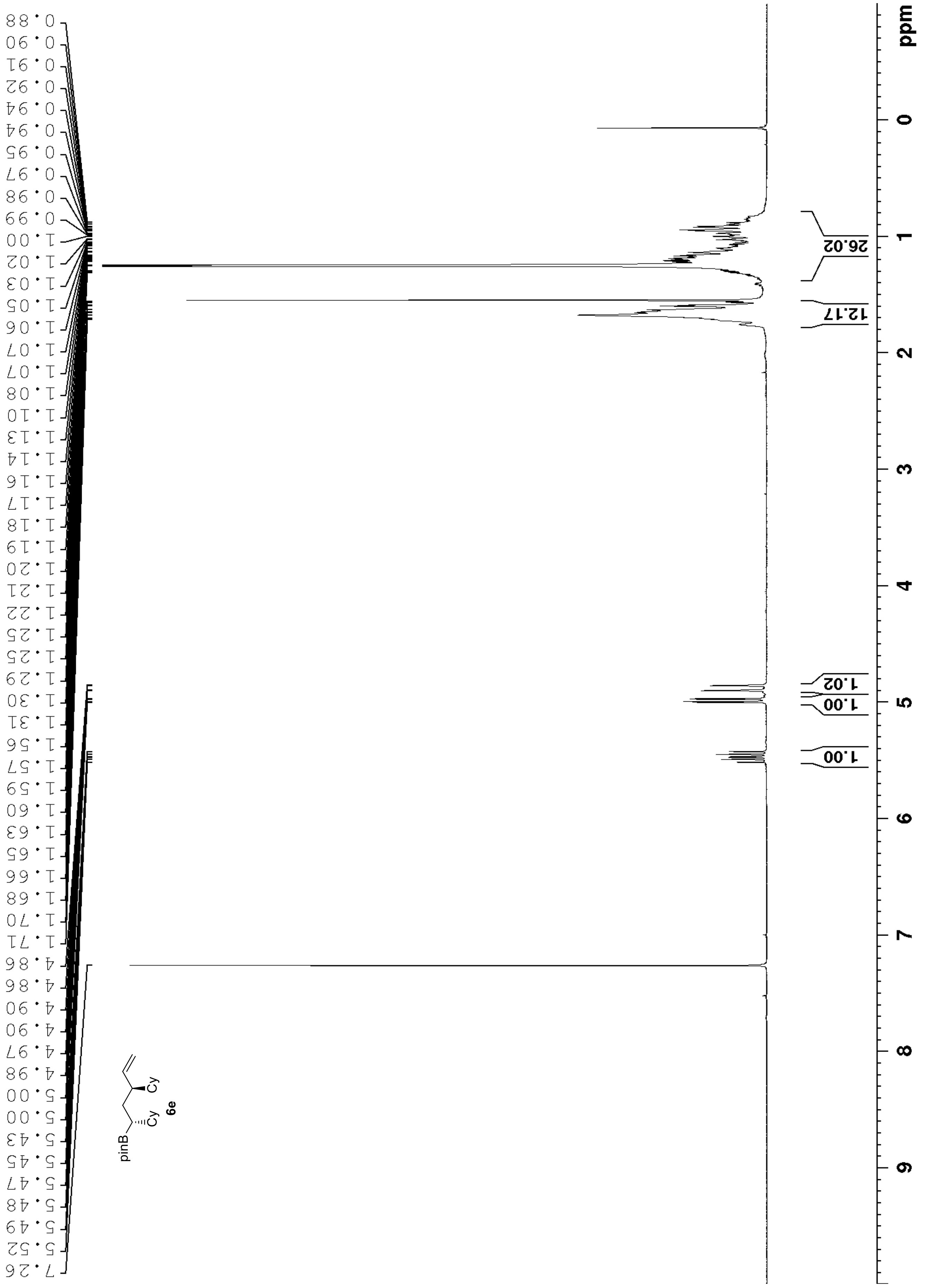
0.88
0.90
0.91
0.92
0.94
0.94
0.95
0.97
0.98
0.99
1.00
1.02
1.03
1.05
1.06
1.07
1.07
1.08
1.10
1.13
1.14
1.16
1.17
1.18
1.19
1.20
1.21
1.22
1.25
1.25
1.29
1.30
1.31
1.56
1.57
1.59
1.60
1.63
1.65
1.66
1.68
1.70
1.71
4.86
4.86
4.90
4.90
4.97
4.98
5.00
5.00
5.43
5.45
5.47
5.48
5.49
5.52
7.26
pinB
Cy
Cy
6e
26.02
12.17
1.02
1.00
1.00
ppm
0
1
2
3
4
5
6
7
8
9

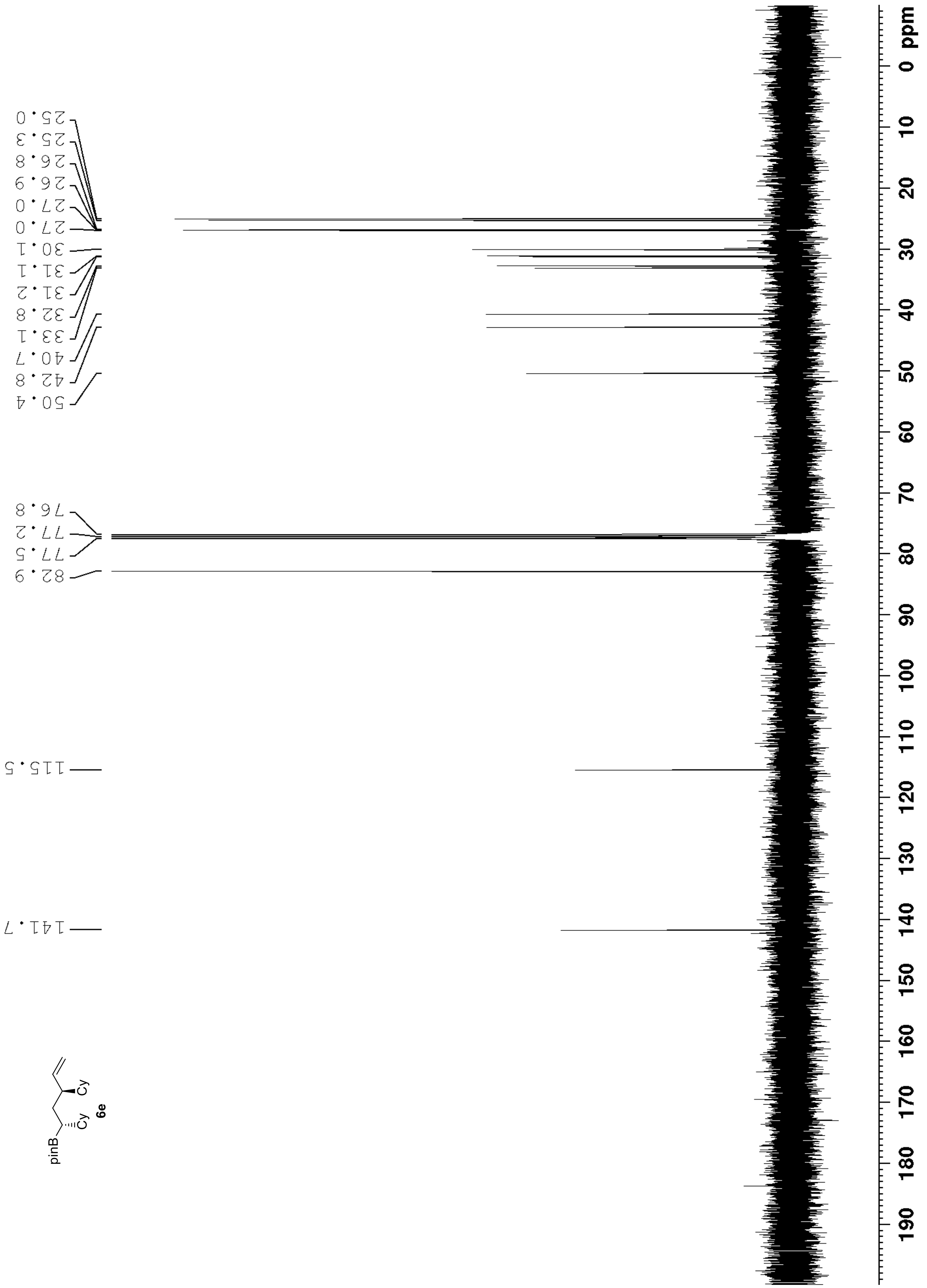
25.0
25.3
26.8
26.9
27.0
27.0
30.1
31.1
31.2
32.8
33.1
40.7
42.8
50.4
76.8
77.2
77.5
82.9
115.5
141.7
pinB
Cy
Cy
6e
ppm

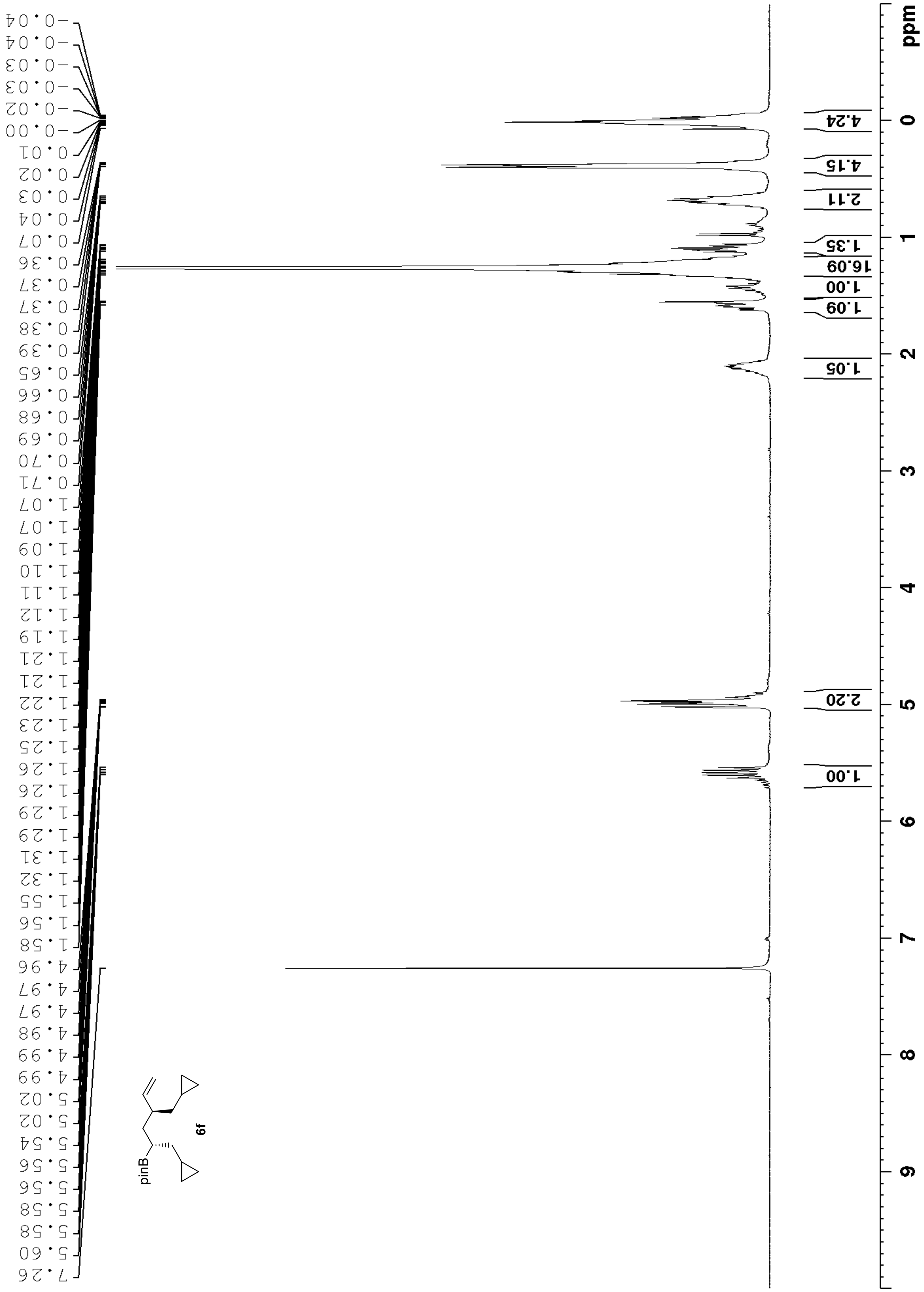
-0.04
-0.04
-0.03
-0.03
-0.02
-0.00
0.01
0.02
0.03
0.04
0.07
0.36
0.37
0.37
0.38
0.39
0.65
0.66
0.68
0.69
0.70
0.71
1.07
1.07
1.09
1.10
1.11
1.12
1.19
1.21
1.21
1.22
1.23
1.25
1.26
1.26
1.29
1.29
1.31
1.32
1.55
1.56
1.58
4.96
4.97
4.97
4.98
4.99
4.99
5.02
5.02
5.54
5.56
5.56
5.58
5.58
5.60
7.26
4.24
4.15
2.11
1.35
16.09
1.00
1.09
1.05
2.20
1.00
ppm
0
1
2
3
4
5
6
7
8
9
pinB
6f

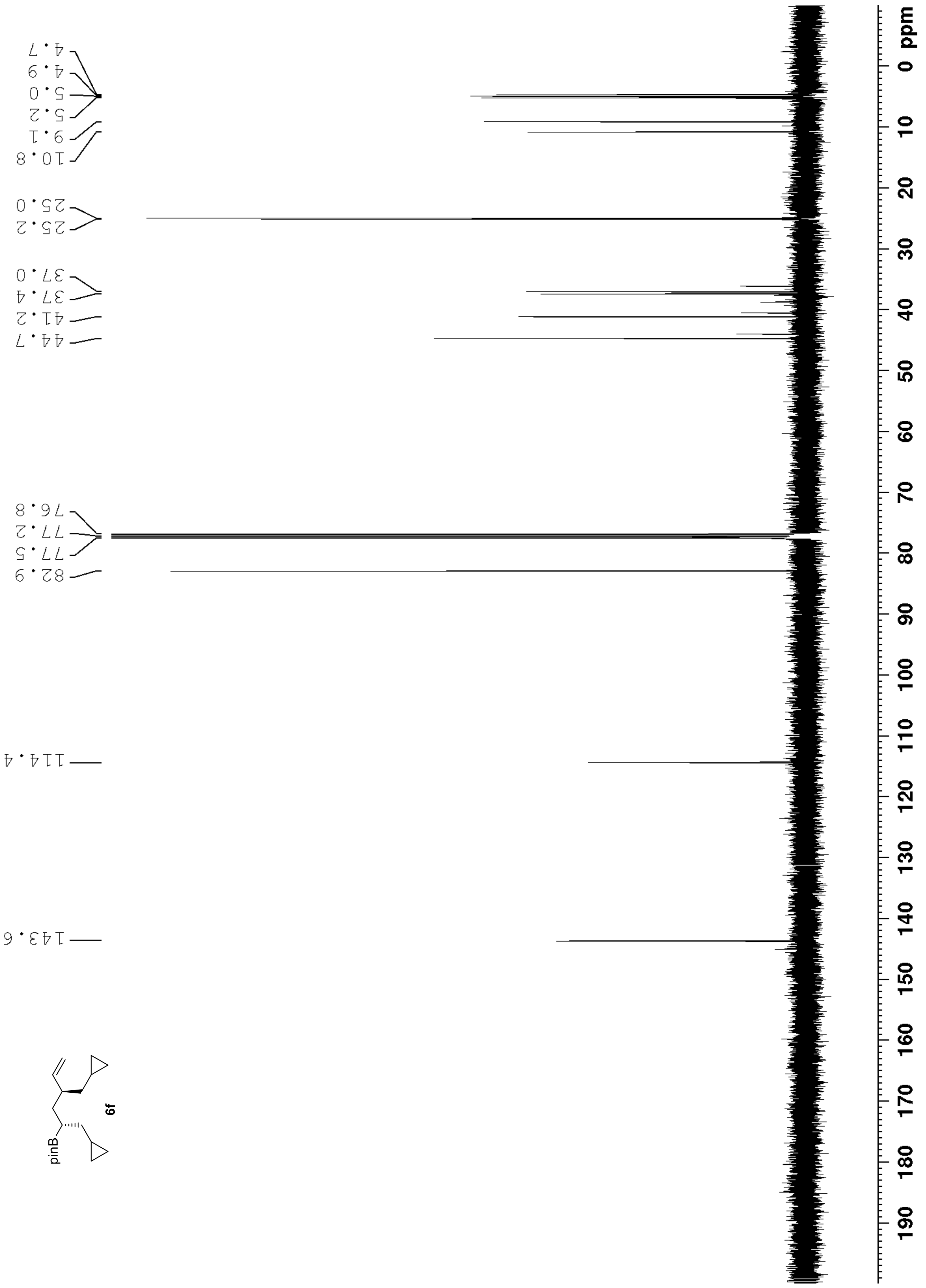
4.7
4.9
5.0
5.2
9.1
10.8
25.0
25.2
37.0
37.4
41.2
44.7
76.8
77.2
77.5
82.9
114.4
143.6
pinB
6f
ppm

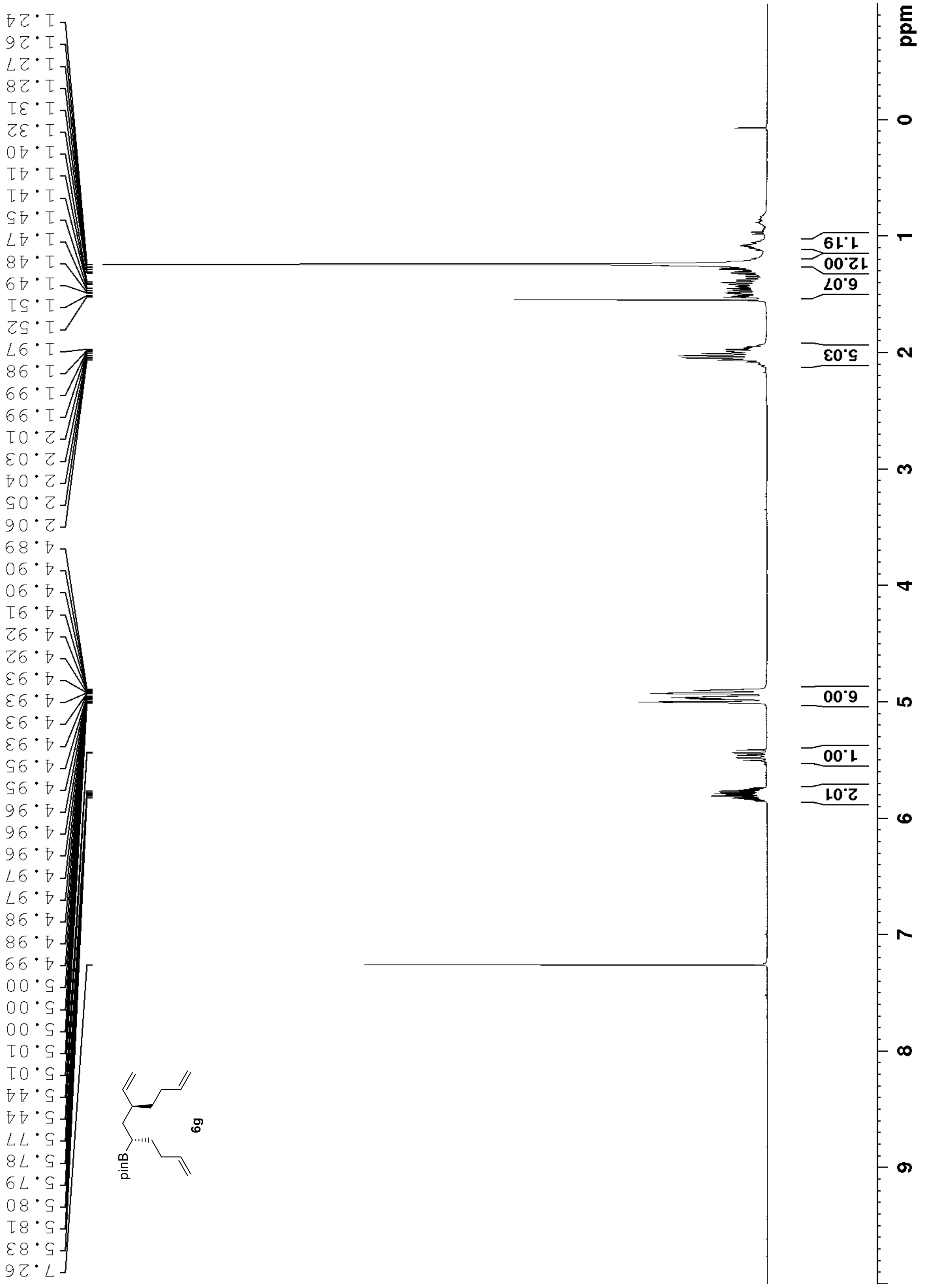
1.24
1.26
1.27
1.28
1.31
1.32
1.40
1.41
1.41
1.45
1.47
1.48
1.49
1.51
1.52
1.97
1.98
1.99
1.99
2.01
2.03
2.04
2.05
2.06
4.89
4.90
4.90
4.91
4.92
4.92
4.93
4.93
4.93
4.93
4.95
4.95
4.96
4.96
4.96
4.97
4.97
4.98
4.98
4.99
5.00
5.00
5.00
5.01
5.01
5.44
5.44
5.77
5.78
5.79
5.80
5.81
5.83
7.26
pinB
6g
1.19
12.00
6.07
5.03
6.00
1.00
2.01
ppm
0
1
2
3
4
5
6
7
8
9

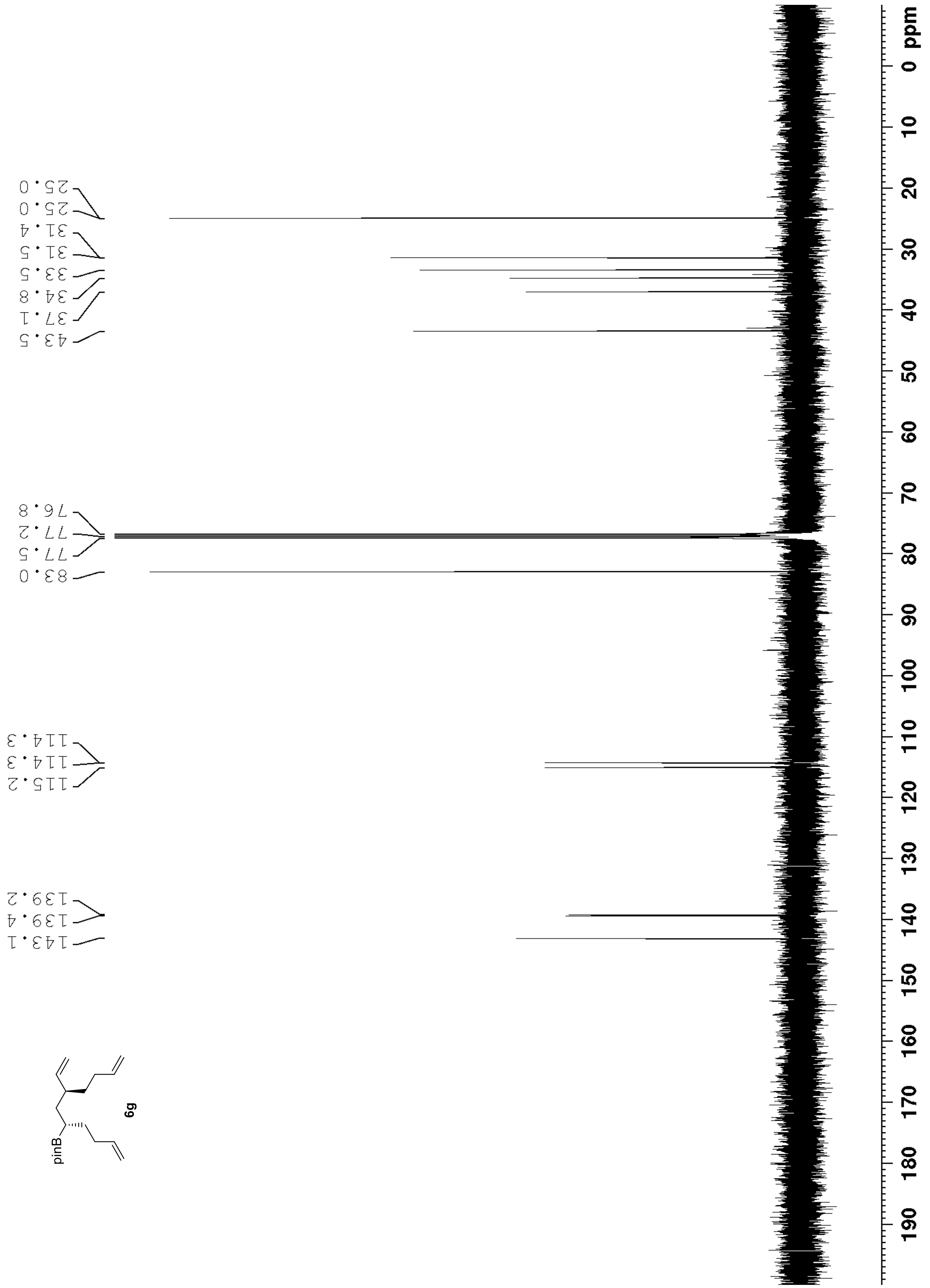
25.0
25.0
31.4
31.5
33.5
34.8
37.1
43.5
76.8
77.2
77.5
83.0
114.3
114.3
115.2
139.2
139.4
143.1
pinB
6g
ppm

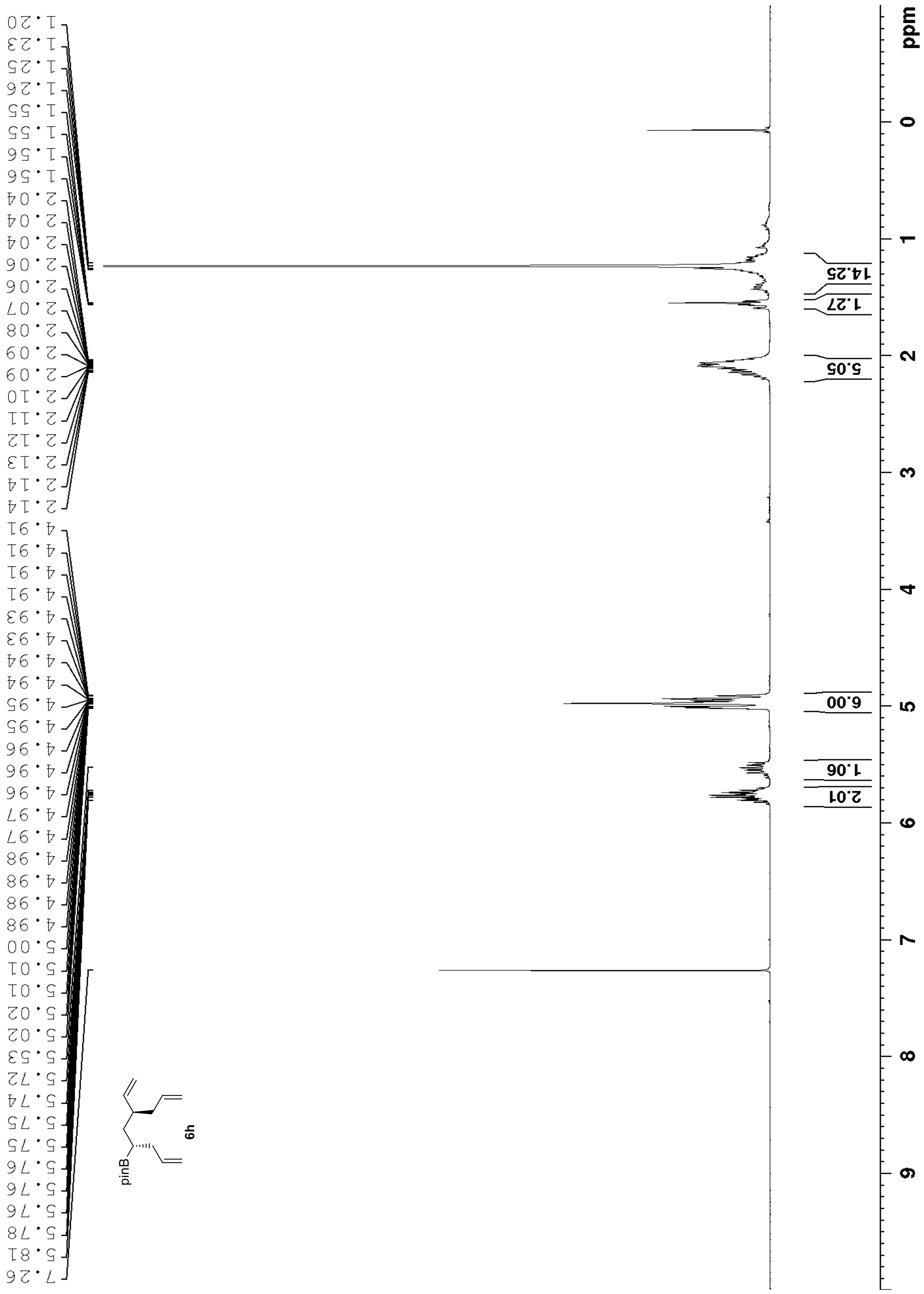
1.20
1.23
1.25
1.26
1.55
1.55
1.56
1.56
2.04
2.04
2.04
2.06
2.06
2.07
2.08
2.09
2.09
2.10
2.11
2.12
2.13
2.14
2.14
4.91
4.91
4.91
4.91
4.93
4.93
4.94
4.94
4.95
4.95
4.96
4.96
4.96
4.97
4.97
4.98
4.98
4.98
4.98
5.00
5.01
5.01
5.02
5.02
5.53
5.72
5.74
5.75
5.75
5.76
5.76
5.76
5.78
5.81
7.26
14.25
1.27
5.05
6.00
1.06
2.01
ppm
0
1
2
3
4
5
6
7
8
9
pinB
6h

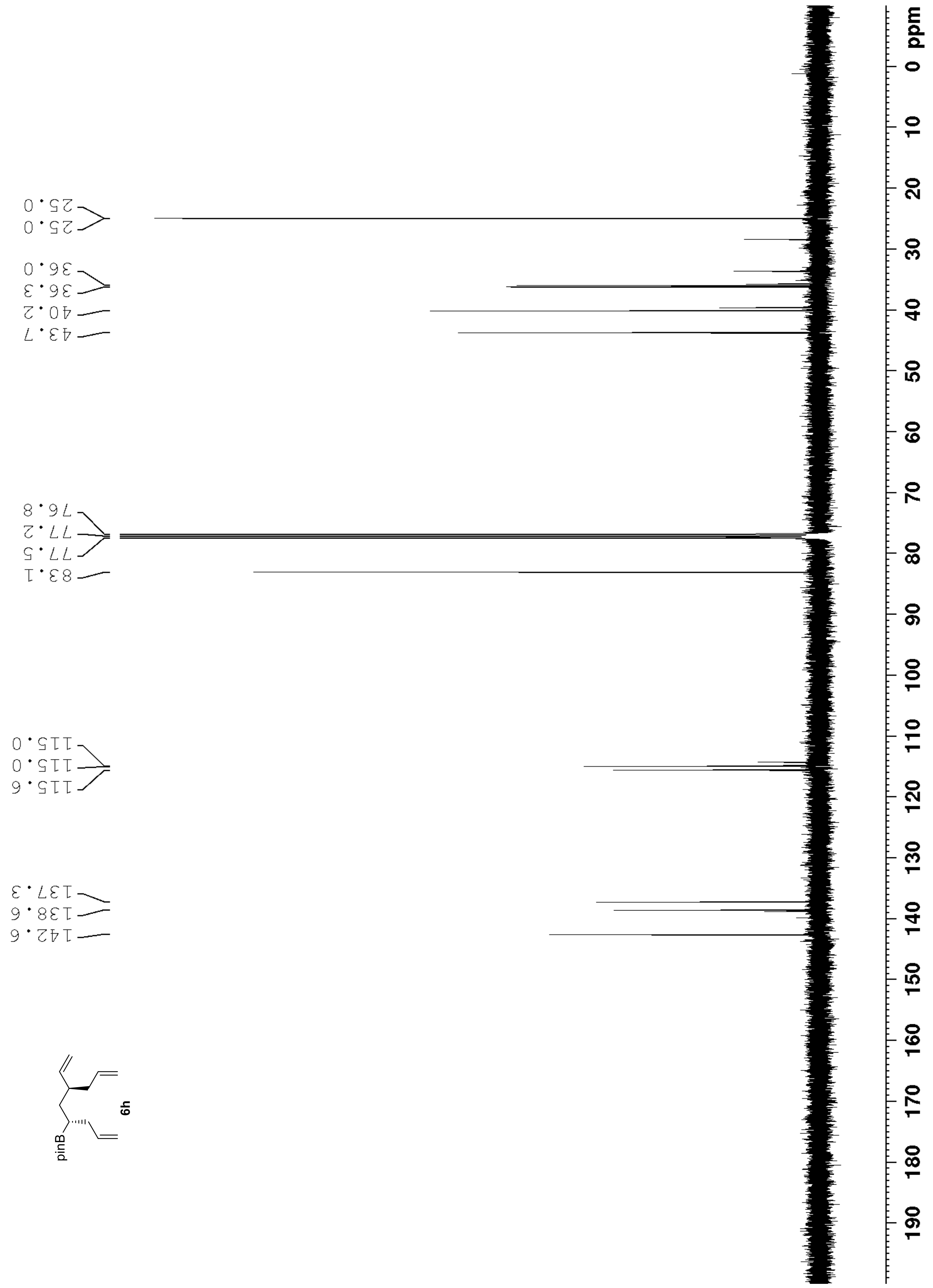
25.0
25.0
36.0
36.3
40.2
43.7
76.8
77.2
77.5
83.1
115.0
115.0
115.6
137.3
138.6
142.6
pinB
6h
ppm
0
10
20
30
40
50
60
70
80
90
100
110
120
130
140
150
160
170
180
190

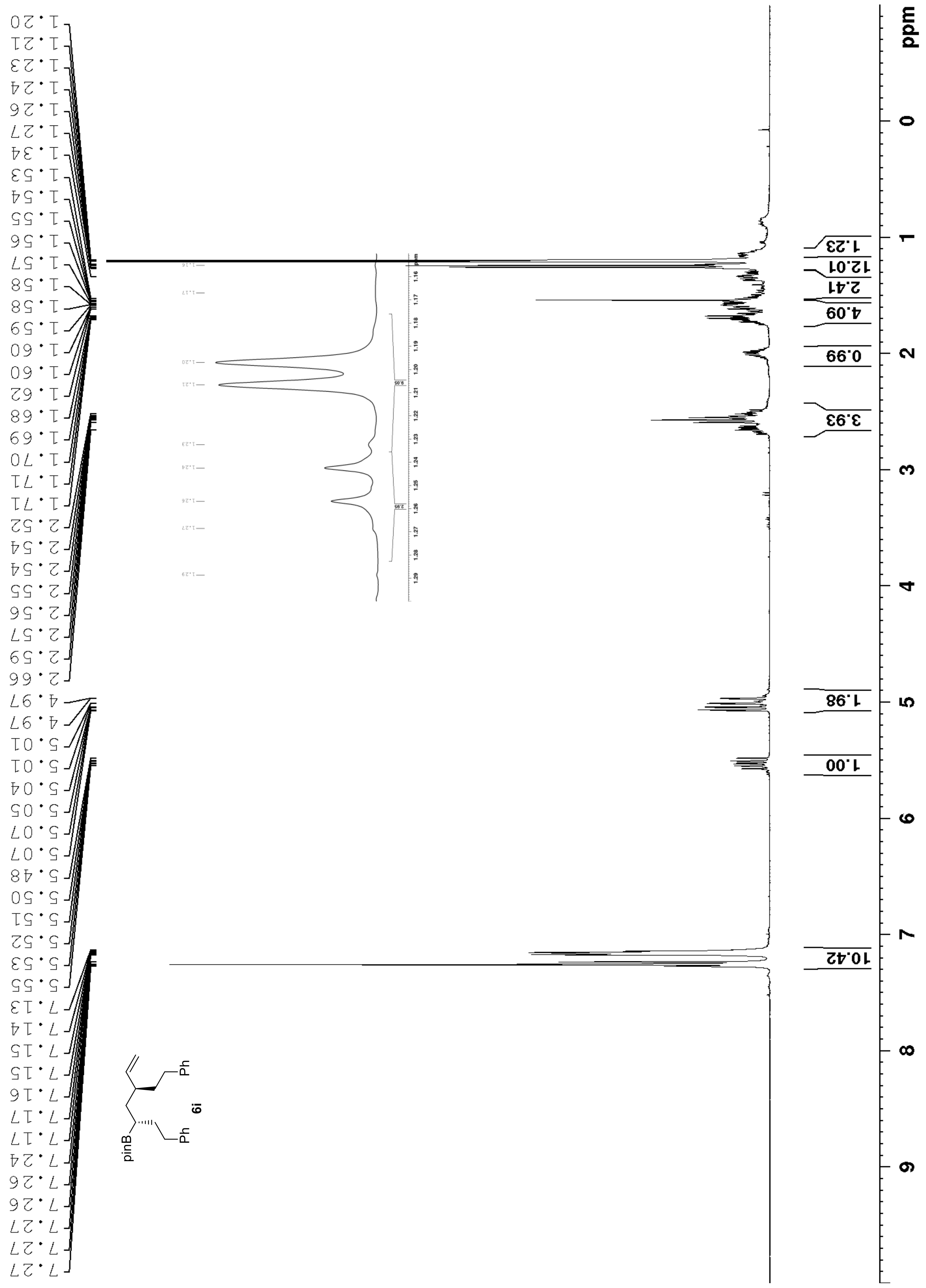
ppm
pinB
Ph
Ph
6i

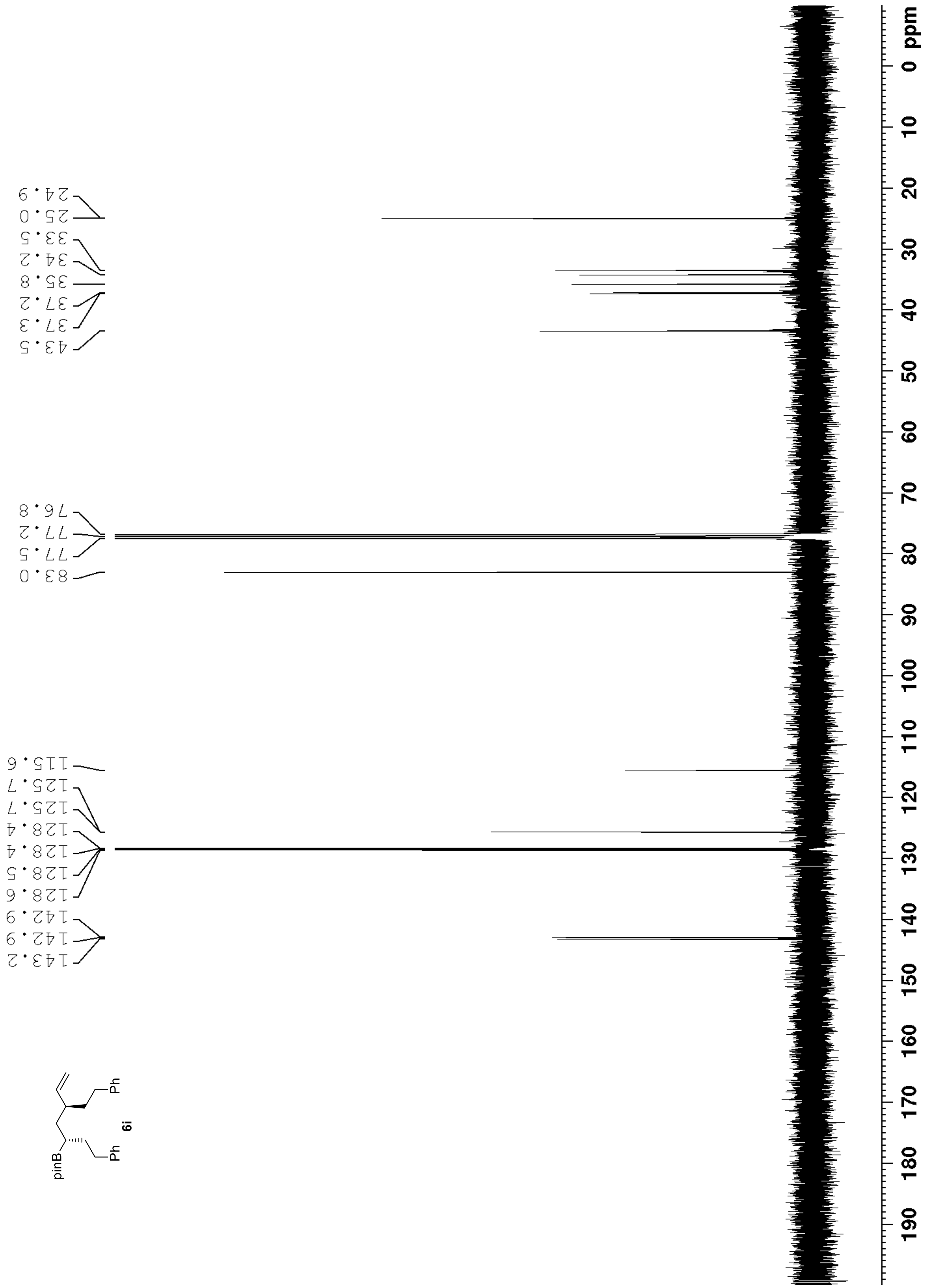
24.9
25.0
33.5
34.2
35.8
37.2
37.3
43.5
76.8
77.2
77.5
83.0
115.6
125.7
125.7
128.4
128.4
128.5
128.6
142.9
142.9
143.2
pinB
Ph
Ph
6i
ppm

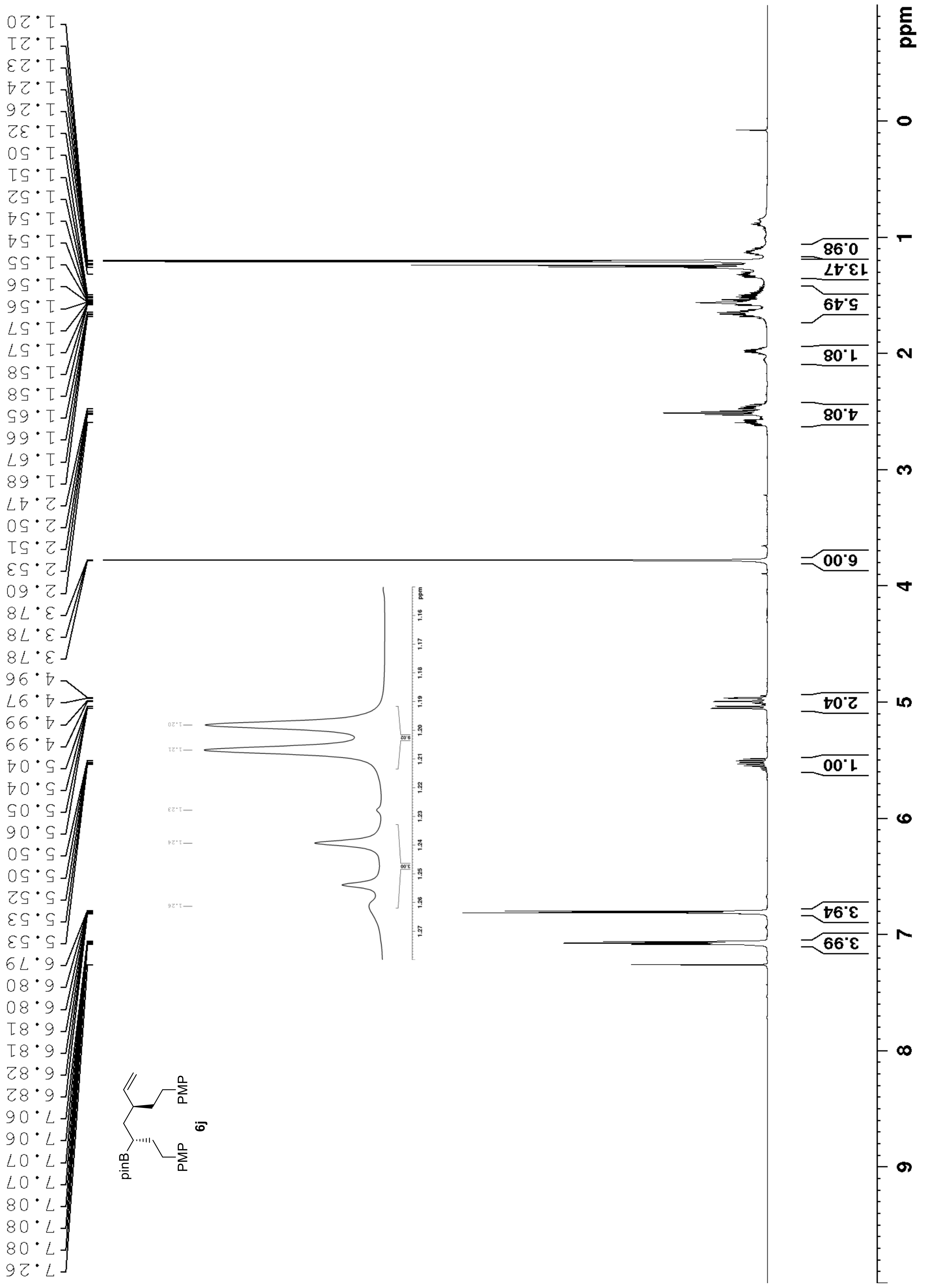
1.20
1.21
1.23
1.24
1.26
1.32
1.50
1.51
1.52
1.54
1.54
1.55
1.56
1.56
1.57
1.57
1.58
1.58
1.65
1.66
1.67
1.68
2.47
2.50
2.51
2.53
2.60
3.78
3.78
3.78
4.96
4.97
4.99
4.99
5.04
5.04
5.05
5.06
5.50
5.50
5.52
5.53
5.53
6.79
6.80
6.80
6.81
6.81
6.82
6.82
7.06
7.06
7.07
7.07
7.08
7.08
7.08
7.08
7.26
0.98
13.47
5.49
1.08
4.08
6.00
2.04
1.00
3.94
3.99
ppm
pinB
PMP
PMP
6j

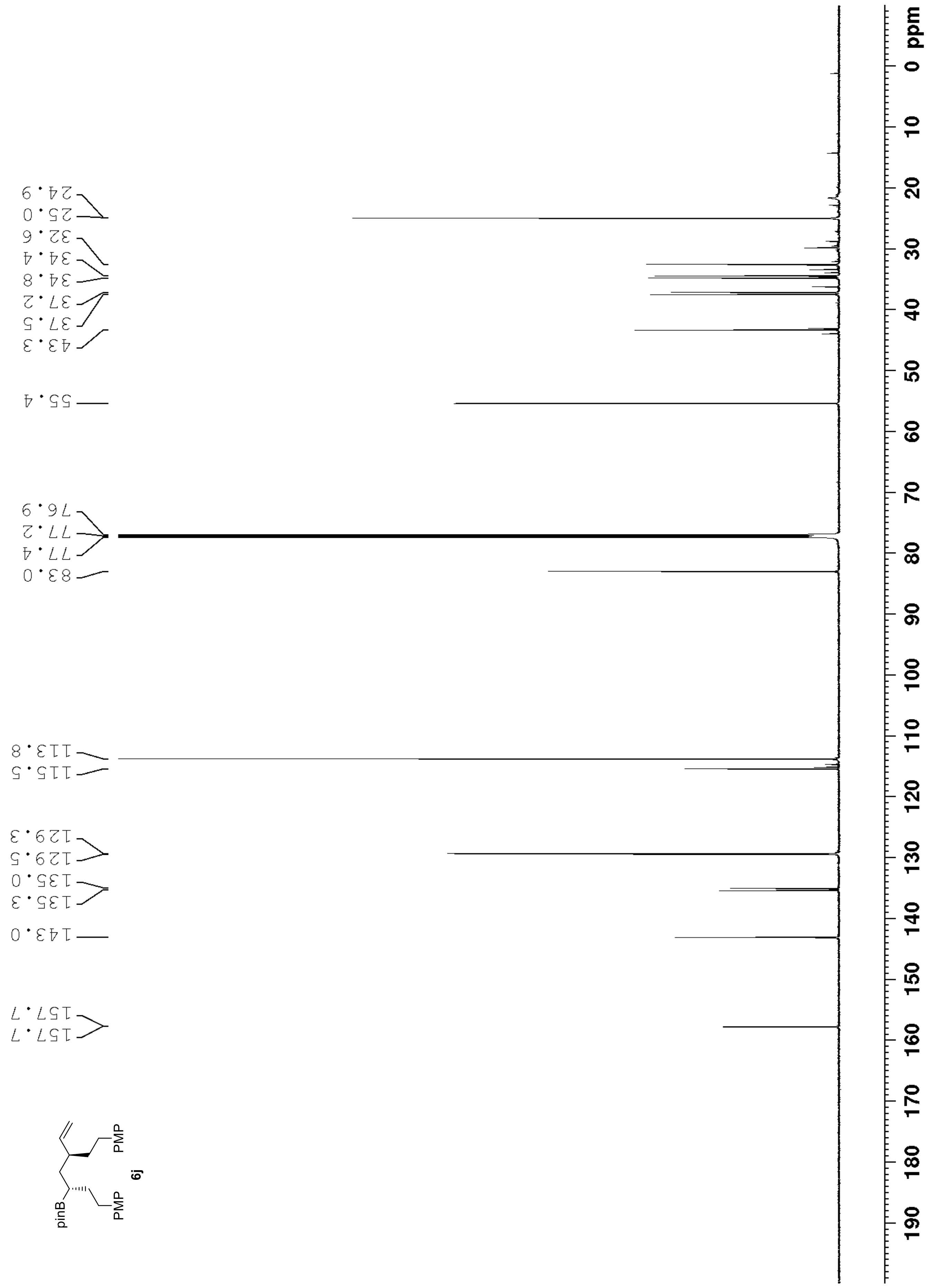
24.9
25.0
32.6
34.4
34.8
37.2
37.5
43.3
55.4
76.9
77.2
77.4
83.0
113.8
115.5
129.3
129.5
135.0
135.3
143.0
157.7
157.7
pinB
PMP
PMP
6j
ppm
0
10
20
30
40
50
60
70
80
90
100
110
120
130
140
150
160
170
180
190

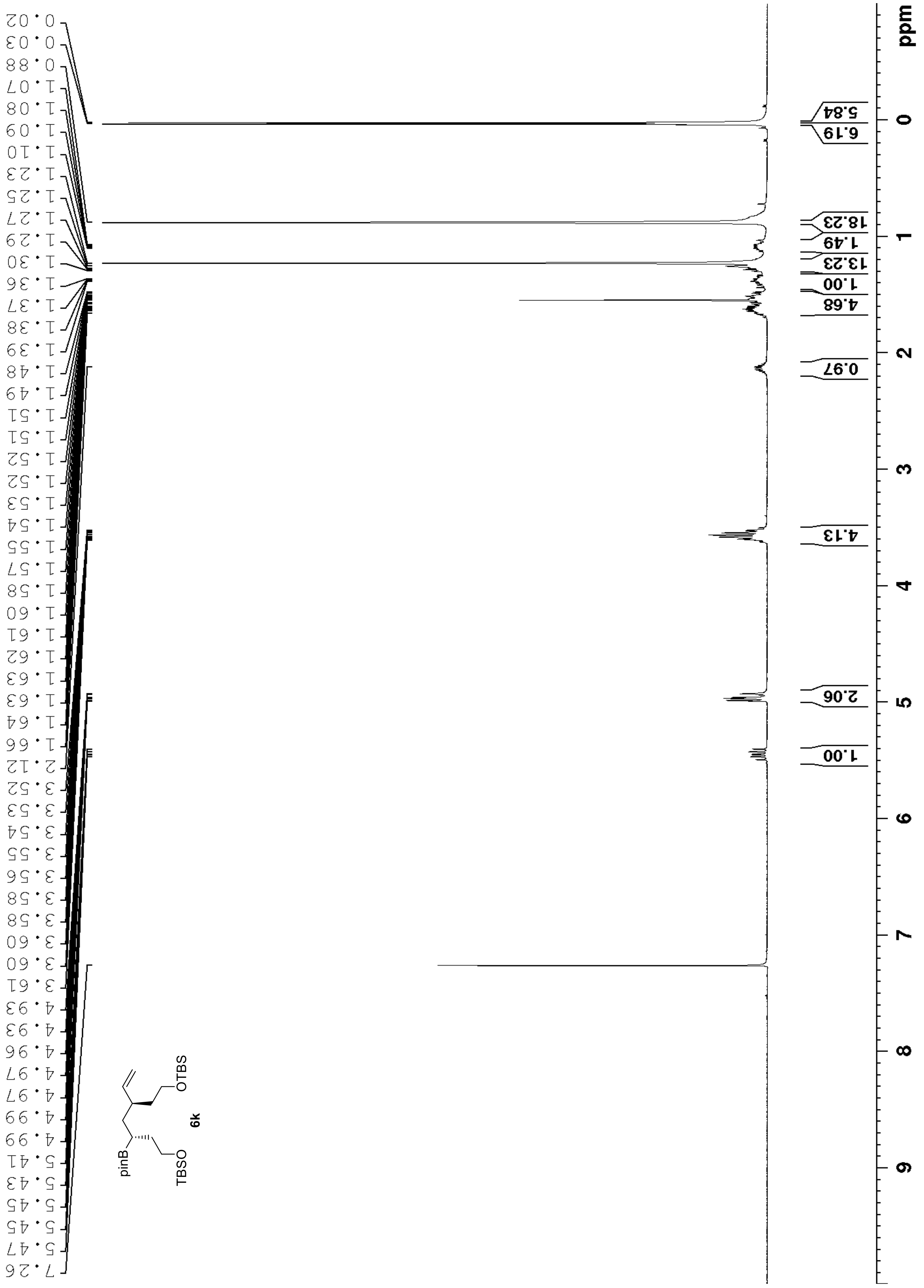
0.02
0.03
0.88
1.07
1.08
1.09
1.10
1.23
1.25
1.27
1.29
1.30
1.36
1.37
1.38
1.39
1.48
1.49
1.51
1.51
1.52
1.52
1.53
1.54
1.55
1.57
1.58
1.60
1.61
1.62
1.63
1.63
1.64
1.66
2.12
3.52
3.53
3.54
3.55
3.56
3.58
3.58
3.60
3.60
3.61
4.93
4.93
4.96
4.97
4.97
4.99
4.99
5.41
5.43
5.45
5.45
5.47
7.26
pinB
OTBS
TBSO
6k
5.84
6.19
18.23
1.49
13.23
1.00
4.68
0.97
4.13
2.06
1.00
ppm
0
1
2
3
4
5
6
7
8
9

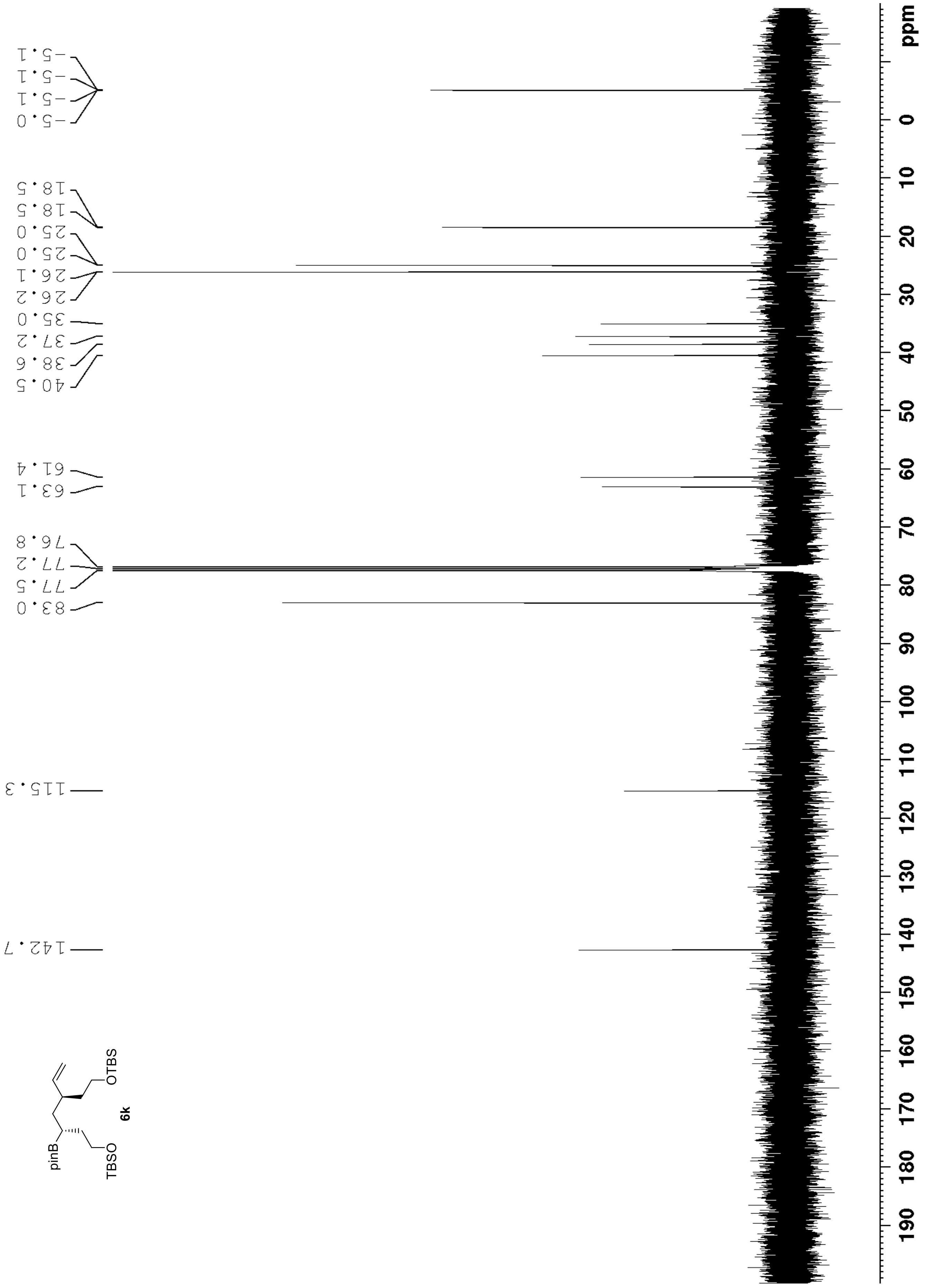
-5.1
-5.1
-5.1
-5.0
18.5
18.5
25.0
25.0
26.1
26.2
35.0
37.2
38.6
40.5
61.4
63.1
76.8
77.2
77.5
83.0
115.3
142.7
pinB
TBSO
OTBS
6k
ppm
0
10
20
30
40
50
60
70
80
90
100
110
120
130
140
150
160
170
180
190

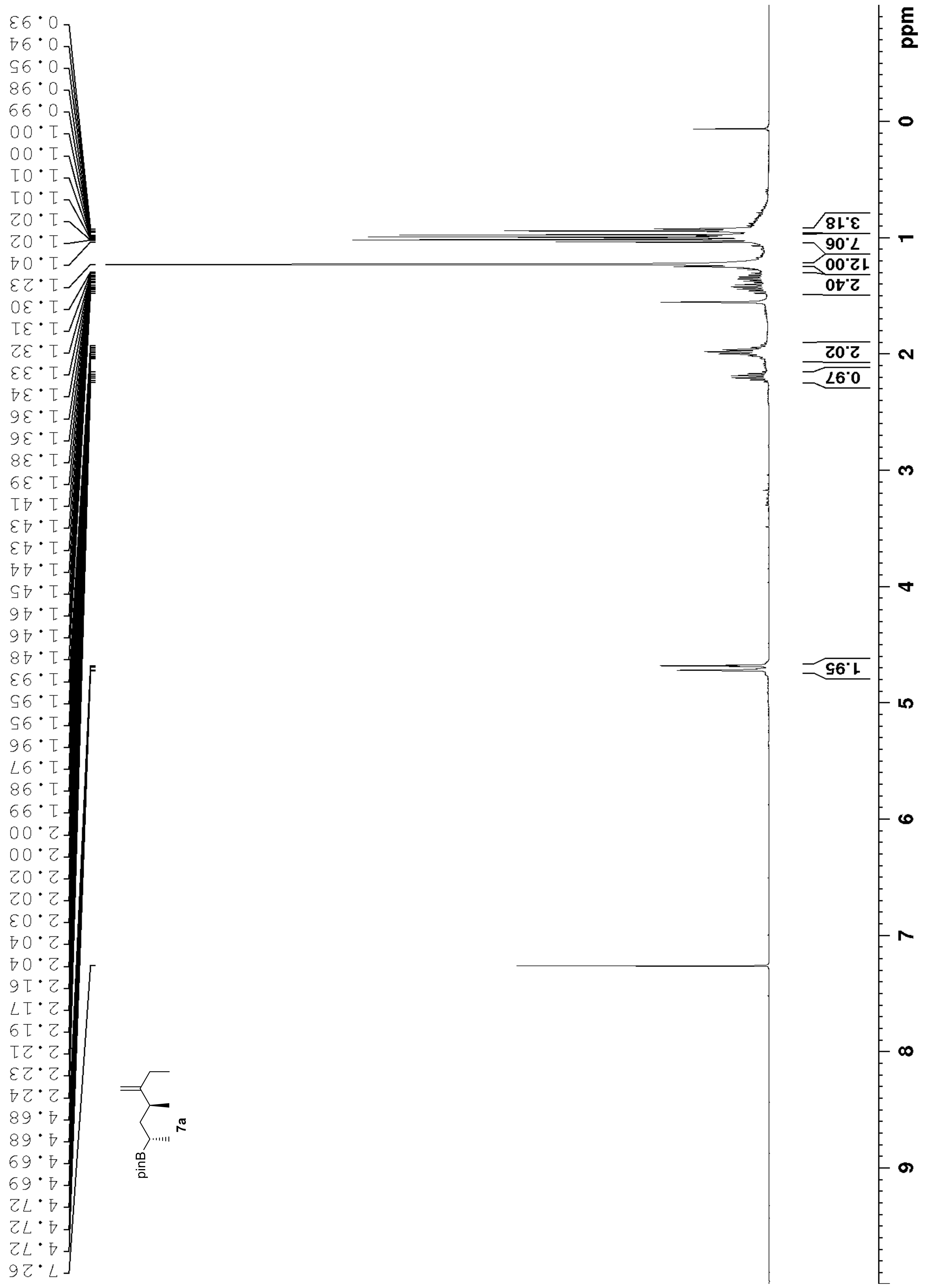
0.93
0.94
0.95
0.98
0.99
1.00
1.00
1.01
1.01
1.02
1.02
1.04
1.23
1.30
1.31
1.32
1.33
1.34
1.36
1.36
1.38
1.39
1.41
1.43
1.43
1.44
1.45
1.46
1.46
1.48
1.93
1.95
1.95
1.96
1.97
1.98
1.99
2.00
2.00
2.02
2.02
2.03
2.04
2.04
2.16
2.17
2.19
2.21
2.23
2.24
4.68
4.68
4.69
4.69
4.72
4.72
4.72
7.26
3.18
7.06
12.00
2.40
2.02
0.97
1.95
pinB
7a
ppm

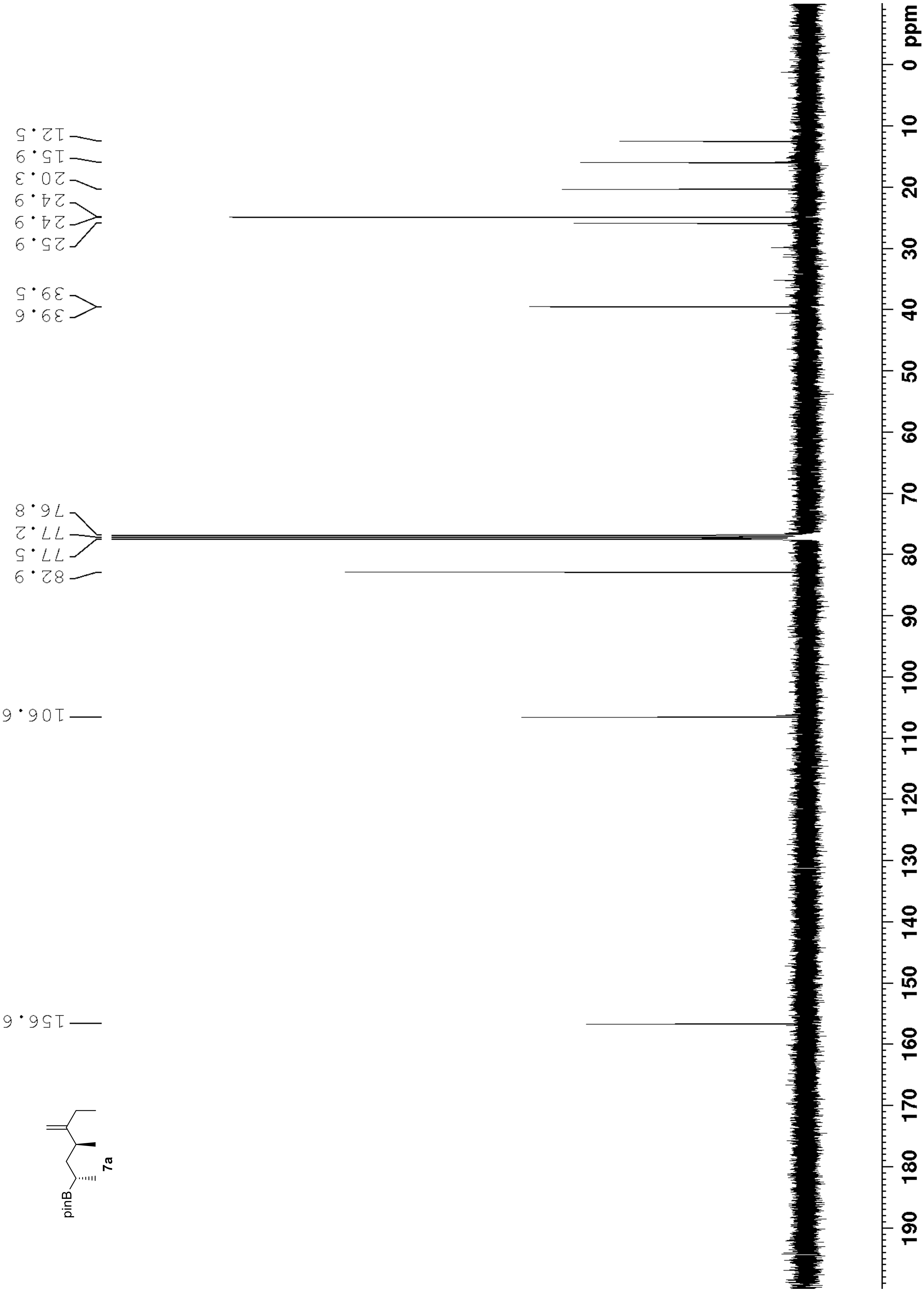
12.5
15.9
20.3
24.9
24.9
25.9
39.5
39.6
76.8
77.2
77.5
82.9
106.6
156.6
pinB
7a
0 10 20 30 40 50 60 70 80 90 100 110 120 130 140 150 160 170 180 190 ppm

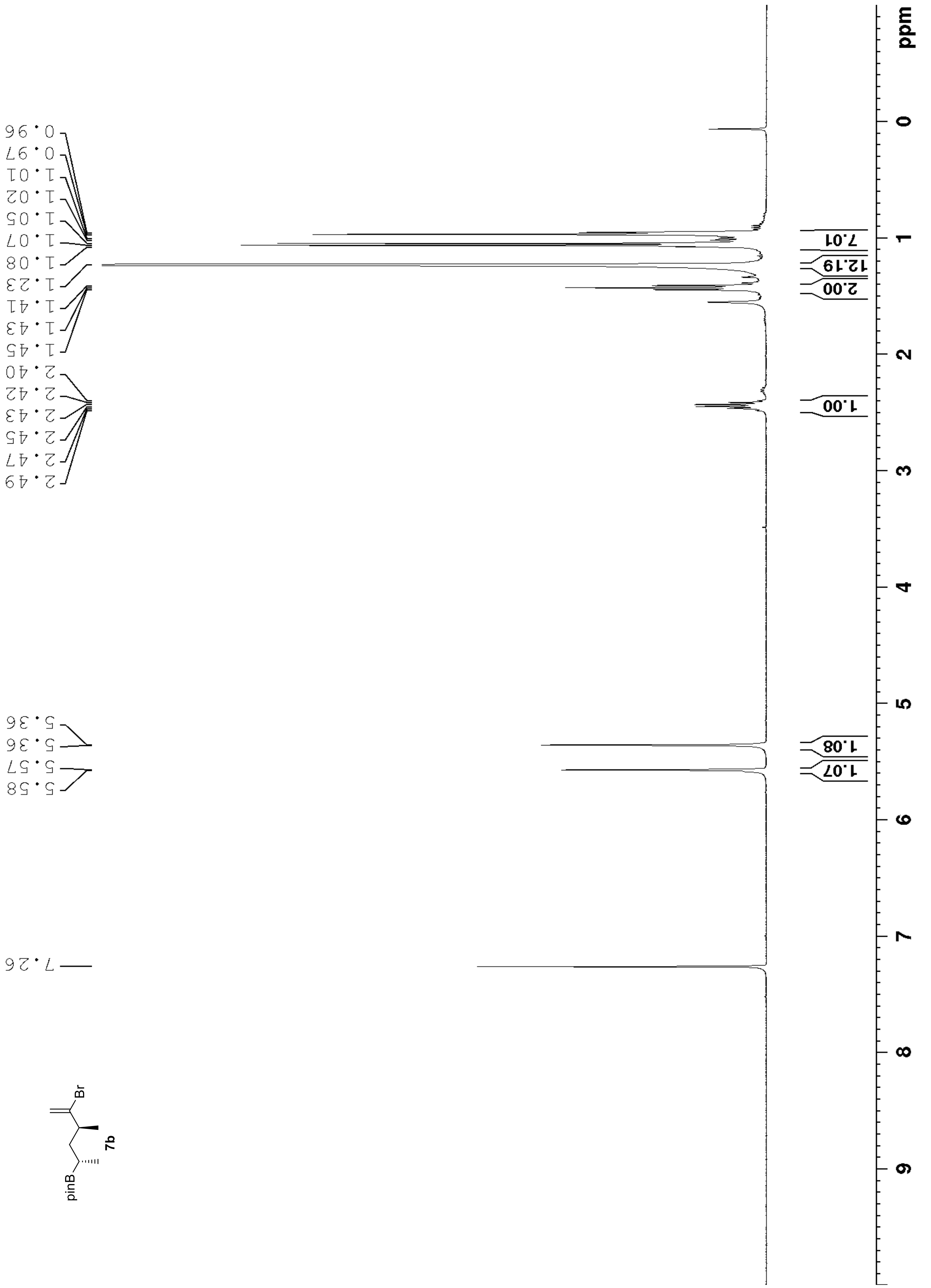
0.96
0.97
1.01
1.02
1.05
1.07
1.08
1.23
1.41
1.43
1.45
2.40
2.42
2.43
2.45
2.47
2.49
5.36
5.36
5.57
5.58
7.26
7.01
12.19
2.00
1.00
1.08
1.07
ppm
0
1
2
3
4
5
6
7
8
9
Br
pinB
7b

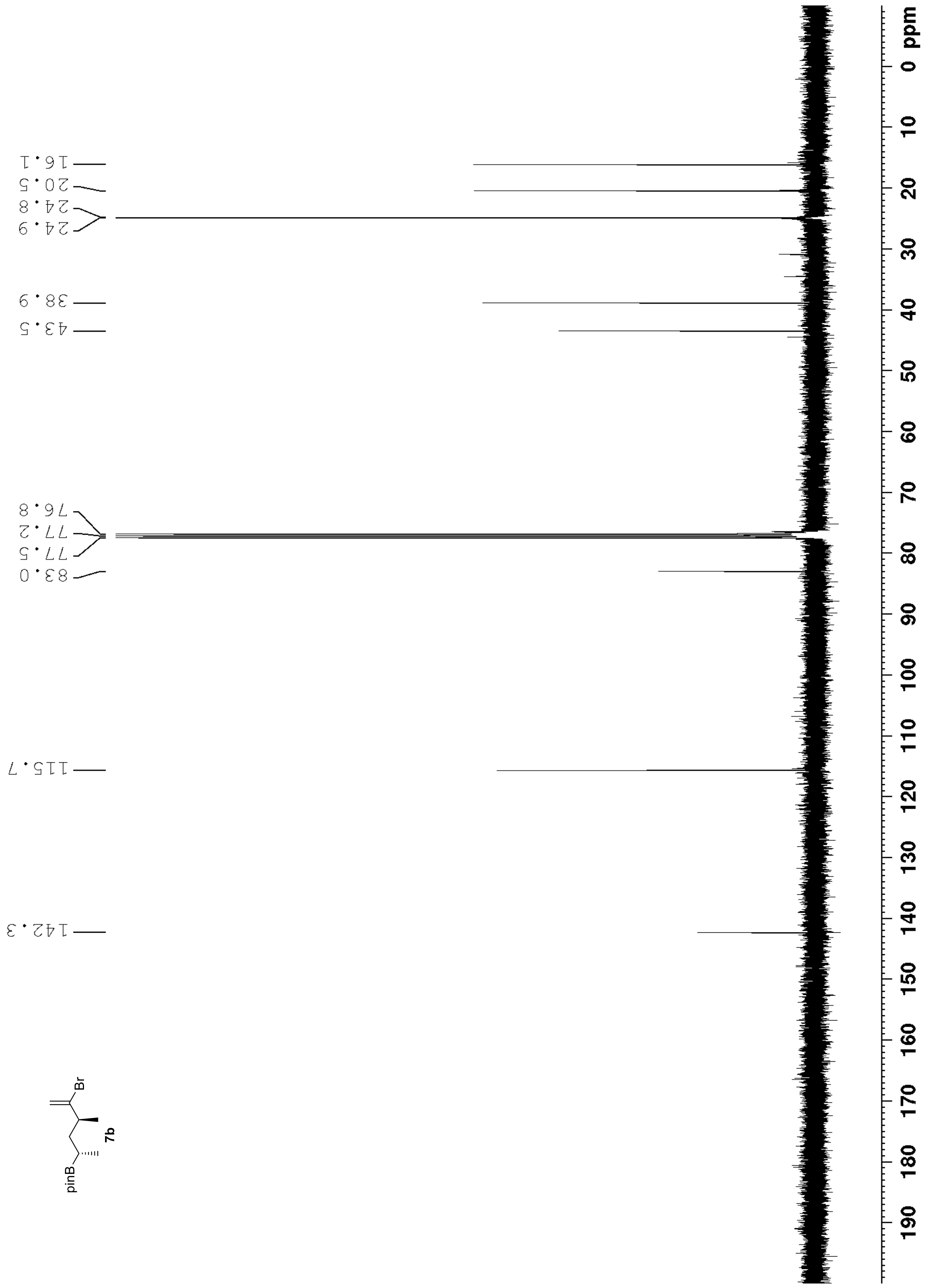

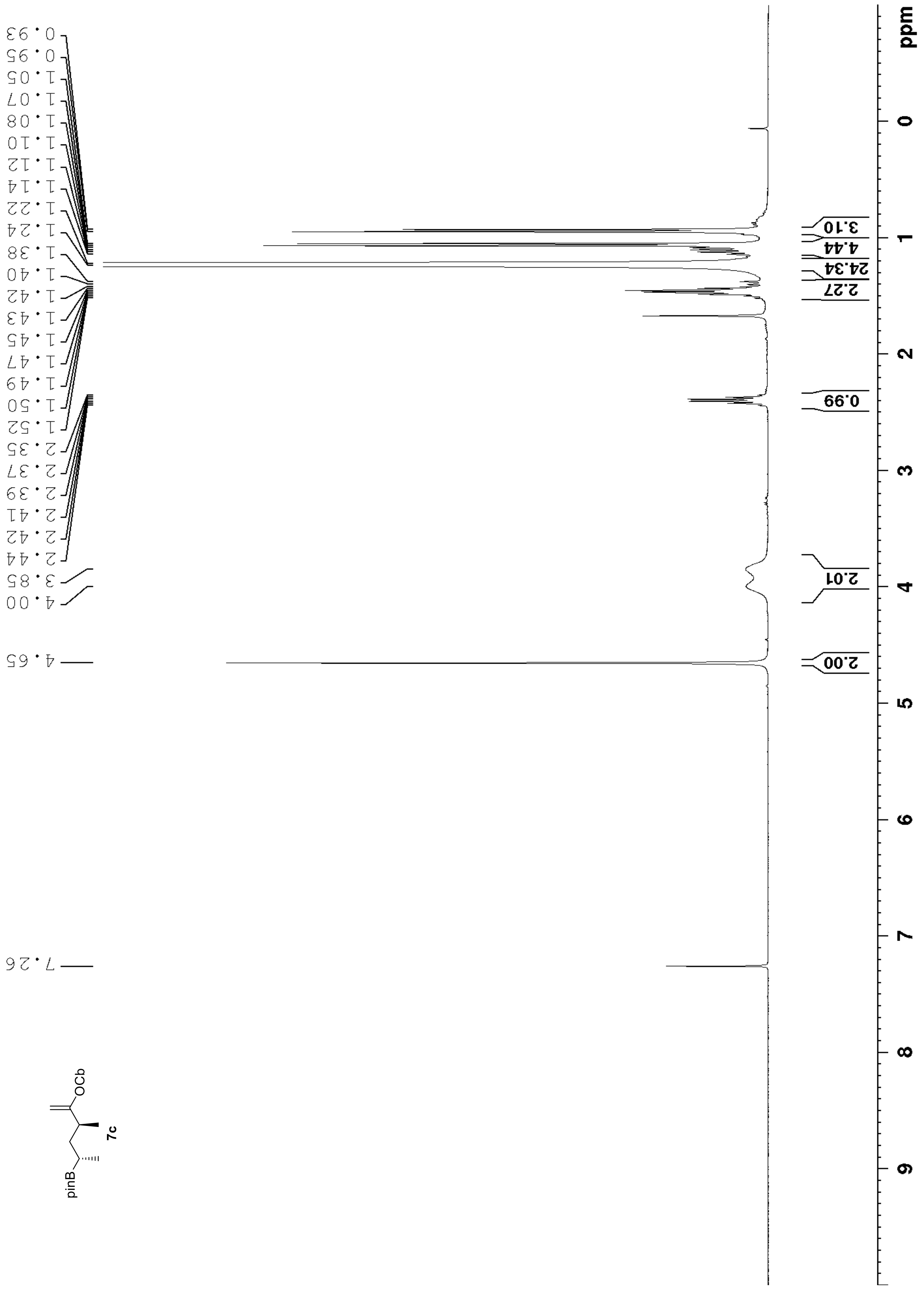
0.93
0.95
1.05
1.07
1.08
1.10
1.12
1.14
1.22
1.24
1.38
1.40
1.42
1.43
1.45
1.47
1.49
1.50
1.52
2.35
2.37
2.39
2.41
2.42
2.44
3.85
4.00
4.65
7.26
3.10
4.44
24.34
2.27
0.99
2.01
2.00
ppm
0
1
2
3
4
5
6
7
8
9
OCb
pinB
7c

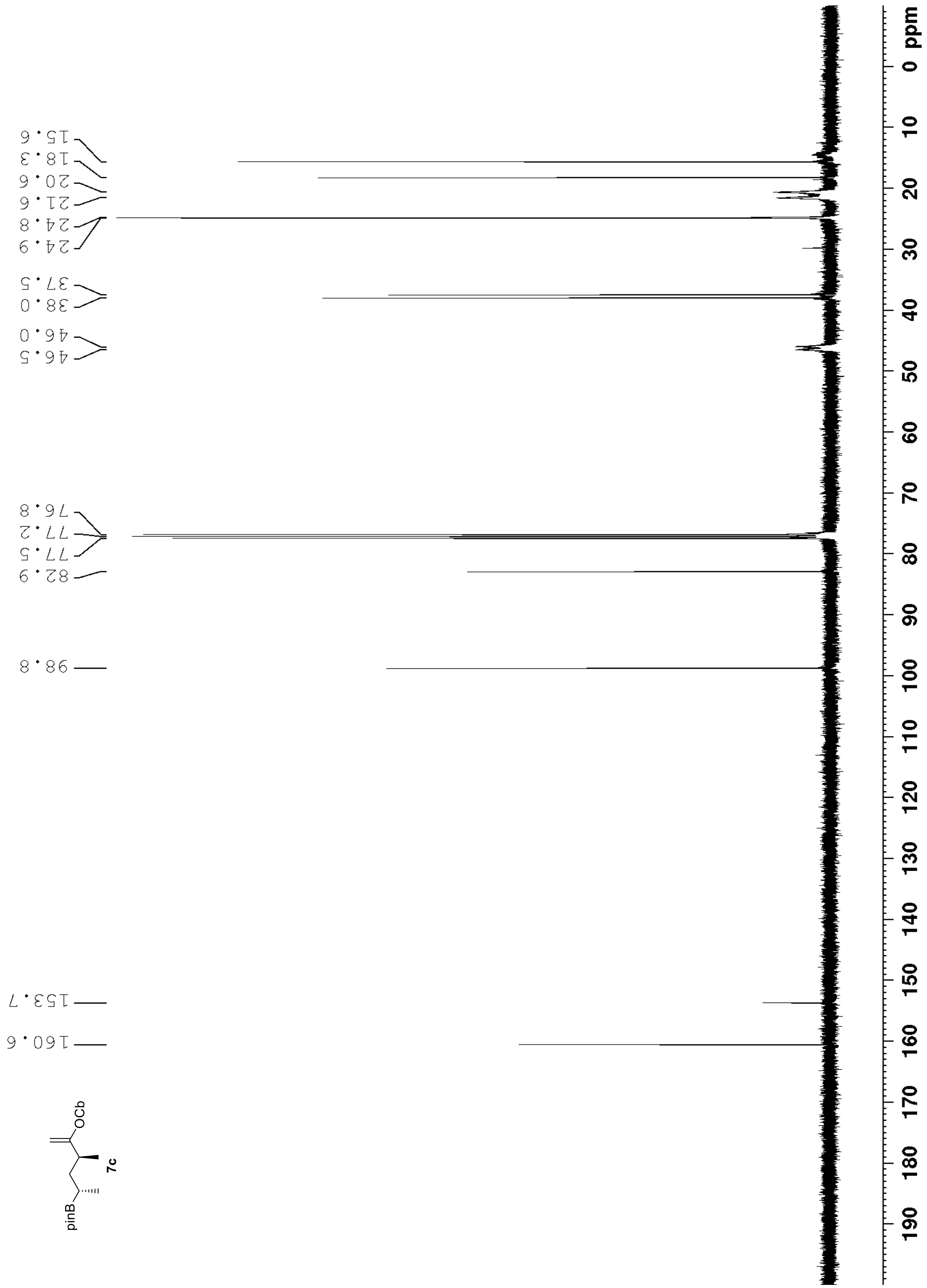
15.6
18.3
20.6
21.6
24.8
24.9
37.5
38.0
46.0
46.5
76.8
77.2
77.5
82.9
98.8
153.7
160.6
pinB
OCb
7c
ppm

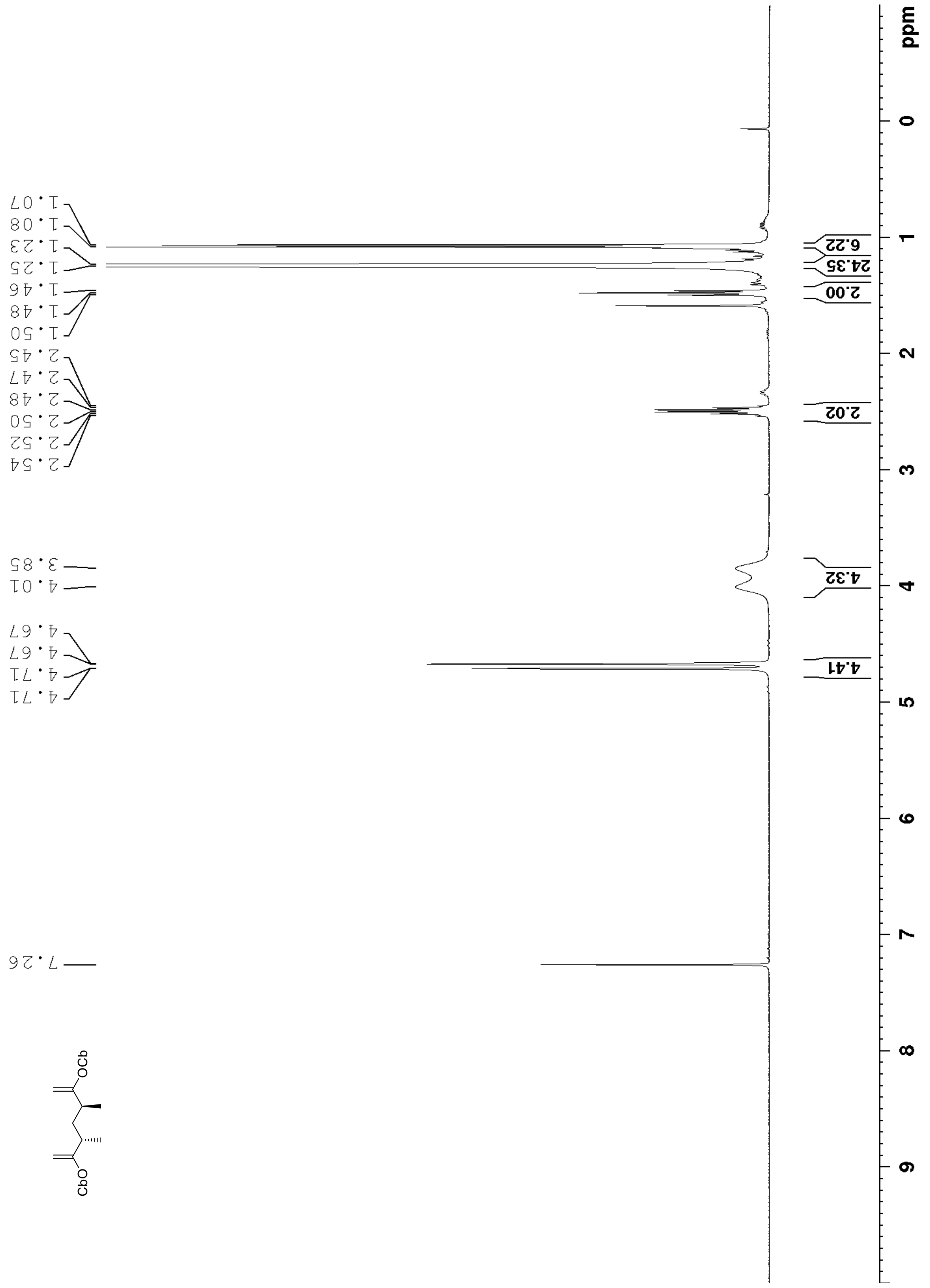
ppm
1.07
1.08
1.23
1.25
1.46
1.48
1.50
2.45
2.47
2.48
2.50
2.52
2.54
3.85
4.01
4.67
4.67
4.71
4.71
7.26
6.22
24.35
2.00
2.02
4.32
4.41
OCb
CbO

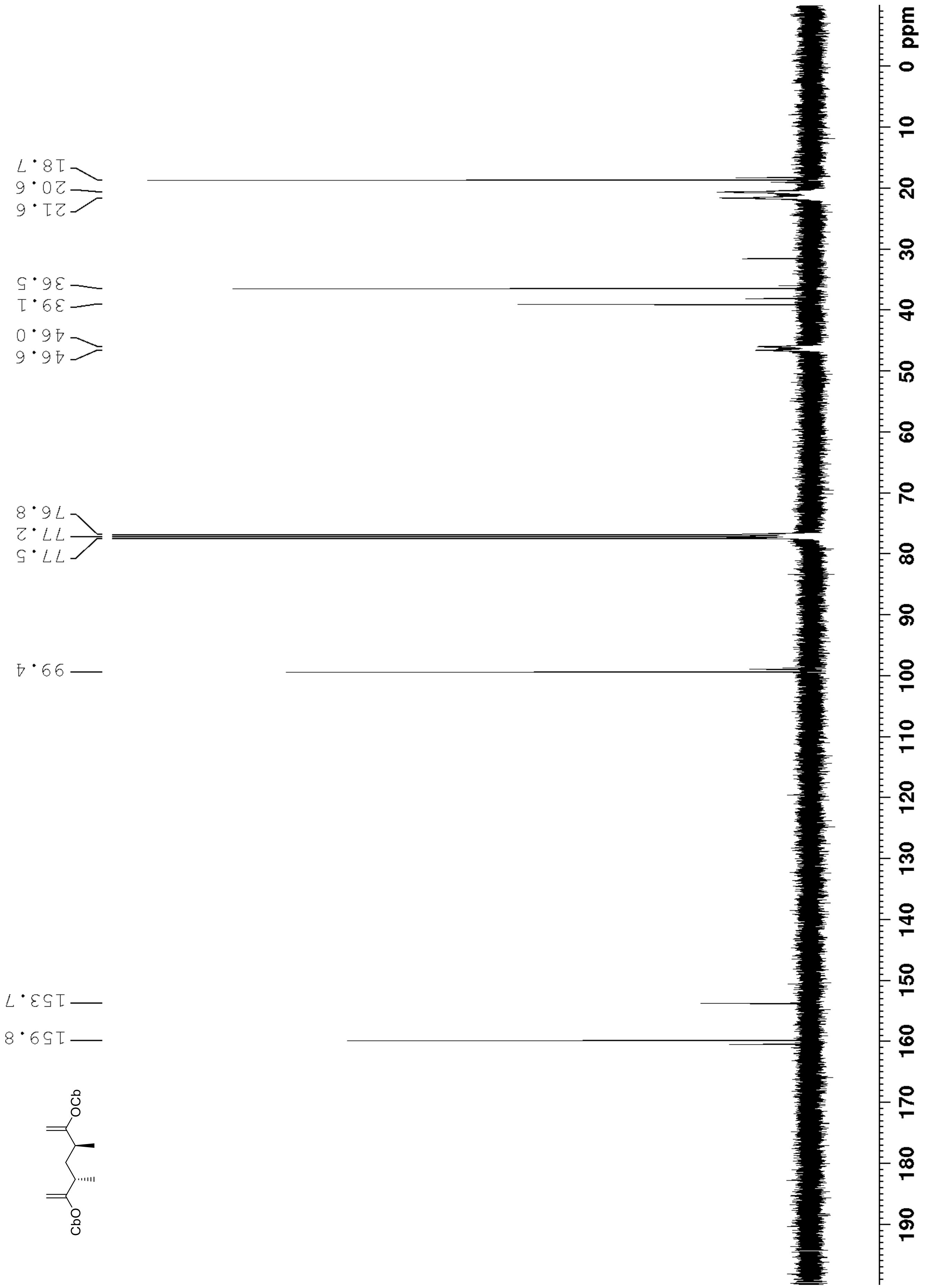
18.7
20.6
21.6
36.5
39.1
46.0
46.6
76.8
77.2
77.5
99.4
153.7
159.8
OCb
CbO
ppm

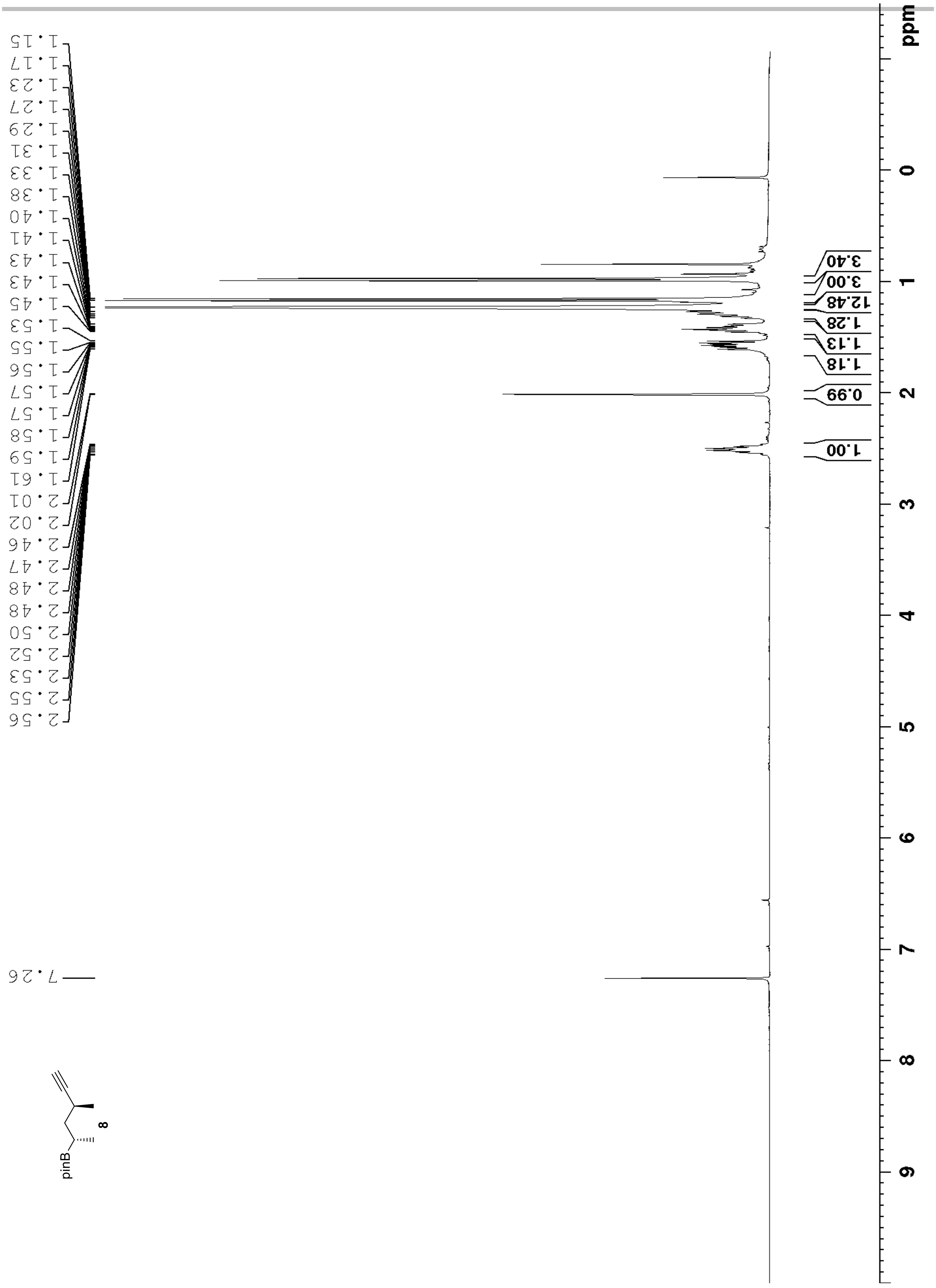
1.15
1.17
1.23
1.27
1.29
1.31
1.33
1.38
1.40
1.41
1.43
1.43
1.45
1.53
1.55
1.56
1.57
1.57
1.58
1.59
1.61
2.01
2.02
2.46
2.47
2.48
2.48
2.50
2.52
2.53
2.55
2.56
7.26
pinB
8
3.40
3.00
12.48
1.28
1.13
1.18
0.99
1.00
ppm
0
1
2
3
4
5
6
7
8
9

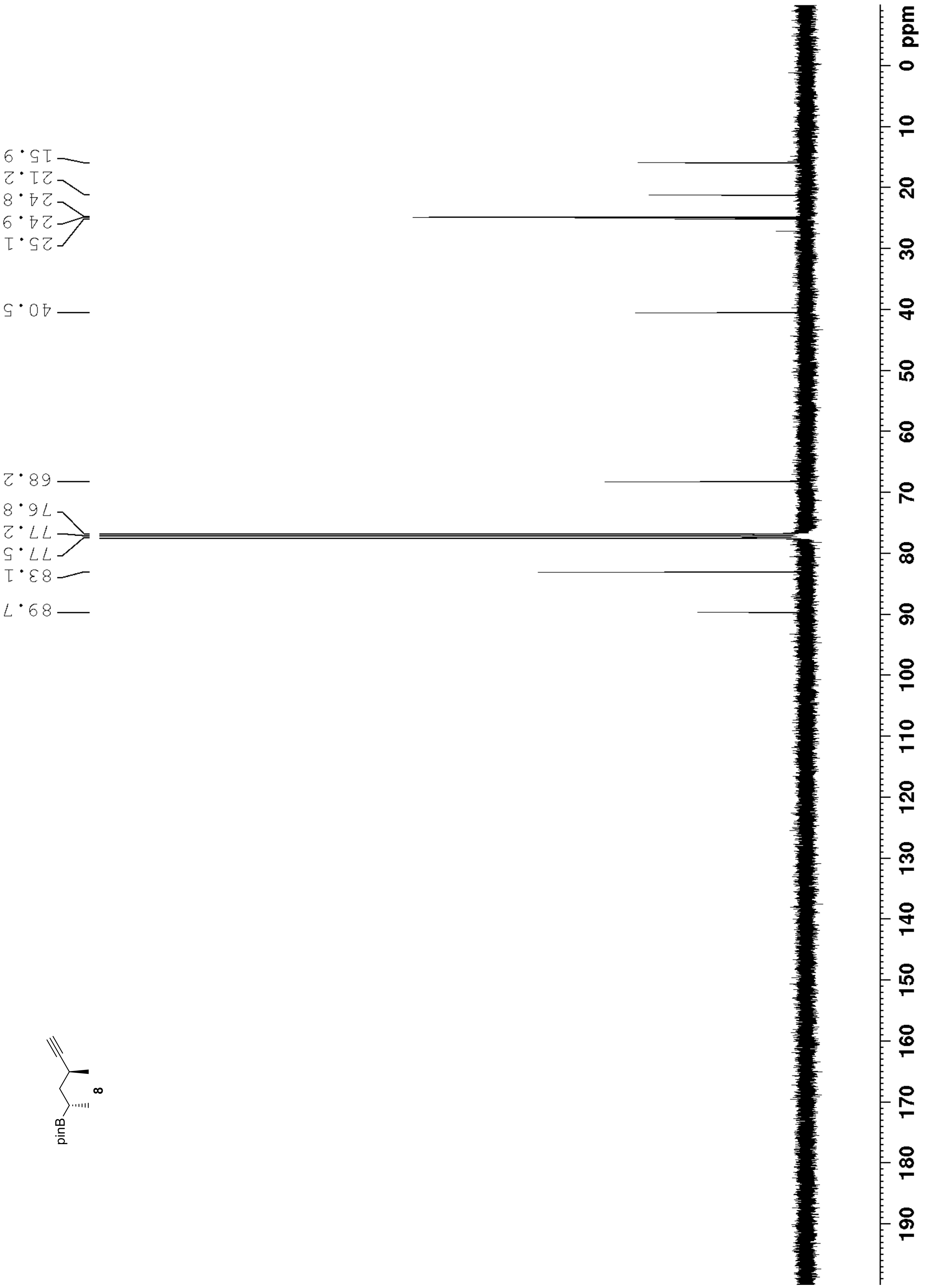
15.9
21.2
24.8
24.9
25.1
40.5
68.2
76.8
77.2
77.5
83.1
89.7
pinB
8
ppm
0
10
20
30
40
50
60
70
80
90
100
110
120
130
140
150
160
170
180
190

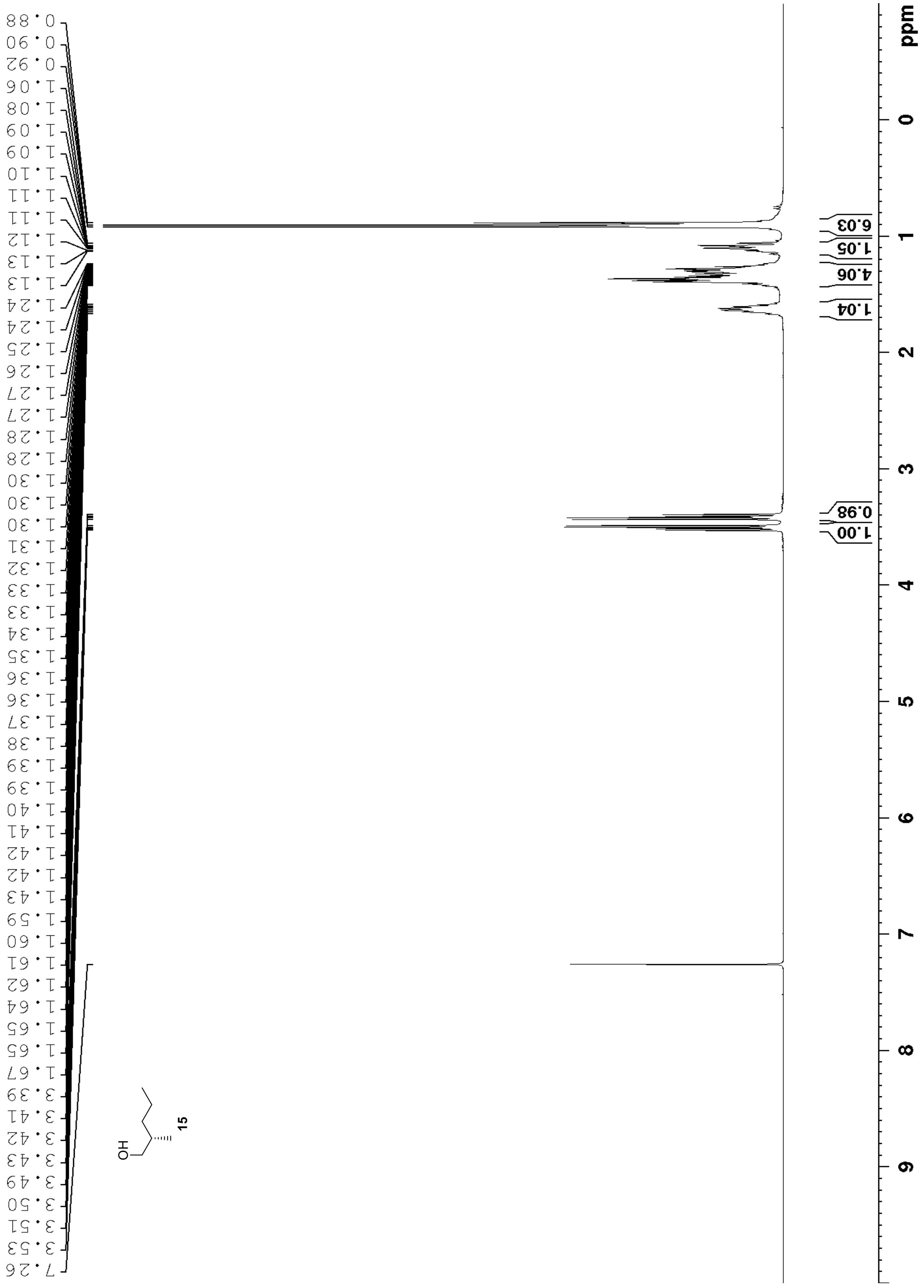
OH
15
ppm

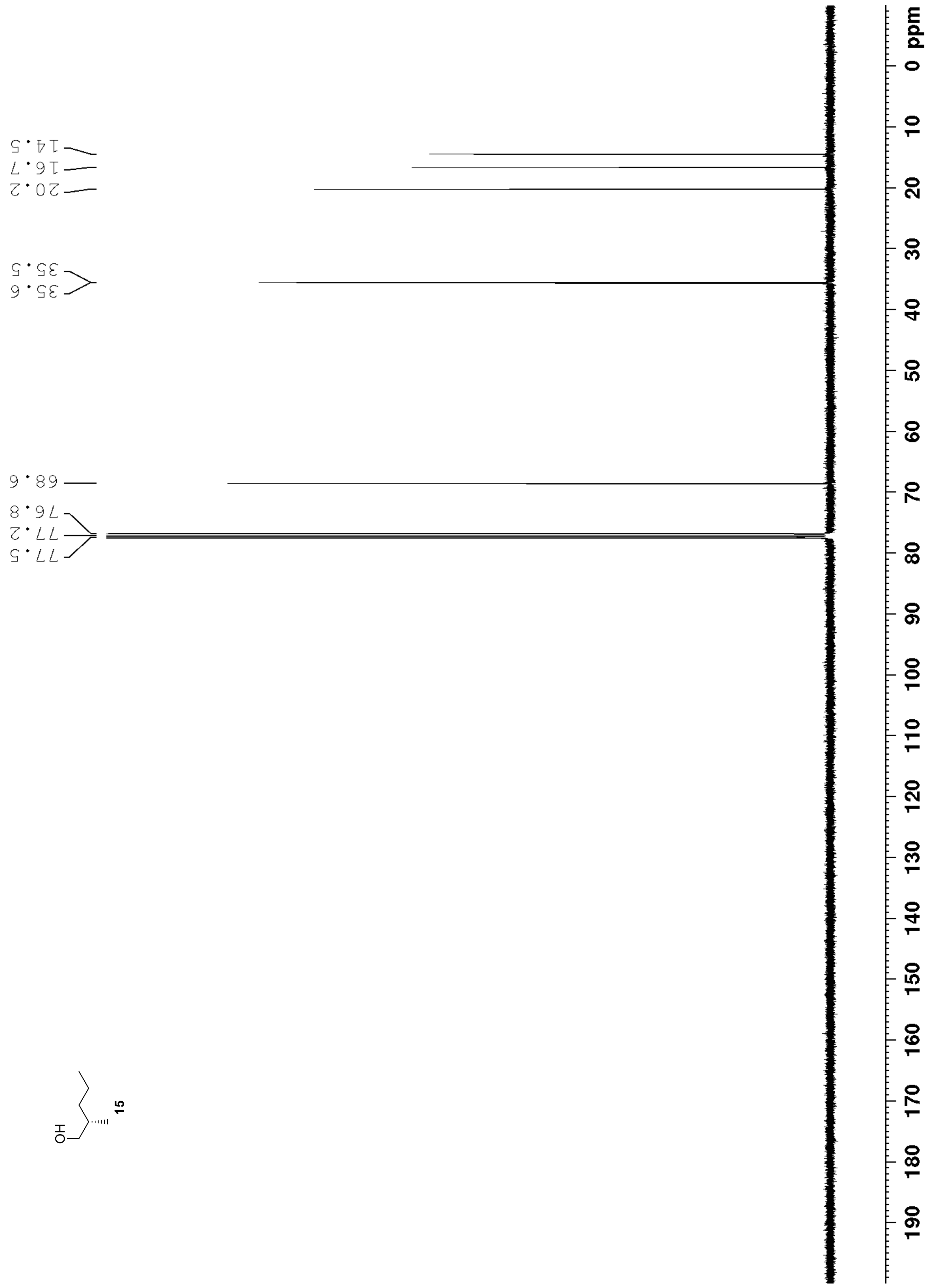
14.5
16.7
20.2
35.5
35.6
68.6
76.8
77.2
77.5
OH
15
ppm

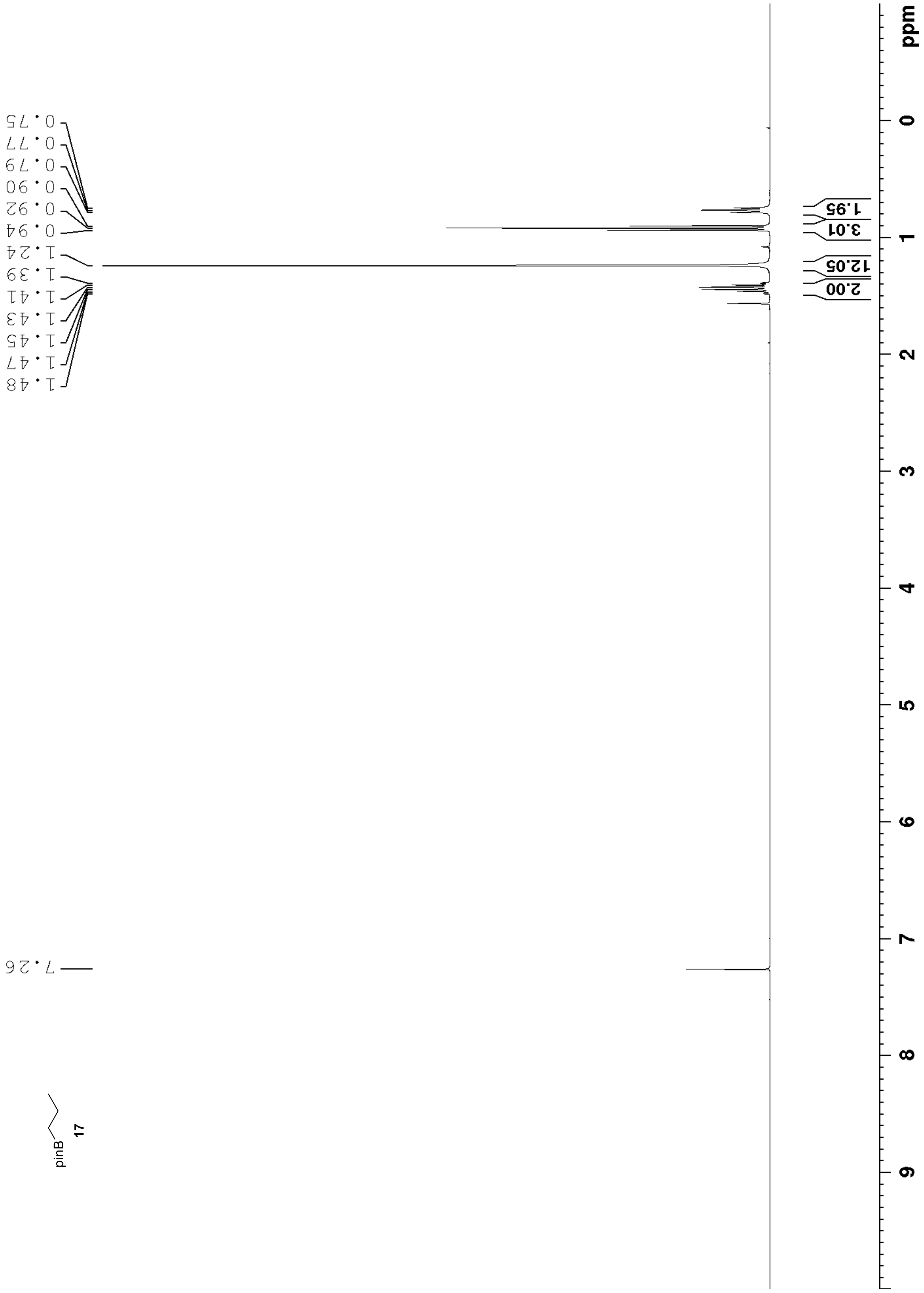
0.75
0.77
0.79
0.90
0.92
0.94
1.24
1.39
1.41
1.43
1.45
1.47
1.48
7.26
1.95
3.01
12.05
2.00
ppm
0
1
2
3
4
5
6
7
8
9
pinB
17

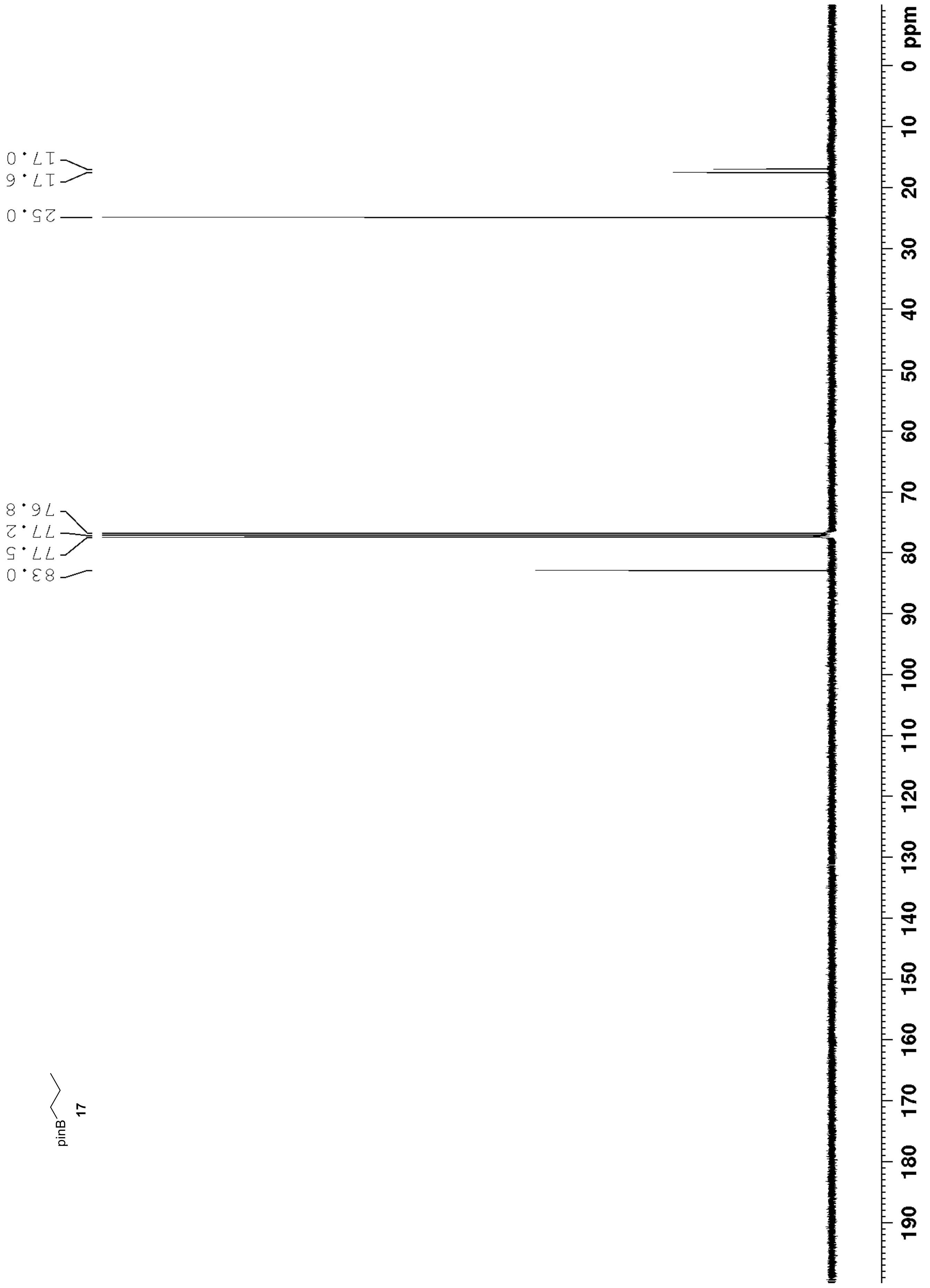
17.0
17.6
25.0
76.8
77.2
77.5
83.0
pinB
17
ppm
0
10
20
30
40
50
60
70
80
90
100
110
120
130
140
150
160
170
180
190

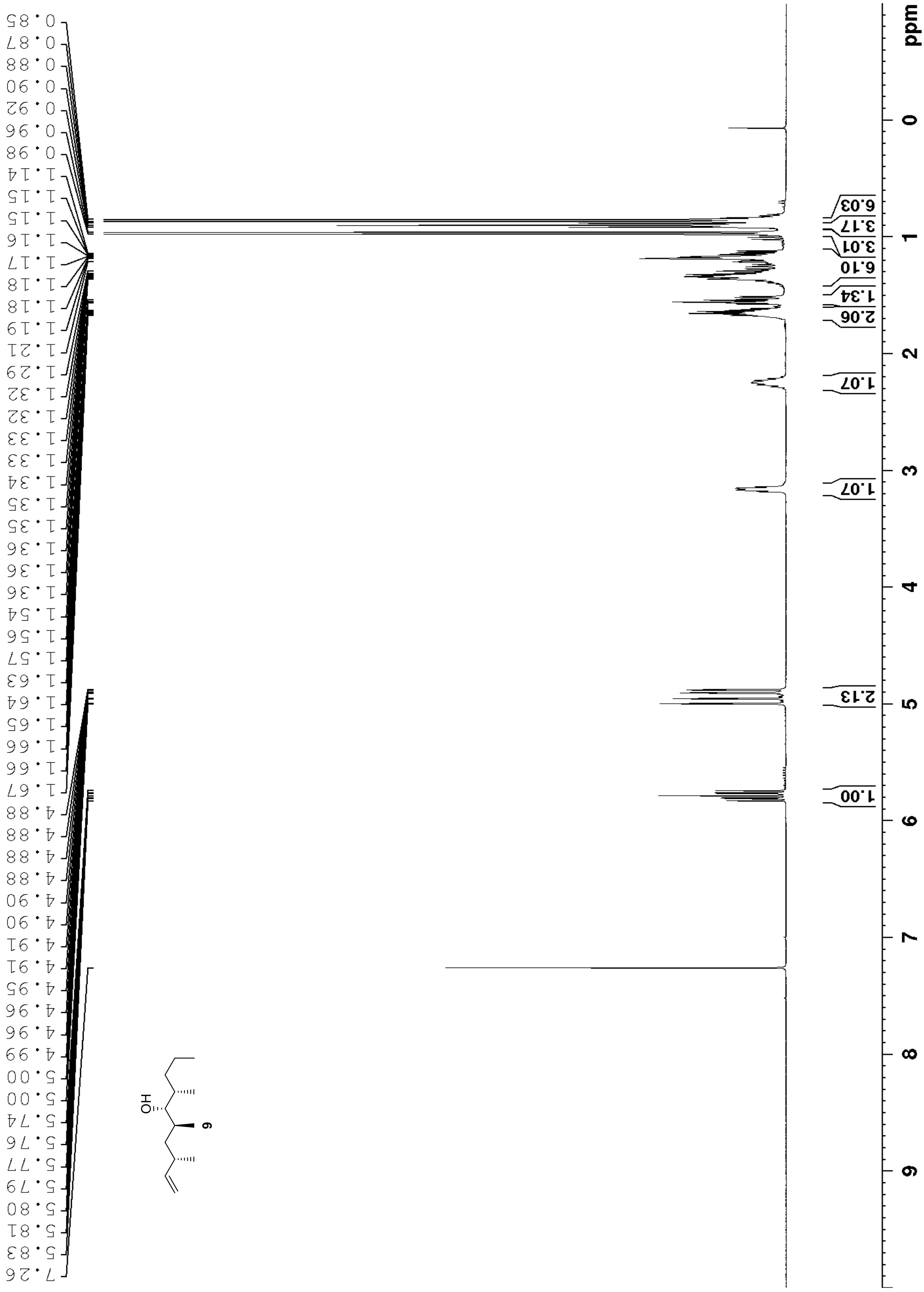
0.85
0.87
0.88
0.90
0.92
0.96
0.98
1.14
1.15
1.15
1.16
1.17
1.18
1.18
1.19
1.21
1.29
1.32
1.32
1.33
1.33
1.34
1.35
1.35
1.36
1.36
1.36
1.54
1.56
1.57
1.63
1.64
1.65
1.66
1.66
1.67
4.88
4.88
4.88
4.88
4.90
4.90
4.91
4.91
4.95
4.96
4.96
4.99
5.00
5.00
5.74
5.76
5.77
5.79
5.80
5.81
5.83
7.26
OH
6
6.03
3.17
3.01
6.10
1.34
2.06
1.07
1.07
2.13
1.00
ppm
0
1
2
3
4
5
6
7
8
9

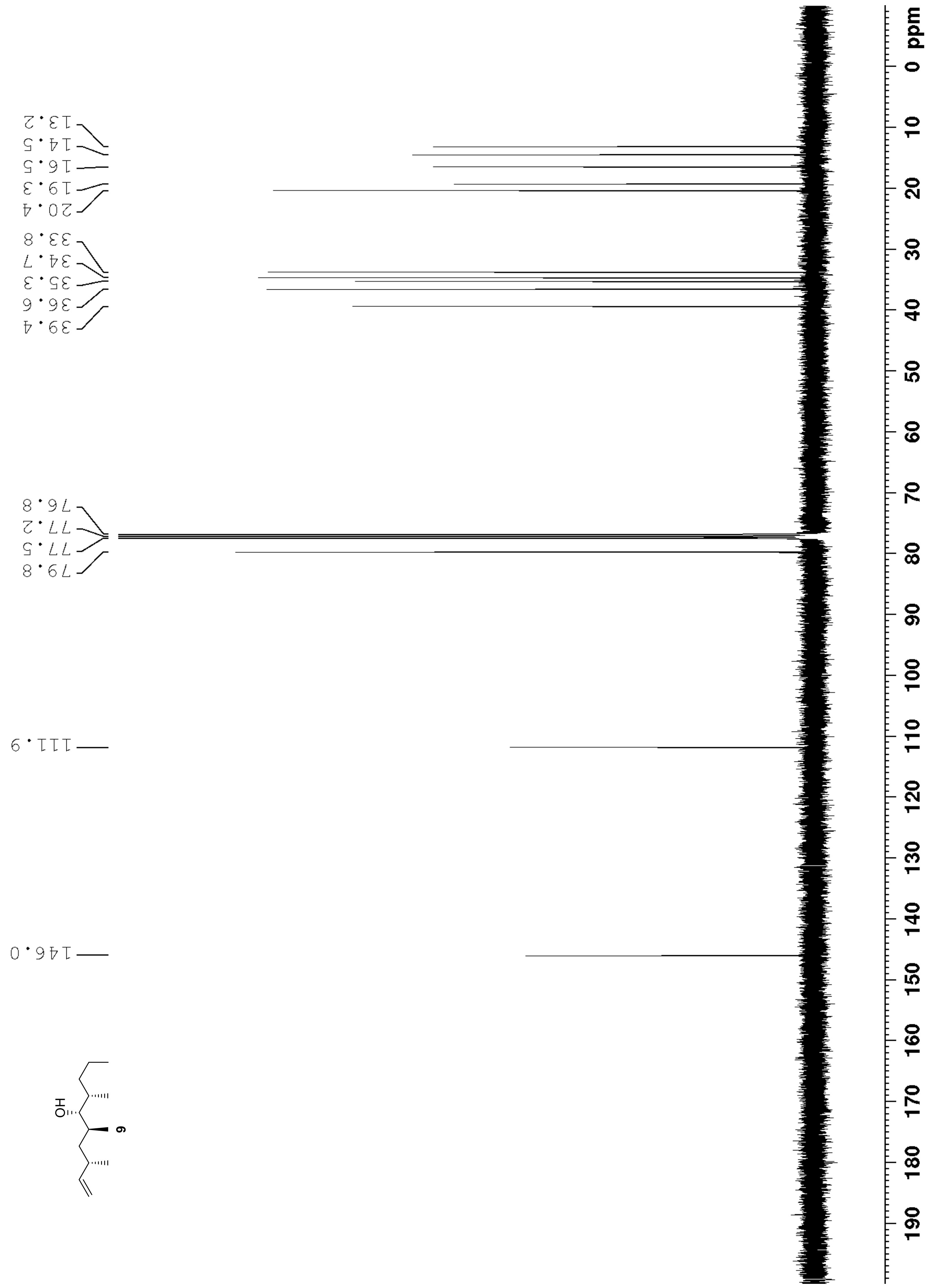
13.2
14.5
16.5
19.3
20.4
33.8
34.7
35.3
36.6
39.4
76.8
77.2
77.5
79.8
111.9
146.0
OH
9
ppm

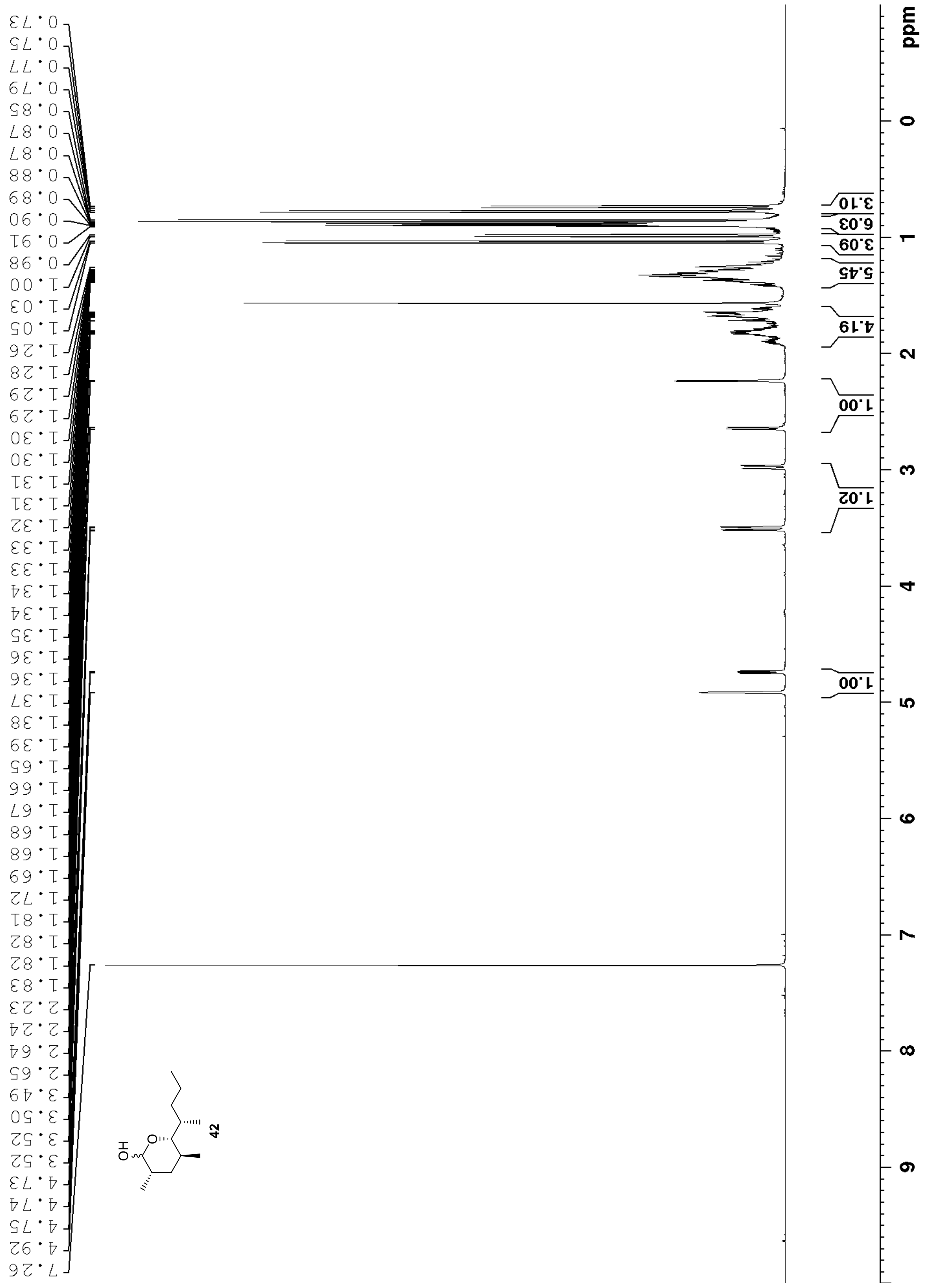
0.73
0.75
0.77
0.79
0.85
0.87
0.87
0.88
0.89
0.90
0.91
0.98
1.00
1.03
1.05
1.26
1.28
1.29
1.29
1.30
1.30
1.31
1.31
1.32
1.33
1.33
1.34
1.34
1.35
1.36
1.36
1.37
1.38
1.39
1.65
1.66
1.67
1.68
1.68
1.69
1.72
1.81
1.82
1.82
1.83
2.23
2.24
2.64
2.65
3.49
3.50
3.52
3.52
4.73
4.74
4.75
4.92
7.26
3.10
6.03
3.09
5.45
4.19
1.00
1.02
1.00
ppm
0
1
2
3
4
5
6
7
8
9
HO
O
42

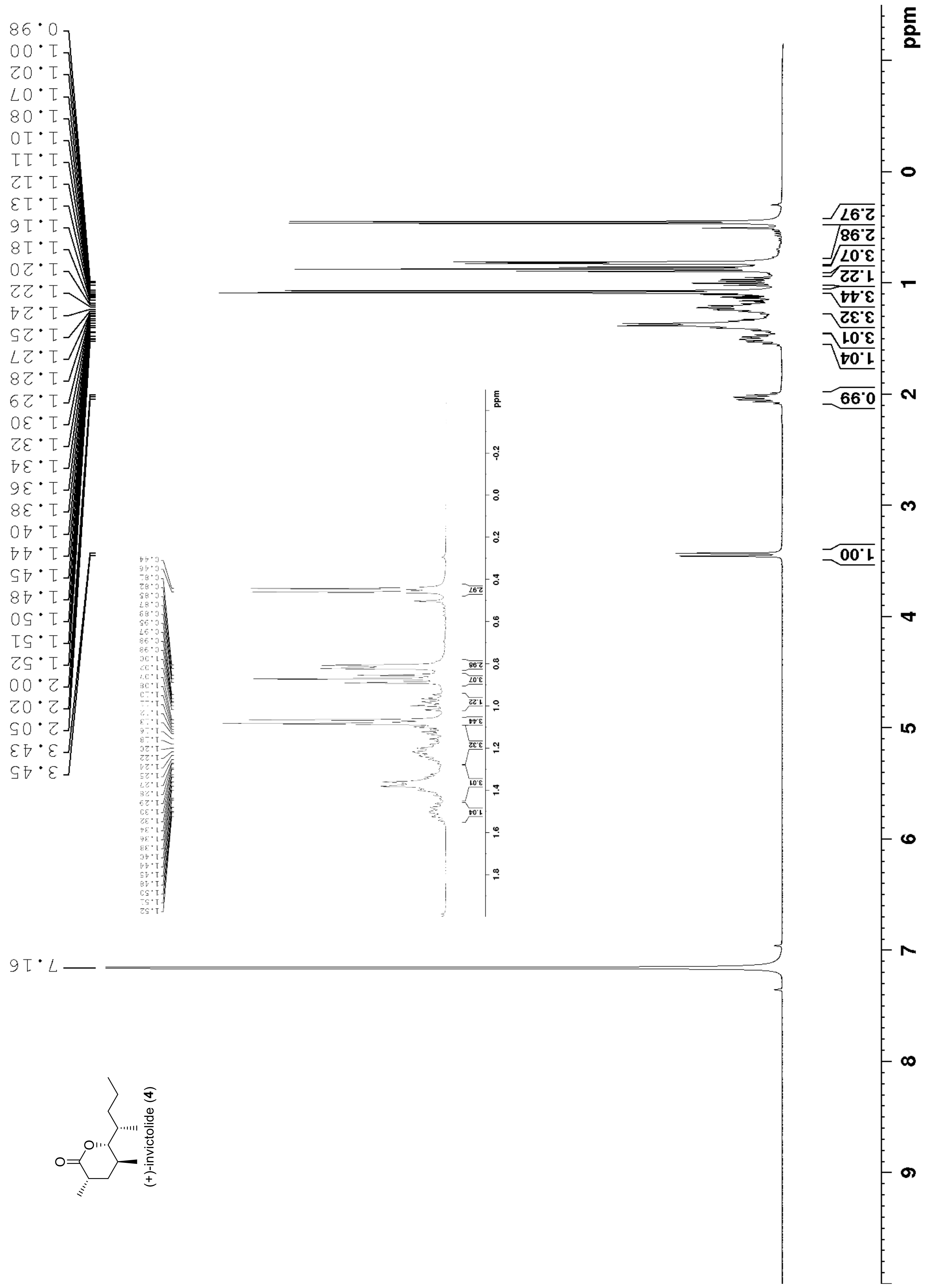
(+)-invictolide (4)

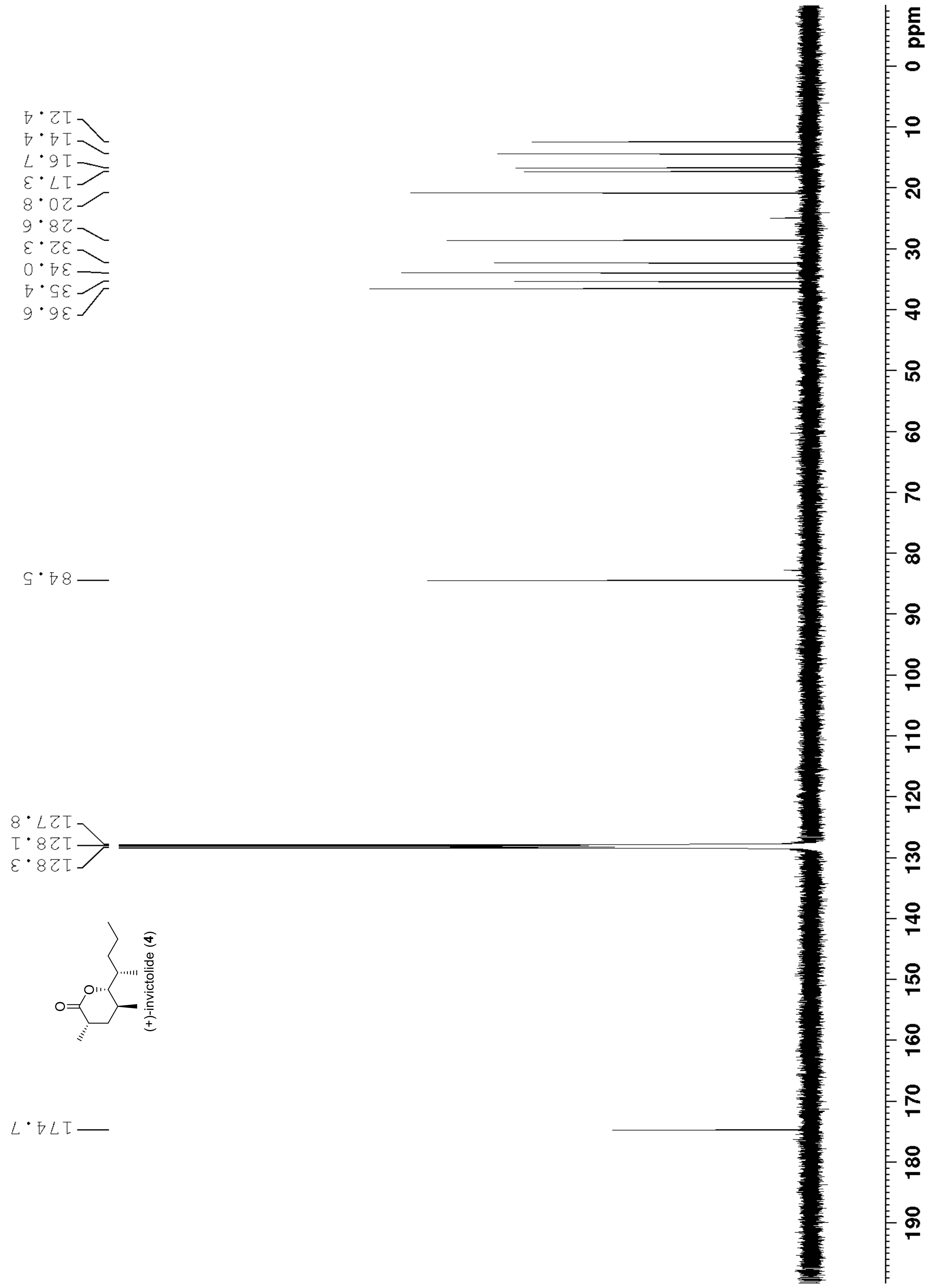
12.4
14.4
16.7
17.3
20.8
28.6
32.3
34.0
35.4
36.6
84.5
127.8
128.1
128.3
174.7
(+)-invictolide (4)
ppm

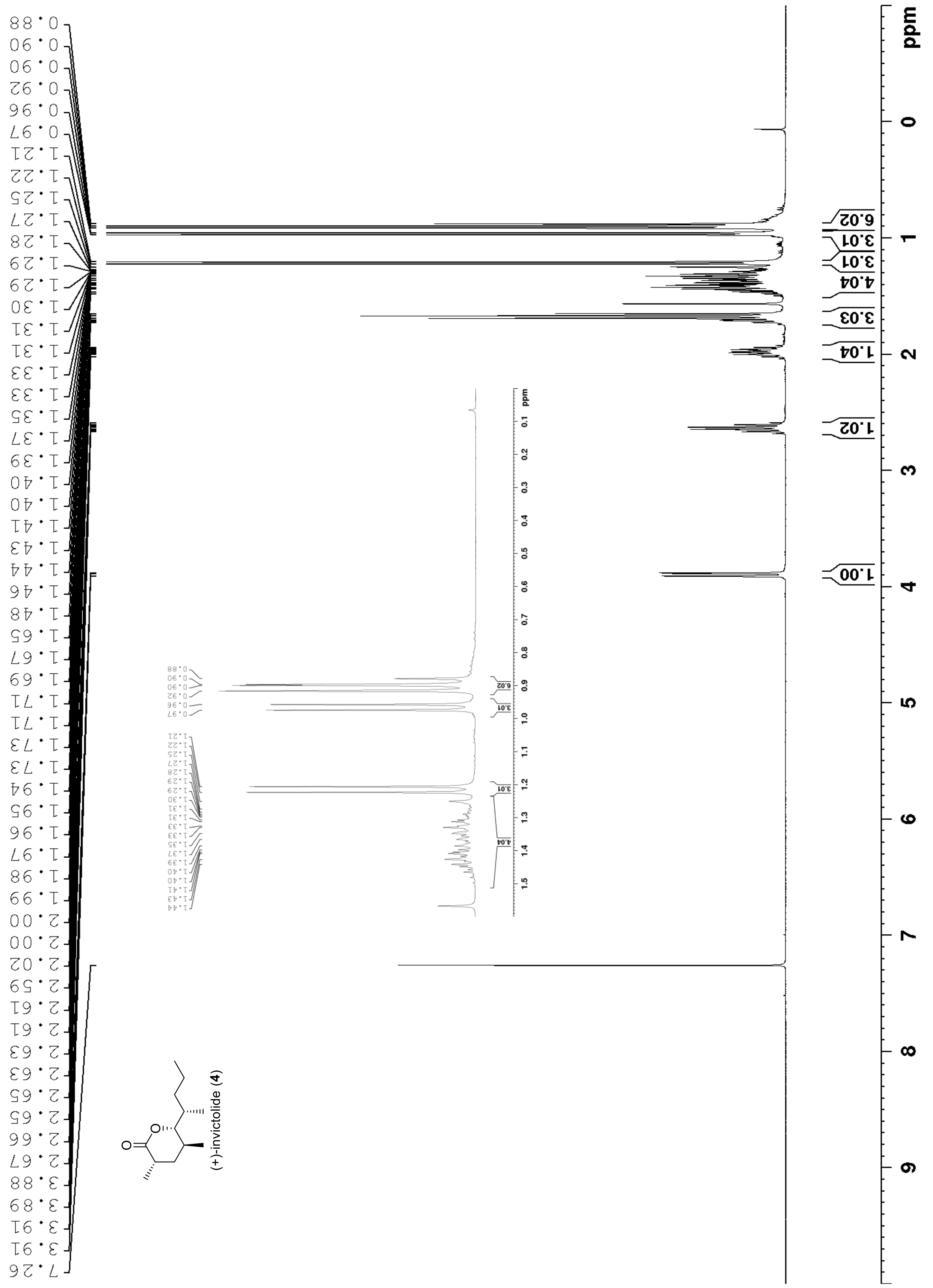
(+)-invictolide (4)
ppm

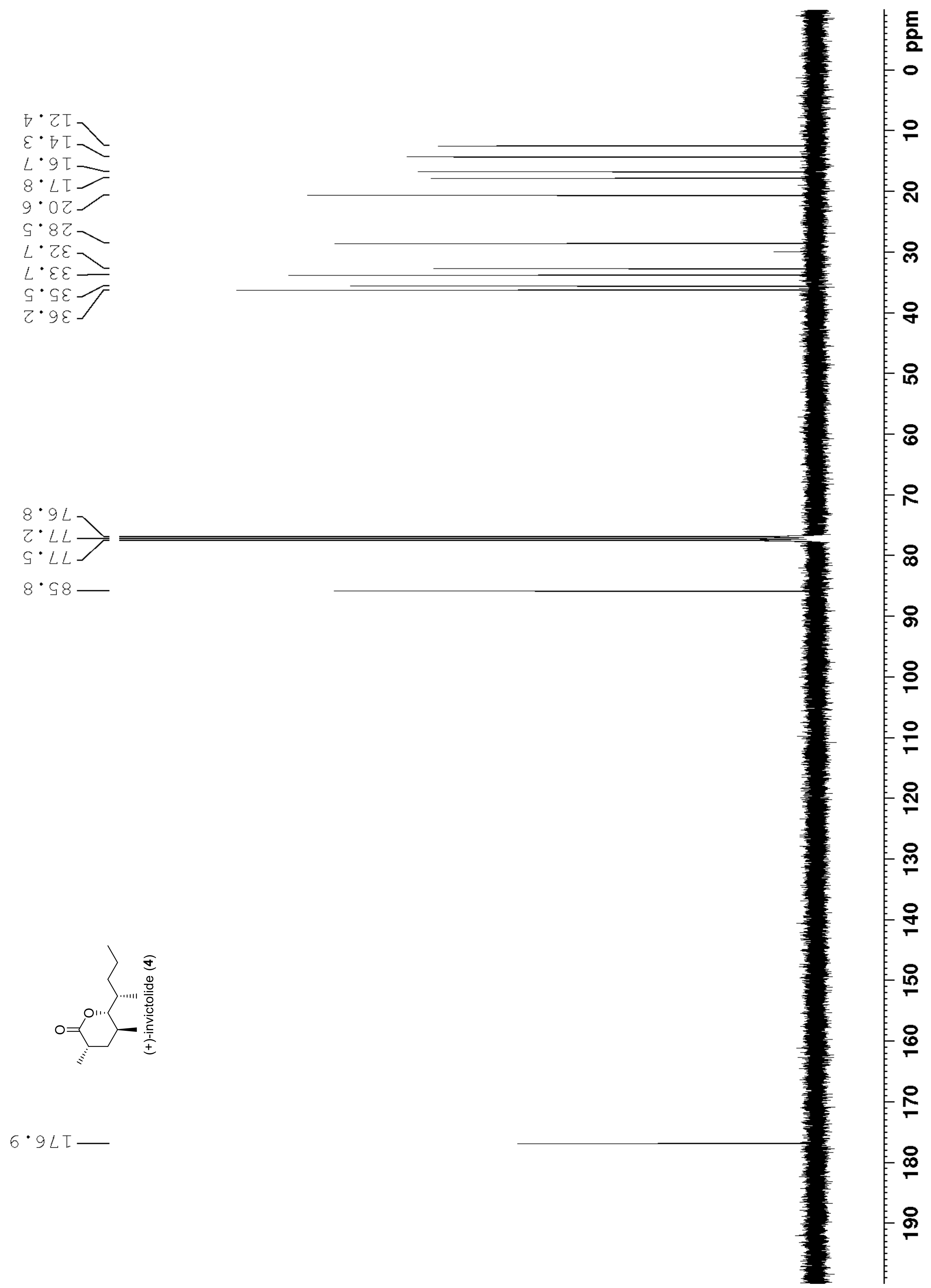
12.4
14.3
16.7
17.8
20.6
28.5
32.7
33.7
35.5
36.2
76.8
77.2
77.5
85.8
176.9
(+)-invictolide (4)
ppm

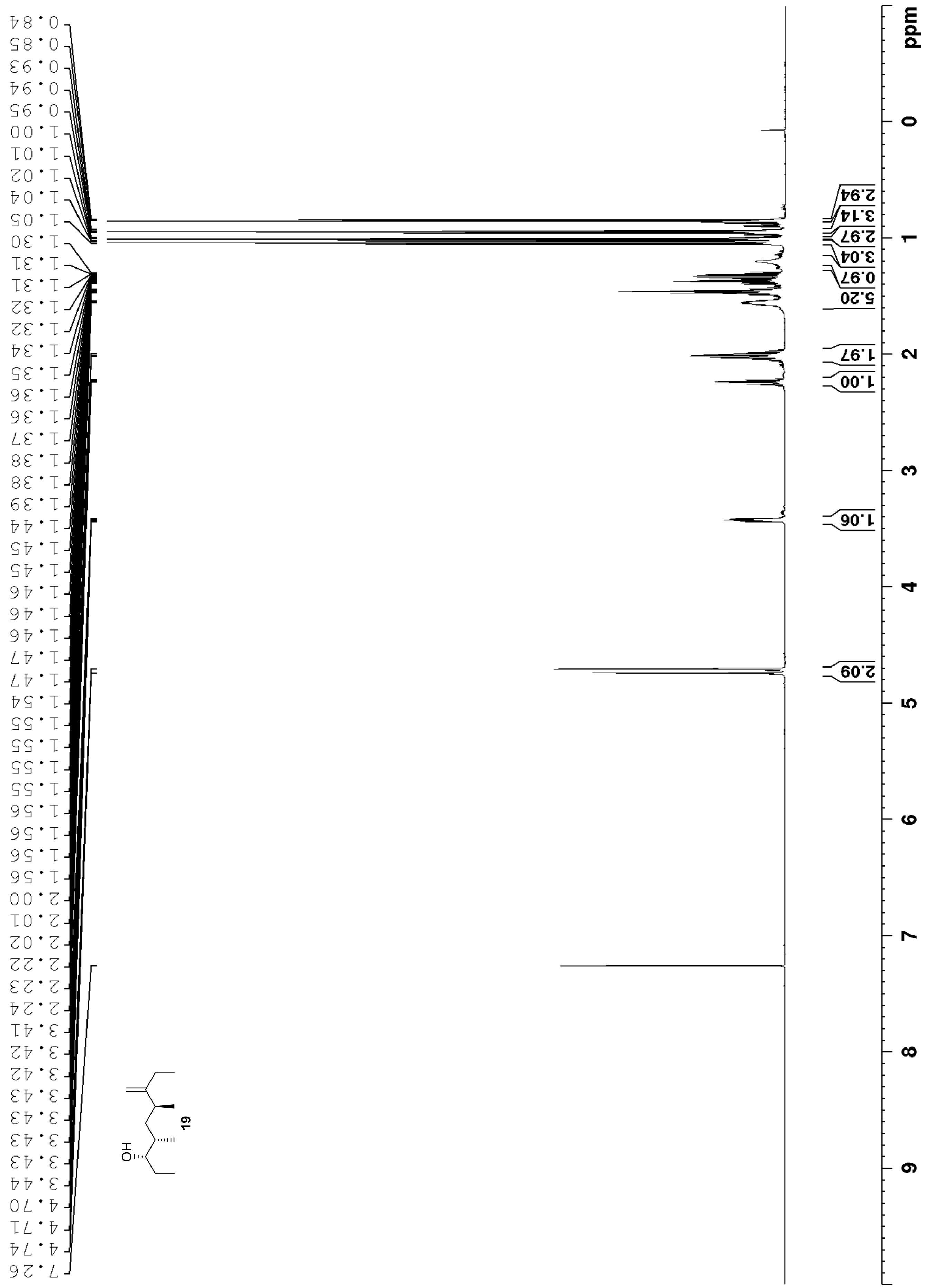
0.84
0.85
0.93
0.94
0.95
1.00
1.01
1.02
1.04
1.05
1.30
1.31
1.31
1.32
1.32
1.34
1.35
1.36
1.36
1.37
1.38
1.38
1.39
1.44
1.45
1.45
1.46
1.46
1.46
1.47
1.47
1.54
1.55
1.55
1.55
1.55
1.56
1.56
1.56
1.56
2.00
2.01
2.02
2.22
2.23
2.24
3.41
3.42
3.42
3.43
3.43
3.43
3.43
3.44
4.70
4.71
4.74
7.26
OH
19
2.94
3.14
2.97
3.04
0.97
5.20
1.97
1.00
1.06
2.09
ppm
0
1
2
3
4
5
6
7
8
9

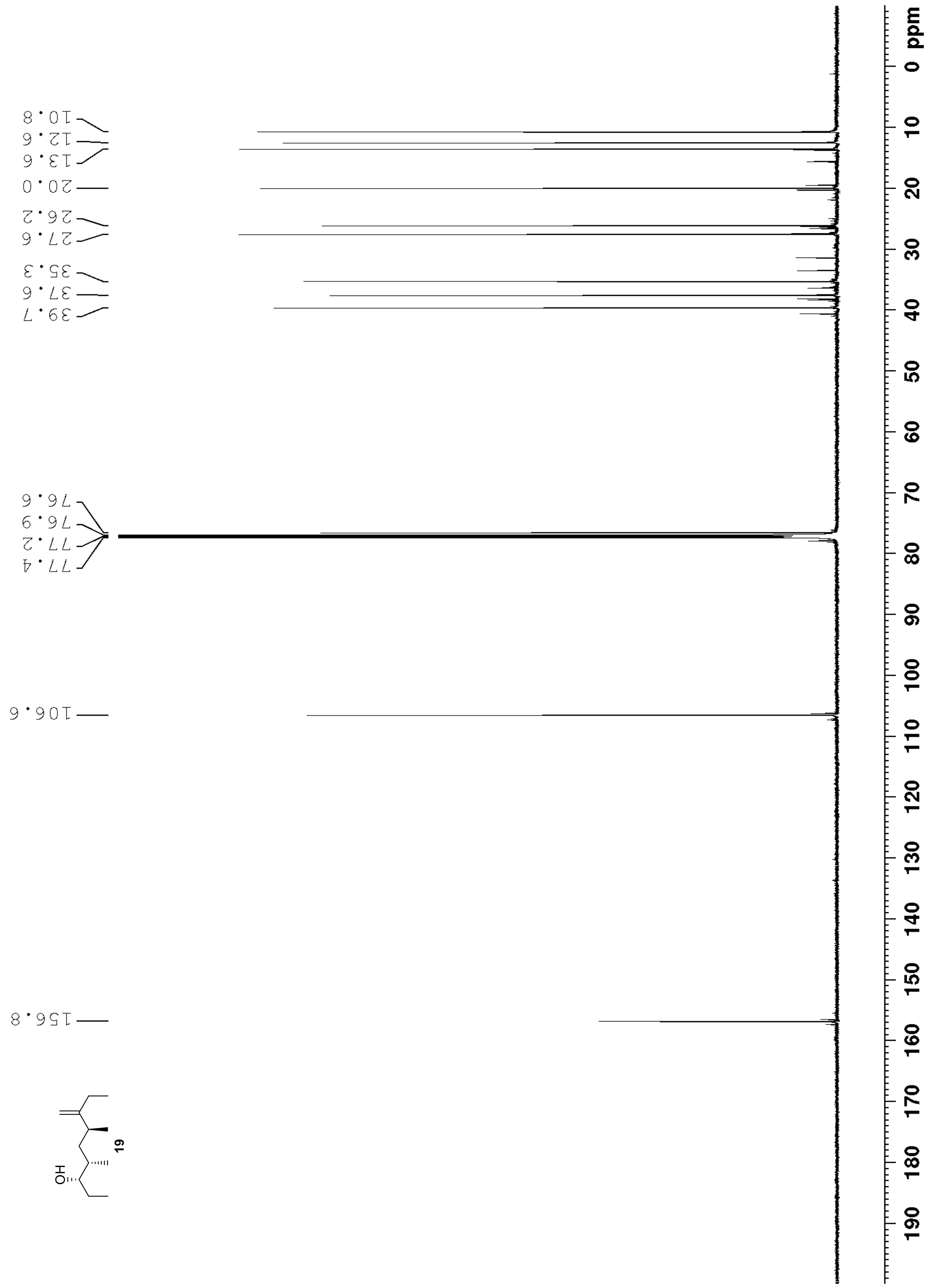
10.8
12.6
13.6
20.0
26.2
27.6
35.3
37.6
39.7
76.6
76.9
77.2
77.4
106.6
156.8
OH
19
ppm

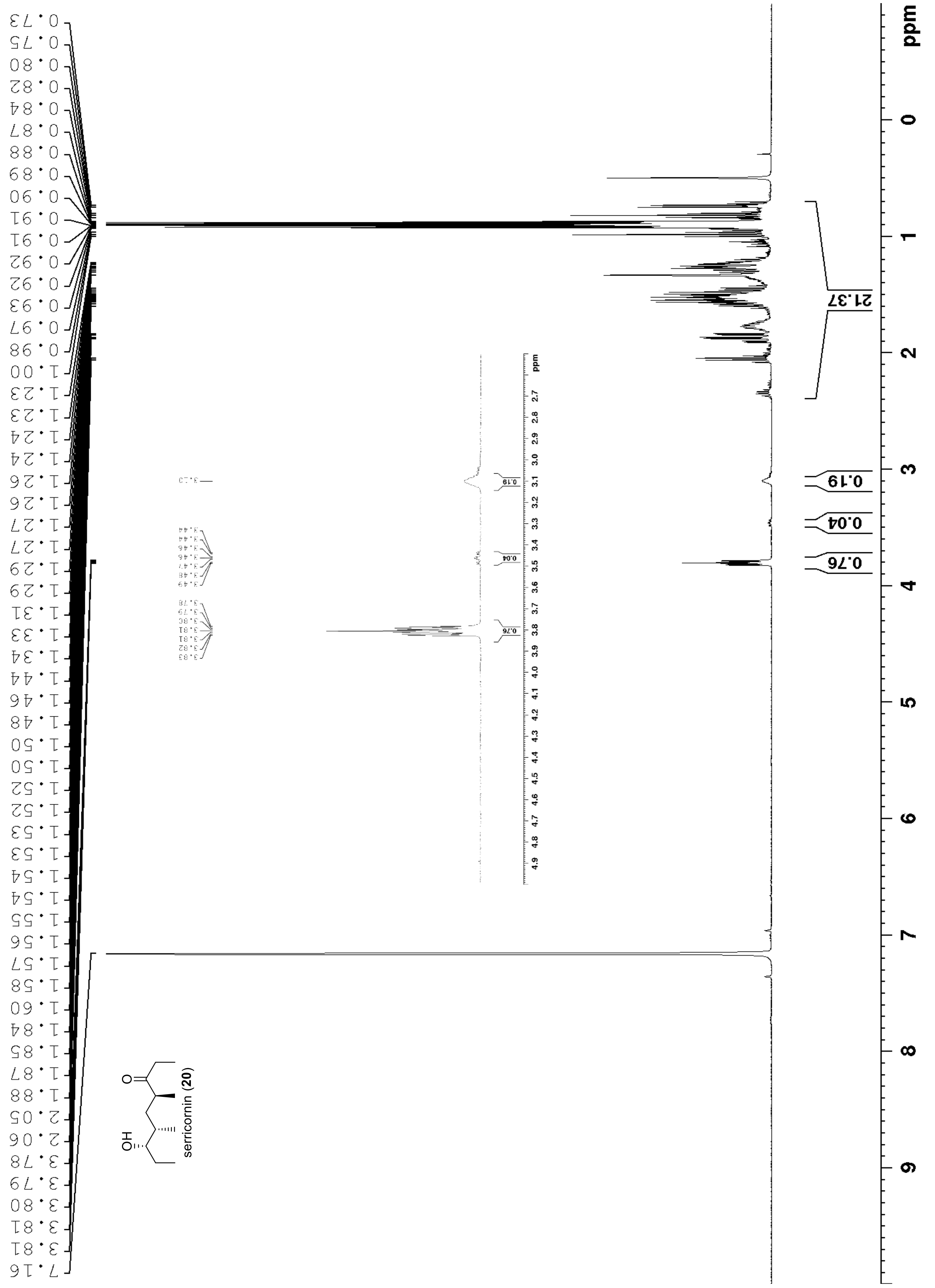
0.73
0.75
0.80
0.82
0.84
0.87
0.88
0.89
0.90
0.91
0.91
0.92
0.92
0.93
0.97
0.98
1.00
1.23
1.23
1.24
1.24
1.26
1.26
1.27
1.27
1.29
1.29
1.31
1.33
1.34
1.44
1.46
1.48
1.50
1.50
1.52
1.52
1.53
1.53
1.54
1.54
1.55
1.56
1.57
1.58
1.60
1.84
1.85
1.87
1.88
2.05
2.06
3.78
3.79
3.80
3.81
3.81
7.16
21.37
0.19
0.04
0.76
ppm
serricornin (20)

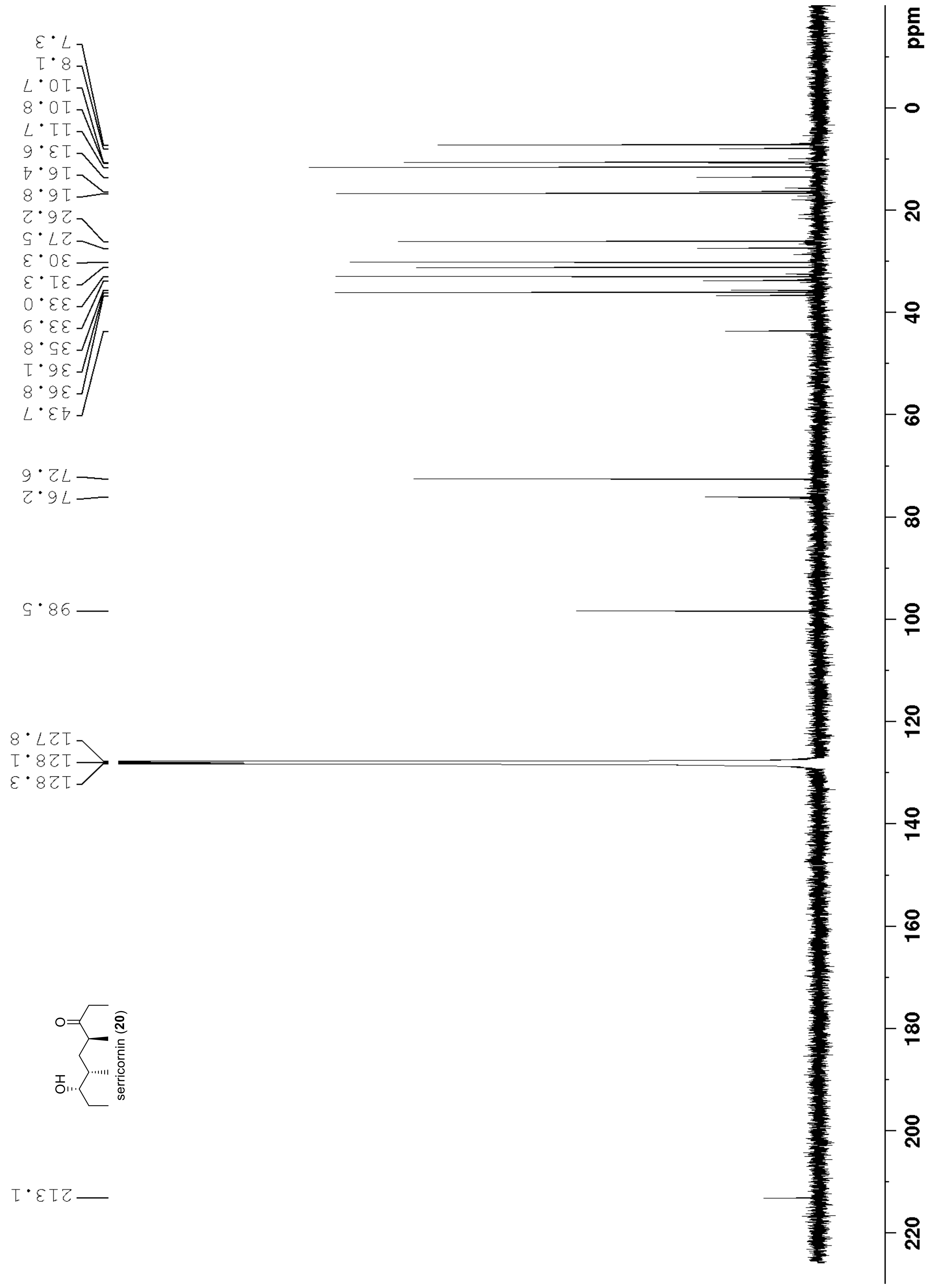
7.3
8.1
10.7
10.8
11.7
13.6
16.4
16.8
26.2
27.5
30.3
31.3
33.0
33.9
35.8
36.1
36.8
43.7
72.6
76.2
98.5
127.8
128.1
128.3
213.1
OH
O
sericornin (20)
ppm
0
20
40
60
80
100
120
140
160
180
200
220
